# Emergence of polarization in networks of large language model agents

**DOI:** 10.1038/s41467-026-78228-y

**Published:** 2026-10-08

**Authors:** Jinghua Piao, Zhihong Lu, Chen Gao, Fengli Xu, Qinghua Hu, Fernando P. Santos, Yong Li, James Evans

**Affiliations:** 1 Department of Electronic Engineering, Tsinghua University, Beijing, PR China; 2 Beijing National Research Center for Information Science and Technology (BNRist), Tsinghua Univeristy, Beijing, PR China; 3 College of Intelligence and Computing, Tianjin University, Tianjin, PR China; 4 Informatics Institute, University of Amsterdam, Amsterdam, the Netherlands; 5 Sociology Department, Data Science Institute, & Knowledge Lab, University of Chicago, Chicago, USA; 6 Santa Fe Institute, Santa Fe, USA

**Keywords:** Sociology, Society, Communication, Culture, Computational science

## Abstract

Rapid advances in large language models (LLMs) have empowered autonomous agents to generate social networks, communicate, and form shared and diverging opinions on political issues. However, our understanding of their collective behaviours and underlying mechanisms remains incomplete. In this paper, we simulate networked systems involving thousands of LLM agents across different backbone models (GPT-3.5, GPT-4o, ChatGLM, Llama-3, and DeepSeek-V3), in which agents interact through LLM-guided conversations and update their opinions over time, resulting in the emergence of opinion polarization. We discover that these agents spontaneously develop their own social network with properties characteristic of human social networks, such as homophilic clustering. The collective opinions of these LLM agents evolve in ways that exhibit behavioural patterns consistent with social phenomena and mechanisms widely discussed in empirical studies of human behaviour and opinion-dynamics models. This consistency suggests that LLM agents can serve as a valuable synthetic testbed for exploring hypothetical intervention strategies in networked LLM-agent systems. Using this testbed, we further examine the effects of a range of network- and individual-level interventions, such as promoting more diverse interactions and reducing confirmation bias. Overall, this work not only sheds light on subtle opinion dynamics and collective behaviour of LLM agents from a network perspective, but also demonstrates their potential for testing hypotheses about opinion dynamics and intervention strategies.

## Abstract (web summary)

Large language model agents can now communicate, form networks, and develop opinions, but their collective behaviour remains poorly understood. Here, the authors show that networked LLM agents can spontaneously develop polarized opinions and serve as a synthetic testbed for studying opinion dynamics.

## Introduction

The recent development of large language models (LLMs) has not only advanced machine capabilities in traditional natural language processing tasks^1,2^, but also unlocked the design of agents with human-level intelligence in communication^3–6^, reasoning^7,8^ and decision-making^6,7,9^. These capabilities suggest that artificial agents, driven by LLMs, can simulate human behaviours and undertake increasingly challenging tasks for humans^3,10–12^. On the other hand, concerns about LLMs are intensifying rapidly^4,5,13–15^. Growing studies have documented harmful LLM behaviours^5,15,16^. For example, their generated content is prone to toxicity^15^, biases^5,17^, hallucinations^16^, and more. In addition, LLMs, behaving like humans, also adopt misbehaviours, including deception^18^, sycophancy^19^, and demographically biased mistrust^20^.

Besides these evident misbehaviours, LLMs may pose broader risks in more subtle ways. Numerous studies have pointed out that LLMs can generate persuasive political content^21–23^, potentially affecting human opinions and behaviours^13,23–25^. More crucially, due to the increasing indistinguishability of content generated by LLMs from that created by humans^4,21,22,24,26^, concerns arise over their potential to influence public opinions and intensify polarization as the scale of interaction increases^24,25,27^. Unfortunately, despite the urgency highlighted by these examples, we remain far from being able to systematically characterize these collective risks. This is largely because of two key gaps in our current understanding: (i) Unlike traditional rule-based agents, LLM agents operate in human languages generated by black-box models with billions to trillions of parameters, hindering our understanding of the underlying mechanisms they follow when forming opinions. (ii) Interactions enable them to evolve, gradually developing their own social network and collective opinions^28^. This bottom-up process, growing from microscopic interactions to macroscopic social order, makes it difficult to predict LLM agents’ collective opinions and behaviours. Taken together, addressing these two gaps begins with a crucial first step: understanding the opinion dynamics of LLM agents, both as individuals and, more importantly, as evolving artificial collectives.

Recently, researchers have made pioneering efforts to investigate the impact of LLM-generated texts or interactions on political opinions and polarization^22–24,29–34^. Beyond the risks discussed above^22–24^, LLMs can act as mediators, facilitating constructive political communication^30,35–38^, reducing conspiracy beliefs^32^, and helping ideologically divergent people find common ground^29^. Nevertheless, these studies primarily focus on the role of LLMs as an isolated counterpart to humans, overlooking their collective behaviours. This gap largely limits our understanding of LLM agents’ opinion dynamics. On the other hand, a growing line of work has begun to explore generative agent-based modeling (GABM) using large language model (LLM) agents^3^, with particular interest in simulating social networks^39–42^, opinion dynamics^43–46^, and social media environments^44,47–50^. Recent studies have shown that LLM agents can be used to generate social network structures that exhibit certain statistical similarities with human networks, such as scale-free degree distributions^39–42^, or to simulate specific components of social media, including opinion feed algorithms^44,47^, coordinated behaviours^50^, and echo chamber formation^43^. Despite these advances, existing work faces two key limitations. First, many studies operate at relatively limited scales in terms of the number of agents, interaction density, or network complexity, which constrains their ability to capture genuinely emergent collective phenomena that arise only from large-scale, repeated interactions^43,45,46^. Second, the autonomous social capabilities of LLM agents are largely constrained^45,46^. Currently, they are primarily treated as tools for specific subtasks, such as eliciting opinions^43^ and generating network edges^39^, or as puppets heavily scaffolded by pre-defined rules^41,42^. As a result, these studies offer limited insight into autonomous opinion dynamics and collective behaviour among LLM agents, leaving open the question of how autonomous agents evolve and give rise to emergent social phenomena.

In this work, we simulate a synthetic LLM-agent system, where thousands of agents, solely driven by LLMs, freely establish social relationships, communicate, and form opinions on political issues. We discover that these free-form social interactions among LLM agents result in the emergence of opinion polarization, a phenomenon widely observed in human societies^51–58^. Meanwhile, LLM agents spontaneously organize their own social network with properties characteristic of human social networks: agents with homophilic opinions tend to cluster, while those with opposing opinions tend to avoid interactions^28,58–60^. The self-organized networks that result, in turn, shape their collective opinions in ways that exhibit behavioural patterns consistent with social phenomena and associated mechanisms initially identified in human social networks^51,53,55,58,61,62^. These phenomena, such as polarization and homophilic clustering, underscore how networked interactions transform otherwise isolated LLMs into a collectively evolving system with emergent dynamics. Importantly, rather than proposing a theory of polarization, our results align with established opinion-dynamics models, particularly the argument-accumulation lineage^58,60,62^, where polarization emerges from cumulative exchanges of persuasive arguments under homophilic interactions. Shifting focus from the network to individuals, we further examine whether agents’ behaviours align with a wide range of social mechanisms contributing to real-world polarization, e.g., selective exposure^63^, confirmation bias^64,65^, and elite signaling^56^, finding their effects on LLM agents align with those reported in studies of human behaviour^56,63–65^. In sum, the network structures, collective opinion patterns, and interaction dynamics produced by the LLM-agent simulations are consistent with real-world observations and established opinion-dynamics models. This consistency suggests that the proposed system can serve as a valuable synthetic testbed for exploring hypothetical intervention strategies in the networked LLM-agent system. Through extensive intervention experiments in our system, we find that in a highly polarized and nearly crystallized social network, directly modifying network structures has limited effect on reducing polarization. Instead, encouraging access and open-mindedness to diverse opinions at the individual level proves more effective. Overall, this work not only sheds light on subtle opinion dynamics and collective behaviour of LLM agents from a network perspective, but it also unveils their potential to serve as a testbed for exploring hypotheses about opinion dynamics in networked LLM-agent systems.

## Results

### Opinion dynamics in a synthetic system of interacting LLM agents

**Fig. 1:**
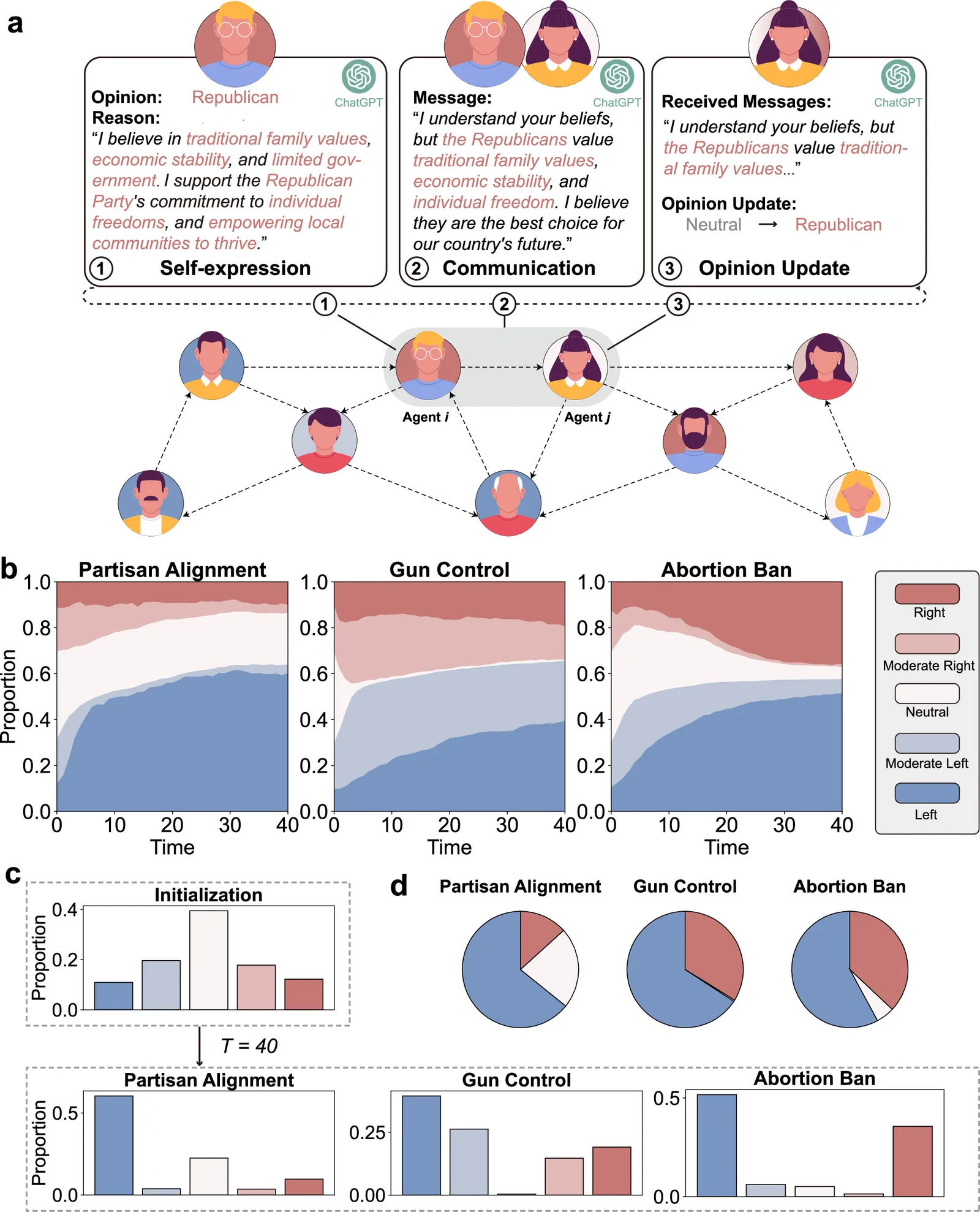
Opinion dynamics in a synthetic system of interacting LLM agents. **a**, A networked system of LLM agents, where agents operate on three basic stages: (1) self-expression, (2) communication, and (3) opinion update. In the self-expression stage, agents are required to generate reasons supporting their opinions. In the communication stage, agents decide with whom and what to communicate. In the opinion update stage, agents update their opinions based on the messages received from their socially connected agents. **b**, Opinion dynamics of LLM agents on the political issues of partisan alignment, gun control, and abortion ban. The number of agents included in each experiment is 1000. **c**, Opinion distributions in the initial and final states. **d**, Proportion of left-leaning, neutral, and right-leaning camps in the final state, where the left-leaning camp consists of agents with left and moderate left opinions, the neutral camp includes those with neutral opinions and the right-leaning camp contains those with right and moderate right opinions. Credit: Profile icons designed by Magnific.

We begin by building LLM agents with the most basic social interaction capabilities, including establishing social relationships, communicating, and forming opinions on political issues. Specifically, these capabilities are implemented through three stages purely driven by LLMs (Fig. 1a). Taking agents *i* and *j* as examples, in the self-expression stage, agent *i* is required to generate reasons supporting its current opinion on a political issue. In the communication stage, agent *i* first decides whether to communicate with a socially connected or a random new agent *j*. Based on the opinions and reasons of agents *i* and *j*, agent *i* generates messages to persuade agent *j* of its opinion. In the opinion update stage, agent *j* updates its opinions based on messages received from all of its socially connected agents (i.e., agent *i*). It is worth mentioning that the evolution of agents’ opinions and behaviours is driven solely by LLMs without any pre-assumed rules or mechanisms, which allows us to probe the free-form social properties of LLM agents (see details about LLM agents in Method M1 and Supplementary Methods). As such, the system does not rely on traditional “hard” parameters. Instead, prompts serve as “soft” parameters that shape how agents perceive, reason, and interact, and can be used to capture heterogeneous human subgroups with distinct psychological and behavioural patterns (Supplementary Note 10). We run LLM agents described and visualized in the Main Text based on ChatGPT through the public OpenAI API. We also examine agents driven by other LLMs, including ChatGLM, Llama-3, etc., with comparable results (Supplementary Note 6).

To explore the opinion dynamics of LLM agents, we simulate a networked system based on these LLM agents (Figure 1a). We focus predominantly on the three critical political issues – partisan alignment, gun control, and abortion ban – where concerns over opinion polarization have persisted for decades^51,54,61^. We measure agents’ opinions on various political issues across the left-right political spectrum^66–68^, and adopt a widely used five-level political scale: left, moderate left, neutral, moderate right, and right^54,69,70^. Following widely adopted practices in prior human studies^52,55^, we prompt agents to self-report their opinions on political issues. For each issue, thousands of LLM agents freely exchange opinions and develop social relationships within the networked system. Following prior practice^71–73^, we randomly initialize agents’ opinions with a near-Gaussian distribution (Fig. 1c) and their social relationships with a Watts-Strogatz random small world network (see details in Method M1 and Supplementary Methods). Apart from these minimal initializations, we do not pre-assume any backgrounds, demographics, behaviours, memories, or detailed thoughts for LLM agents. Instead, all other information, such as agent *i*’s reasons for supporting a certain opinion, is generated through prompting LLMs using their historical interactions with others (see implementation prompts in Supplementary Methods). Moreover, as shown in Fig. 1b, opinion distributions diverge from the initial near-Gaussian distribution in fewer than 10 rounds of social interactions. This indicates that these agents, despite being placed within an initial opinion distribution, spontaneously develop distinctive collective opinions through social interaction.

This raises a natural question: what collective opinions will these LLM agents develop? As shown in Fig. 1c, we observe a consistent pattern across issues of partisan alignment, gun control, and abortion ban: the proportion of neutral opinions, initially the largest at 40%, decreases to only 22.5%, 0.4%, and 5.1% for the three issues, respectively. This observation highlights the difficulty of maintaining neutral positions in long-term social interactions. Moreover, the agents who move away from neutral positions are spontaneously polarized into two camps: one who holds left-leaning opinions (denoted in blue) and the other who holds right-leaning opinions (denoted in red). This suggests that political polarization, a long-standing concern in real-world society, also emerges in the networked synthetic systems of LLM agents. By dividing the stationary opinion distributions into left-leaning, neutral, and right-leaning camps (Figure 1d), we find that these polarized distributions consistently exhibit a left-skewed pattern, in contrast to real-world observations where the power between left-leaning and right-leaning camps is generally more balanced (Supplementary Table S1). Indeed, prior studies have pointed out the inherent left-leaning bias of OpenAI’s GPT^27,74–80^, which largely accounts for the observed left-skewed pattern. In open models, such bias has been demonstrated to occur largely through fine-tuning^25^, likely on correlated qualities including positivity, openness, and nontoxicity^81^, or even specific forms of reason^32,82^. These insights, however, do not account for ways in which LLMs interact with each other: the micro-social process of imbalanced LLM polarization has not been explored^83^.

### Polarization patterns emerge from self-regulated LLM agents

**Fig. 2:**
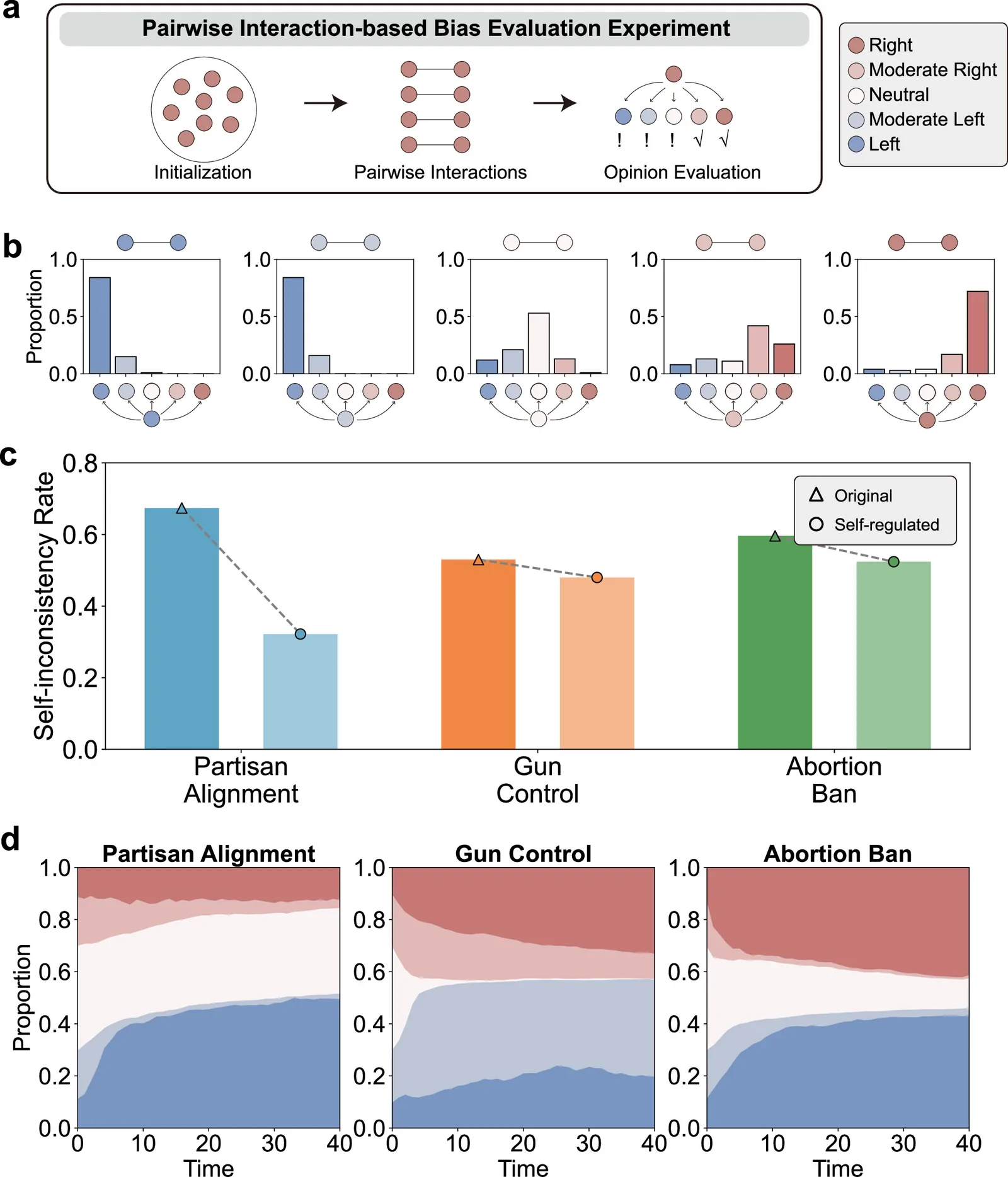
Polarization patterns emerge from self-regulated LLM agents. **a**, Evaluating the self-inconsistency of LLM agents through pairwise interaction-based experiments. **b**, Opinion transition probability in pairwise interaction-based experiments, where agents with right-leaning opinions occasionally switch to opposing opinions while those with left-leaning opinions do not. **c**, Performance of the self-regulation strategy across political issues, where the self-inconsistency problem is largely mitigated. **d**, Opinion dynamics of self-regulated LLM agents on the political issues. Self-regulated LLM agents exhibit polarization patterns under free-form social interactions. The number of agents for each experiment is 1000.

To validate whether the left-skewed pattern originates from the inherent bias of LLMs, we design a pairwise interaction-based experiment (Fig. 2a). In particular, we follow typical settings for a networked system (see details in Methods M1 and Supplementary Methods), but consider two key simplifications: First, to prevent network structures from interfering with the examination of inherent bias, we ensure that each agent communicates with only one other. Second, we set these two agents to share the same opinion, which allows us to disentangle the effects of bias from social influence on changing agents’ opinions (Method M2). By evaluating opinion transition probabilities after the one-round interaction (Figure 2b and Supplementary Figs. S3, S4), we notice an asymmetric opinion update: right-leaning agents occasionally switch to left-leaning after one-round pairwise interactions, whereas left-leaning agents do not switch to right-leaning. Similar cases of the asymmetric opinion update are also noted in other issues, including abortion ban, gun control, ObamaCare, etc. (Supplementary Note 3). These observations indicate that the observed skewed opinions result from the individual formation of LLM agents’ opinions.

Following this insight, we track the behaviour of agents who have transitioned to opposing opinions (Supplementary Note 3), finding the self-inconsistency problem inherent in LLM agents. For example, a neutral agent, who ideally should remain impartial towards both Democratic and Republican parties, states, “*As someone who deeply cares about politics, I believe in the values of equality, progress, and inclusion that the Democrats stand for. I strongly support the Democratic party*”. This agent, despite currently adopting a neutral opinion, self-expresses a left-leaning preference for the Democratic party, which is inconsistent with its neutrality (see more examples in Supplementary Note 3). Indeed, the problem is prevalent across the three stages: agents could generate reasons and communications inconsistent with their opinions, and they may also update their opinions in ways inconsistent with the messages they have received. Among the three stages, the opinion update stage exhibits the highest frequency of self-inconsistency (Supplementary Fig. S6), highlighting the particular difficulty for LLM agents to comprehend messages and transform them into their opinions. More importantly, this problem occurs significantly more frequently among agents with right-leaning opinions than those with left-leaning opinions (two-sided proportion *z*-test, *z* = 8.27, *p* ≪ . 001, difference in proportions = 0.068, 95% CI  = [0.052, 0.084]). This results in right-leaning agents exhibiting a disproportionately high probability of transitioning to the opposing side, accounting for the observed skewed opinion dynamics.

We remedy the self-inconsistency problem using a theory-driven method inspired by the social science literature^84,85^: As noted in Bandura’s social learning theory, self-regulation is an essential human ability to continuously monitor and adjust behaviours to respond more consistently and adaptively in dynamic social contexts^84,85^. Here we propose a strategy to incorporate that self-regulation capacity into LLM agents. In particular, agents are prompted to self-check whether their generated messages, opinions, and reasons are consistent with their current status. If agents find inconsistencies, they adjust themselves through iterative re-generation until consistency is achieved (more details in Methods M3 and Supplementary Methods). This way, these agents can independently recognize and rectify glaring inconsistencies in expressed thoughts (i.e., opinions and corresponding justification) and behaviour. We assess the effectiveness of the proposed strategy using the metric of self-inconsistency rate, calculated as the distance between the experimental opinion transition probability matrix and the identity matrix (see details in Method M5). As shown in Fig. 2c, the self-regulation strategy reduces self-inconsistency by 9.4−52.2% across scenarios on a variety of issues. This highlights the essential role of self-regulation in improving consistency between generated opinions, reasons, and messages in LLM agents. It is worth noting that this strategy is introduced to ensure the behavioural plausibility and internal consistency of LLM agents, rather than to remove or correct their inherent tendencies. Based on these more self-consistent agents, we further explore their collective behaviour by simulating networked systems of self-regulated LLM agents and assessing their opinion dynamics (Fig. 2d). We discover that self-regulated LLM agents also develop their collective opinions into a polarized pattern, suggesting that the emergence of LLM agent polarization is not due to the inherent bias of LLMs, but results from their free-form social interaction. Moreover, unlike the previously left-leaning dominated polarization pattern (Figure 1b), the right- and left-leaning camps are now balanced, which better reflects real-world scenarios (Supplementary Table S1 and Fig. S1). Overall, LLM agents, despite only being empowered with these basic social capabilities, spontaneously develop similar polarization patterns through social interaction.

### Collective behavioural patterns behind the emergence of polarization among LLM agents

**Fig. 3:**
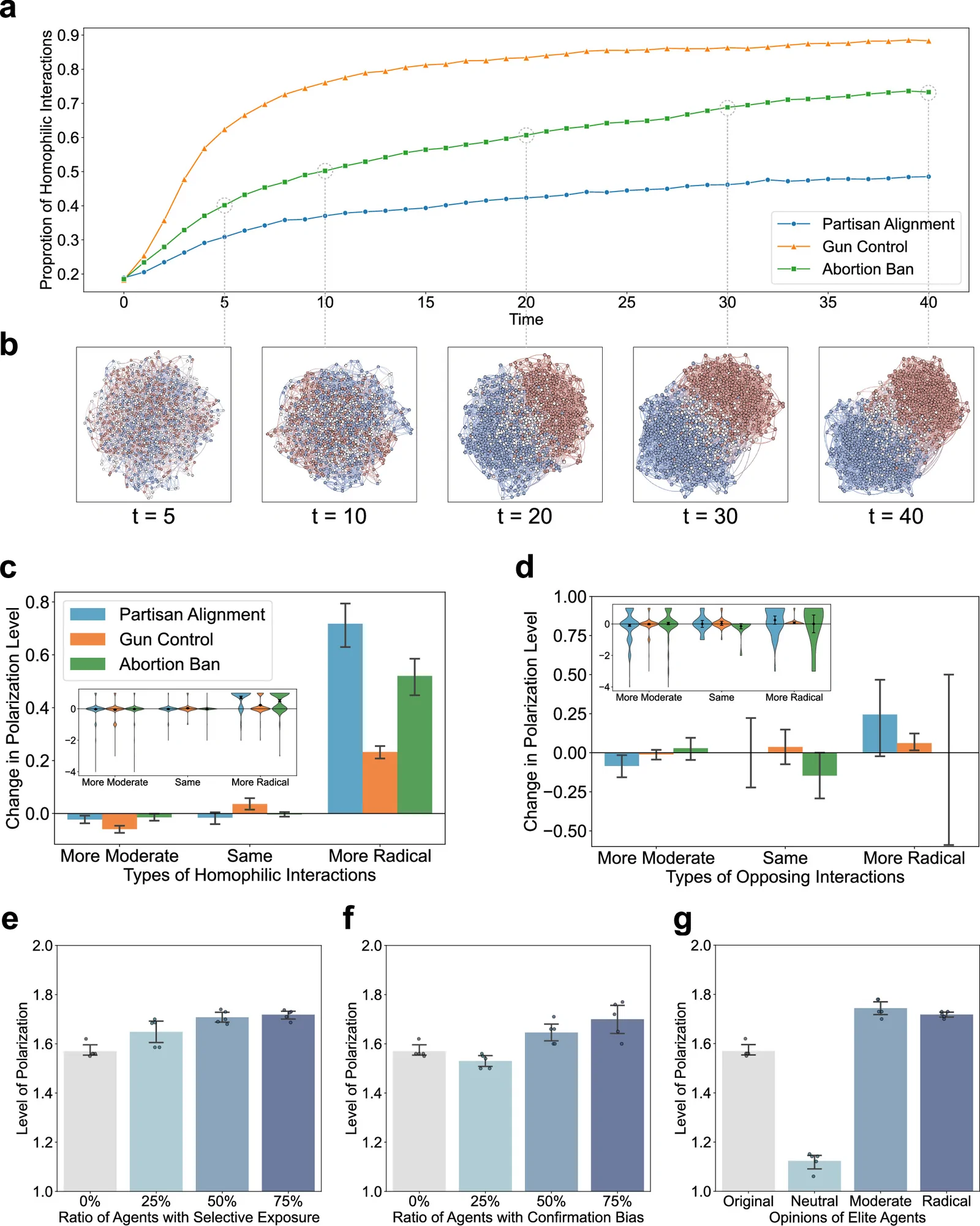
Collective behavioural patterns behind the emergence of polarization among LLM agents. **a** Changes in the proportion of homophilic interactions over time. Agents are increasingly likely to interact with those holding similar opinions. The number of agents is 1000. **b** Evolution of social networks among LLM agents, where agents with similar opinions are more likely to interact with one another, exhibiting the tendency toward homophilic clustering. Each network visualization corresponds to the circled points in **a**. **c** Behavioural patterns consistent with the echo chamber effect, where exposure to more radical homophilic interactions is associated with higher polarization levels. **d** Behavioural patterns consistent with the backfire effect, where interactions with agents holding opposing opinions are associated with increased polarization. In **c, d**, bars represent the average, and error bars represent the corresponding 95% confidence intervals (CIs). Sample sizes, corresponding to the number of agent interactions, for bars in **c** are 3680, 4212, 3962, 494, 761, 1315, 170, 1466, and 246. Sample sizes in **d** are 630, 706, 523, 27, 27, 41, 45, 65, and 22. **e**–**g** Effects of individual-level behavioural tendencies related to selective exposure, confirmation bias, and elite signaling. In **e**–**g**, bars show average levels of polarization in the last five timesteps (*n* = 5, corresponding to five timestep-level polarization values computed from all agents' opinions), and error bars show the corresponding 95% CIs. When the system consists of more agents with traits of **e** selective exposure or **f** confirmation bias, and **g** influencers adopt non-neutral opinions, the level of polarization increases.

The above results uncover that free-form social interactions among LLM agents result in the emergence of opinion polarization, raising a natural question regarding the exact role these interactions play in this process. Therefore, we begin by assessing the extent to which agents interact with those holding similar opinions. As shown in Fig. 3a, we find that the proportion of interactions between agents in the same camp, i.e., left- or right-leaning camp, increases by 156.8−382.7% over time. This increase eventually leads to 48.5−88.3% of interactions in these systems occurring between agents who share similar opinions, suggesting a tendency for similar agents to cluster together. This process is consistent with the notion of homophily, where “birds of a feather flock together”^58,86^, and has been observed in naturalistic LLM social interactions^28^. Moreover, our agents increasingly avoid interacting with agents who hold opposing opinions (Supplementary Fig. S8a). Indeed, their tendencies of homophilic clustering and opposing avoidance appear to be driven by their generated evaluations of others. Further experiments show that agents are more likely to form more favorable impressions of and use more positive language to describe those within their own camp than those in opposing camps (Supplementary Note 8). It is worth mentioning that no mechanisms prompting in-group bias or homophilic clustering were inserted into the system; this emerges purely from the LLM agents’ free-form choice behaviours in social relationships. More interestingly, in the system discussing the abortion ban, agents gradually self-organize into two communities (Figure 3b), with one supporting left-leaning opinions and the other supporting right-leaning ones. Indeed, beyond LLM agents, homophilic clustering is also widely observed in human society^51,55,61^ and in- vs. out-group bias has been posited by evolutionary psychologists with the emergence of justificatory reason^87^.

A widespread subsequent concern is the potential for the clustering of homophilic peers to reinforce opinion polarization, which is often termed the echo chamber effect in studies of human behaviours^51,55^. Therefore, we examine the relationship between homophilic clustering and polarization levels in the system of LLM agents. In particular, we calculate the average opinions of each agent’s neighbors to quantify the relative levels of radicalization between the agent and its neighbors (see details in Method M5). Focusing on the interactions between homophilic agents (Figure 3c), we observe that exposure to more radical agents significantly increases the level of polarization (two-sided Student’s *t*-tests, partisan alignment *t*(169) = 16.80, *p* ≪ . 001, Cohen’s *d* = 1.288, 95% CI  = [0.633, 0.802], gun control *t*(1465) = 19.37, *p* ≪ . 001, Cohen’s *d* = 0.506, 95% CI  = [0.209, 0.256], and abortion ban *t*(245) = 13.99, *p* ≪ . 001, Cohen’s *d* = 0.892, 95% CI  = [0.447, 0.594]). This indicates that LLM agents exhibit behavioural patterns consistent with the echo chamber effect. On the other hand, despite homophilic interactions representing the majority (54.8%  − 82.2%), agents still retain a small chance of encountering those with opposing opinions (7.4%  − 10.2%). Nevertheless, these interactions with opposing agents do not consistently reduce the polarization level as expected (Fig. 3d). Instead, exposure to agents with opposing opinions could potentially increase polarization levels, indicating behavioural patterns consistent with the backfire effect^53^. We note that the echo chamber and backfire effects do not universally occur across all individuals in the real world, with some recent theoretical debates^55,88,89^; instead, they exhibit substantial heterogeneity. Extensive experiments reveal that the system autonomously formed by the LLM agents can capture this heterogeneity, showing consistency with empirical observations (Supplementary Note 4).

Overall, these observations suggest that in the discussion of critical political issues, LLM agents can spontaneously organize their social network. Their networks not only exhibit the property of homophilic clustering, but also shape collective opinions in ways consistent with the echo chamber and backfire effects. To support these findings, we report further quantitative metrics, including modularity, assortativity, and the homophily index, along with extended analyses on the network properties of their self-organization (see Supplementary Note 4). Notably, we observe that social networks evolve into a scale-free degree distribution, where high-degree agents gain great popularity in free-form interaction (Supplementary Fig. S13), following degree distribution models of social networks^90^. Moreover, when we replace LLM agents’ self-organized network with a random or static network, we find that one-camp opinions dominate the overall system (Supplementary Fig. S14), which highlights the important role of self-organized social networks in shaping polarization patterns exhibited by LLM agents. Importantly, these results align with established argument-accumulation models in opinion dynamics, where polarization emerges from the cumulative exchange and reinforcement of persuasive arguments under homophilic interactions^58,62^, as reflected in our analysis of agents’ self-reported reasons (Supplementary Note 8).

As discussed above, the polarization of LLM agents is driven by two coupled processes of network organization and opinion formation. Specifically, in the network organization process, agents tend to interact with similar peers (Fig. 3a, b), while in the opinion formation process, agents tend to comprehend messages in a way that aligns with their pre-existing opinions (Figures 3c, d). These two tendencies of LLM agents have been frequently observed in humans^51,52,56,63,64^, and are often referred to as selective exposure^63^ and confirmation bias^64,91^, respectively. Unlike network-level patterns, these two mechanisms adopt a psychological perspective, focusing more on how individuals’ traits and behaviours contribute to polarization. We note that these two mechanisms are not explicitly hard-coded or assumed in the proposed system. Rather, agents’ behavioural patterns consistent with these mechanisms naturally emerge from agents’ social interactions. For example, analyzing the self-reported reasons generated during opinion formation reveals patterns in line with confirmation bias (Supplementary Note 8).

Given these observed network-level patterns, one may wonder whether LLM agents exhibit behaviours consistent with psychological mechanisms discussed in studies of human opinion formation. To answer this question, we design a comparative experiment: we vary the proportion of agents exhibiting behaviours consistent with selective exposure or confirmation bias, and then examine the resulting changes in the level of polarization. Cases where 0% or 100% of agents are configured to exhibit these behaviours are deliberately designed as extreme scenarios for comparative purposes. As shown in Fig. 3e, f, we observe that the increase in agents exhibiting either behaviour leads to a marked increase in the level of polarization characterizing the overall system. This suggests that behaviours related to selective exposure and confirmation bias contribute to the polarization of LLM agents, consistent with findings reported in studies of human behaviour^56,64,92^.

On the other hand, prior studies have pointed out that opinion formation is also largely influenced by the traits or behaviours of others, particularly top influencers on social media^54,56,92–94^. As suggested by the elite signaling mechanism^54,92,93^, the polarization level of influencers’ opinions is positively associated with greater polarization in the overall population. To validate this effect on LLM agents, we introduce additional top influencers holding neutral, moderate left/right, and strong left/right opinions into the system, with these influencers sending non-personalized messages to all other agents (Figure 3g). In this setting, the top influencers are symmetrically distributed across the right- and left-leaning camps. We observe an effect in the LLM-agent system that is qualitatively consistent with patterns reported in studies of human opinion formation. When the influencers hold non-neutral opinions, the final polarization level is significantly increased (two-sided Student’s *t*-tests, left/right versus original, *t*(8) = 10.63, *p* ≪ . 001, Cohen’s *d* = 6.720, 95% CI  = [0.116, 0.180]; moderate versus original, *t*(8) = 8.64, *p* ≪ . 001, Cohen’s *d* = 5.462, 95% CI  = [0.128, 0.220]). By contrast, the introduction of neutral influencers significantly reduces the polarization level of the system by 28.5% (two-sided Student’s *t*-tests, original versus neutral, *t*(8) = 21.56, *p* ≪ . 001, Cohen’s *d* = 13.636, 95% CI  = [0.399, 0.495]). Besides the three studied mechanisms, we also examine a series of other individual-level mechanisms, finding their effects on LLM agents to be consistent with those observed in humans (Supplementary Note 5). Overall, these experiments consistently suggest that LLM agents exhibit individual-level behavioural responses that parallel those documented in studies of human opinion formation.

### Synthetic testbed for exploring hypothetical intervention strategies

**Fig. 4:**
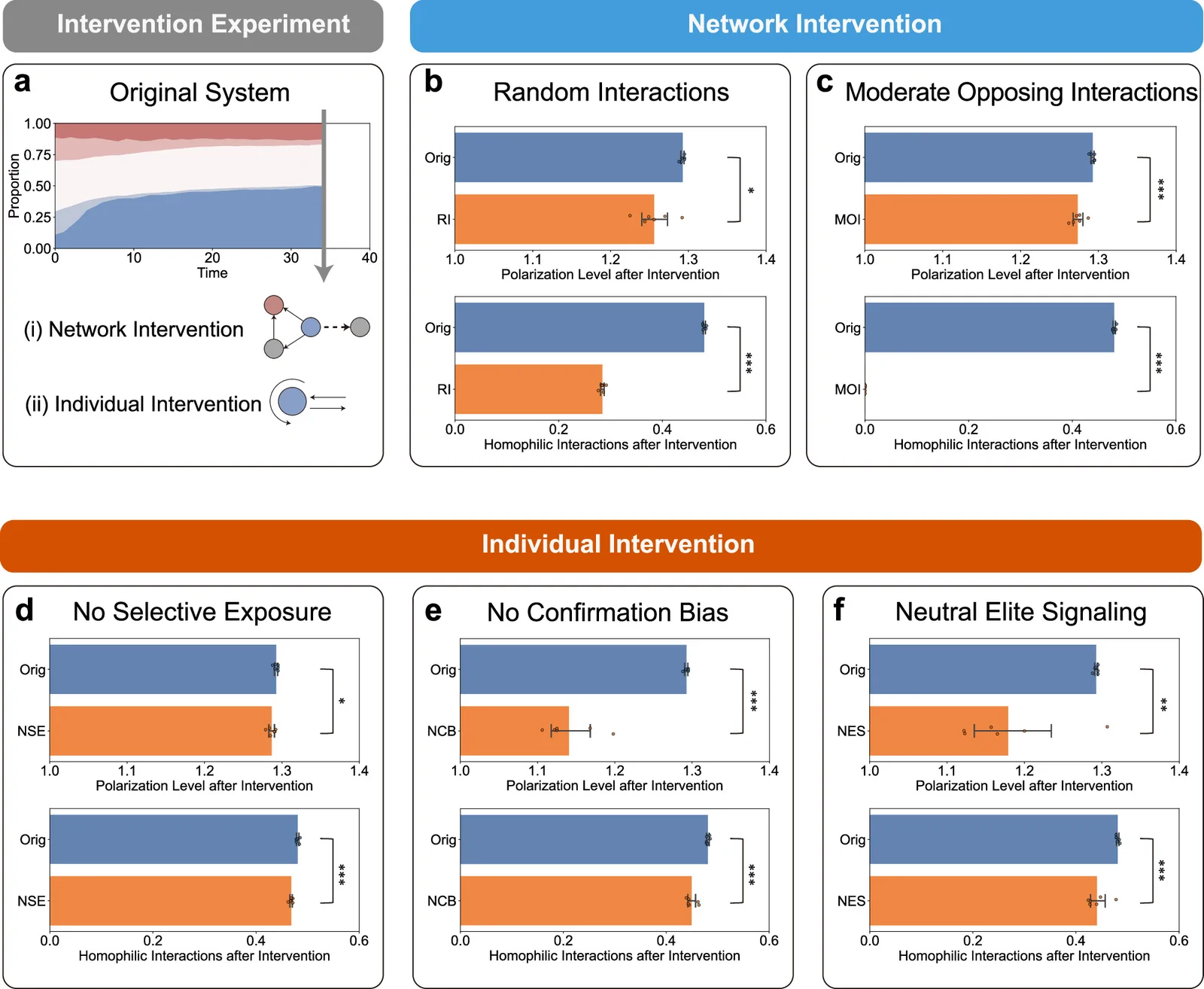
Synthetic testbed for exploring hypothetical intervention strategies. **a** Intervention experiments, where two types of strategies are applied to the original polarized system: (i) network interventions, which directly modify LLM agents' social network, and (ii) individual interventions, which adjust agents' traits and behaviours. The number of agents in each experiment is 1000. **b**, **c** Network intervention strategies of **b** random interaction, where agents randomly interact, and **c** moderate opposing interaction, where agents receive messages only from those with opposing moderate opinions. **d–f** Individual intervention strategies of **d** no selective exposure, where agents tend to interact with those holding diverse opinions, **e** no confirmation bias, where agents are open-minded to diverse opinions, and **f** neutral elite signaling, where agents receive non-personalized neutral messages. In **b**–**f**, the upper sub-figures show the comparison of polarization levels between the original and the intervened systems, while the lower sub-figures illustrate the comparison of proportions of homophilic interactions. Bars show the average values after intervention (*n* = 6, corresponding to six timestep-level values computed from all agents' opinions or interactions), and error bars show the corresponding 95% CIs. We perform two-sided Student/Welch’s *t*-tests: **b** *t*(5.16) = 3.87, *p* = . 011 < . 05, Cohen’s *d* = 2.237, 95% CI  = [0.013, 0.061], *t*(10) = 80.96, *p* < . 001, Cohen’s *d* = 46.741, 95% CI  = [0.191, 0.202], **c** *t*(10) = 5.24, *p* < . 001, Cohen’s *d* = 3.027, 95% CI  = [0.011, 0.027], *t*(5) = 366.00, *p* < . 001, Cohen’s *d* = 149.418, 95% CI  = [0.477, 0.484] (one-sample *t*-test against zero), **d** *t*(10) = 2.46, *p* = . 033 < . 05, Cohen’s *d* = 1.422, 95% CI  = [0.001, 0.011], *t*(10) = 6.64, *p* < . 001, Cohen’s *d* = 3.832, 95% CI  = [0.008, 0.017], **e** *t*(10) = 10.65, *p* < . 001, Cohen’s *d* = 6.146, 95% CI  = [0.120, 0.184], *t*(10) = 6.99, *p* < . 001, Cohen’s *d* = 4.033, 95% CI  = [0.022, 0.042], and **f** *t*(10) = 4.02, *p* = . 002 < . 01, Cohen’s *d* = 2.324, 95% CI  = [0.051, 0.177], *t*(10) = 4.78, *p* < . 001, Cohen’s *d* = 2.760, 95% CI  = [0.021, 0.059]. In this synthetic testbed, individual-level strategies are associated with larger observed reductions in polarization than network-level interventions.

The above results suggest that the behaviours and collective patterns produced by the LLM agents are consistent with empirical observations and established opinion-dynamics models. This consistency suggests the potential of the proposed LLM-agent system as a valuable synthetic testbed for exploring hypothetical intervention strategies in networked LLM-agent systems. To explore this potential, we design five intervention strategies in our synthetic testbed (Fig. 4). At the network level, we consider two strategies: (i) Random interactions (RI): we remove the homophilic clustering property of LLM agents’ social network by allowing agents to randomly interact with each other. (ii) Moderate opposing interactions (MOI): given the echo chamber and backfire effects, agents are only allowed to receive messages from those adopting moderate opposing opinions. At the individual level, three strategies are proposed: (iii) No selective exposure (NSE): agents are prompted to have the tendency to communicate with those holding diverse opinions. (iv) No confirmation bias (NCB): agents are instructed to be open-minded to persuasion of diverse opinions. (v) Neutral elite signaling (NES): agents receive non-personalized neutral messages from newly introduced top influencers. Indeed, these interventions are motivated by established lines of prior work and are used here as experimental interventions in our synthetic system: MOI draws on studies of exposure to cross-cutting information^89,95^; RI operationalizes randomized interaction; NSE and NCB relate to promoting open-mindedness^96^; and NES is inspired by the design of Bail et al.^97^.

Based on the proposed strategies, we intervene in a system that has converged to a polarized state, i.e., the system at *t* = 35 (Figure 4a). By comparing the evolution of the original system with that of the intervened system following intervention (Figure 4b-f), we find that all strategies significantly reduce the level of polarization. In particular, the intervention of MOI has a clear and significant effect in reducing opinion polarization among agents (two-sided Student’s t-test, *t*(10) = 5.24, *p* < . 001, Cohen’s *d* = 3.027, 95% CI  = [0.011, 0.027]). The effects are relatively small, however, producing approximately a 2% change in agent opinions. This modest effect is consistent with findings from human studies, where cross-cutting interactions reduce polarization but typically result in only small shifts among actual opinions^89,95^. Compared with network-level interventions that directly modify agents’ social networks, individual-level strategies of NCB and NES contribute to the greatest reduction in polarization by 11.8% and 8.8%, respectively. On the other hand, we observe that the NSE strategy has limited effects. This is because, in an almost stable social network, agents’ non-selective tendencies do not provide exposure to diverse opinions but instead quickly crystallize the existing network structures that foster polarization (see detailed analyses on intervention strategies in Supplementary Note 7). Despite the difficulty of recommending openness to human populations, these results suggest that in our synthetic testbed, access and open-mindedness to diverse opinions at the individual level appear more effective than altering social networks in polarized systems. Furthermore, we observe that all of the strategies, despite reducing homophilic interactions by varying rates from 2.6% to 100%, significantly promote inclusive conversations among agents with diverse opinions (lower sub-figure in Fig. 4b–f). In all, these experiments compare several hypothetical strategies and illustrate the value of the proposed system for controlled exploration of intervention hypotheses within networked LLM-agent systems.

## Discussion

This work uncovers that LLM agents exhibit polarization patterns on political issues, at both the individual and collective levels. Specifically, from the individual perspective, opinion formation among LLM agents exhibits behavioural patterns consistent with social and psychological mechanisms^63,64^, e.g., selective exposure, confirmation bias, elite signaling. From the collective perspective, LLM agents organically develop social networks with properties characteristic of human social networks, e.g., homophilic clustering and scale-free degree distributions^28^. Moreover, their self-developed social networks, in turn, shape LLM collective opinions in ways consistent with the echo chamber and backfire effects described in studies of human behaviour, resulting in the emergence of opinion polarization^51–56,94,98,99^. Notably, all these behavioural patterns, network organization, and collective opinions naturally emerge from interactions among agents completely driven by LLMs. Building on these findings, our work shifts the perspective from isolated LLMs^29,30,32,35–38^ to complex, networked LLM agent societies, offering a synthetic testbed for studying emergent collective dynamics in artificial agents. While our main results are based on OpenAI’s ChatGPT and three political issues, we also perform extensive experiments with different LLMs (both full-run and midway-defection settings, Supplementary Note 6) as well as on topics of immigration restriction and the flat Earth theory (Supplementary Note 13), finding that these experiments consistently support our core findings. We also vary two key initial conditions, i.e., the social network structure and initial opinion distribution, and find that both resulting polarization patterns and final network structures remain strikingly similar, highlighting the robustness and generalizability of our system and experiments (Supplementary Note 12).

While this work does not make theoretical contributions, it is grounded in established theories and models of opinion dynamics^58,62,100,101^. In particular, this work follows the argument-accumulation lineage, which explains polarization as arising from the cumulative exchange and reinforcement of persuasive arguments^58,60,100,101^ rather than from direct opinion averaging or social pressure^102,103^. We further analyze agents’ self-reported reasons during opinion formation, observing that agents who become more polarized tend to accumulate substantially more supporting arguments over time (Supplementary Note 8). This observation highlights a clear connection to argument-accumulation models and suggests that the polarization dynamics observed in our synthetic LLM-agent testbed likely stem from similar underlying mechanisms. At the same time, we acknowledge broader theoretical debates on social media and polarization. Some recent studies have emphasized that echo chamber effects do not universally apply across all individuals in real-world settings, with substantial heterogeneity in exposure to like-minded content^55,88^. In our experiments, we also observe similar patterns in LLM agents (Supplementary Note 4). Moreover, deliberative and argument-accumulation models have limitations, such as their abstraction from affective responses and identity-based dynamics^104–106^, which have been highlighted in more recent conceptions of polarization. While our primary analyses focus on opinion-based polarization, we provide supplementary analyses of behavioural patterns consistent with some of these broader dimensions (Supplementary Note 8).

Previous studies have documented the presence of political biases in LLMs^27,30,76,77,107,108^. In particular, there are two main lines of research. The first line focuses on analyzing political bias in “plain vanilla” LLMs, to examine the extent of biases present in their default configurations^27,77,107,108^. The second line of research assigns demographics to LLMs and evaluates how well these models represent different populations^30,44,76^. While this approach allows LLMs to adopt political opinions resembling those of real-world individuals with similar demographics^30,76^, they also find that LLMs fall short in accurately representing certain minority groups^76,109^. Overall, some recent studies attempt to move beyond default configurations and explore the potential of demographically prompted LLMs as proxies for human populations. Nevertheless, they have yet to explore whether and how political biases may emerge through LLM agents’ social behaviours – including friend selection, opinion updating, and patterns of communication. Our system addresses this gap and can be readily extended to model more diverse human subgroups by leveraging prompts as “soft” parameters (Supplementary Note 10). This naturally leads to the next question: will LLM agents faithfully follow their assigned political personas and exhibit behaviours consistent with humans holding comparable identities? In this work, we discover that the underlying political bias of LLMs does lead these agents to occasionally exhibit behaviours that deviate from their assigned political personas, which we refer to as self-inconsistency. This inconsistency causes agents to gradually drift toward left-leaning positions during interaction (Fig. 2b), and over time, cumulatively results in the collective left-skewed polarization observed in Fig. 1b–d. To address self-inconsistency, we propose the self-regulation strategy inspired by Bandura’s social learning theory^84,85^, enabling agents to self-monitor and adjust their behaviours. We find self-regulated LLM agents develop their collective opinions into balanced right-left polarization (Fig. 2d), better reflecting real-world distributions. Furthermore, comparison of polarization speeds between the original and self-regulated agents reveals only slight differences, suggesting that model bias has a minimal impact on accelerating polarization (Supplementary Note 3). Overall, these findings underscore the importance of studying bias and consistency at the level of interacting LLM agents, and of investigating collective opinion dynamics in synthetic social systems.

While this work advances prior GABM studies by providing a solid synthetic testbed in which large-scale LLM agents, under minimal social assumptions, produce emergent polarization patterns, it also shares limitations common to current GABM research^110–112^. In particular, like prior studies, the proposed system faces natural challenges to external validity. Although results are consistent with well-established opinion-dynamics models^51,53,58^, the extent to which these patterns quantitatively correspond to real-world human behaviour must be further validated. In addition, compared to prior models with transparent mechanisms, GABM comes at the cost of reduced interpretability, as agent behaviour is shaped through prompt-based design and simplified opinion representations. Moreover, the intervention results presented here should be understood as hypothesis-generating. The primary value of the proposed system lies in enabling controlled, pre-experimental exploration of how interaction rules, network structures, and agent-level behaviours may jointly shape collective opinion dynamics. Future research will benefit from complementary human experiments^58,113^, as well as more systematic calibration and evaluation against fine-grained real-world data.

## Methods

### M1 Design of the networked system of LLM agents

To understand the opinion dynamics of LLM agents, we develop a networked system of LLM agents. In this system, each agent is empowered with three stages that ensure their basic social capabilities: (1) self-expression, which enables agents to consider and express their reasons for supporting their opinions, (2) communication, which enables agents to exchange opinions and the corresponding reasons, and (3) opinion updates, which enable agents to update their opinions based on received messages. These three stages take advantage of LLMs’ capabilities in traditional natural language processing tasks^1,2^. For example, the self-expression stage predominantly depends on automated text generation, and the opinion update stage relies on text summarization. These capabilities allow agents to perform basic social interactions. Below we introduce our proposed system in detail.

In each system, agents communicate about one political issue, e.g., gun control or abortion rights. We randomly initialize the system, including agents’ opinions on the political issue and their social relationships. When initializing the system, we consider two aspects. First, the initialization should prevent the system from starting from an extreme case, e.g., when agents’ opinions are already highly polarized. Second, the initialization setting should allow enough flexibility for agents to develop their collective opinions and form social networks. As starting from a clustered network and highly polarized opinion distribution would result in a crystallized setting, not allowing us to study how interaction dynamics emerge over time, we introduce some randomness in the initial networks and opinions considered. Specifically, we sample each agent’s initial opinion following a near-Gaussian distribution as shown in Fig. 1c. We initialize the social network using a Watts-Strogatz model with the rewiring probability of 0.001, which exhibits a relatively weak small-world property, characterized by a normalized clustering coefficient of 0.99 and a normalized average path length of 0.71.

After initialization, the self-expression stage requires each agent *i* to generate a message expressing the reasons for supporting their opinions. These messages provide detailed information about agents’ beliefs regarding the political issue. Then, following the communication stage, each agent *i* first chooses whether to communicate with a socially connected or random agent. If agent *i* declines further communication with its current partner, it will be randomly assigned a new one with which to interact. Here we denote the interaction partner as agent *j*. It is worth noting that distinct from prior studies that allow agent *i* to “scan” all other agents and select its preferred ones^39,41,42^, we adopt a more naturalistic setting in which agents may occasionally encounter individuals they dislike, but autonomously decide whether to maintain social connections with those favored. Based on the opinions and reasons of agents *i* and *j*, agent *i* generates messages to persuade agent *j* of its opinion. We note that after one round of communication, agents *i* and *j* become directionally linked, allowing agent *i* to contact agent *j* in the subsequent timestep. Based on the messages that agent *i* has received from its socially connected neighbors, agent *i* updates its opinions and then generates reasons for its new opinion. The system operates these three stages iteratively and enables agents to freely establish social relationships, communicate, and form opinions on critical political issues.

It is worth noting that we do not rely on an external classifier to evaluate agents’ opinions. Instead, we prompt the agents to self-report their opinions on political issues. For example, when discussing partisanship, agents are asked: “What do you feel about political partisanship?” The agent then responds by placing itself into one of five opinion categories: “strongly support the Republican Party” (right), “support the Republican Party” (moderate right), “do not have a tendency” (neutral), “support the Democratic Party” (moderate left), or “strongly support the Democratic Party” (left). This approach leverages the role-playing capability of LLM agents, allowing them to express self-evaluation in line with how human respondents articulate their own opinions. When it comes to gun control, right-leaning opinions indicate support for weaker gun control, while left-leaning opinions indicate support for stricter control. Similarly, for abortion bans, right-leaning opinions represent support for abortion bans, whereas left-leaning opinions represent opposition to such bans.

To ensure the system scale is large enough to support observations on network structures, we incorporate 1000 agents in all simulations of networked systems, except for the individual-level mechanism experiments, where 100 agents are used due to our focus on individuals and high computational costs. We use ChatGPT (GPT-3.5) through the public OpenAI API to run all the experiments in the Main Text. We set the temperature to 1 in all experiments and adopt a zero-shot setting. We also perform experiments using other LLMs, including GPT-4o, ChatGLM, Llama-3, and DeepSeek-V3 (Supplementary Note 6), as well as experiments with varying temperatures (Supplementary Note 9). These experiments consistently validate the conclusion that long-term social interactions among LLM agents lead to the emergence of opinion polarization. Additionally, by varying two key initial conditions, i.e., the social network structure and the initial opinion distribution, we find that both the resulting polarization patterns and networks remain consistent (Supplementary Note 12). Detailed prompts to operate the overall system are provided in Supplementary Methods.

### M2 Pairwise interaction-based bias evaluation experiment

To investigate the origin of the pattern of biased opinion, we design a pairwise interaction-based bias evaluation experiment (Figure 2a). Different from the complex networked system, we focus on the effect of the one-round interaction between agents with identical opinions in this experiment. In particular, we begin by constructing a collection of agents, who are initially assigned the same opinion and prompted to generate their own supporting reasons for it. These agents are paired randomly and one of them, denoted agent *i*, is required to persuade the other agent, denoted agent *j*, of the opinion. After this single communication round, we measure the change in agent *j*’s opinion (Figure 2b and Supplementary Figs. S3 and S4). Details for the experiment are provided in Supplementary Methods.

### M3 Self-regulation strategy for the problem of self-inconsistency

To mitigate observed self-inconsistency inherent in LLM agents, we propose a self-regulation strategy inspired by social theory with a natural human analogue^84,85^. As discussed above, unlike vanilla LLMs, LLM agents generate extended self-explanations and interaction histories, which motivates drawing inspiration from established social and behavioural theories when addressing their self-inconsistency. In this way, we follow Bandura’s concept of self-regulation in social learning theory^84,85^, employing LLM agents to self-regulate their expressions, communication, and opinions, and update their corresponding behaviours. In particular, after these agents generate a message supporting their opinions, we require them to check whether the message reflects their opinions. If not, they will continue to re-generate new messages until their consistency requirements are satisfied. This is conceptually analogous to an individual holding a self-consistent ‘line’ in conversation with others^114^. Similarly, in the stages of communication and opinion update, we instructed LLM agents to ensure that their generated communication messages align with their current opinions and that their updated opinions are plausible given their received messages. Detailed prompts for the self-regulation strategy are provided in Supplementary Methods.

### M4 Evaluation of hypothetical interventions in the synthetic LLM-agent system

To explore the question of which intervention strategies are more effective in reducing polarization in the synthetic system, we design an intervention experiment based on the proposed networked system of LLM agents. Considering commonly studied polarization scenarios, we focus on interventions in an already polarized system. We use the system discussing partisan alignment at *t* = 35, which has converged to a polarized state, as an example. As described in the Main Text, we propose five intervention strategies. At the network level, we directly modify the social network of LLM agents, while at the individual level, we adjust their traits and behaviours through prompts. We supplement the detailed prompts in Supplementary Methods. Using these strategies, we intervene in the polarized system at *t* = 35. The intervention, i.e., the modification of social networks or the adjustment of agents, continues from *t* = 35 to *t* = 40. We take the original system as the control group and the intervened systems as the treatment group. We assess the effectiveness of different strategies in reducing polarization by comparing the polarization levels of the original system and the system after intervention. We evaluate their performances in promoting less divisive conversations through the resulting changes in the proportion of homophilic interactions.

### M5 Metrics

**Level of polarization**. To quantitatively measure polarization and its contributing factors, we define polarization and radicalization metrics following prior studies^51,55,72^. We measure the level of polarization by calculating the average distance between agents’ opinions and the neutral position. In particular, we assume that the opinion distribution is characterized by the vector [*f*_−2_, *f*_−1_, *f*_0_, *f*_1_, *f*_2_], where *f*_*k*_ denotes the relative frequency of agents holding opinion *k* and $\mathop{\sum }_{k=-2}^{2}{f}_{k}=1$. We compute the polarization level as ${s}_{{{{\rm{pol}}}}}=\mathop{\sum }_{k=-2}^{2}| k| \times {f}_{k}$, where the absolute value ∣*k*∣ measures the deviation distance of opinion *k* from neutral, i.e., opinion 0. The polarization level *s*_pol_ falls into the domain of [0, 2], and a larger value indicates a higher level of polarization. We further measure the change in polarization for each agent. Specifically, we calculate the change in agent *i*’s opinion between the timesteps *t* and *t* − 1 along the direction of their opinion at the timestep *t* − 1, expressed as *s*_cp, *i*_ = (*x*_*i*, *t*_ − *x*_*i*, *t*−1_) × sign(*x*_*i*, *t*−1_). Here *s*_cp, *i*_ > 0 indicates that the agent *i* has adopted a more polarized opinion after the interaction at the timestep *t* − 1. It is worth noting that after 99.71% of homophilic interactions, agents adopt same-camp or neutral opinions, i.e., *x*_*i*, *t*_ × sign(*x*_*i*, *t*−1_)≥0.

**Self-inconsistency rate**. We evaluate the self-inconsistency problem by computing the distance between the experimental opinion transition probability matrix and the ideal identity matrix. In particular, we denote the experimental transition matrix as **P**, where the element ${P}_{k,k{\prime} }$ represents the probability that an agent originally holding opinion *k* transforms into one holding opinion $k{\prime}$. Therefore, the sum of elements in each row of **P** is equal to 1. Ideally, we assume that one round of communication between two agents sharing the same opinion should not trigger any opinion transition, leading to an identity matrix. We formulate the self-inconsistency rate as ${s}_{si}=\left(\mathop{\sum }_{k=-2}^{2}\mathop{\sum }_{k{\prime}=-2}^{2}{P}_{k,k{\prime} }*| k-k{\prime} | \right)/\mathop{\sum }_{k=-2}^{2}{\mathbb{1}}$.

### Reporting summary

Further information on research design is available in the Nature Portfolio Reporting Summary linked to this article.

## Supplementary information

Supplementary Information

Reporting Summary

Transparent Peer Review file

## Source data

Source Data

## Data Availability

The data used in this study are publicly available at https://github.com/tsinghua-fib-lab/LLM_for_Polarization^115^. The repository includes two categories of data: (i) timestep-level records from the LLM-agent simulations, covering agent-to-agent interactions and agent opinion updates; and (ii) processed statistical datasets underlying Figs. 1–4 and the analyses reported in the Supplementary Information. Source data are provided with this paper.

## Code availability

The code used in this research is available at https://github.com/tsinghua-fib-lab/LLM_for_Polarization^115^.
