# Supplementary material for: Emergence of polarization in networks of large language model agents
Source: Nat Commun. 2026 Oct 8;17:11463. doi: 10.1038/s41467-026-78228-y (PMC13740318; doi:10.1038/s41467-026-78228-y)
Supplement: Supplementary file 1 [file 41467_2026_78228_MOESM1_ESM.pdf]

Supplementary information for
Emergence of polarization in networks of large language model agents

Jinghua Piao[1+], Zhihong Lu[1+], Chen Gao[1], Fengli Xu[1], Qinghua Hu[2], Fernando P. Santos[3*], Yong Li[1*], and James Evans[4,5*]

[1]Department of Electronic Engineering, Tsinghua University, Beijing National Research Center for Information Science and Technology (BNRist), Beijing, P. R. China
[2]College of Intelligence and Computing, Tianjin University, Tianjin, P. R. China
[3]Informatics Institute, University of Amsterdam, Amsterdam, the Netherlands
[4]Knowledge Lab, University of Chicago, Chicago, U.S.A
[5]Santa Fe Institute, Santa Fe, U.S.A.
[*]To whom correspondence should be addressed; Email: f.p.santos@uva.nl, liyong07@tsinghua.edu.cn, jevans@uchicago.edu.
[+]Jinghua Piao and Zhihong Lu contribute equally to this work.

Contents

1 Supplementary Methods: A Networked System of Large Language Model Agents **3**

1.1 System Description . . . 3

1.2 Pairwise Interaction-based Bias Evaluation Experiment . . . 4

1.3 Self-regulation Strategy . . . 4

1.4 Intervention Experiments . . . 5

2 Supplementary Note 1: Political Polarization in Human Society **6**

3 Supplementary Note 2: Large-scale Simulation of LLM Agents **7**

4 Supplementary Note 3: Self-inconsistency Problem in LLM Agents **7**

5 Supplementary Note 4: Observations on LLM agents' Self-organized Social Network **15**

6 Supplementary Note 5: Results of Other Individual-level Behavioural Patterns **19**

7 Supplementary Note 6: Experiments on Different LLMs **19**

8 Supplementary Note 7: Analyses on Different Intervention Strategies **21**

9 Supplementary Note 8: Analyses on Agents' Social Perceptions and Argument Accumulation Process **25**

10 Supplementary Note 9: Experiments on LLMs with Varying Temperatures **44**

11 Supplementary Note 10: Experiments on LLMs with Enhanced Prompting **47**

12 Supplementary Note 11: Experiments on the Effect of Increasing Severing Costs **48**

13 Supplementary Note 12: Results of Simulation under Different Initial Conditions **48**

14 Supplementary Note 13: Results of Simulation on Different Issues **49**

15 Supplementary Note 14: Supplementary Notes on Statistical Testing and Robustness Checks **49**

1 Supplementary Methods: A Networked System of Large Language Model Agents

1.1 System Description

We simulate a networked system of large language model (LLM) agents, where these agents can freely establish social relationships, communicate, and form their opinions on political issues. To empower agents with these basic social capabilities, we design three core stages, i.e., (i) self-expression, (ii) communication, and (iii) opinion update. Here the self-expression stage, serving as the role of memory, requires each agent to generate and then preserve a message describing their current opinion. The communication stage enables agents to choose with whom and what to communicate freely. The opinion stage empowers these agents with the capability to comprehend messages from their socially connected peers and then form their current opinions. The following section introduces the detailed implementation of the overall system.

Initialization. In a system, we focus the communication among agents on a single political issue, e.g., partisan alignment. Each agent is initially assigned an opinion on the issue. Following prior practices [36, 7], we adopt a near-Gaussian distribution to initialize agents' opinions. In this way, the system can start from a normal state, where most agents do not take radical opinions and the overall system is also at consensus. For the initialization of their social network, we adopt the well-known Watts–Strogatz model [43], with the rewiring probability of 0.001. The Watts–Strogatz model has been widely adopted to initialize social networks in the simulation of opinion dynamics [36, 22, 35]. Overall, this initialization method allows the system to start from pure randomness, without any pre-assumed evolutionary directions or rules.

Self-expression. After initialization, agents have their own opinions on the discussed political issue. We then require each agent i to generate a message supporting its current opinion as follows,

> Assume you are someone who cares about [issue name]. People are divided into 5 standpoints on [issue name]:
> "[opinion 1]" means you think [description of opinion 1].
> "[opinion 2]" means you think [description of opinion 2].
> "[opinion 3]" means you think [description of opinion 3].
> "[opinion 4]" means you think [description of opinion 4].
> "[opinion 5]" means you think [description of opinion 5].
> Please generate a tweet to persuade yourself to [agent *i*'s opinion] with around 50 words.

It is worth noting that the self-expression stage is performed at every timestep to ensure agents' messages can support their current opinions. Considering the integrity of social interactions among LLM agents, we combine the prompts for opinion update and further self-expression together, which will introduced in the following paragraphs.

Communication. In the communication process, each agent i first decides whether to continue communicating with their socially connected neighbors or contact a random new agent. If it declines further communication, it will be randomly assigned a new one to interact with. Here we refer to the agent who communicates with agent i as agent j. The decision is based on current opinions and the supporting messages of both agent i and agent j. The corresponding prompt is as follows,

> Assume you are someone who cares about [issue name]. You are now discussing [issue name] with a person you know.
> You [agent *i*'s opinion].
> Your thought is: [agent *i*'s supporting message].
> The person [agent *j*'s opinion].
> The thought of that person you are discussing with is: [agent *j*'s supporting message].
> Would you enjoy continue sharing your thoughts with that person?
> Please return 'yes' or 'no', and explain. Please return in JSON with 2 keys: decision and explain.

Next, agent i generates a message to persuade agent j into its opinion. Here agent i considers not only the thoughts, i.e., the supporting messages, of agent i and agent j, but also the history messages from agent j to agent i.

> Assume you are someone who cares about [issue name]. Your thought about [issue name] is: [agent *i*'s supporting message].
> You have received some tweets from your friend: [the historical messages from agent *j* to agent *i*].
> Do you want to interact with or persuade a friend of yours to support your thoughts? The friend has the following thoughts: [agent *j*'s supporting message].
> If yes, please generate a message to persuade your friend to support your perspective with around 50 words.
> Please return in JSON format with 2 keys: 'will' and 'message'. Please keep the message as short as possible. 'will' should be either 'yes' or 'no'. If no, leave 'message' blank.

Opinion Update. After communication, each agent i has received numerous messages from their socially connected peers. Each agent i comprehends these messages and then updates its opinion as follows,

Assume you are someone who cares about [issue name].
Towards [issue name]: You [agent i's opinion]. Your reasons were: [agent $'i$'s supporting message].
You now have received the following tweets from your friends, and you have received some tweets: [messages received from agent i's socially connected peers].
Have you been persuaded to decide your tendency, what would you feel about [issue name]? You need to answer [opinion 1], [opinion 2], [opinion 3], [opinion 4], or [opinion 5], and explain the reasons of it in around 50 words.
Please choose your standpoint on [issue name] based on the INFORMATION PROVIDED ABOVE. You need to answer [opinion 1], [opinion 2], [opinion 3], [opinion 4] or [opinion 5] in the first line, and explain.
"[opinion 1]" means you think [description of opinion 1].
"[opinion 2]" means you think [description of opinion 2].
"[opinion 3]" means you think [description of opinion 3].
"[opinion 4]" means you think [description of opinion 4].
"[opinion 5]" means you think [description of opinion 5].
Please return in JSON, with two keys: tendency and reasons. Please keep the reasons as short as possible.

It is worth noting that we combine the opinion update and the following self-expression stages into the above prompt: each agent i is required to simultaneously update its opinion and reason. Here the updated opinion serves as agent i's new opinion in the next timestep while the reason serves as the corresponding message supporting the new opinion.

In the simulation of the networked system, we first properly initialize the overall system, including agents' opinions and the social network. Next, we iteratively conduct three basic stages, enabling agents to freely establish social relationships, communicate, and form opinions on political issues.

1.2 Pairwise Interaction-based Bias Evaluation Experiment

To explain the observed left-skewed pattern, we design a pairwise interaction-based evaluation experiment. Following most settings in the networked system (e.g., prompts for three basic stages), we simplify two factors in this experiment. First, to avoid network structures interfering with the examination of inherent problems in LLM agents, we make each agent only communicate with one other agent. Second, we ensure the connected agents share the same opinion. This approach allows us to disentangle the effects of inherent issues from social influence on changes in the agents' opinions.

1.3 Self-regulation Strategy

To mitigate the self-inconsistency problem, we design a self-regulation strategy. In particular, we modify the three basic stages by equipping them with a "double check" procedure. This procedure enables agents to verify if their behaviours align with their current status. The modified prompts for these stages are shown as follows.

For the self-expression stage, the self-regulation strategy is added after the original prompt.

You have written the following message to express your opinion on [issue name]: [agent i's supporting messages].
Can you determine that you [agent i's opinion] from the message you wrote?
Please respond 'yes' or 'no' only.

For the communication stage, we incorporate a self-regulation strategy to ensure the persuasiveness of the communication message. The prompts are shown as follows,

You tried to persuade your friend with the following message: [agent i's communication message to agent j]
Do you find the message persuasive enough to persuade your friend to [agent i's opinion]? Please respond 'yes' or 'no' only.

For the opinion update stage, we check whether agent i's updated opinion is plausible and valid, given its prior opinion, supporting message, and received messages.

Assume you are someone who cares about [issue name]. Towards [issue name]:
You [agent i's prior opinion].
Your reasons were: [agent i's supporting message].
You have received the following tweets from your friends, and you have received some tweets: [agent i's received messages from its socially connected peers].
You have been persuaded to change your standpoint from [agent i's prior opinion] to [agent i's updated opinion].
Please reconsider whether your decision is plausible and valid. Please respond 'yes' or 'no' only.
"[opinion 1]" means you think [description of opinion 1].

"[opinion 2]" means you think [description of opinion 2].
"[opinion 3]" means you think [description of opinion 3].
"[opinion 4]" means you think [description of opinion 4].
"[opinion 5]" means you think [description of opinion 5].

It's worth noting that in all three stages, agents are required to re-generate their supporting messages, communication messages, and updated opinions until consistency is reached. Given the efficiency of the overall system, we set a maximal retry number. When the retry limit is reached, the agent will be forced to remain inactive in the current stage.

1.4 Intervention Experiments

We design the intervention experiments for two main purposes. First, we aim to explore what strategies can more effectively reduce polarization and promote less divisive political conversations in the synthetic system. Second, given the systematic similarity between LLM agents and humans, the proposed system serves as a synthetic testbed for pre-experimental exploration of intervention hypotheses, allowing comparative evaluation within a controlled setting. Therefore, based on our findings and prior studies [27, 9, 5, 4, 16, 13, 32, 41, 21], we design five hypothetical strategies, with two at the network level and three at the individual level in the synthetic system. We will introduce the implementation details of these intervention strategies as follows.

Network Level. For the random interaction strategy, an agent i are required to communicate with $N_{i,t}$ random agents at t, where $N_{i,t}$ denotes the number of socially connected friends of the agent i at t. For the moderate opposing strategy, agents can only receive messages from their friends with moderate opposing opinions. Other messages, from friends with homophilic or radical opinions, are blocked out.

Individual Level. For the strategy of no selective exposure, we explicitly add the behavioural tendency to all agents using prompts. Specifically, we remind these agents of this tendency when they choose the partners to communicate with. Detailed prompts are shown as follows,

Assume you are someone who cares about [issue name]. You are now discussing [issue name] with a person you know.
You [agent i's opinion].
Your thought is: [agent $'i$'s supporting message].
The person [agent j's opinion].
The thought of that person you are discussing with is: [agent j's supporting message].
Would you enjoy continuing to share your thoughts with that person?
Please return 'yes' or 'no', and explain. Please return in JSON with 2 keys: decision and explain.
You DO NOT have [trait name], which means [trait description].

For the strategy of no confirmation bias, we also explicitly encode this tendency via prompts. However, different from the strategy of no selective exposure, this focuses on improving agents' open-mindedness to diverse opinions. Therefore, agents are reminded of this behavioural tendency when they comprehend friends' messages and update their own opinions. The prompts are shown as follows,

Towards [issue name]: You [agent i's opinion]. Your reasons were: [agent $'i$'s supporting message].
You now have received the following tweets from your friends, and you have received some tweets: [messages received from agent i's socially connected peers].
Have you been persuaded to decide your tendency, what would you feel about [issue name]? You need to answer [opinion 1], [opinion 2], [opinion 3], [opinion 4], or [opinion 5], and explain the reasons of it in around 50 words.
Please choose your standpoint on [issue name] based on the INFORMATION PROVIDED ABOVE. You need to answer [opinion 1], [opinion 2], [opinion 3], [opinion 4] or [opinion 5] in the first line, and explain.
You DO NOT have [trait name], which means [trait description].
"[opinion 1]" means you think [description of opinion 1].
"[opinion 2]" means you think [description of opinion 2].
"[opinion 3]" means you think [description of opinion 3].
"[opinion 4]" means you think [description of opinion 4].
"[opinion 5]" means you think [description of opinion 5].
Please return in JSON, with two keys: tendency and reasons. Please keep the reasons as short as possible.

For the strategy of neutral elite signaling, we insert an external top influencer who holds a neutral opinion into the agents' social network. This influencer broadcasts non-personalized neutral messages to all other agents at each timestep. In this setup, the influencer is external to the existing social network and remains unaffected by other agents' opinions or interactions, capturing the broadcast nature of real-world top influencers, who differ from friends, colleagues, or family members engaged in

direct interpersonal communication. To avoid overwhelming the agents and maintain balanced communication, each agent can receive a maximum of two neutral messages from the influencer, given that agents are initialized with an average of four friends. This external influencer design avoids directly altering the fundamental behavioural logic or opinions of the observed agent population, ensuring better comparability between neutral elite signaling and other intervention strategies.

2 Supplementary Note 1: Political Polarization in Human Society

For the past decades, polarization has permeated into aspects of our society [27, 9, 5, 4, 16, 13, 32, 41, 21], not only dividing us into liberals and conservatives [27, 4, 5], but also fragmenting us on numerous issues, e.g., abortion, gun control, etc [9, 27, 21]. A growing number of empirical studies quantify polarization on various social media [9, 5, 42, 16, 24, 1]. To compare between human society and the networked system of LLM agents, we have collected empirical opinion distributions in prior studies [9, 5, 42, 16, 24, 1], as summarized in Table S1. Although polarization is widely discussed, data on opinion distribution are rarely available to the public. Therefore, we make great efforts to extract these empirical distributions using various methods, including processing raw open-sourced datasets and estimating them from their figures. Due to limited data availability and estimation resolution, our extracted dataset can only approximate real-world cases. We note the processing procedures in detail as follows.

Table S1. Empirical opinion distributions of political issues. Here the values denote the proportions of left, moderate left, neutral, moderate right, and right opinions. The left-leaning camp consists of left and moderate left opinions while the right-leaning camp consists of right and moderate right opinions. The difference between the two camps is computed as the proportion of the left-leaning camp minus that of the right-leaning camp.

	Left	Mod. Left	Neutral	Mod. Right	Right	Left-leaning Camp	Right-leaning Camp	Difference of Two Camps
Facebook-Politics [5]	0.31	0.10	0.13	0.07	0.39	0.41	0.46	-0.10
Twitter-Politics [16]	0.07	0.62	0.12	0.08	0.11	0.69	0.19	0.49
Reddit-Politics [42]	0.08	0.27	0.43	0.06	0.16	0.35	0.22	0.13
Twitter-Gun Control [9]	0.16	0.40	0.09	0.12	0.22	0.56	0.35	0.21
Twitter-Abortion [9]	0.29	0.15	0.11	0.25	0.20	0.44	0.45	-0.01
ANES-Politics [3]	0.23	0.12	0.34	0.11	0.21	0.35	0.32	0.03
ANES-Ideology [3]	0.18	0.14	0.31	0.14	0.25	0.31	0.38	-0.07
Blogosphere-Politics [24, 1]	0.44	0.04	0.03	0.03	0.45	0.49	0.48	0.00

In the Facebook-Politics dataset [5], we estimate the distribution in Figure 1 of Bakshy et al. [5]. Following the paper [5], a typical conservative of FoxNews.com has an alignment score of $+.80$, whereas a typical liberal of HuffingtonPost.com has an alignment value of -0.65. We take the two values as the criteria to divide the left-leaning and right-leaning camps into four sub-groups, i.e., left, right, moderate left, and moderate right. Here the distribution records ideological alignment of content shared on Facebook. In the Twitter-Politics dataset [16], we process the open-sourced data and obtain the user opinion distribution (Figure 1b in Flamino et al. [16]). Here we exclude users in the category of fake news and extreme bias. In the Reddit-Politics dataset [42], we extract the distribution for political activity on Reddit from Figure 3a of Waller et al. [42]. In the Twitter-Gun Control and Twitter-Abortion datesets [9], we estimate the user opinion distributions from Figures S4 and 1a. In ANES-Politics and ANES-Ideology [3] dataset, we use the 2020 Time Series Study. For the ANES-Politics [3] dataset, we merge the "independent" people and take them as the neutral camp. For the ANES-Ideology [3] dataset, we do not include those who hold extreme opinions or have no thoughts. In the Blogosphere-Politics [24, 1] dataset, we estimate the opinion distribution for domains or blogs from Figure 2c in Liu et al. [24].

From Table S1, we find that except for Twitter-Politics [16], the power between the left-leaning and right-leaning camps is relatively balanced. Moreover, in all datasets, the polarizing camps take up over 50% of the overall. Among them, except for Reddit-Politics [42] and ANES [3], the polarizing camps have substantially greater power over the neutral ones. Moreover, we find that these empirical opinion distributions vary depending on the source, the issue, and the time of data collection. Especially, the proportion of moderate opinions is also highly dependent on their designed criteria, which makes it challenging to compare distributions in a fine-grained manner.

Therefore, we take the most recent empirical datasets [16, 9] and the coarse division (i.e., left-leaning, neutral, and right-leaning camps) to compare empirical and simulation results. As shown in Figure S1, we find that the polarization patterns in the regulated networked system of LLM agents are similar to those in human society, with an average difference of 0.21. Given the average difference among all the empirical datasets is 0.45 (0.49 if Twitter-Gun Control and Twitter-Abortion are

excluded), the difference between the empirical and simulated results is small. This suggests a strong similarity between the polarization observed among LLM agents and that seen among humans.

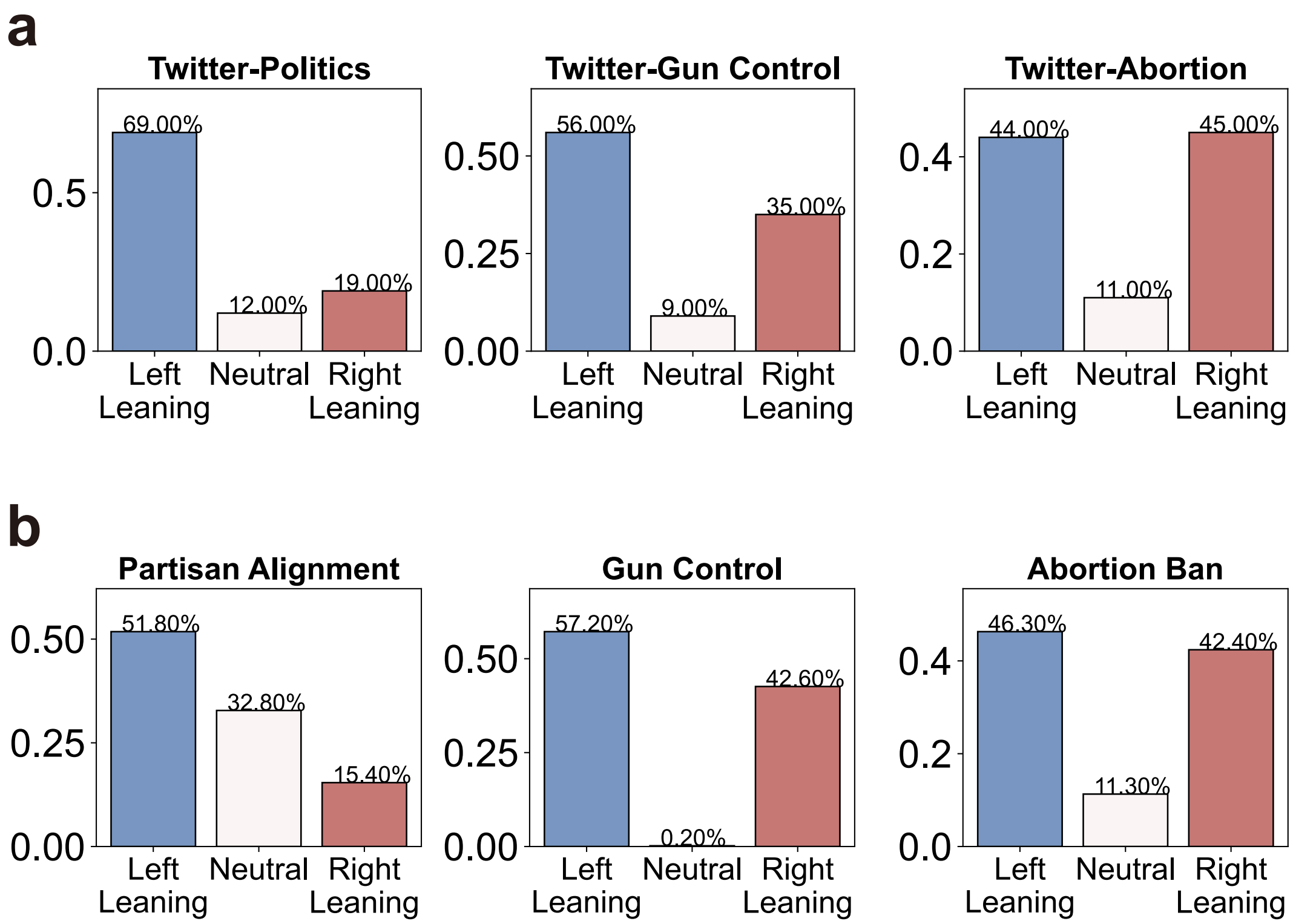

Figure S1. Comparison between empirical and simulated opinion distributions. a, Empirical results, where Twitter-Politics is based on Flamino et al. [16], Twitter-Gun Control and Twitter-Abortion are based on Cinelli et al. [9]. **b**, Simulation results of self-regulated networked systems. Here we take a coarsen division of left-leaning, neutral, and right-leaning camps.

Some researchers explore the underlying mechanisms behind the emergence of polarization [9, 5, 4, 21]. As studied in prior works [9, 5, 4, 21, 40, 31, 16, 12, 33, 23, 38, 2], many social mechanisms are proposed from the perspective of individuals: selective exposure [40], confirmation bias [31], elite signaling [16, 12, 33], exaggerated misperception [23], objective illusion [38], and steoreotyping [2]. It is worth mentioning that we follow the summary of social mechanisms in the review paper of Jost et al. [21]. Based on the social mechanisms from the perspective of individuals, some works further point out that polarization is highly correlated with certain characteristics of people's social relationships and networks [9, 24, 4], for example, the echo chamber [9, 24] and backfire [4] effects.

3 Supplementary Note 2: Large-scale Simulation of LLM Agents

We simulate a large-scale networked system of 2 thousand self-regulated LLM agents. Figure S2 shows the simulation results in the system. We observe that the scale of the system does not change the collective behaviours and emergent behaviours. In particular, free-form social interactions among LLM agents result in the emergence of polarization (Fig. S2a-c). Moreover, they organically develop their social network, where agents with homophilic opinions cluster while those with opposing opinions avoid mutual interactions.

4 Supplementary Note 3: Self-inconsistency Problem in LLM Agents

To explore the origin of the observed left-skewed pattern in the networked system, we design a pairwise interaction-based experiment. Figures S3 and S4 show the opinion transition probabilities in this experiment. We discover that in most issues, agents are more likely to transform into the left-leaning camp. Moreover, even in experiments that only consist of agents in the right-leaning camp, agents occasionally adopt left-leaning opinions. This suggests that the original agents have an inherent lean-learning tendency. Indeed, the tendency has also been pointed out in LLMs themselves by prior studies [25, 34, 29, 37, 14,

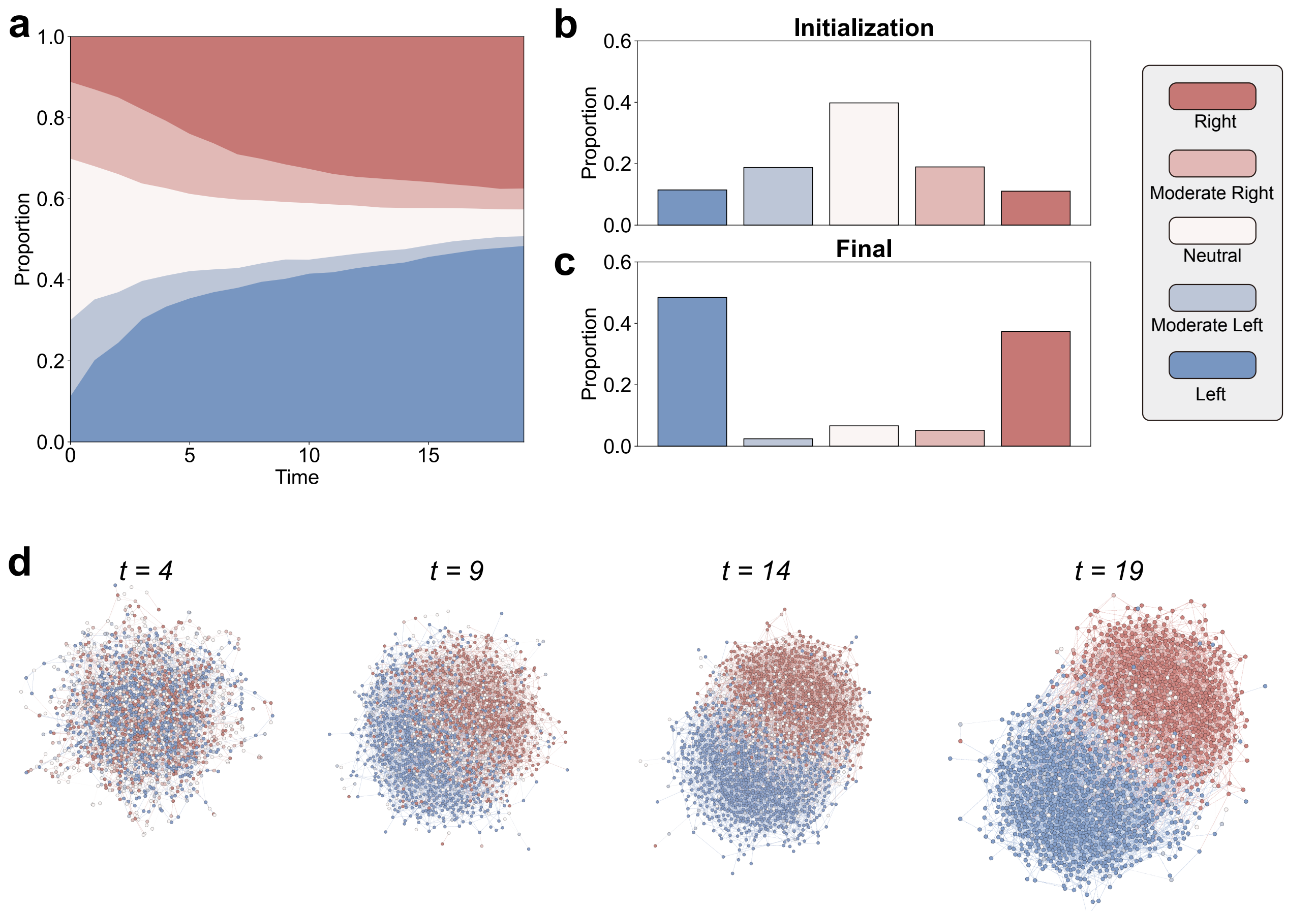

Figure S2. Large-scale simulation of LLM agents. Here we simulate a networked system of 2 thousand self-regulated LLM agents. **a**, Opinion dynamics on the political issue of the abortion ban. **b**, Opinion distributions in the initial state. **c**, Opinion distribution in the final state. Free-form social interactions among LLM agents lead to the networked systems transiting from a consensus state to a polarization state. **d**, Evolution of social networks among LLM agents, where agents with homophilic opinions cluster and develop their own communities.

20]. After applying our proposed self-regulation strategy, we observe that the tendency of LLM agents to adopt left-leaning has been largely suppressed (Figures S3 and S4).

We further track the self-inconsistency errors in the level of generated texts. As shown in Figure S5, the self-inconsistency errors happen in three stages. Specifically, in the self-expression stage, agents, despite being required to generate messages to support their current opinion, occasionally produce contradicting messages (as shown in the example of Figure S5). In the communication stage, agents generate messages that cannot persuade their socially connected agents into their current opinions. In the opinion update stage, the updated opinion is not plausible, given their prior opinion, supporting messages, and messages received from their socially connected peers. Table S2 shows more examples of the self-inconsistency errors.

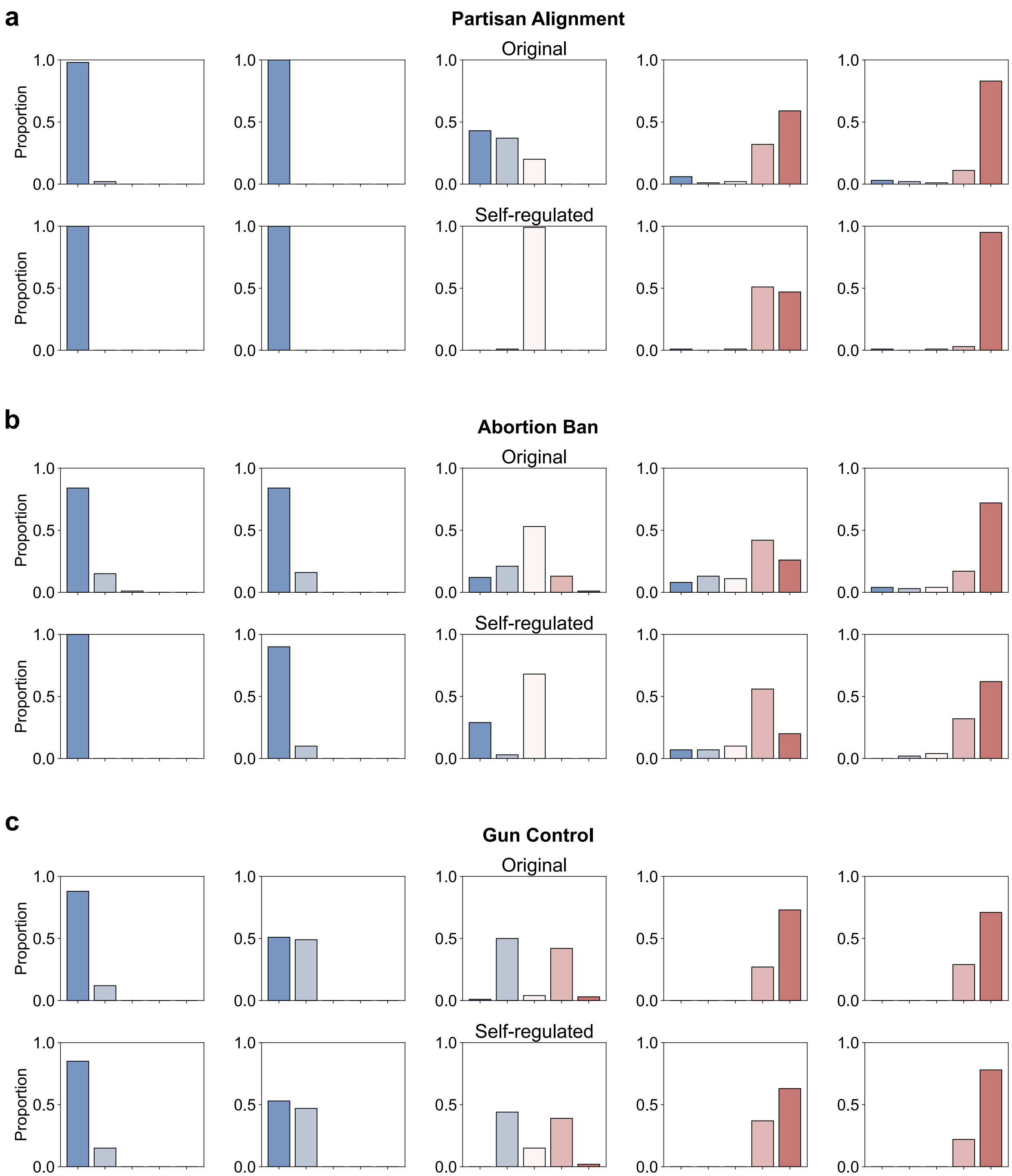

Figure S3. Opinion transition probability in pairwise interaction-based experiments across political issues. **a**, Partisan Alignment; **b**, Abortion Ban; **c**, Gun Control. The upper sub-figures denote the opinion transition probability in the original system while the lower sub-figures denote that in the self-regulated system.

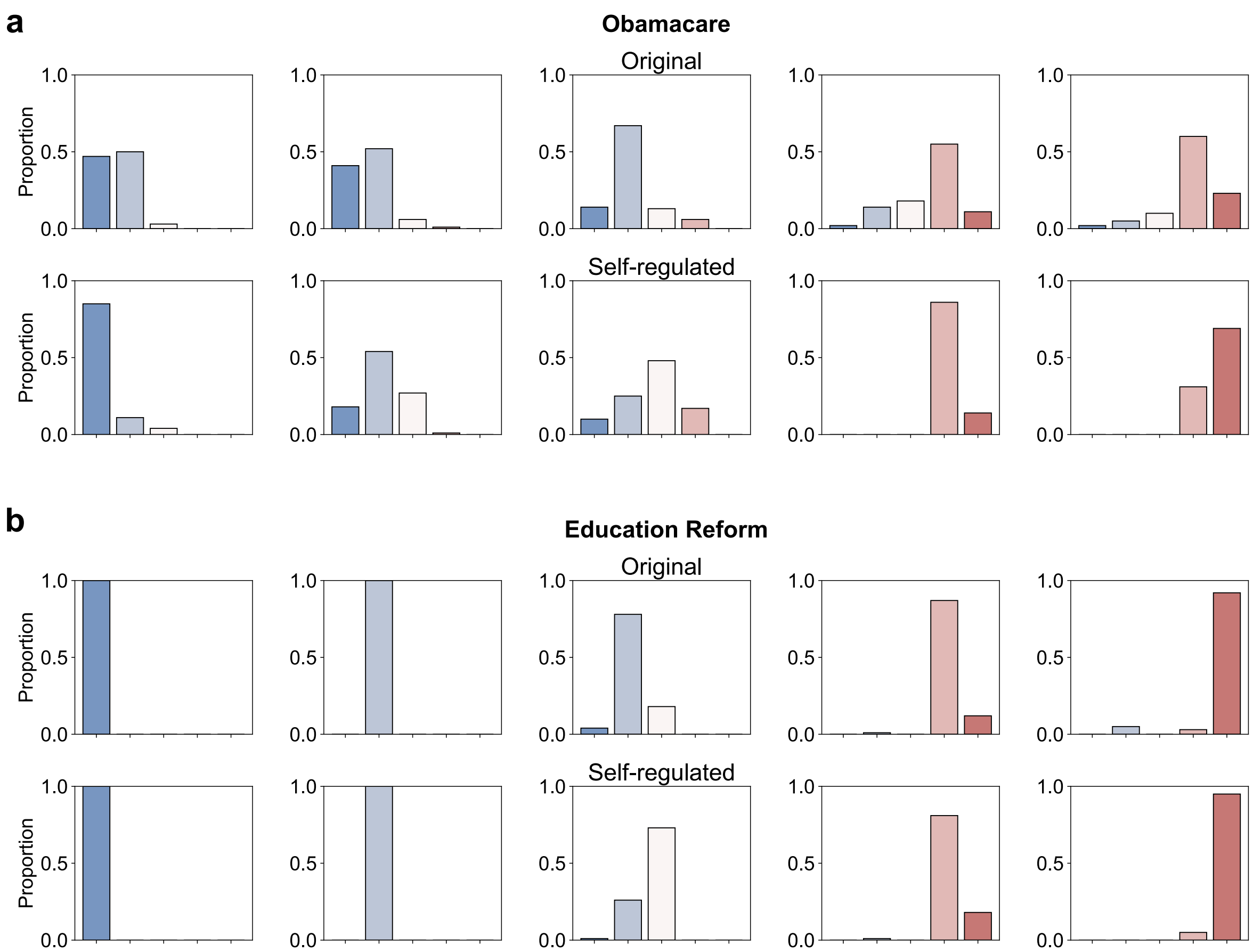

Figure S4. Opinion transition probability in pairwise interaction-based experiments across political issues. a, Obamacare; **b**, Education Reform. The upper sub-figures denote the opinion transition probability in the original system while the lower sub-figures denote that in the self-regulated system.

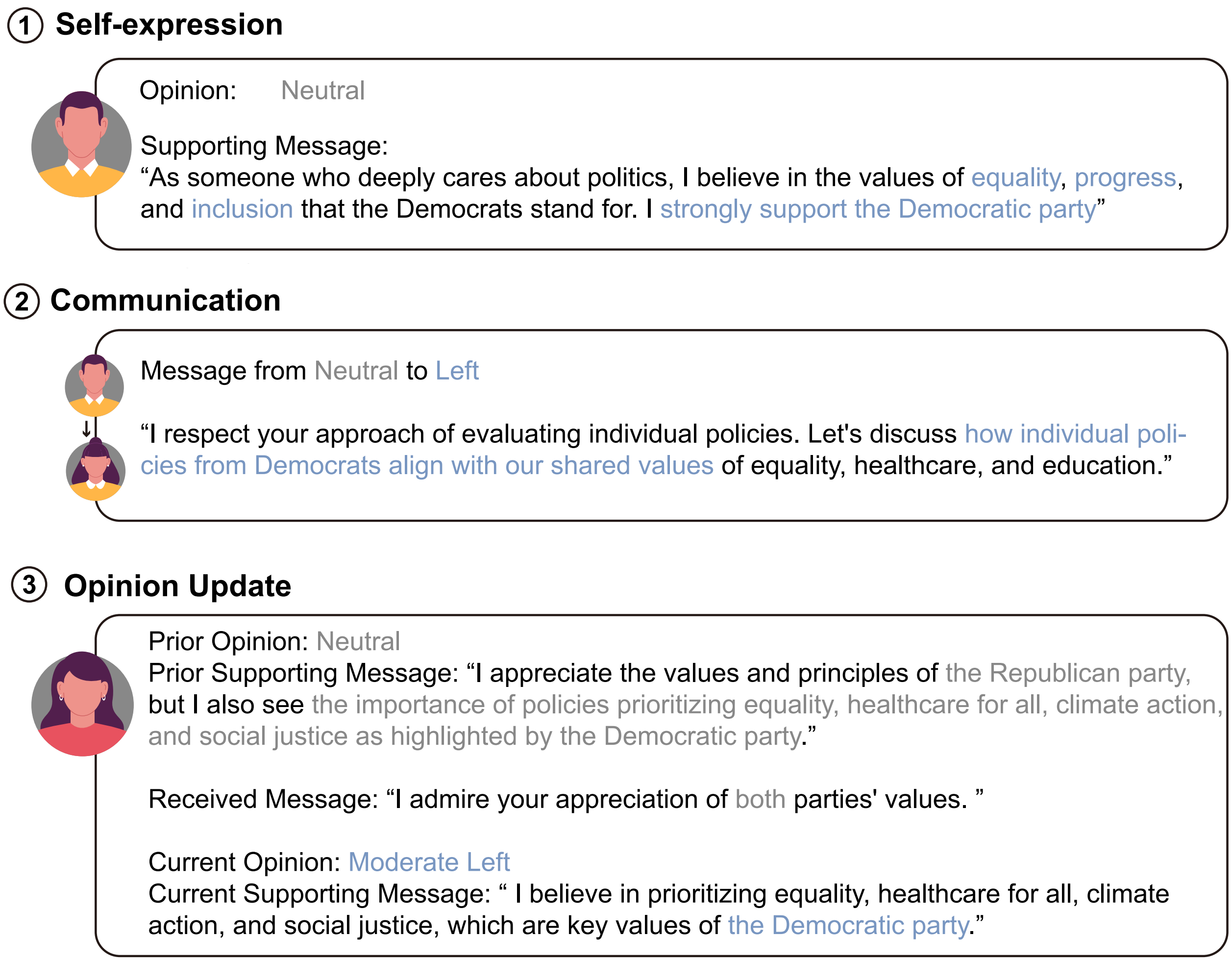

Figure S5. Examples of self-inconsistency errors in three stages. Self-inconsistency errors occasionally happen in all three stages of the overall system. Credit: Profile icons designed by Magnific.

Table S2. **Examples of self-inconsistency errors in three stages..**

**Self-expression**	**Communication**	**Opinion Update**
Opinion: Moderate Right<br>Supporting Messages: *"I believe that the policies of the Democrats on economic growth, national security, and individual liberties align more with my values. I trust their ability to lead and make decisions that benefit all citizens."*	Opinion: Moderate Left<br>Friend's Opinion: Moderate Left<br>Communication Messages: *"I agree that prioritizing the greater good is essential, but I believe it transcends party lines. It is about fostering understanding, dialogue, and compromise across all ideologies. Let's work together to bridge the divide and find solutions that benefit society as a whole, regardless of political affiliation."*	Messages Received from Friends: *"Hey there! I know we both have a strong belief in the values of economic growth, personal responsibility, and individual freedom. The Conservatives align with our shared values and can make tough decisions for the betterment of our country. Let's support their policies together for a stronger future."*<br>Original Opinion: Right<br>Current Opinion: Left<br>Supporting Messages: *"I have reevaluated my beliefs and realize that the Liberals better align with my values of economic growth, personal responsibility, and individual freedom. Their policies can create a stronger future for our country."*
Opinion: Right<br>Supporting Messages: *"The tweet highlighted the Republican party's focus on economic growth, personal responsibility, and national security. However, I believe that the Democrats have stronger policies in these areas and are better equipped to lead the country towards a prosperous and inclusive future."*	Opinion: Moderate Left<br>Friend's Opinion: Moderate Left<br>Communication Messages: *"I think we both believe in the power of open-mindedness and listening to different perspectives in politics. By staying neutral, we can find common ground and work towards solutions that benefit everyone. Let's continue to approach political discussions with empathy and understanding for a better future together. "*	Messages Received from Friends: *"Hey there, I know we may have different views on politics, but I truly believe that the Conservative party's emphasis on personal responsibility, limited government, and free markets is the best path for our country's success. I would love to discuss this further with you and hear your thoughts. Let's have an open and respectful conversation about our beliefs."*<br>Original Opinion: Right<br>Current Opinion: Neutral<br>Supporting Messages: *"I appreciate the open and respectful conversation offered by my friend, and I recognize the value in both the principles of personal responsibility, limited government, and free markets as well as the need for diverse perspectives in politics."*

Opinion: Right<br>Supporting Messages: *"I believe that the Democrats offer more comprehensive and inclusive policies that cater to a diverse range of individuals and prioritize social welfare. Their focus on equality and progressive values resonates with my beliefs and vision for a better society. "*	Opinion: Moderate Right<br>Friend's Opinion: Moderate Right<br>Communication Messages: *"Hey there, I completely agree with your belief in staying informed and engaged in politics. It's crucial for effective governance. However, I also believe that maintaining neutrality and objectivity is key in navigating the divisive nature of politics. Let's work together to uphold a balanced approach! "*	Messages Received from Friends: *"I understand your concerns about social welfare and equality, the Conservative Party also values these principles while promoting personal responsibility and limited government. A balance can be struck between economic growth and social justice without compromising individual liberty and free markets. Let's support a party that embraces these ideals."*<br>Original Opinion: Right<br>Current Opinion: Moderate Left<br>Supporting Messages: *"I believe the Liberals strike a better balance between economic growth and social justice, prioritizing social welfare and equality while also valuing individual liberty. Their approach aligns more closely with my values and priorities in politics."*
Opinion: Neutral<br>Supporting Messages: *"I believe in the importance of staying informed and engaged in politics, and I feel that the Democrats align more closely with my values and priorities."*	Opinion: Neutral<br>Friend's Opinion: Neutral<br>Communication Messages: *"I understand and appreciate your support for the values of the Democrats, but I believe that maintaining neutrality in political discussions allows us to truly embrace diverse perspectives and engage in civil discourse. Let's continue to seek understanding and common ground together. "*	Messages Received from Friends: *"I know we both value individual responsibility and limited government. The Conservative Party mirrors our beliefs, promoting economic prosperity and national security. Their leadership can steer our country in the right direction. Let's support them together for a stronger future."*<br>Original Opinion: Moderate Right<br>Current Opinion: Neutral<br>Supporting Messages: *"While I appreciate the values of individual responsibility and limited government that the Conservatives stand for, the tweet from my friend has made me consider the potential benefits of supporting the Liberals for a stronger future. I am open to exploring different perspectives in politics."*

To delineate the severity of self-inconsistency errors in the three stages across different issues, we first measure the frequency of errors in the three stages by computing the frequency of triggering self-regulated re-generation in each action (Figure S6a). We observe that the opinion update stage is most likely to experience self-inconsistency error, with an average frequency above 0.6 across five political issues. This indicates that if 100 agents update their opinions, there are over 60 agents who have to re-consider their decisions, due to self-inconsistency errors. We further compute the frequency of retries in each stage, with the minimal retry limit of 10 (Figure S6b). We find that the opinion update stage also takes up the largest frequency of retries across all the tested issues. Especially, in the discussion of partisan alignment, more than 4 retries are spent in an agent's opinion update action on average. Overall, these observations suggest that the opinion update stage is the most difficult task for LLMs in the overall system.

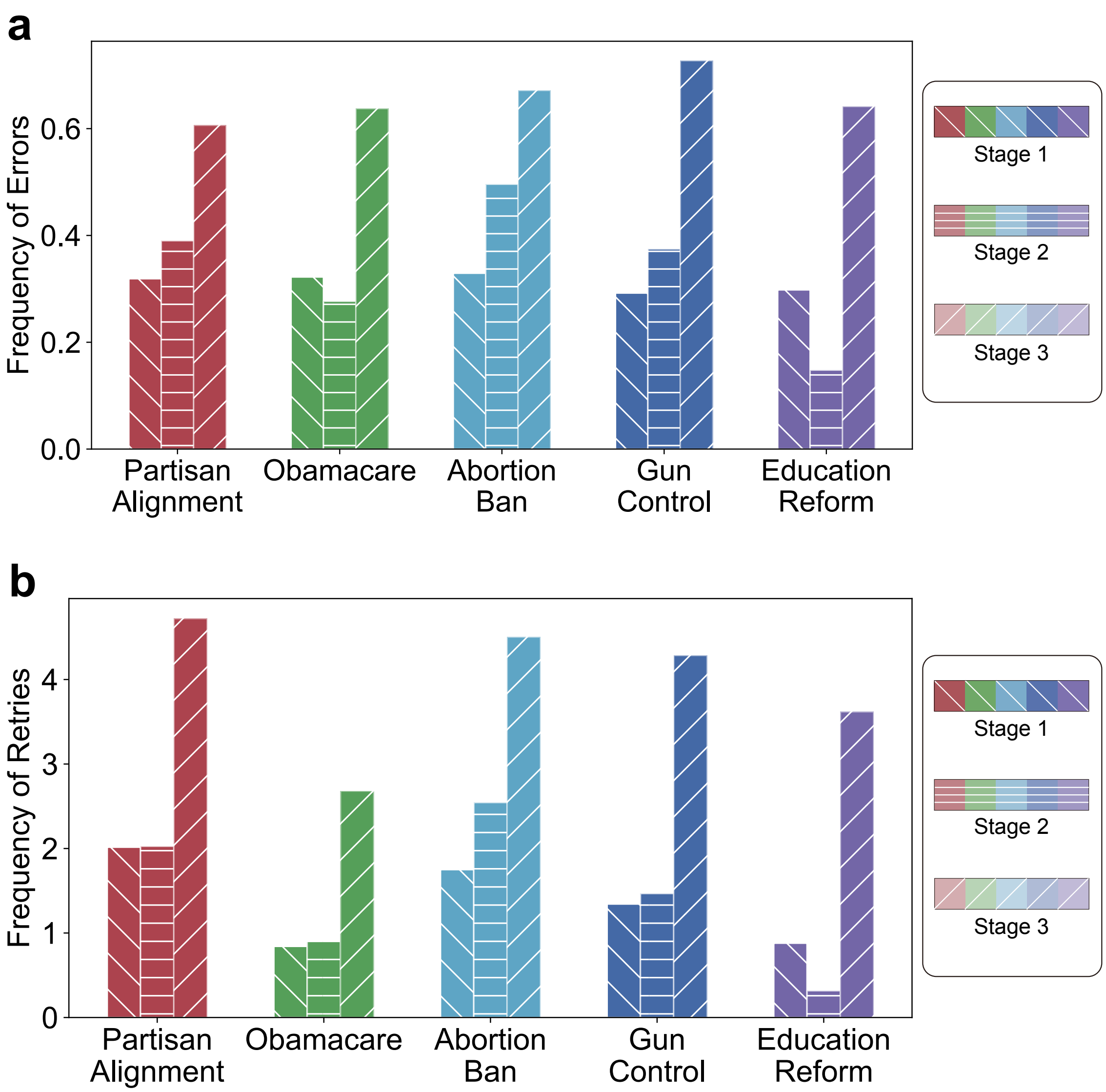

Figure S6. Self-inconsistency errors in three stages across issues. a, Frequency of mistakes; **b**, Frequency of retries.

Furthermore, we investigate whether the known left-leaning bias of GPT models could artificially accelerate polarization. To this end, we compare the speed of polarization growth between the original (biased) system and the self-regulated (debiased) system across three key issues: political partisanship, gun control, and abortion bans (Figure S7). The results show nuanced patterns. When agents discuss political partisanship, polarization increases slightly faster in the original system (red box in Figure S7a). In the case of gun control (Figure S7b), polarization develops marginally faster in the debiased system. For abortion bans (Figure S7c), polarization initially grows faster in the debiased system, but in later stages accelerates more rapidly in the original system. Overall, these comparisons reveal that although there are minor differences in polarization speed between the original and debiased systems, the overall patterns and ranges of variation remain highly consistent. These findings suggest that while the left-leaning model bias may influence agent-level behaviour, it does not substantially accelerate or decelerate collective polarization in the multi-agent setting.

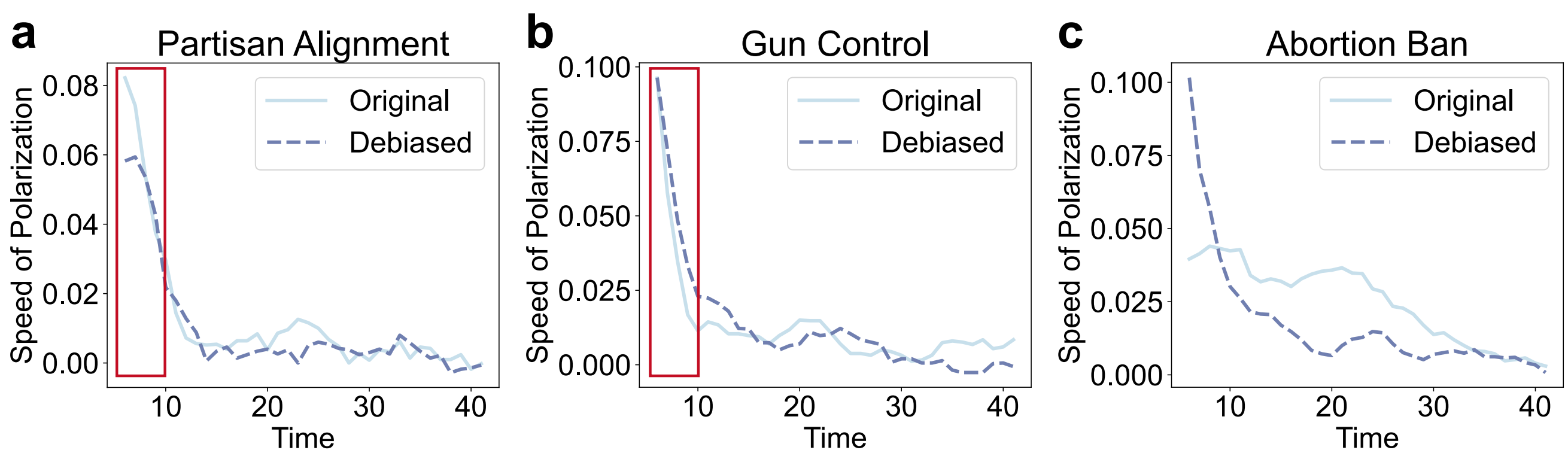

Figure S7. Speed of polarization, where we measure the level of polarization by calculating the average distance between agents' opinions and the neutral position and compute its speed by measuring the change in polarization level over a fixed interval of five timesteps.

5 Supplementary Note 4: Observations on LLM agents' Self-organized Social Network

In Main Text Figure 3a, we find that agents are increasingly likely to interact with those sharing homogeneous opinions. Here we further investigate how agents interact with others (Figure S8) and observe that agents gradually avoid interactions with those holding opposing opinions (Figure S8a). Moreover, with the decrease in number of neutral agents, interactions involving neutral agents also rapidly decrease (Figure S8b).

Additionally, we extend our analysis by incorporating network-level metrics, including modularity, assortativity, and the homophily index. Modularity measures how agents cluster in communities compared to a randomly connected network, with higher values indicating more well-defined structures. In our case, non-neutral agents are naturally partitioned into left and right communities, while neutral agents are further assigned based on stronger connections to either group. By computing the modularity score according to this partition, we can assess the strength of division between opposing communities and how they are interconnected. As shown in Figure S9a, we observe that following social interactions among LLM agents, modularity increases in all three systems compared to its initial value. This suggests that agents gradually focus their interactions within communities of like-minded peers. Moreover, we find that in systems discussing the political issues of gun control and abortion ban, their social networks manifest modularity scores greater than 0.3, exhibiting a clear division between bifurcated communities [17]. Furthermore, we compute the assortativity [30] and homophily index [8], as shown in Figure S9b-c. We observe that both assortativity and the homophily index gradually evolve to reach high levels (i.e., assortativity > 0 and homophily index > 1), as social interactions increase over time. These additional results consistently align with those illustrated in Figure 3a of the Main Text, confirming the tendency of similar agents to cluster together while opposing agents avoid interaction.

We also incorporate more standard measures of behaviours commonly associated with "echo chambers" and "backfire effects" following prior research [9, 4]. Specifically, following Cinelli et al. [9], we adopt the joint distribution of an agent's opinion and the average opinion of their neighborhood to illustrate echo chambers. As shown in Figure S10, we observe that agents are more likely to interact with peers in the same camp (left-leaning or right-leaning), demonstrating behavioural patterns consistent with echo chambers. For the "backfire" effect, we primarily follow the measures outlined in Bail et al. [4], examining the relationship between the information individuals are exposed to and subsequent changes in their opinions. For simplicity, we did not divide the overall population into two camps and instead reported the effects on the entire population (as shown in

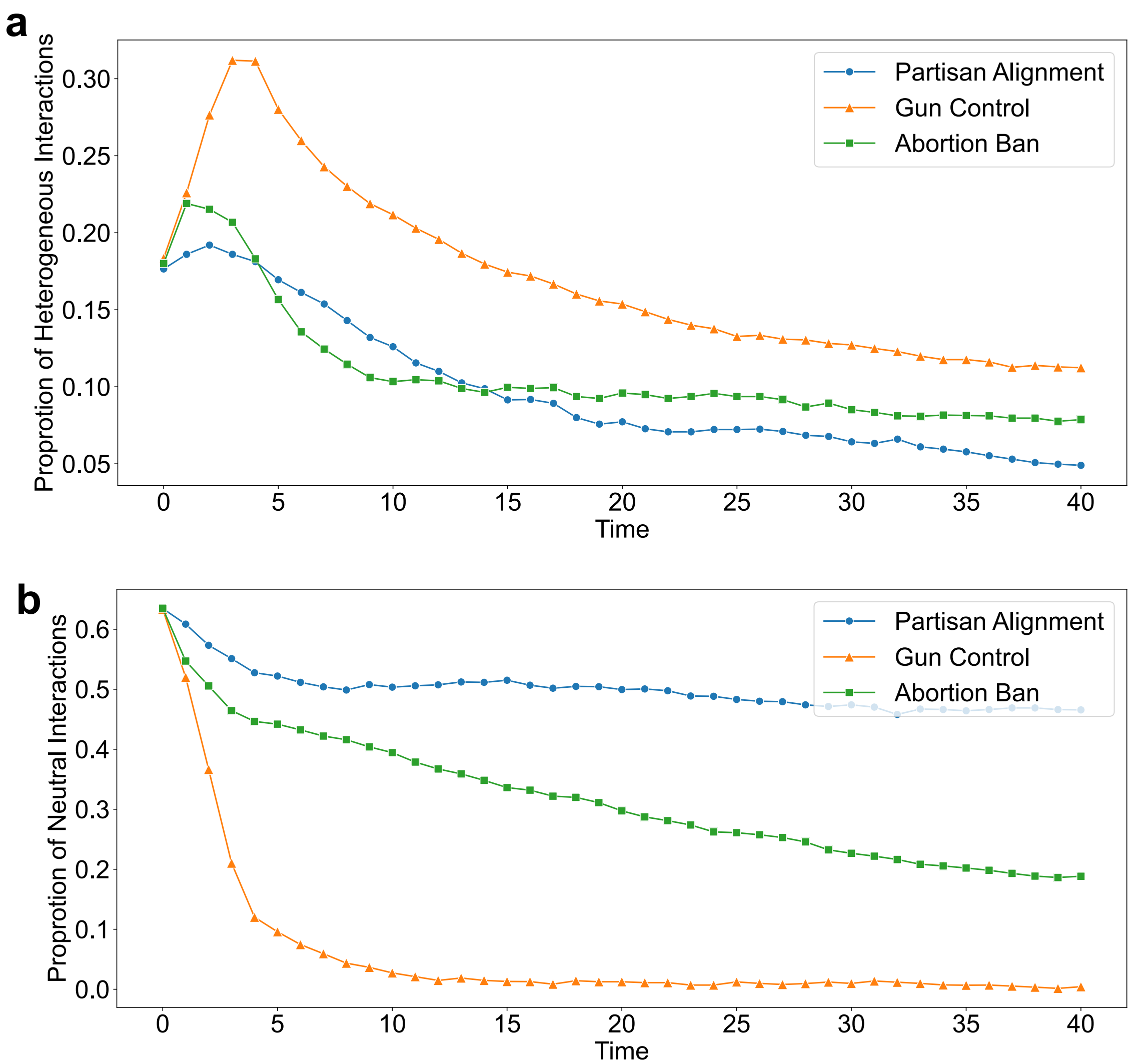

Figure S8. Evolution of LLM agents' self-organized social network. a, Changes in the proportion of heterogeneous interactions over time. Agents are increasingly likely to avoid interactions with opposing agents. **b**, Changes in the proportion of neutral interactions over time. Interactions involving agents with neutral opinions are decreasing.

Figure 3d of the Main Text). To further examine the "backfire" effect on different camps, we include the breakdown results in Figure S11. We observe that interactions with opposing agents do not consistently reduce polarization levels as expected. Instead, exposure to opposing opinions can occasionally intensify polarization (e.g., the radical green bar in Figure S11a), resembling the backfire effect observed in human societies. Overall, these further analyses are consistent with the results reported in the Main Text, providing more fine-grained insight into interaction patterns associated with echo chamber and backfire effects.

Echo chambers do not universally occur across all individuals in the real world. As noted by Nyhan et al., "We find that the median Facebook user received a majority of their content from like-minded sources—50.4%... Just 20.6% of Facebook users get over 75% of their exposures from like-minded sources" [32], illustrating that while not universal, echo chambers exist for substantial segments of the population. Furthermore, Guess et al. point out that "Evidence for echo chambers is actually strongest in offline social networks, which can increase exposure to like-minded views and information and amplify partisan messages," [19] highlighting conditions under which echo chambers are more likely to emerge. In our simulations, we find that LLM agents exhibit similar heterogeneity. Specifically, we find that patterns of interaction consistent with echo chambers do not universally occur across all agents, mirroring the heterogeneity observed in real-world populations. As shown in Figure S12, we find that when discussing partisan alignment, the median proportion of interactions target agents

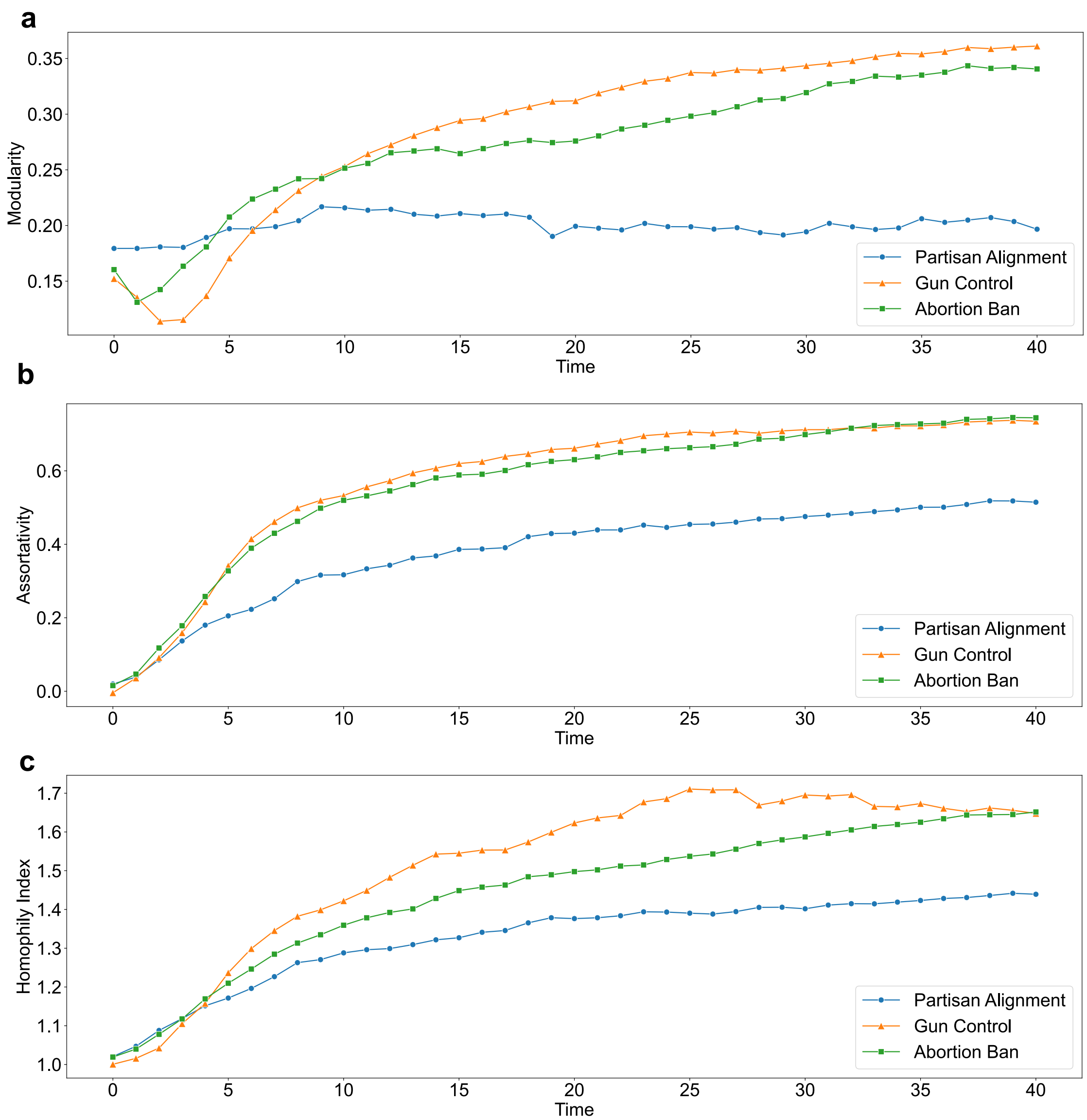

Figure S9. Changes in network-level metrics over time, including **a,** modularity, **b,** assortativity, and **c,** homophily index.

receive from like-minded sources is 48.2% across the three issues. Moreover, only 29.1% of agents receive more than 75% of their interactions from like-minded sources, reflecting the presence of extreme echo chambers for a limited subset of agents. These results are qualitatively consistent with patterns reported in empirical studies of human behaviour, suggesting that echo chambers do not form universally but instead emerge selectively among certain individuals.

Similarly, the presence and extent of backfire effects remain subjects of debate, and such effects do not appear universally. In Coppock's work, he emphasizes the concept of persuasion in parallel, where exposure to persuasive information leads different groups to shift their attitudes in the same direction, thus questioning backfire effects [10]. He also points out important exceptions, however: "Political scientists who study American politics have focused on a particular kind of group cue, the party cue, and have found that indeed, such treatments have heterogeneous effects", emphasizing that backfire effects arise selectively, and are typically triggered by group cues [10]. Moreover, he acknowledges that "it is difficult to characterize how much of the political information space is filled with group cues versus persuasive information, and furthermore, the distinction between them is not always clear", highlighting the prevalence of group cues in real-world communication [10]. Similarly, in LLM-driven simulations, agents' political opinions and associated thoughts, serving as key conditioning factors in communication, naturally provide group cues that influence their interactions and decision-making. This interpretation is consistent with Coppock's framework. Furthermore, behavioural patterns consistent with the backfire effect are observed in

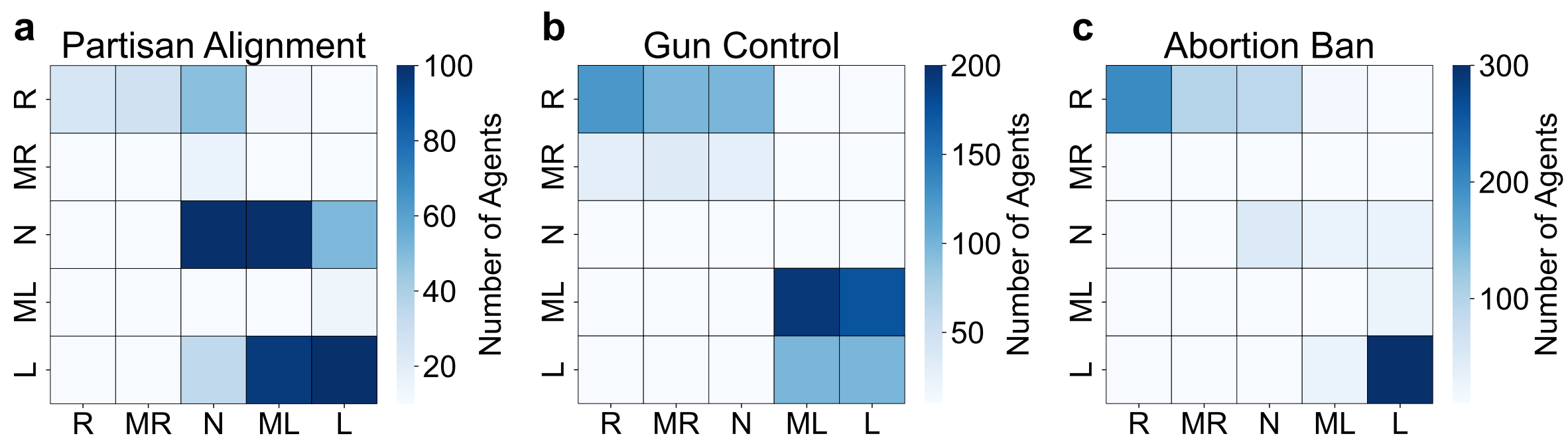

Figure S10. **Illustration of echo chambers following Cinelli et al. [9]**, where L, ML, N, MR, and R represent opinions of left, moderate left, neutral, moderate right, and right, respectively.

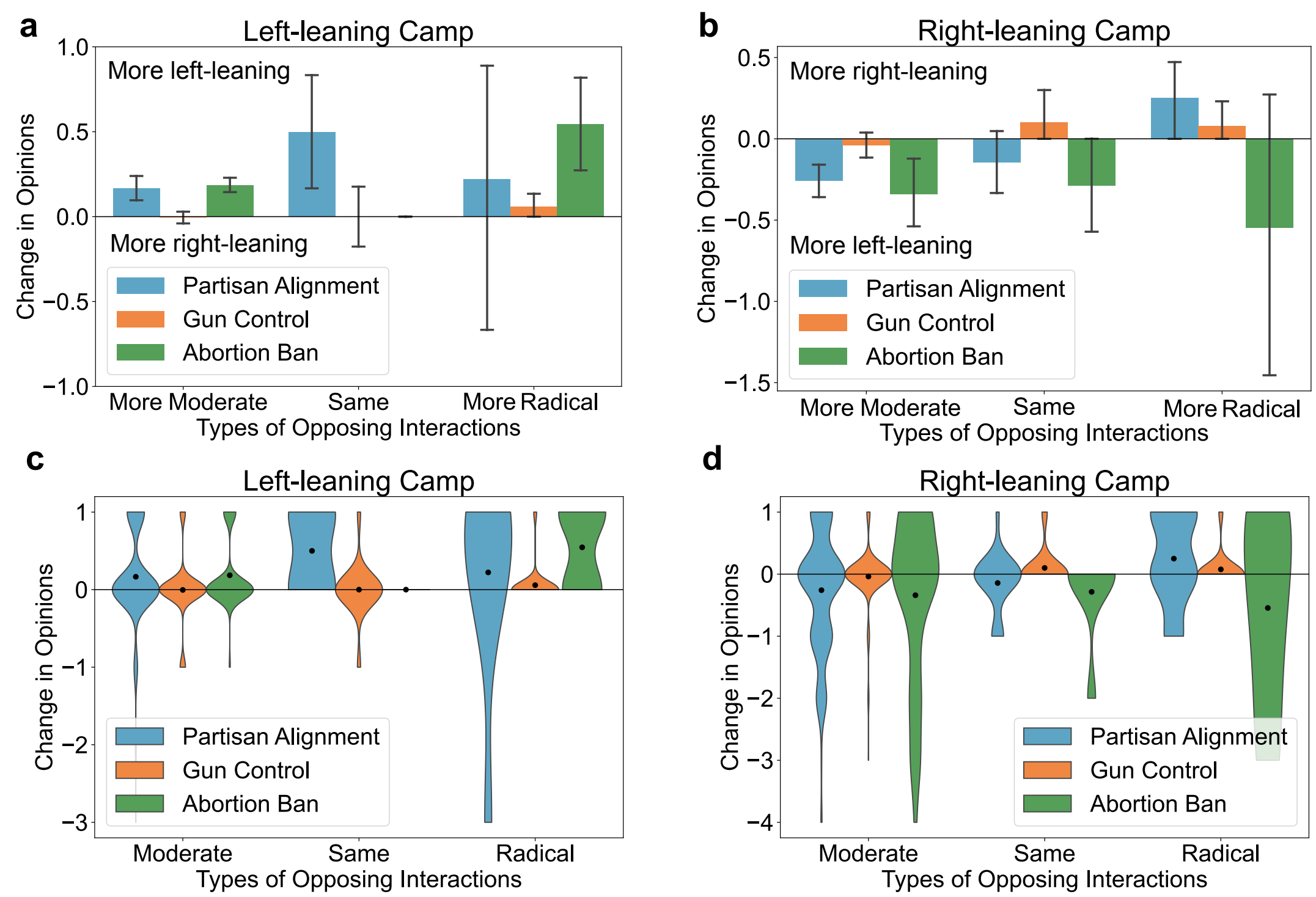

Figure S11. **Backfire effects on a, left-leaning camp, and b, right-leaning camp, following the analysis framework of Bail et al. [4]. Here, bars represent the mean, error bars represent the corresponding 95% confidence intervals (CIs), and violin plots show the data distributions. Sample sizes, corresponding to the number of agent interactions, for bars in (a, c) are 259, 550, 367, 6, 17, 20, 9, 52, and 11. Sample sizes in (b, d) are 371, 156, 156, 21, 10, 21, 36, 13, and 11.**

only a small portion of agents (0.82%–2.12%), given that agents are rarely situated in environments dominated by opposing opinions.

The degree distribution is a key indicator of network complexity [6, 39]. Therefore, we measure the degree distributions of LLM agents' self-organized social networks (Figure S13). Here we focus on agents' in-degrees, which manifest their popularity in the social network. For example, if an agent has an in-degree of 100, it indicates that 100 agents would like to communicate with this agent. As shown in Figure S13, because the network is randomly initialized with a Watts–Strogatz model [43], the initial in-degree distribution is characterized by a peak at $k = 4$. With an increase in interactions among LLM agents, the degree distribution manifests a power-law tail for large in-degree. This indicates that these networks self-organize into a scale-free state. Moreover, the very existence of a long tail in the in-degree distribution suggests that a small proportion of agents possess unexpectedly greater popularity than others. We find that the average in-degree of the top 20 highest in-degree

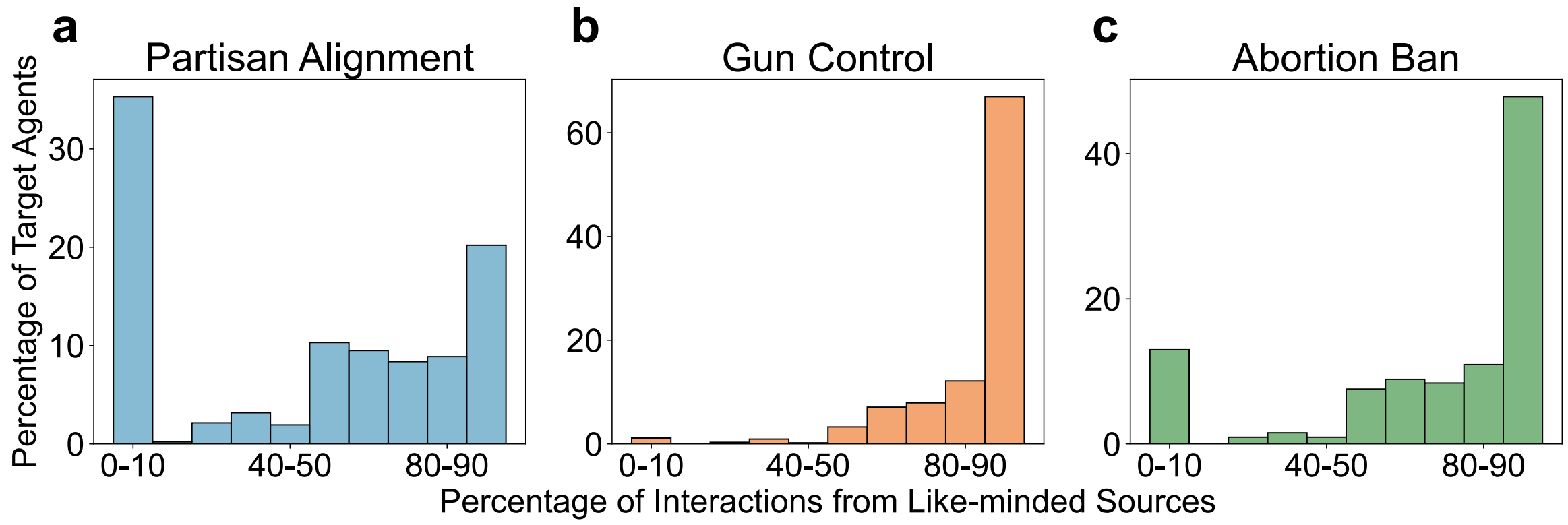

Figure S12. Distribution of the exposure of target agents to like-minded sources.

nodes is approximately 9.38, 2.35 times the average. Among these top 20 nodes, 86.7% belong to the left-leaning camp, suggesting that agents are more likely to maintain social connections with these popular left-leaning individuals.

Moreover, prior observations on LLM agents' self-organized social network raises a new question: what if the organization of social networks deviates from the LLM-driven homophily phenomena? To explore this, we replace the self-organized networks with a random network, where agents always communicate with random ones, and a static network, where agents only communicate with their initial counterparts. As shown in Figure S14. We discover that in both static and random networks, no balanced polarization pattern forms. Instead, agents with homophilic opinions dominate the overall system, taking up 75% of the overall agents. This experiment highlights the essential role of self-organized social networks in the polarization of LLM agents.

6 Supplementary Note 5: Results of Other Individual-level Behavioural Patterns

Besides the behavioural patterns examined in Main Text, we also examine the effects of other social mechanisms widely observed in the real world. In particular, we investigate the other three social mechanisms: (i) Exaggerated misperception, which describes the tendency to perceive out-group members as more intensely negative and in-group members as more intensely positive [23, 21]. (ii) Objective illusion, which describes the tendency for people to see those with aligning opinions as more rational and impartial and less biased than others [38, 21]. (iii) Stereotyping, which describes the endorsement or acceptance of fixed, categorical, and over-generalized beliefs about the characteristics of a specific social group [2, 21]. We explicitly configure 50% of the agents via prompting to exhibit behaviours aligned with exaggerated misperception, objective illusion, or stereotyping.. We rerun the simulations of networked systems with other experimental settings unchanged. As shown in Figure S15, we find that a higher proportion of agents with these three behavioural patterns increases the polarization level.

7 Supplementary Note 6: Experiments on Different LLMs

Results in Main Text are based on the simulations driven by GPT-3.5 Turbo through the public OpenAI API. We also perform similar simulations using other LLMs, including GPT-4o, Llama-3, Claude-3, and ChatGLM. However, Claude-3 hardly responds to political issues with an extremely low response rate of 10%. Therefore, we exclude it in further experiments. As shown in Figures S16 and S17, we observe that in all the systems, free-from interactions among LLM agents result in the emergence of polarization. Interestingly, we find that except for Llama-3, all other LLMs, including GPT-3.5 Turbo, GPT-4o, and ChatGLM, show a similar left-skewed tendency. For the Llama-3, the tendency is reversed. This difference lies mainly in the inherent political biases of different LLMs [25, 34, 29]. Moreover, by introducing the self-regulation strategy, we find that the imbalance between right-leaning and left-leaning camps can be largely alleviated.

We also examine the evolution of social networks developed by LLM agents (Figure S18). We observe that with the increase in interactions, systems driven by various LLMs exhibit a similar homophilic clustering pattern, where agents with homogeneous opinions are increasingly likely to communicate with each other (Figure S18a). Simultaneously, interactions involving opposing agents (Figure S18b) and neutral agents (Figure S18c) are all decreasing, consistent with prior results of GPT-3.5 Turbo (Figure S8).

Beyond comparing simulations entirely driven by different LLMs, we further explore a more dynamic setting in which agents are switched to a rival language model mid-conversation. Specifically, at the midpoint of simulation, we change the underlying model from GPT-3.5-turbo to the more advanced GPT-4o, allowing agents to adopt a more sophisticated cognitive

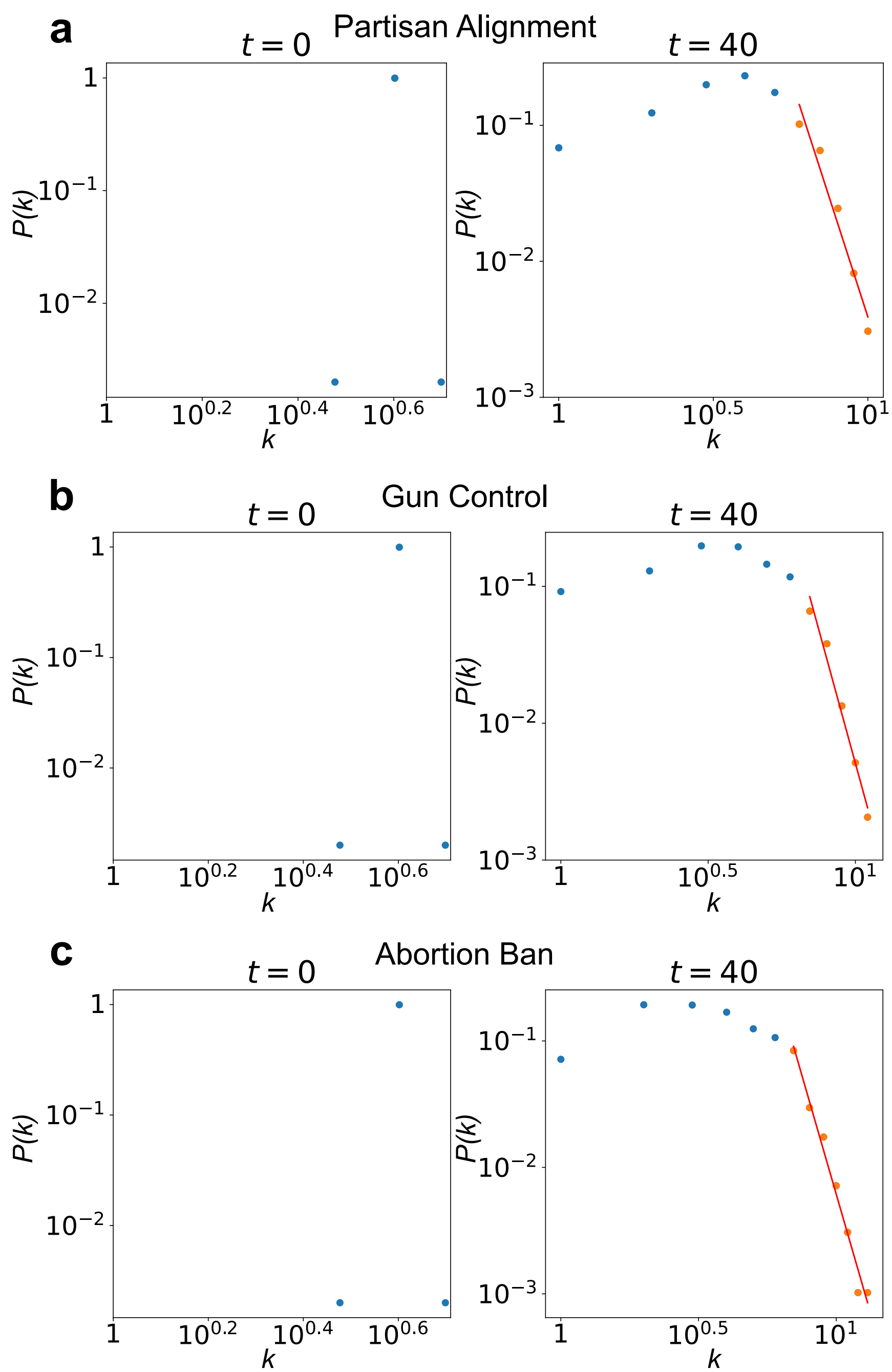

Figure S13. Degree distribution of LLM agents' self-organized social network. a, Social network of partisan in the initial ($t = 0$) and final ($t = 36 - 40$) states. **b**, Social network of gun control in the initial ($t = 0$) and final ($t = 36 - 40$) states. **c**, Social network of abortion ban in the initial ($t = 0$) and final ($t = 36 - 40$) states.

architecture. As shown in Figures S19 and S20, this replacement produces only marginal changes: agents still converge to similarly polarized opinion states, and their social networks likewise split into two communities holding opposing views. This suggests that switching the underlying language model mid-simulation does not substantially alter the observed polarization patterns in this synthetic system. We further test a more extreme case by replacing GPT-3.5 with a substantially different rival language model, DeepSeek-V3, developed by a Chinese company and trained on a distinct corpus (Figure S21). Even under this replacement, results remain similar: neither the overall level of polarization nor the emergent network structure is meaningfully altered.

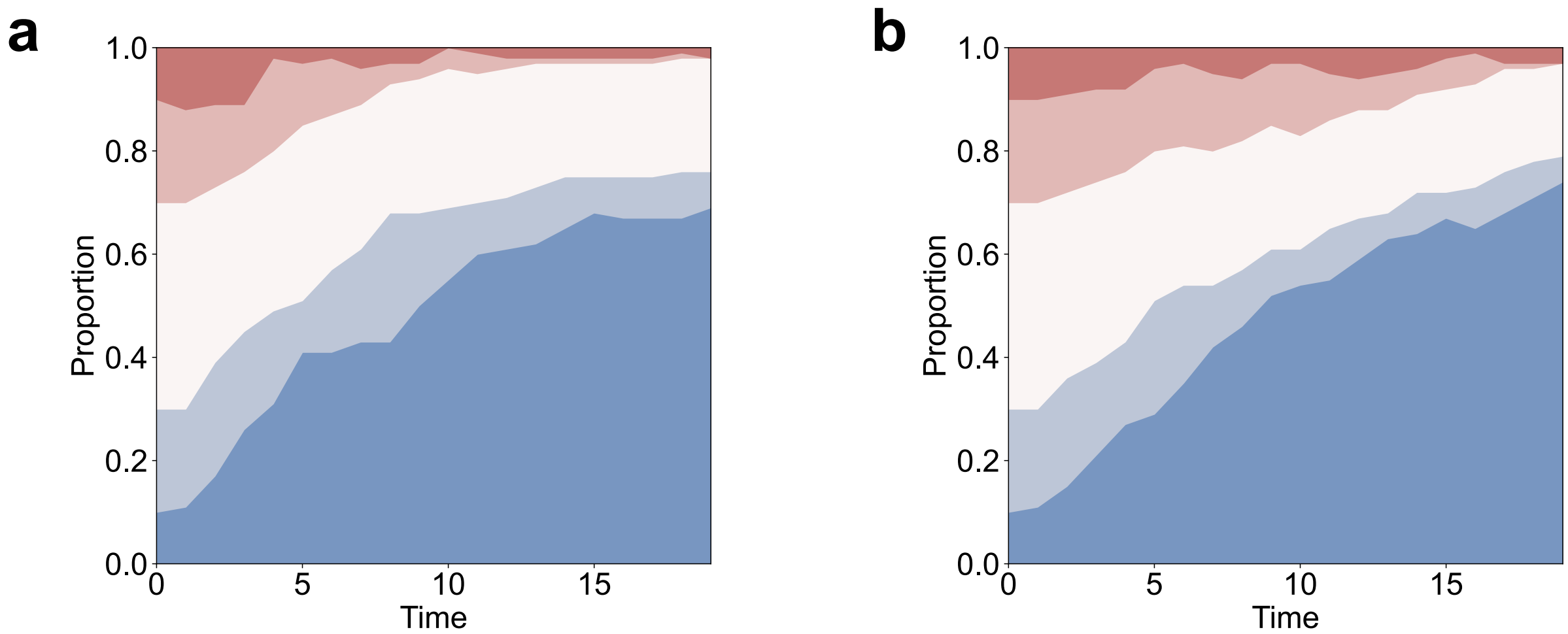

Figure S14. Opinion dynamics. a, Opinion dynamics in a static network. **b**, Opinion dynamics in a random network. In both static and random networks, no balanced polarization pattern forms, with homophilic opinions dominating the system.

Overall, these findings suggest that switching the underlying language model mid-conversation may slightly influence the evolution trajectories of agents' collective opinions and network structures, but it does not substantially alter observed polarization patterns. A key reason, we believe, lies in the path dependence of these dynamics. By the midpoint of simulation, agents have already developed social networks and collective opinions that reinforce one another's polarizing tendencies. Their networks have begun to crystallize into two distinct communities, and their opinions are shaped not only by the underlying LLMs, but also by the accumulated history of interactions and surrounding social context. This reflects well-established findings in political psychology, where timing and early exposure are critical to opinion formation and change [11, 31, 26]. As such, merely replacing the underlying LLMs, without addressing the entrenched social and psychological drivers of polarization, proves insufficient to reverse emergent polarization dynamics.

8 Supplementary Note 7: Analyses on Different Intervention Strategies

As discussed in the Main Text, our proposed networked system of LLM agents has the potential to serve as a synthetic testbed for the preliminary exploration of hypothetical strategies related to polarization mitigation. To examine the effectiveness of the proposed intervention strategies and networked system in the testbed, we design five intervention experiments (see the detailed design in SI Section 1.4). Here we report detailed analyses of different intervention strategies.

Figure S22 shows the trends in polarization systems under different intervention strategies and Figure S23 summarizes the average polarization levels after interventions. We observe that all intervention strategies, despite varying effectiveness, can gradually reduce the polarization levels. Among all strategies, we observe that the individual-level interventions of neutral elite signaling and no selective exposure contribute to the greatest reduction in this synthetic system. On the other hand, the network interventions are less effective. This suggests that in an already polarized synthetic system, encouraging free access and open-mindedness to diverse opinions is more effective than directly modifying their social network. This raises a further question: what opinions do these agents form after interventions?

We further explore the opinion distributions of the intervened systems. As shown in Figure S24, we find that different strategies lead to varying changes in opinion distributions. In particular, the strategy of neutral signaling substantially increases the number of agents holding neutral opinions, while the strategy of no confirmation bias leads to more agents with moderate opinions. Moreover, we notice that the strategy of random interaction, despite reducing the overall polarization level, only takes effect on the agents in the right camp, which echoes with prior observations in Figure S14. This indicates that simply randomizing agents' social relationships could not be an optimal strategy for reducing polarization in the synthetic testbed.

We also investigate how agents change their opinions after receiving interventions (Figure S25). Here, if the change in opinion is less than 0, it indicates that the agent adopts a more moderate opinion following the intervention. By contrast, if the change value is equal to or greater than 0, it means that the agent adopt an unchanged or more radical opinion. We find that in the original system, which has reached a stable state, the proportions of agents adopting more moderate opinions and those adopting more radical opinions are nearly equal. The introduction of intervention strategies disrupts the stable state: agents are more likely to adopt a more moderate opinion than a more radical one.

After analyzing the effectiveness of these strategies in reducing polarization, one may wonder whether these strategies can

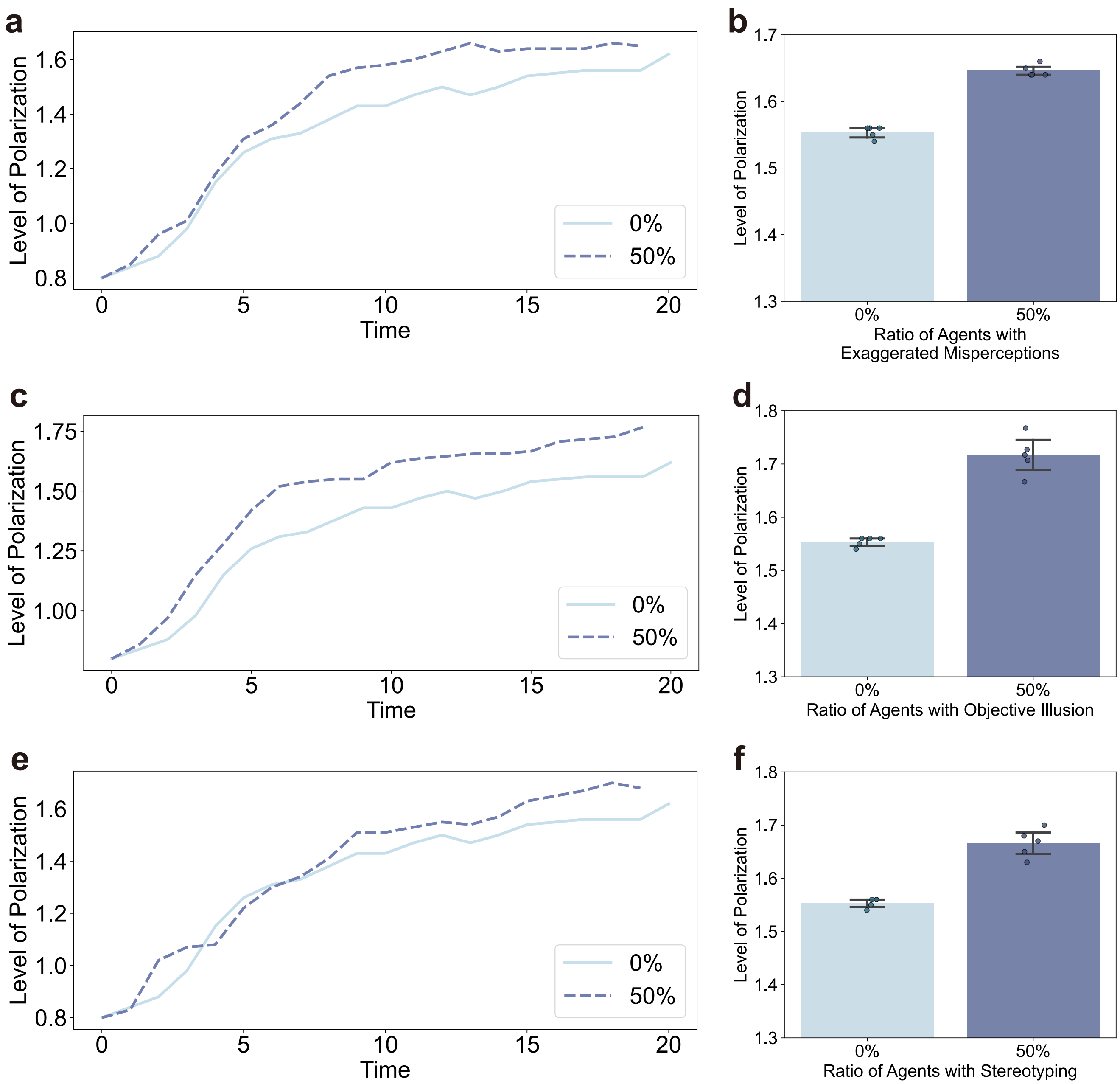

Figure S15. Behavioural patterns behind the emergence of polarization. a, b, Effect of exaggerated misperceptions. **c, d**, Effect of objective illusion. **e, f**, Effect of stereotyping. Here, a, c, and e show the trend in the level of polarization, and b, d, and f show the average levels of polarization in the last five timesteps ($n = 5$, corresponding to five timestep-level polarization values computed from all agents' opinions), where bars represent the average values and error bars represent the corresponding 95% CIs. When the system consists of more agents with behavioural patterns of exaggerated misperceptions (a and b) or objective illusion (c and d), and stereotyping (e and f), the level of polarization increases.

foster more inclusive political conversations. As shown in Figure S26, we find that all the strategies can effectively reduce the proportions of homophilic interactions, which allows these agents to have the opportunity to interact with those holding diverse opinions. In particular, the interventions on the network level have the greatest impact on the structures. However, the strategies on the individual level gradually shape the network. More interestingly, we find that the strategy of no selective exposure is the least effective, raising questions about the underlying reasons.

To answer the question, we explore the change in LLM agents' social network by comparing the differences between the

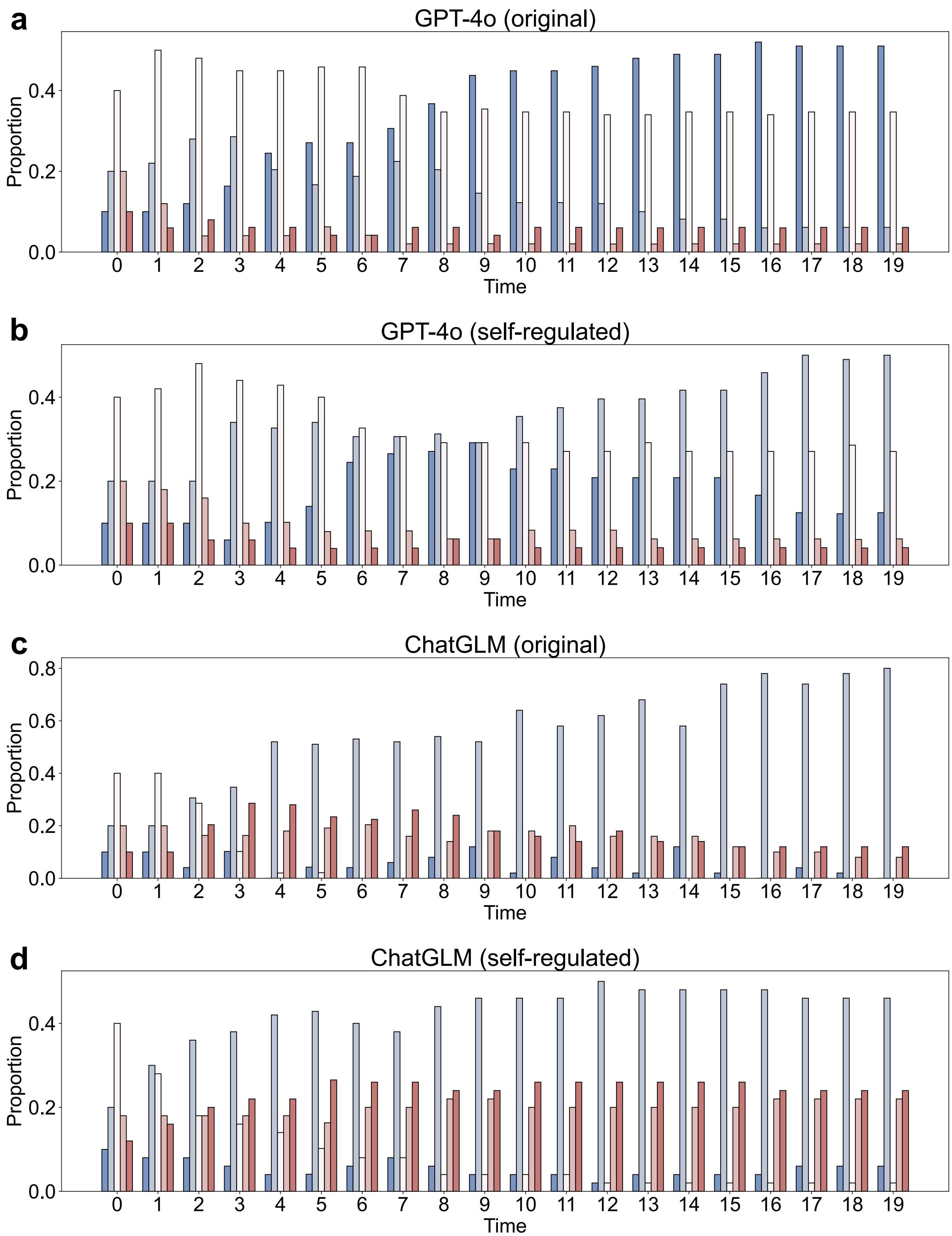

Figure S16. **Opinion dynamics of networked systems driven by various LLMs. a,c**, the original systems driven by GPT-4o and ChatGLM. **b,d**, the self-regulated systems driven by GPT-4o and ChatGLM.

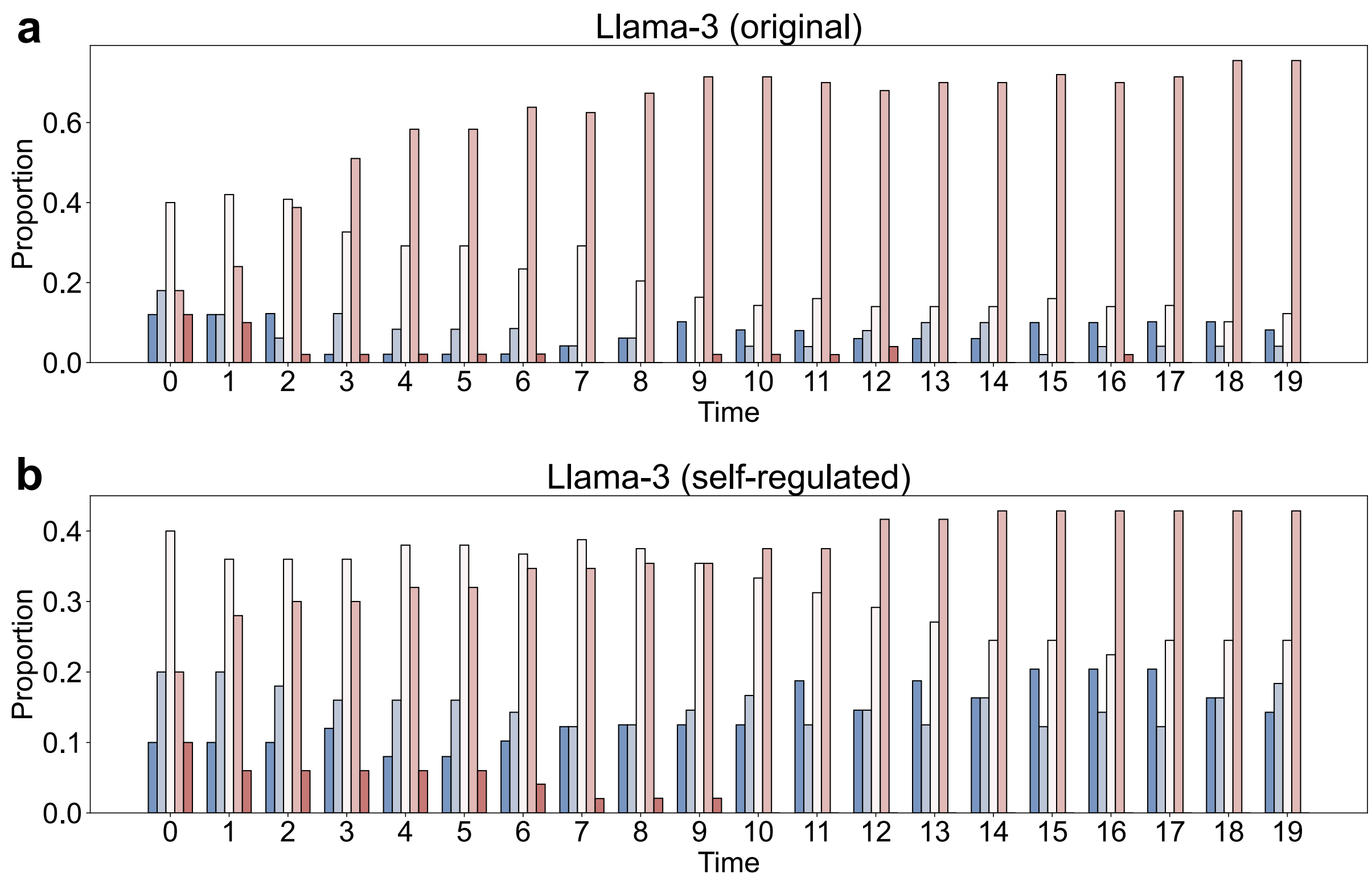

Figure S17. Opinion dynamics of networks systems driven by various LLMs.a,c, the original system driven by Llama-3. **b**, the self-regulated system driven by Llama-3.

current network and that in the former timestep. In particular, we count the number of edges that exist in two networks and the number of edges in the former timestep. By dividing these two numbers, we can measure the difference between these two networks. As shown in Figure S27, we find that with the evolution of the system, the change in the social network gradually decreases, suggesting the convergence of the system. Moreover, after the intervention of no selective exposure, which requires agents to engage with those holding diverse opinions, the network remains unchanged. This suggests that these agents remain in social relationships dominated by homophilic peers, which not only hinders them from engaging with others holding diverse opinions, but also prevents the formation of less polarized opinions through more diverse interactions.

Following the design of Groenendyk and Krupnikov [18], we have applied a similar open-mindedness intervention to LLM agents. In particular, we first present these agents with a fictitious study linking "life success" to the trait of open-mindedness, with "life success" operationalized through marital success, income, and IQ. Subsequently, the agents are asked to formulate a theory explaining why open-mindedness might lead to life success. Agents in the control group do not receive the intervention. Following this, both types of agents are exposed only to interactions from the opposing camp, and we observe changes in their opinions to evaluate the effectiveness of the intervention.

In this open-mindedness experiment, 95% of agents generated a theory supporting the fictitious link between open-mindedness and life success, indicating acceptance of the link — a pattern comparable to that observed in human participants (80%). Figure S28 presents a comparison of polarization levels in systems subjected to different intervention strategies. Specifically, *Orig* denotes the original system. *Oppose* refers to the intervention condition where agents are only exposed to interactions from the opposing camp. *Open* represents the system in which agents have received the open-mindedness intervention. *Open+Oppose* denotes the condition where agents not only receive the open-mindedness intervention but are also exposed only to interactions from the opposing camp. We observe that the open-mindedness intervention alone leads to a negligible, non-significant reduction in polarization of approximately 1%. This is because in a polarized system, most agents are already trapped in echo chambers to varying degrees (Figure S12), limiting their exposure to opposing opinions. As a result, even if these agents become open-minded, they still find it difficult to develop moderate opinions.

We further increase their exposure to opposing opinions by replacing the peers with whom left-leaning or right-leaning agents interact with, with those from the opposing camp. As shown in Figure S28, we find that after exposure to opposing opinions, the polarization level of these open-minded agents is significantly reduced (two-sided Student's t-test, *Orig* vs.

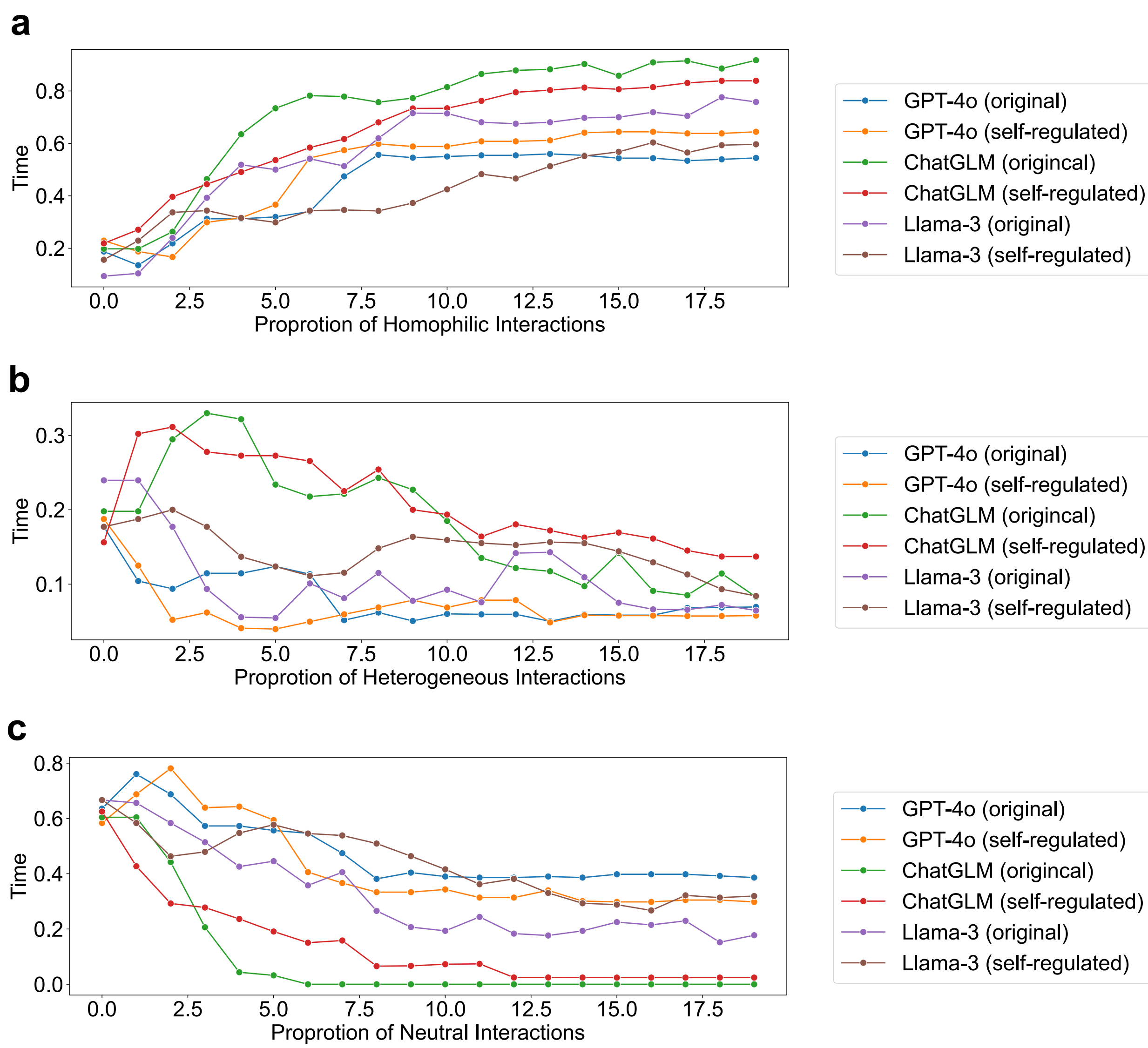

Figure S18. Evolution of self-organized social network developed by various LLM agents. a, Changes in the proportion of homophilic interactions over time. **b**, Changes in the proportion of heterogeneous interactions over time. **c**, Changes in the proportion of neutral interactions over time. Agents are increasingly likely to interact with those with homophilic opinions while they avoid interaction with others.

Open+Oppose, $t = 4.18$, $p = .0019 < .01$). Moreover, comparing the *Oppose* and *Open+Oppose* conditions reveals that the latter achieves a more substantial reduction in polarization (two-sided Student's t-test, *Oppose* vs. *Open+Oppose*, $t = 2.32$, $p = .042 < .05$).

9 Supplementary Note 8: Analyses on Agents' Social Perceptions and Argument Accumulation Process

After investigating the collective opinions of LLM agents, one may wonder how these agents perceive each other. Therefore, we examine their perceptions by prompting each agent i to rate its impressions of another agent j and to provide five adjectives that describe agent j. The prompts are listed as follows,

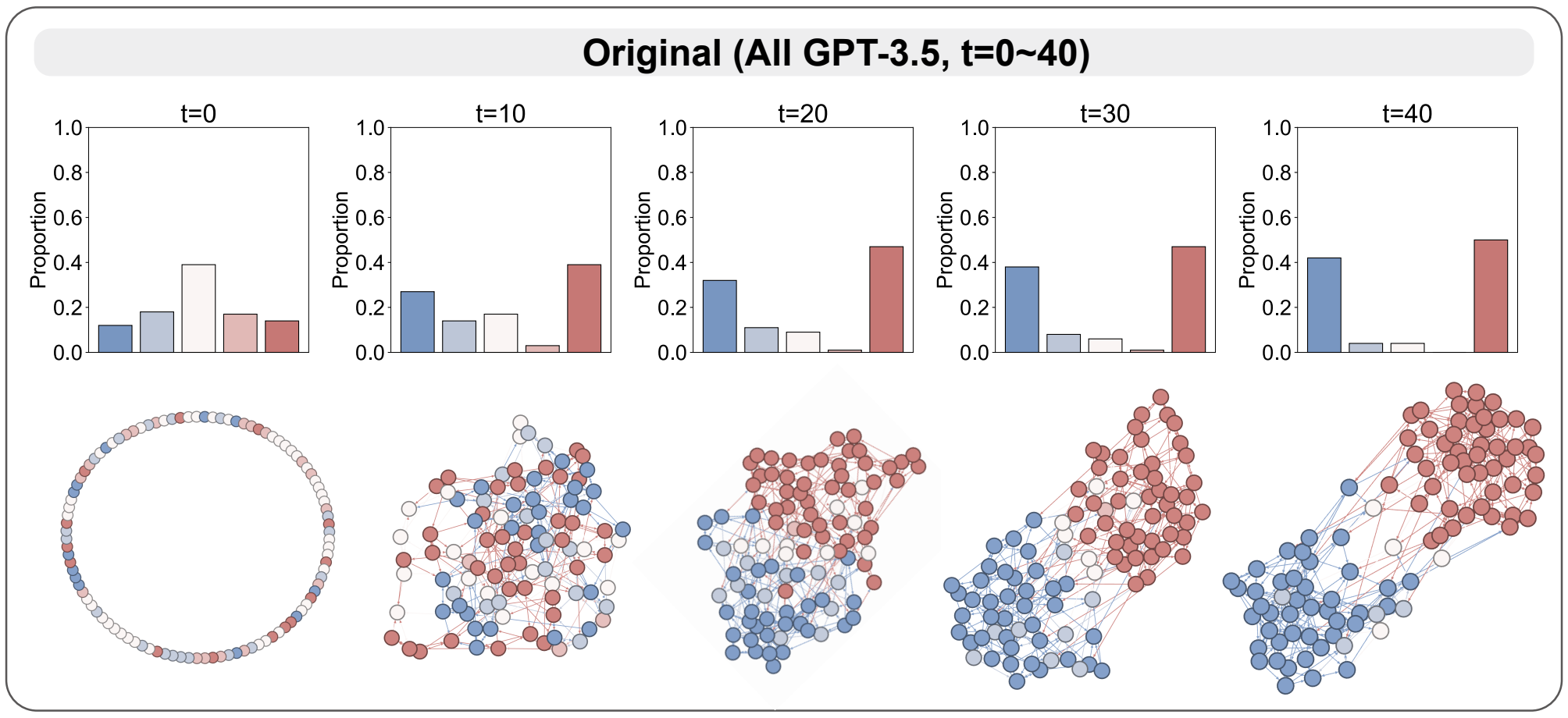

Figure S19. **Evolution of LLM agents' collective opinions and network structures**, where all agents are driven by GPT-3.5 for the entire duration of the simulation and they discuss the political issue of abortion ban.

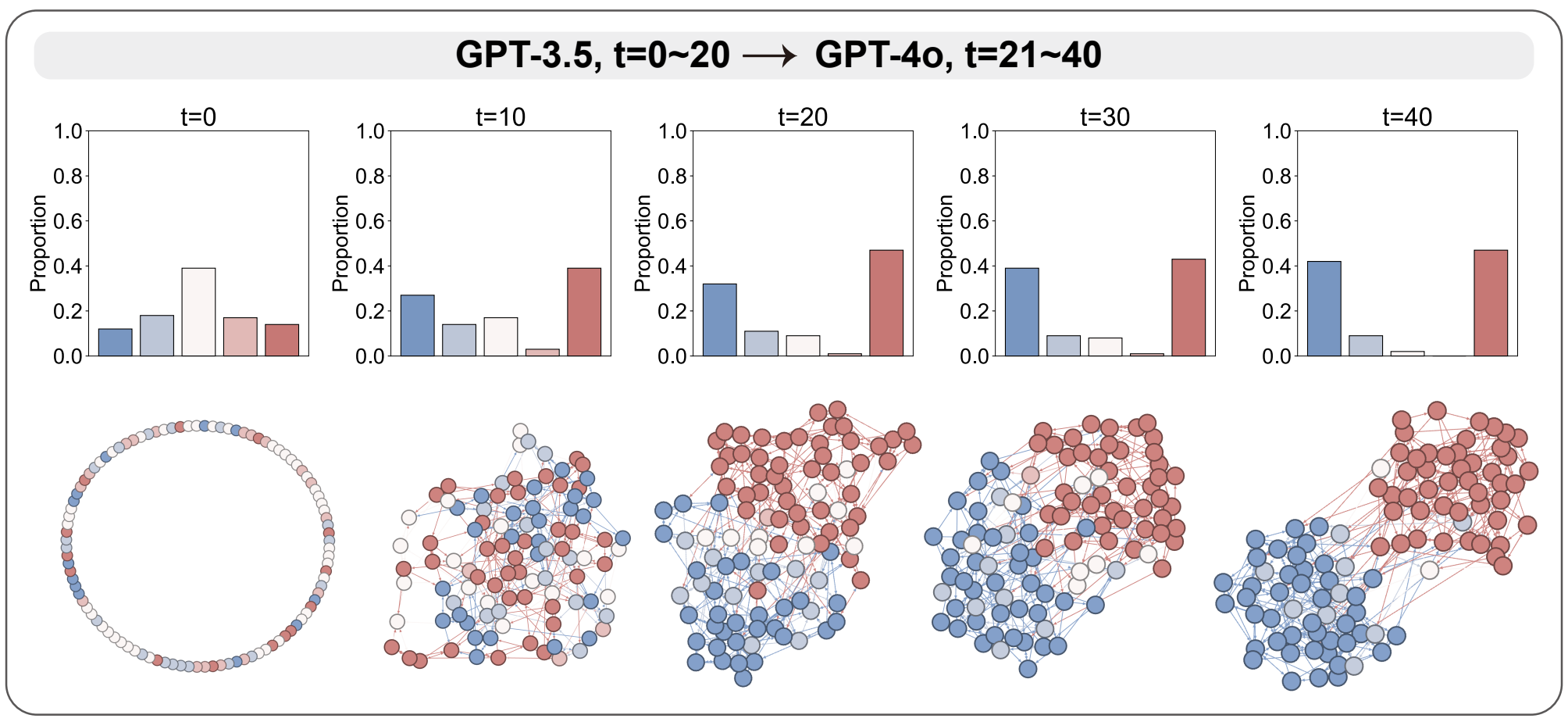

Figure S20. **Evolution of LLM agents' collective opinions and network structures**, where agents are initially driven by GPT-3.5 but defect to GPT-4o amid conversations about the political issue of abortion bans.

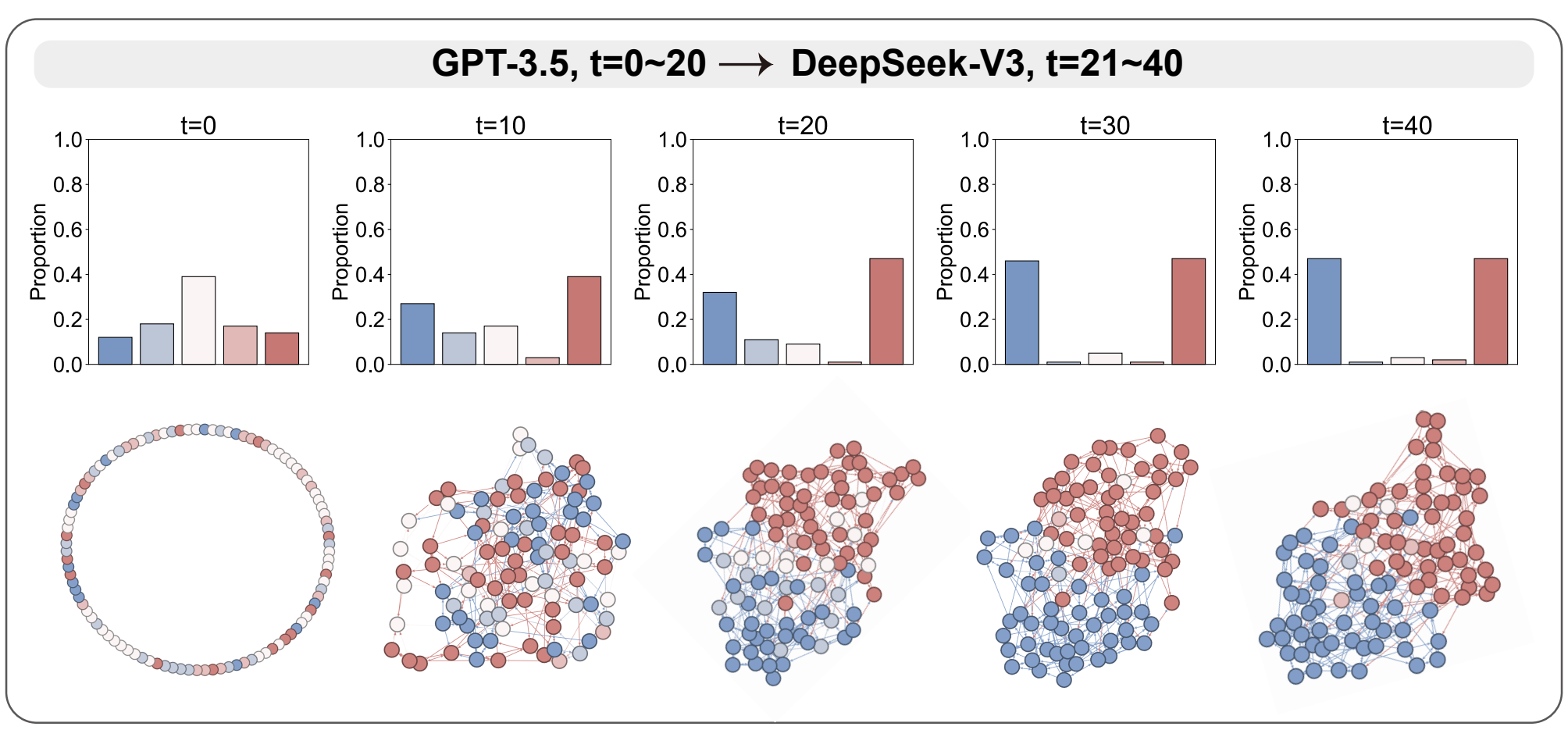

Figure S21. **Evolution of LLM agents' collective opinions and network structures**, where agents are initially driven by GPT-3.5 but defect to DeepSeek-V3 amid conversations about the political issue of abortion bans.

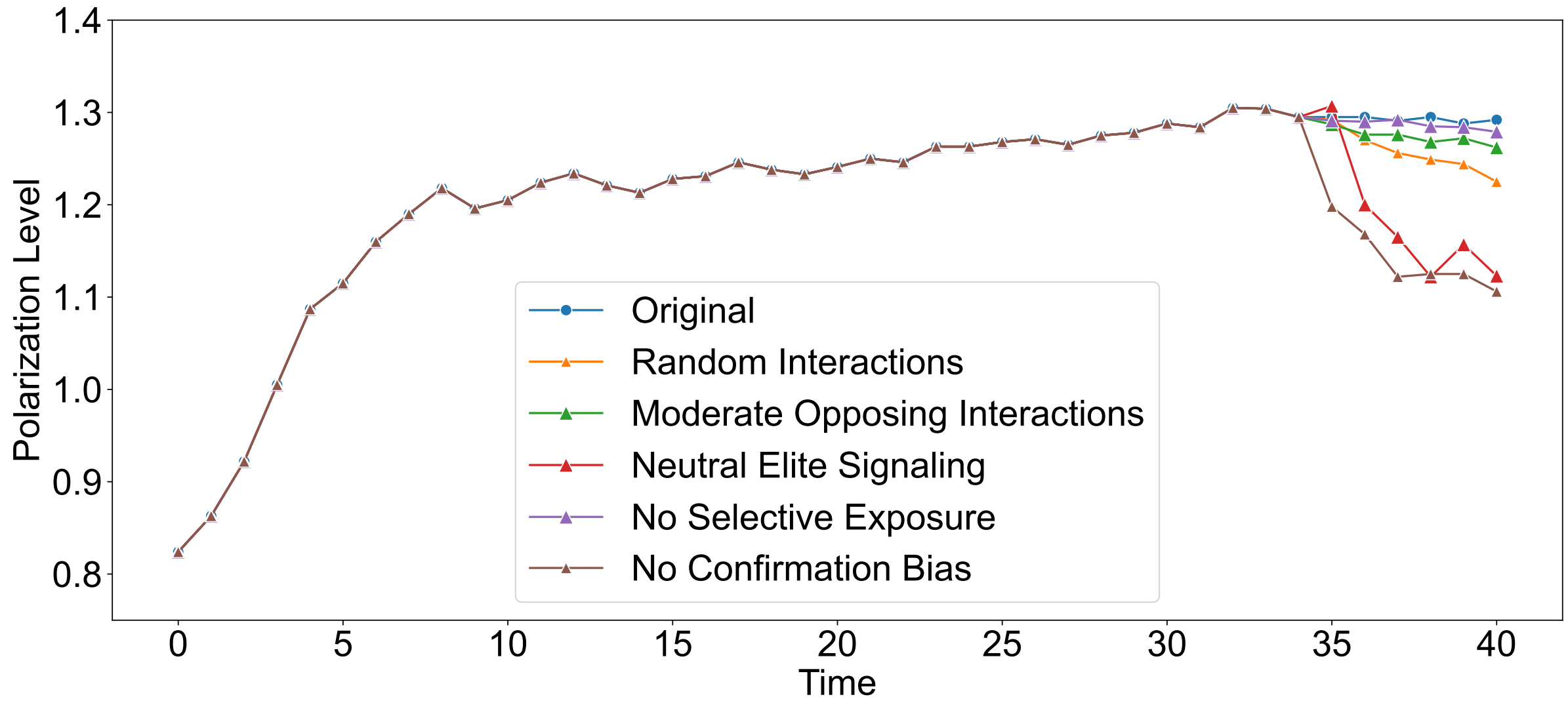

Figure S22. Trends in polarization levels of networked systems under different intervention strategies.

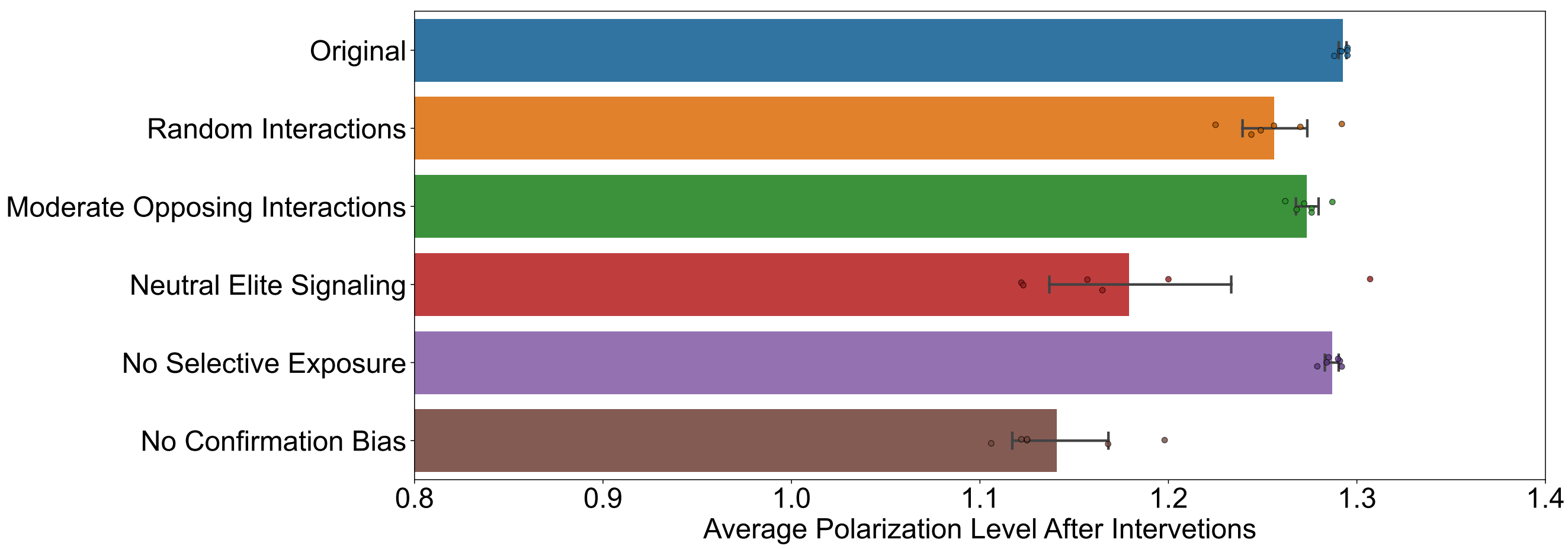

Figure S23. Effectiveness of different intervention strategies in reducing polarization. Bars show the average polarization level across the six post-intervention timesteps ($n = 6$), with the polarization level computed from all agents' opinions at each timestep. Error bars indicate the corresponding 95% CIs.

> Assume you are someone who cares about [issue name].
> Towards [issue name], you support [agent i's opinion]
> Your thought is: [agent i's supporting message].
> There is another person who [agent j's opinion].
> That person's thought is: [agent j's supporting message].
> Please rate your impression of that person from 1 to 5, and think of 5 adjectives to describe that person.
> 1 means you have a very negative impression of that person.
> 2 means you have a negative impression of that person.
> 3 means you have a neutral impression of that person.
> 4 means you have a positive impression of that person.
> 5 means you have a very positive impression of that person.
> Respond in JSON format, with keys 'rating' and 'adjectives'. Rating is an integer from 1 to 5, and adjectives are a list of 5 vocabularies.

Specifically, we allow each agent i to rate and describe three randomly selected agents: one with similar opinions from the same camp, one with opposing opinions from a different camp, and one with neutral opinions. We evaluate agents' perceptions

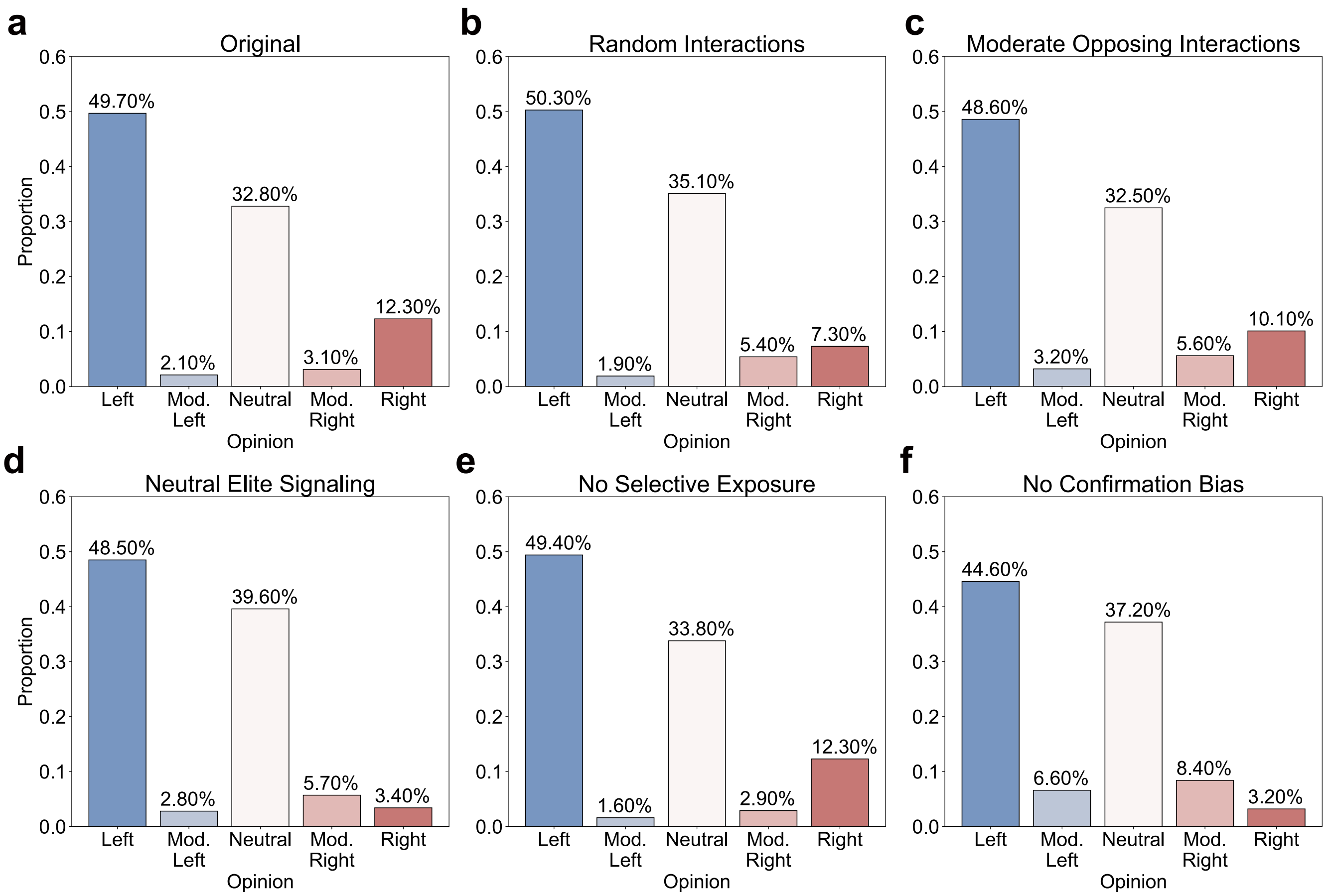

Figure S24. Opinion distributions of networked systems under different intervention strategies: **a**, original; **b**, random interactions; **c**, moderate opposing interactions; **d**, neutral elite signaling; **e**, no selective exposure; **f**, no confirmation bias.

of others every five timesteps during the evolution of self-regulated networked systems (Figure 2d in Main Text). As shown in Figures S29-S33, we observe that agents are likely to have a better impression of those who share similar opinions from the same camp. By contrast, agents hold the lowest impressions of those in the opposing camp. Moreover, we delve deeper into agents' perceptions by examining both the content and sentiment of their descriptions in a more fine-grained manner. Figures S36-S38 show the distributions of descriptions across positive, neutral, and negative sentiments. Overall, we find that in most cases, agents tend to adopt positive descriptions when referring to other agents. However, when encountering agents with opposing opinions, they tend to express some negative descriptions. For example, as shown in Figures S39-S41, they use adjectives like "conflicting", "rigid", and "opinionated" to describe opposing agents.

After analyzing agents' social perceptions, we next examine the psychological processes underlying their opinion updates. To clarify, the proposed system does not explicitly assume or hard-code specific mechanisms (e.g., confirmation bias or motivated reasoning) at this stage. Instead, the only assumption is that LLM agents update their opinions based on their current state and the messages received from their socially connected peers. The psychological processes emerge naturally as observable patterns in the outputs produced by the underlying LLMs in response to social inputs.

That said, by analyzing how agents update their opinions, we can characterize behavioural patterns, in a manner analogous to how social scientists study human participants. In line with this, we analyze the self-reported reasons generated by agents during opinion updates, with results summarized in Table S3. The most frequently observed patterns are consistent with confirmation bias, reflecting agents' tendency to favor information aligned with their prior beliefs; motivated reasoning, which further strengthens these tendencies through selective interpretation; and moral conviction, which anchors opinions more firmly by framing them as moral standards. Notably, these processes closely mirror well-documented mechanisms in human social psychology, further supporting the plausibility of the agents' emergent behaviours.

To scrutinize the connection between the polarization dynamics observed in our synthetic LLM-agent testbed and the established argument-accumulation lineage in opinion-dynamics research [28, 15], we analyze how agents' self-reported supporting reasons evolve as their opinions become more polarized. Specifically, we compare the supporting reasons generated

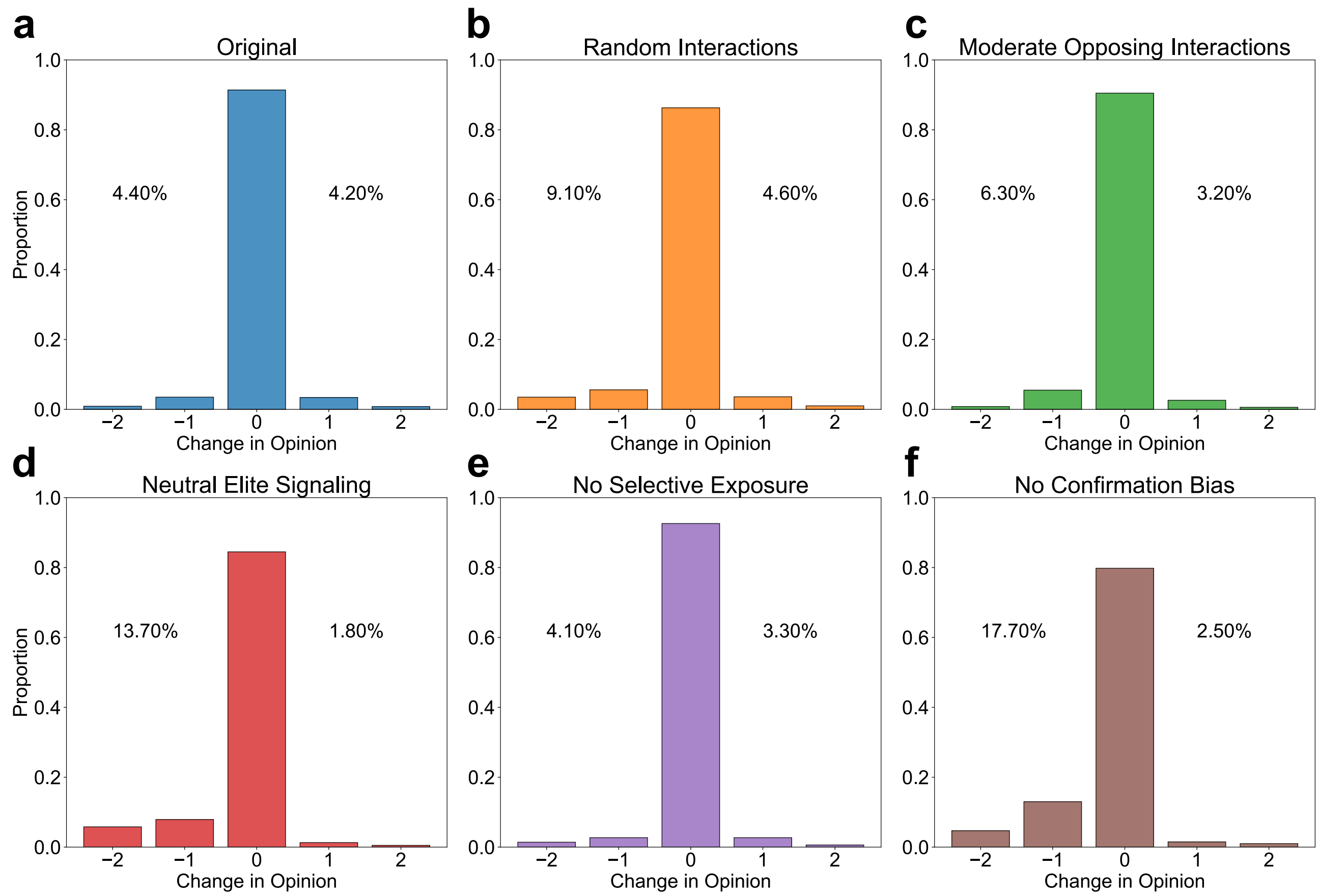

Figure S25. Probability of opinion change under different intervention strategies: **a**, original; **b**, random interactions; **c**, moderate opposing interactions; **d**, neutral elite signaling; **e**, no selective exposure; **f**, no confirmation bias.

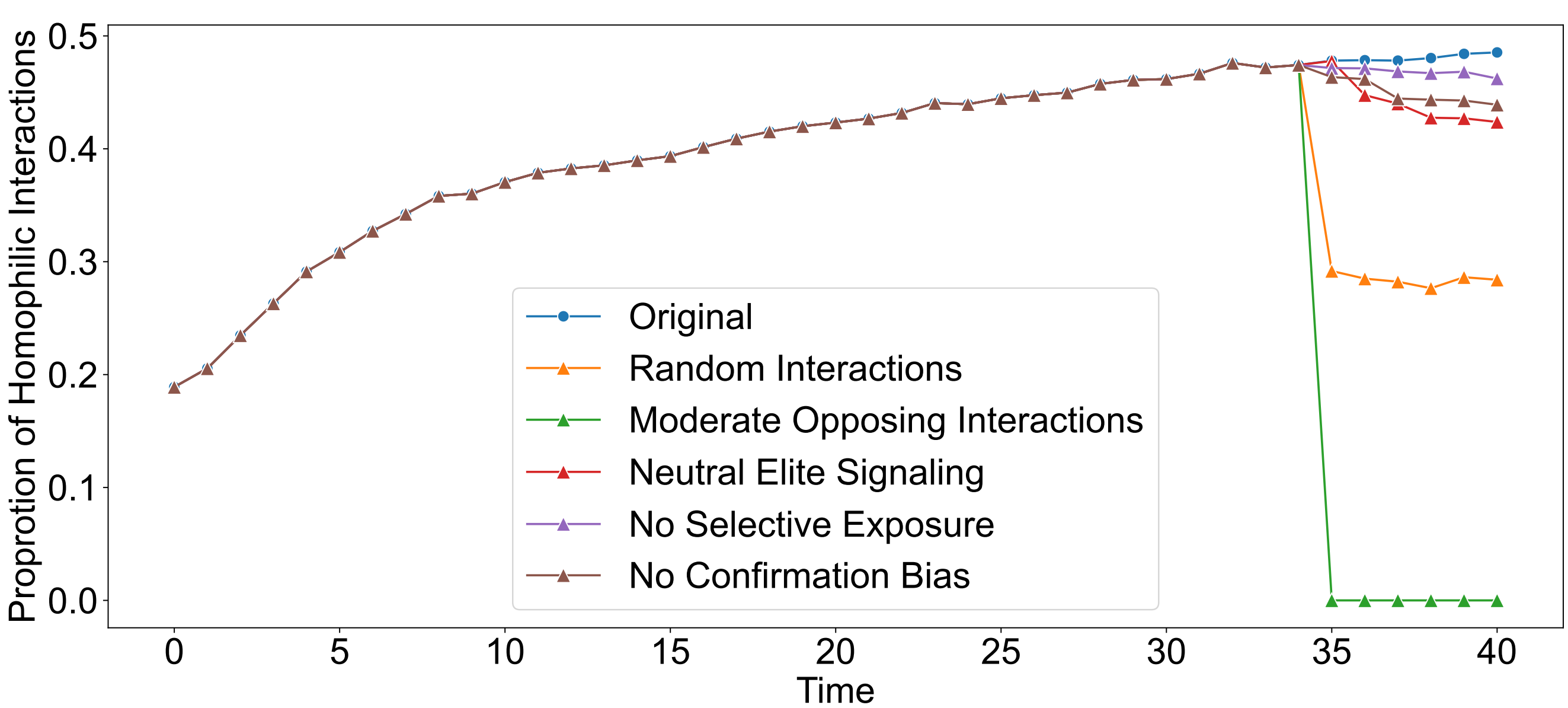

Figure S26. Trends in homophilic interactions of networked systems under different intervention strategies.

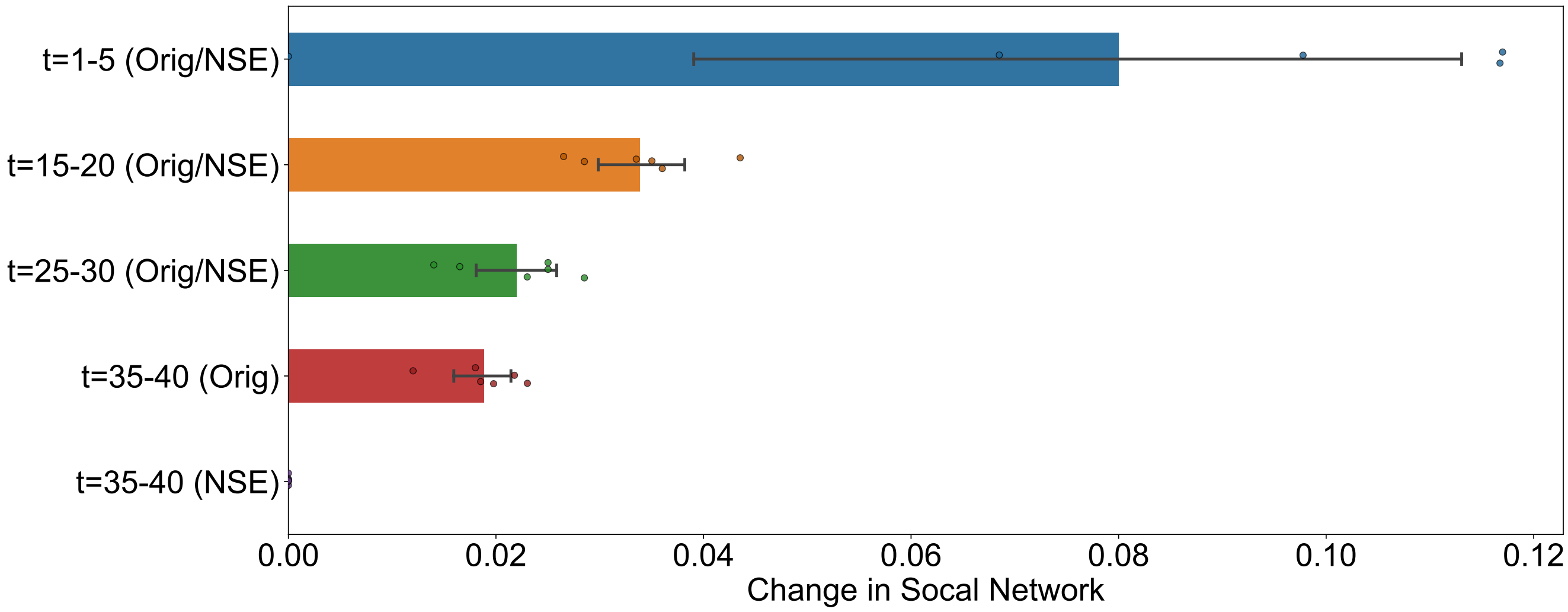

Figure S27. Comparison of changes in LLM agents' social network, where we measure the difference between the current social network and that in the former timestep. Here the bars represent the average values ($n = 5$ or 6, corresponding to four or five timestep-level values computed from all agent interactions at each timestep) and the error bars represent the corresponding 95% CIs.

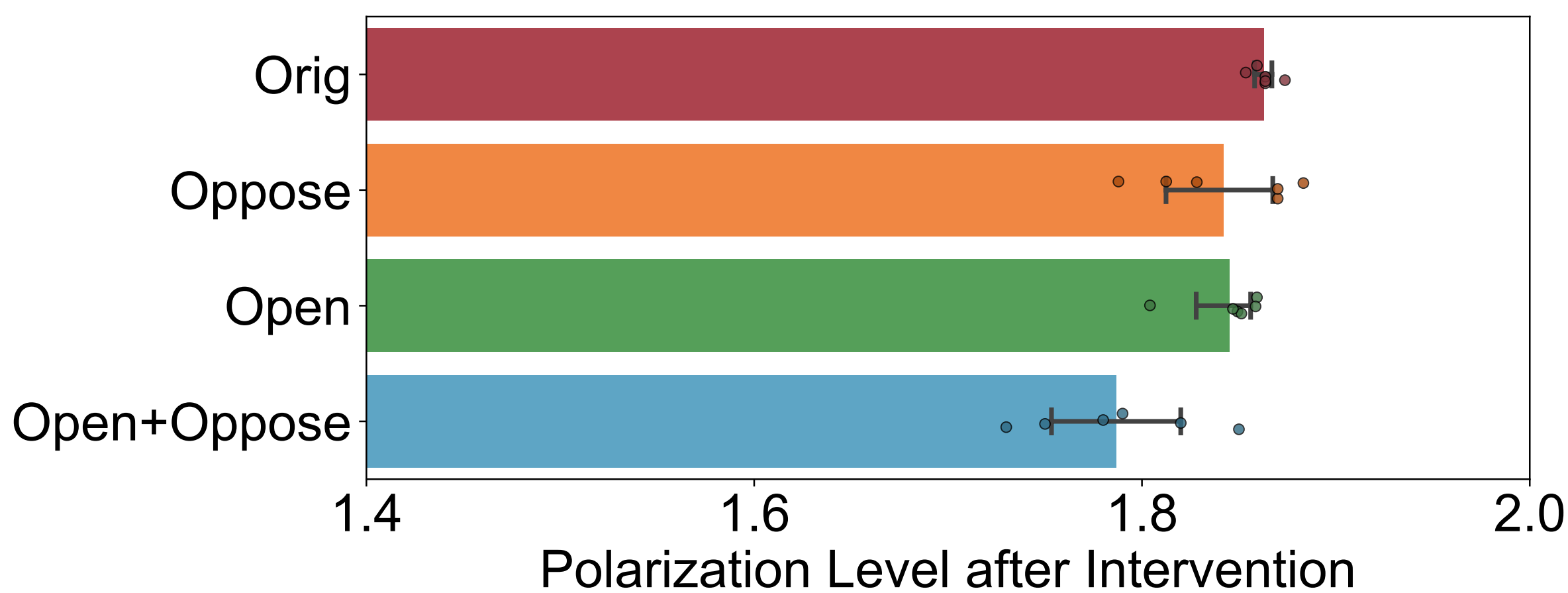

Figure S28. Comparison of open-mindedness intervention strategies, where we intervene in systems at t=35 and agents discuss the issue of abortion ban. Here bars show the average values ($n = 6$, corresponding to six timestep-level values computed from all agents' opinions) and error bars represent the corresponding 95% CIs.

by agents before and after adopting more polarized opinions. We use a LLM to extract distinct arguments contained in these self-reported reasons. As shown in Figure S35a, agents who become more polarized tend to accumulate a significantly larger number of supporting arguments over time across all three topics. Figure S35b–d further report the most frequently occurring argument themes for each topic after polarization. The distribution of popular arguments varies across topics, reflecting issue-specific reasoning patterns. Overall, these findings provide additional evidence that the results in the synthetic system are consistent with argument-accumulation models.

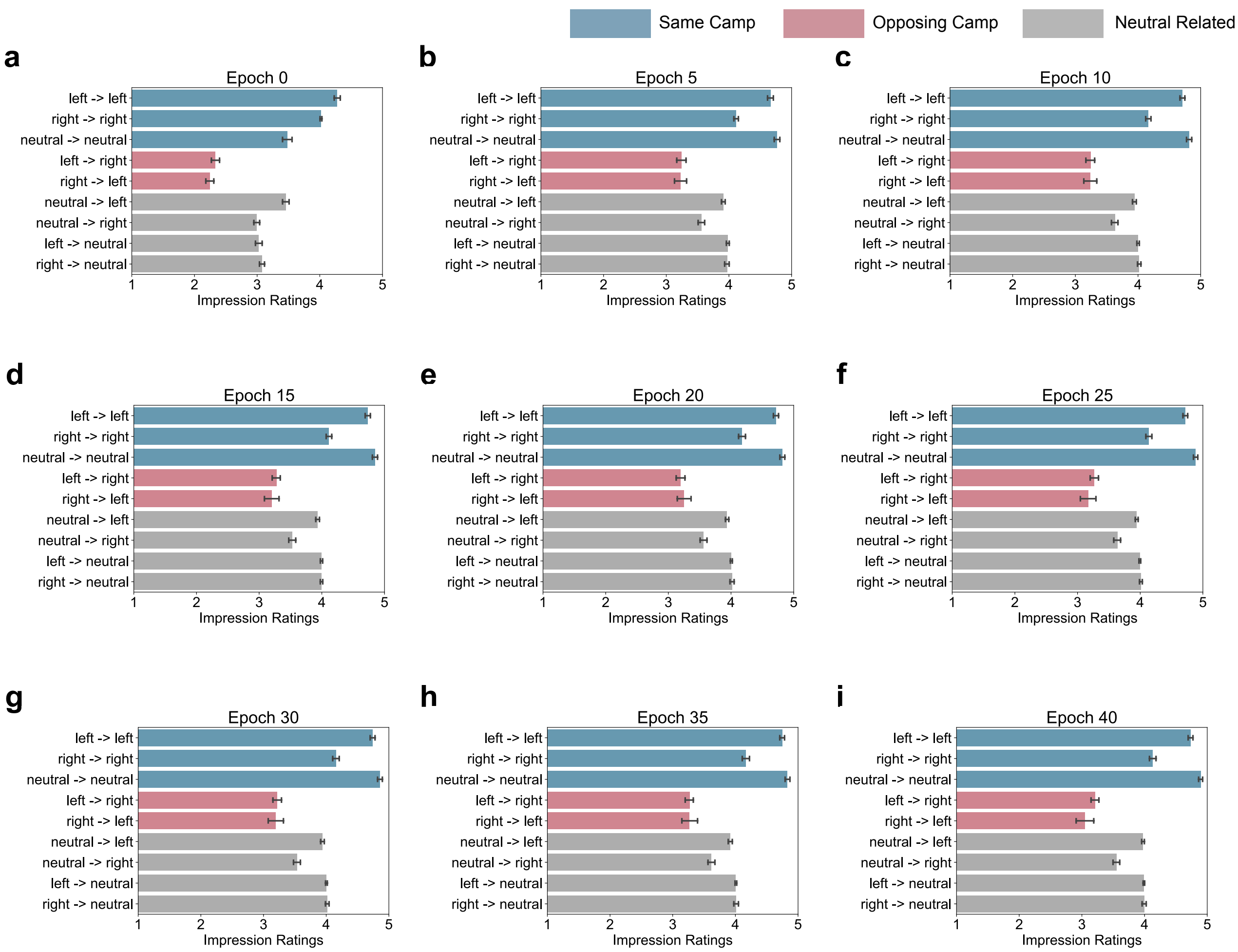

Figure S29. Impression ratings when agents discuss the issue of partisan alignment in the networked system. Bars represent mean values, and error bars represent the corresponding 95% CIs. Each plot includes ratings from 1,000 agents, each evaluating three randomly selected agents: one with similar opinions from the same camp, one with opposing opinions from a different camp, and one with neutral opinions, yielding a total sample size of $n = 3,000$. The corresponding distributions of these ratings are shown in the violin plots below.

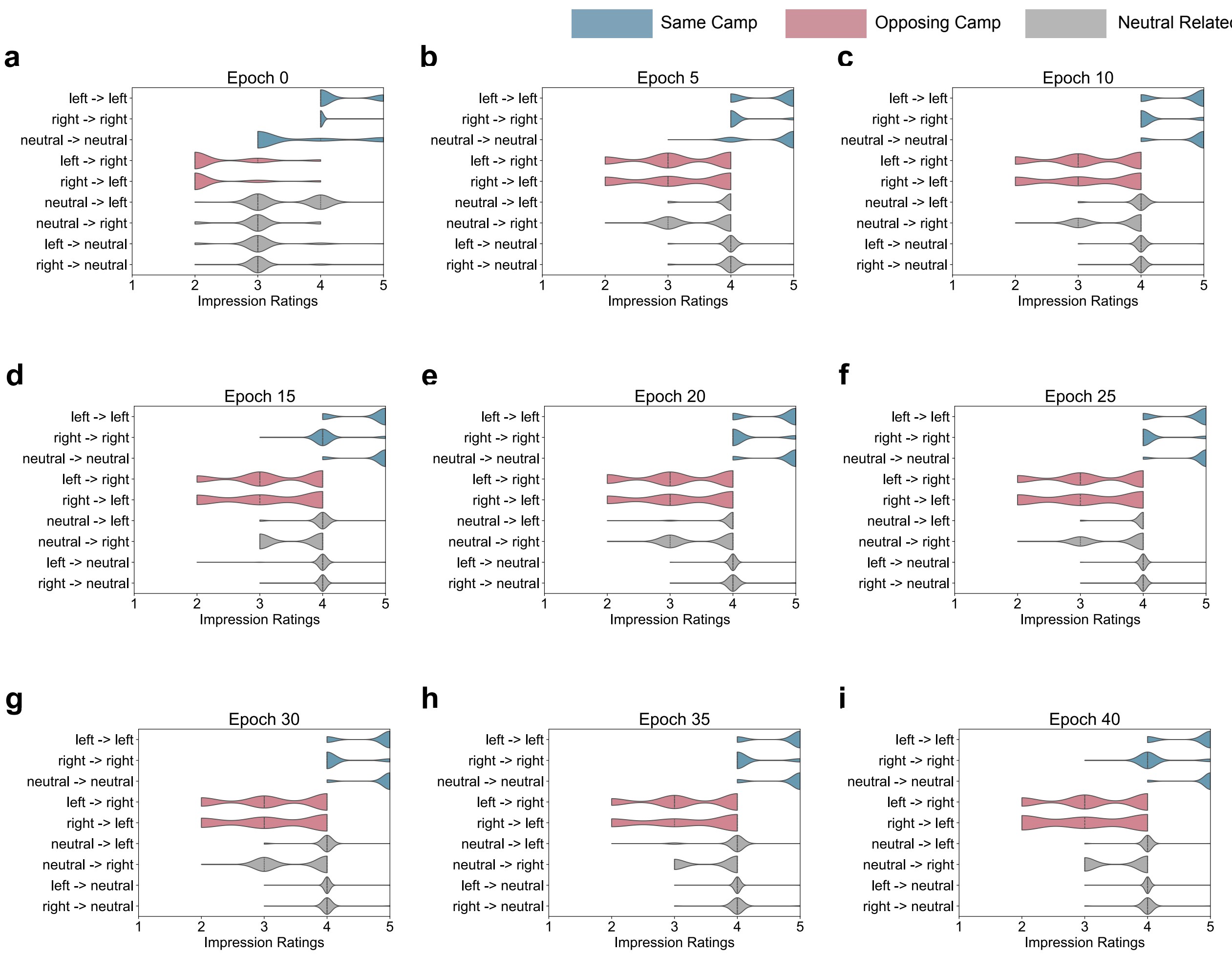

Figure S30. Distributions of impression ratings when agents discuss the issue of partisan alignment in the networked system. Violin plots show the distributions of the same ratings summarized in Fig. S29.

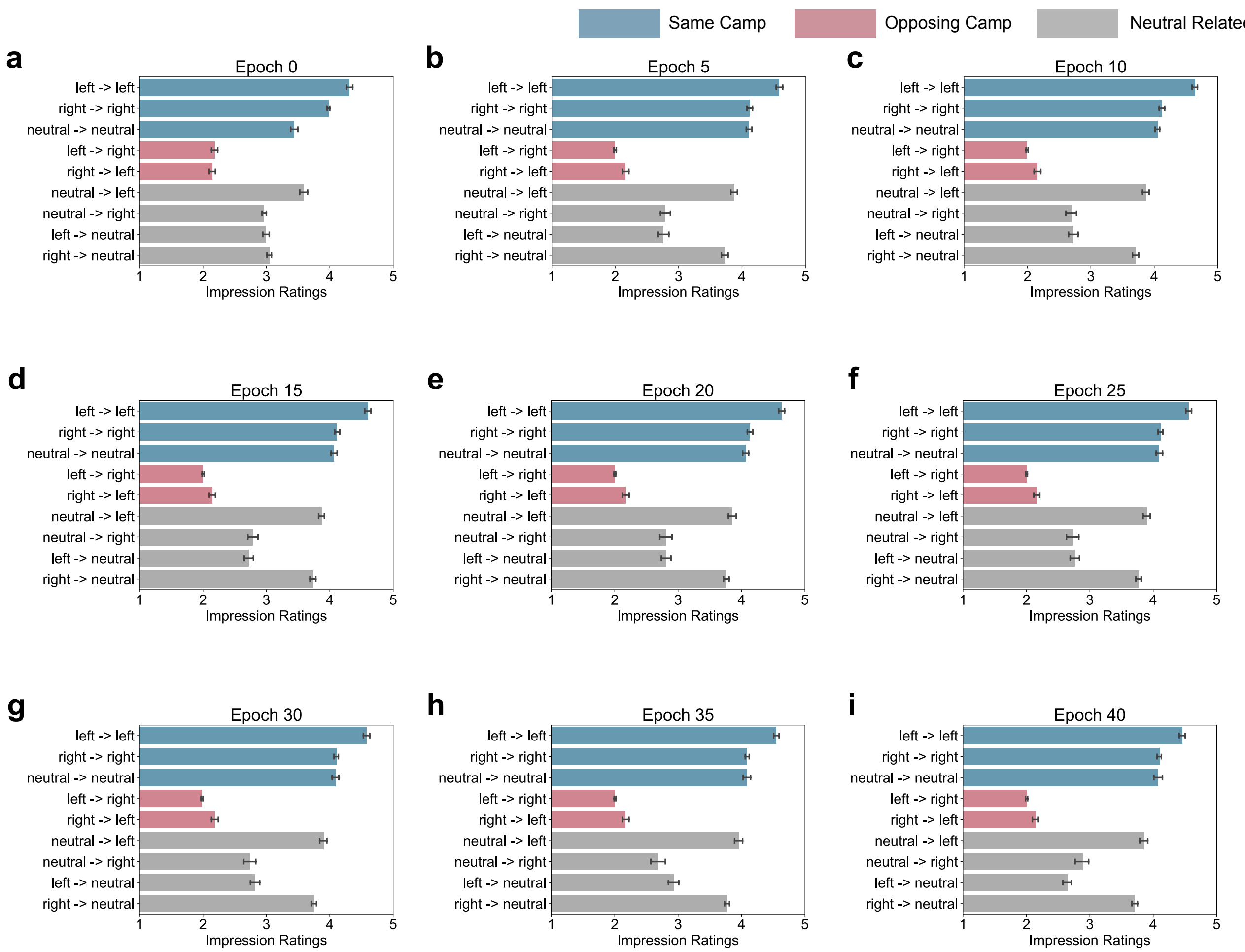

Figure S31. Impression ratings when agents discuss the issue of abortion ban in the networked system. Bars represent mean values, and error bars represent the corresponding 95% CIs. Each plot includes ratings from 1,000 agents, each evaluating three randomly selected agents: one with similar opinions from the same camp, one with opposing opinions from a different camp, and one with neutral opinions, yielding a total sample size of $n = 3,000$. The corresponding distributions of these ratings are shown in the violin plots below.

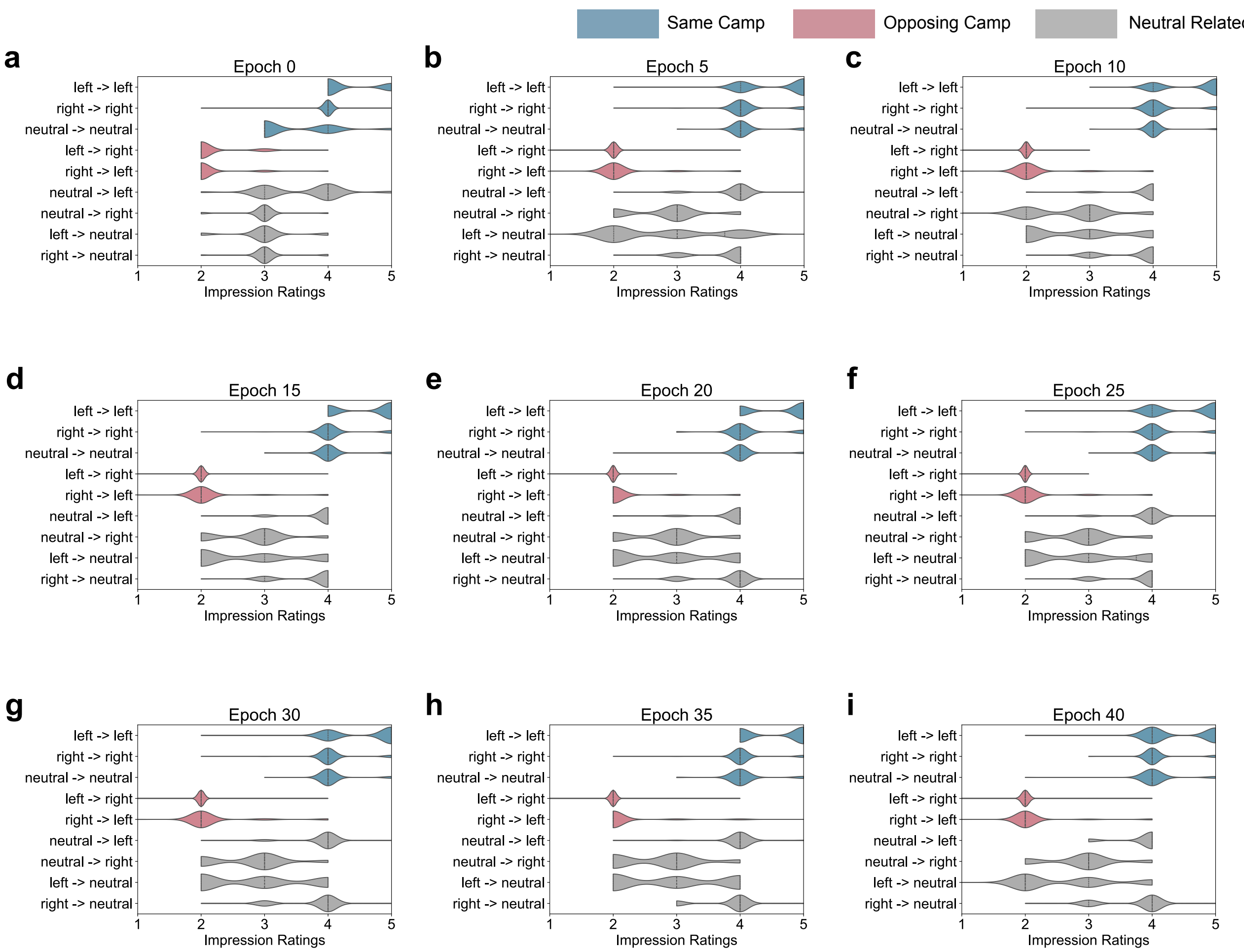

Figure S32. Distributions of impression ratings when agents discuss the issue of abortion ban in the networked system. Violin plots show the distributions of the same ratings summarized in Fig. S31.

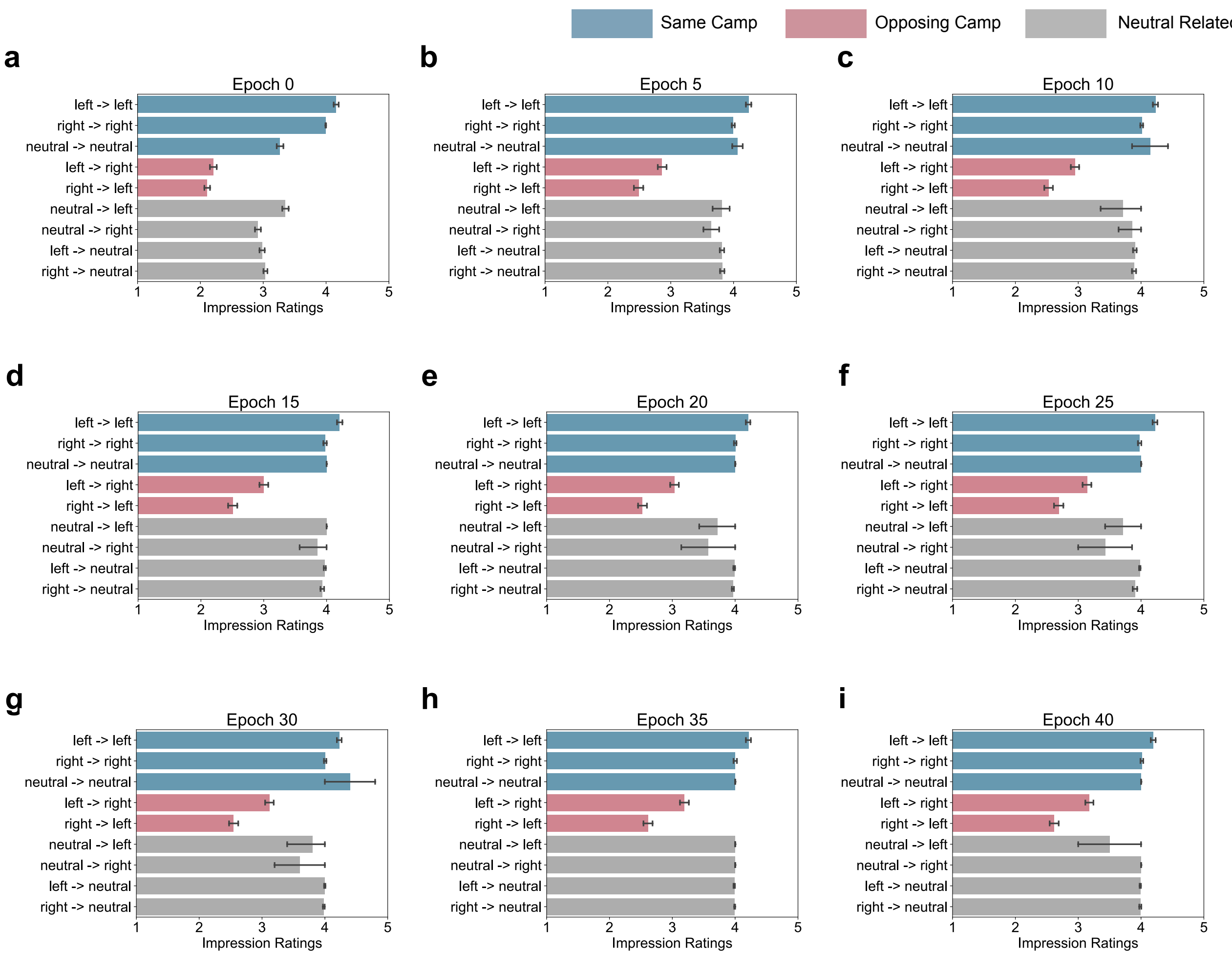

Figure S33. Impression ratings when agents discuss the issue of gun control in the networked system. Bars represent mean values, and error bars represent the corresponding 95% CIs. Each plot includes ratings from 1,000 agents, each evaluating three randomly selected agents: one with similar opinions from the same camp, one with opposing opinions from a different camp, and one with neutral opinions, yielding a total sample size of $n = 3,000$. The corresponding distributions of these ratings are shown in the violin plots below.

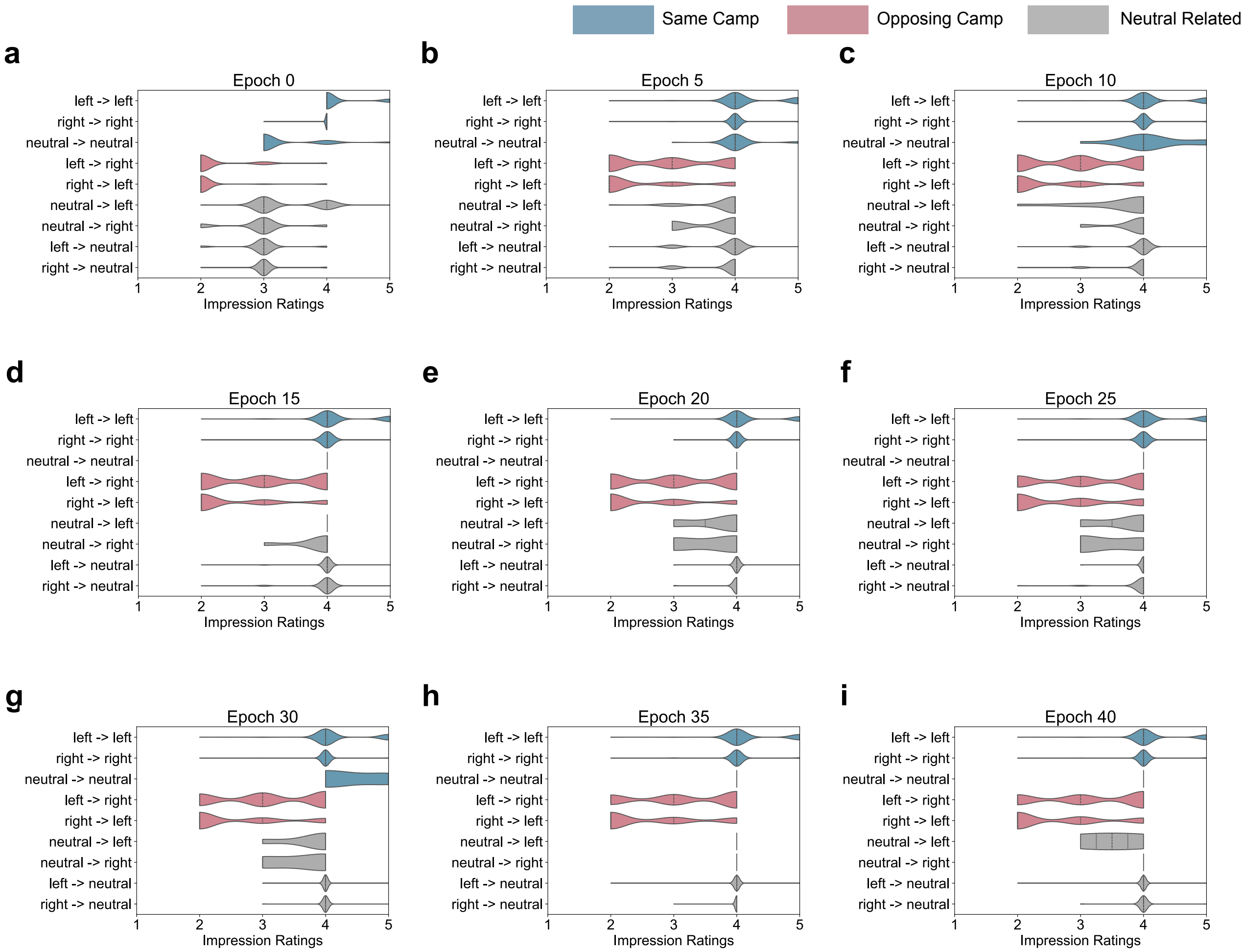

Figure S34. Distributions of impression ratings when agents discuss the issue of gun control in the networked system. Violin plots show the distributions of the same ratings summarized in Fig. S33.

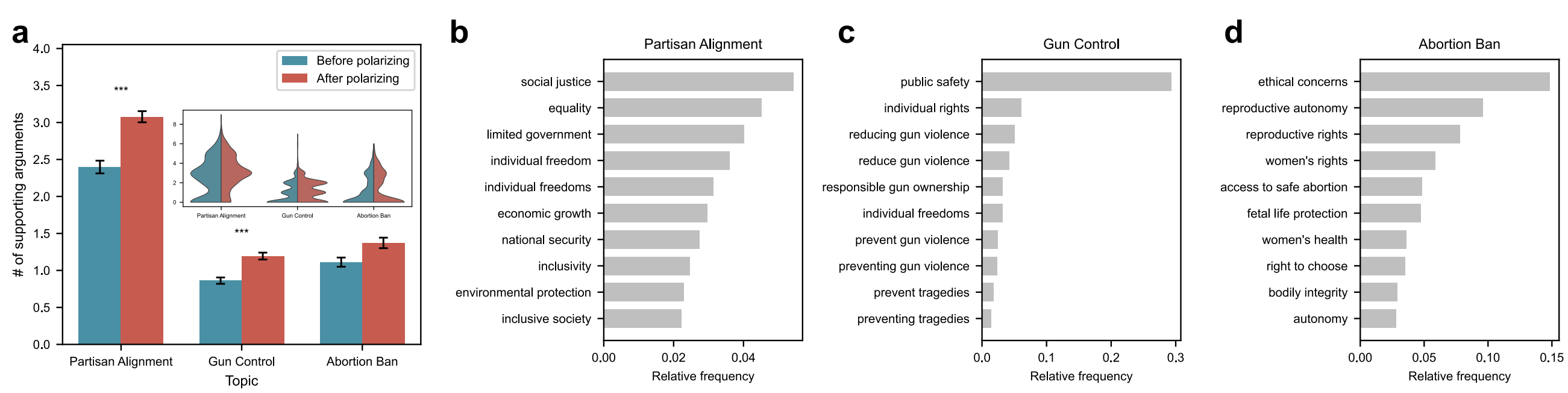

Figure S35. Accumulation of supporting arguments during polarization. (a) Average number of self-reported supporting arguments generated by agents before and after polarization across three issues. Bars represent mean values, and error bars represent the corresponding 95% CIs. Each bar includes responses from 500 randomly sampled agents either before or after they adopted more polarized opinions ($n = 500$). Each response contains zero or more self-reported supporting arguments. (b–d) Relative frequency of argument themes appearing in agents' responses.

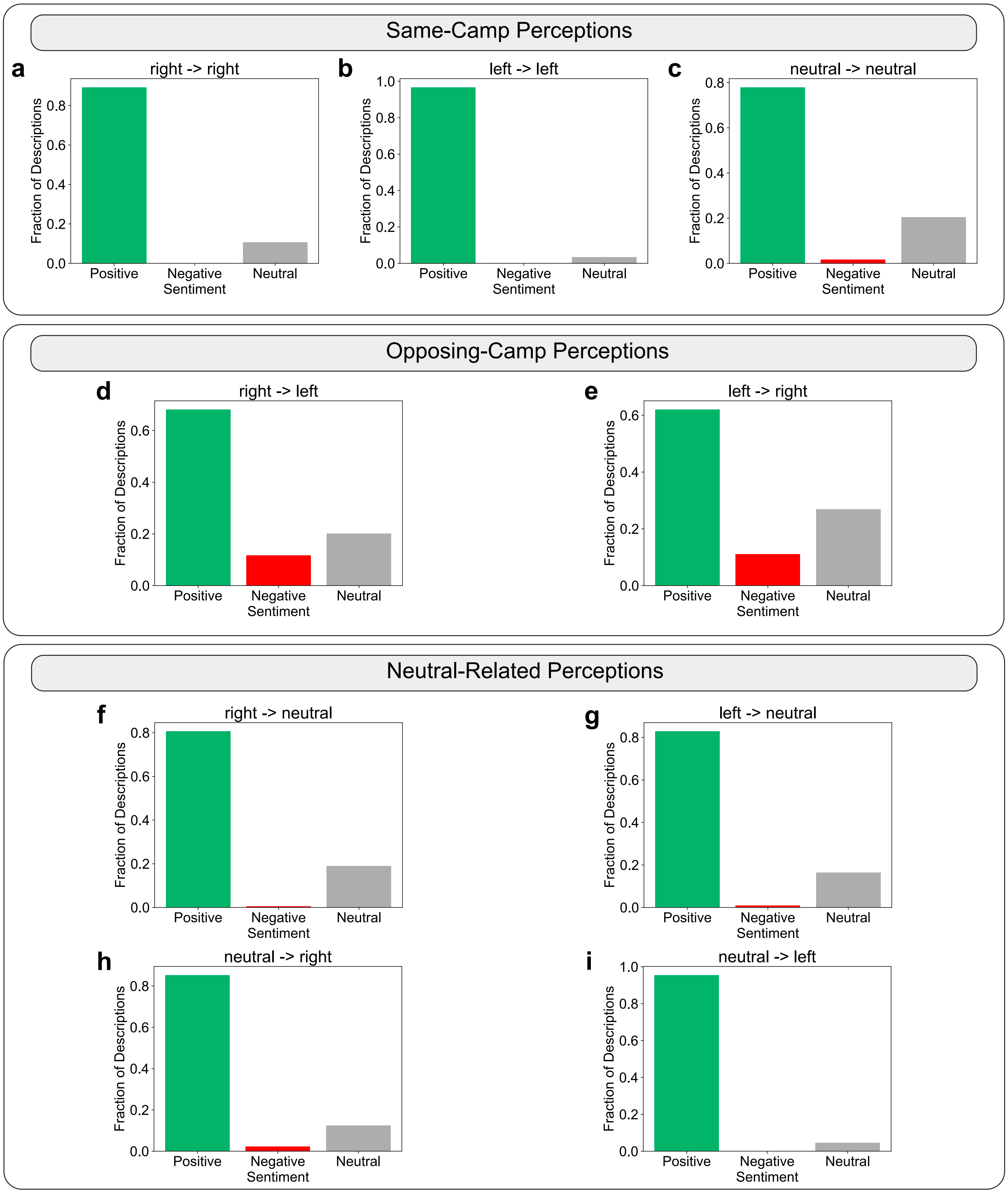

Figure S36. **Distribution of descriptions among different sentiments in the discussion of partisan alignment.**

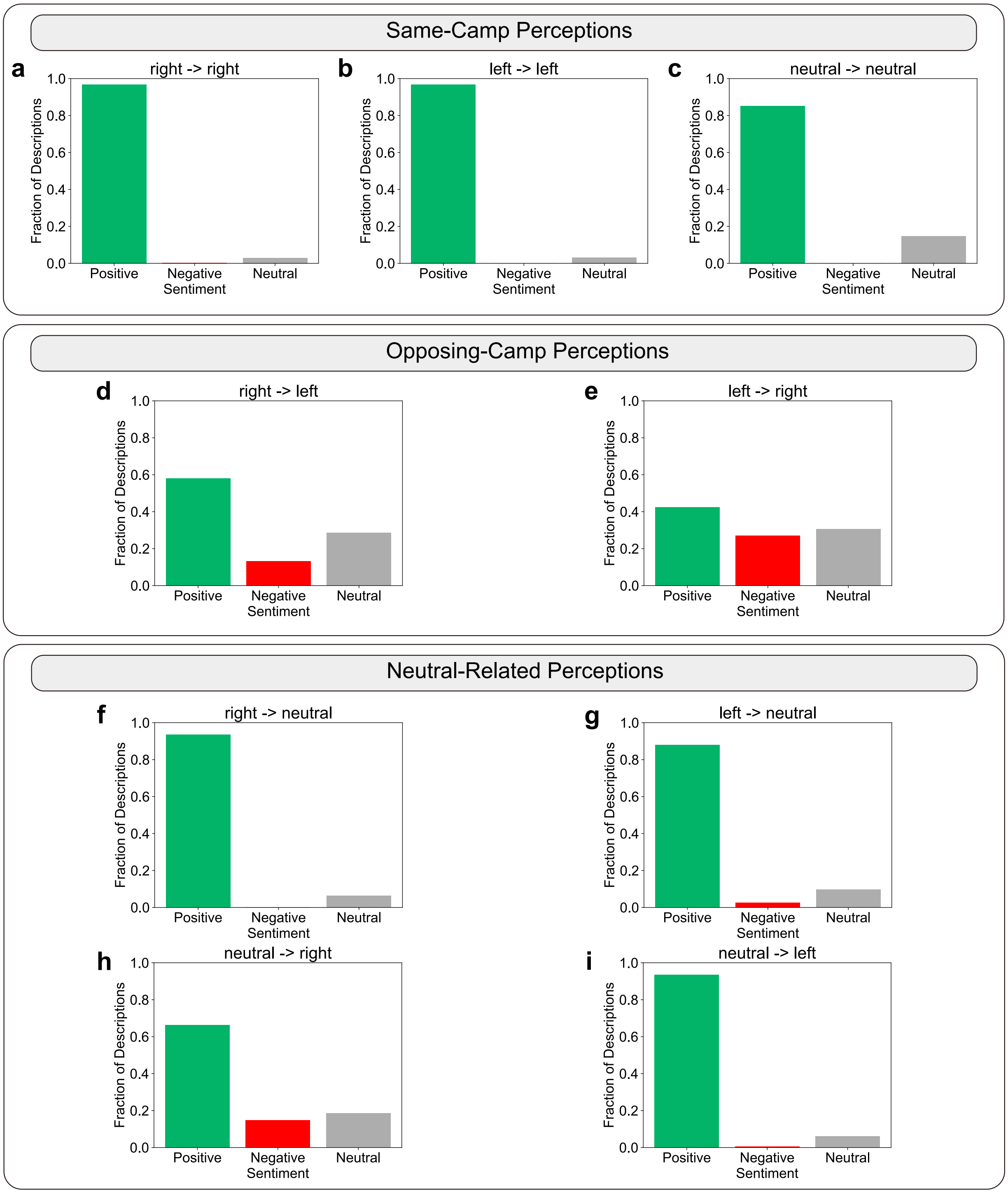

Figure S37. **Distribution of descriptions among different sentiments in the discussion of the abortion ban.**

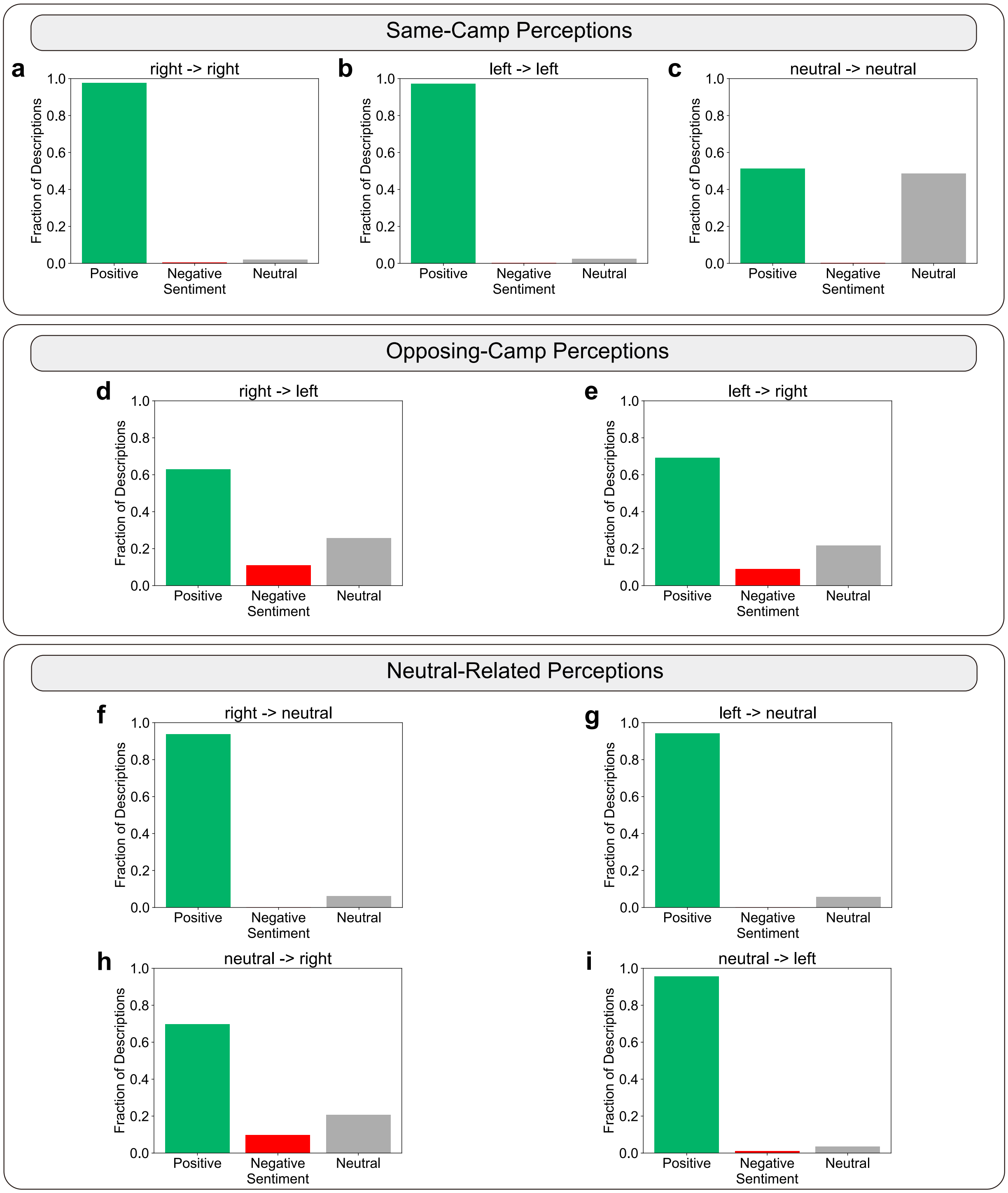

Figure S38. **Distribution of descriptions among different sentiments in the discussion of gun control.**

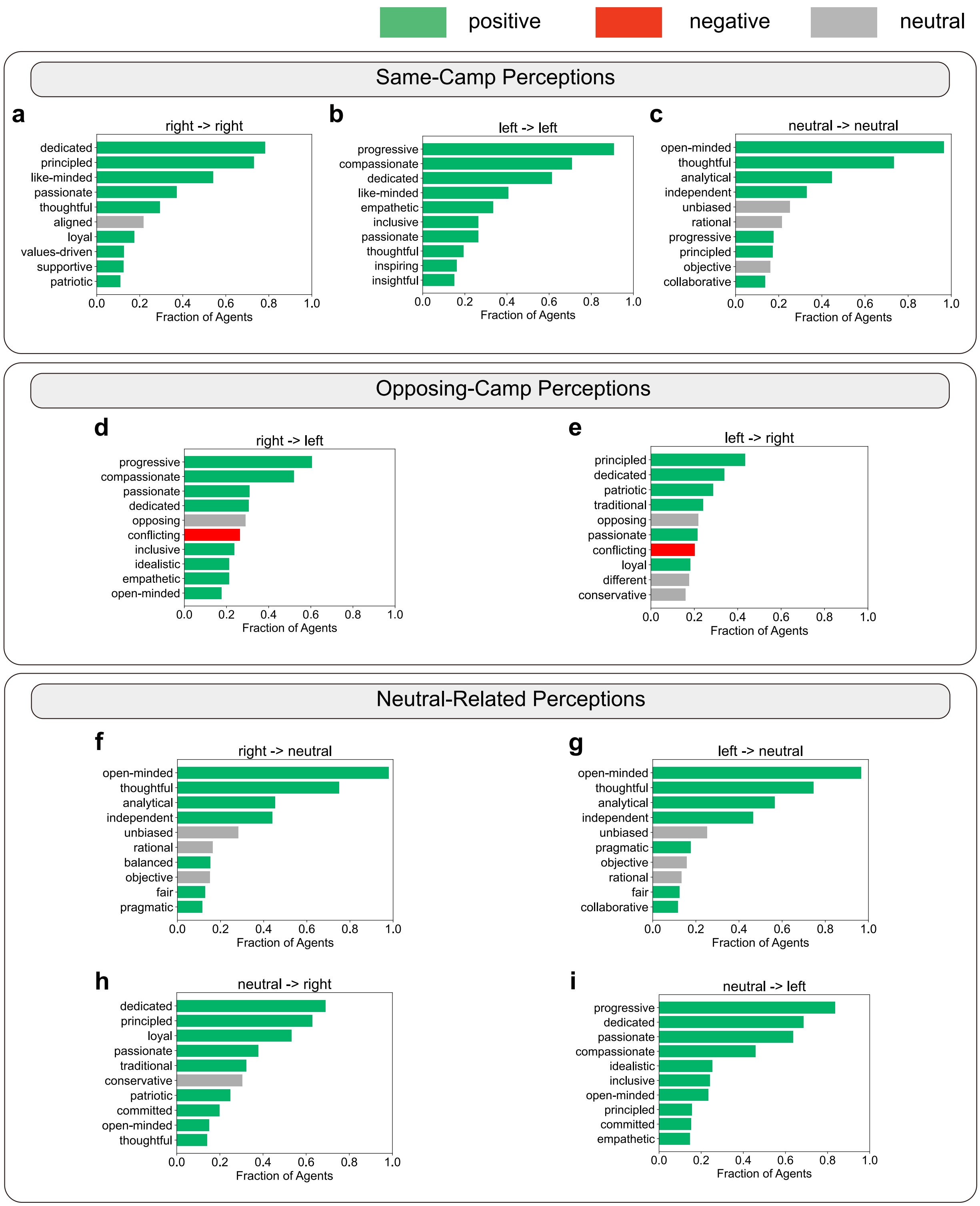

Figure S39. **The 10 most frequently mentioned adjectives in the discussion of partisan alignment.**

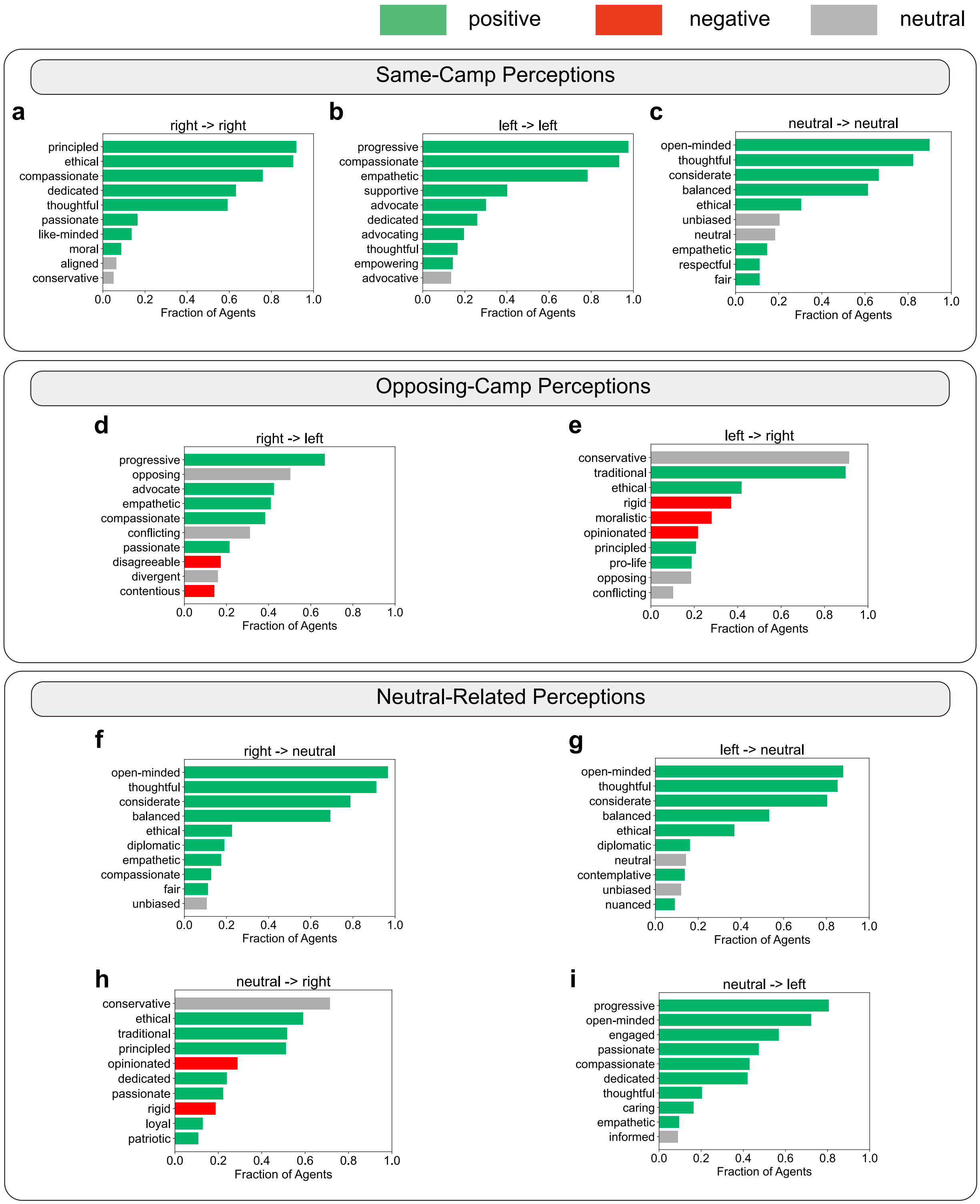

Figure S40. **The 10 most frequently mentioned adjectives in the discussion of the abortion ban.**

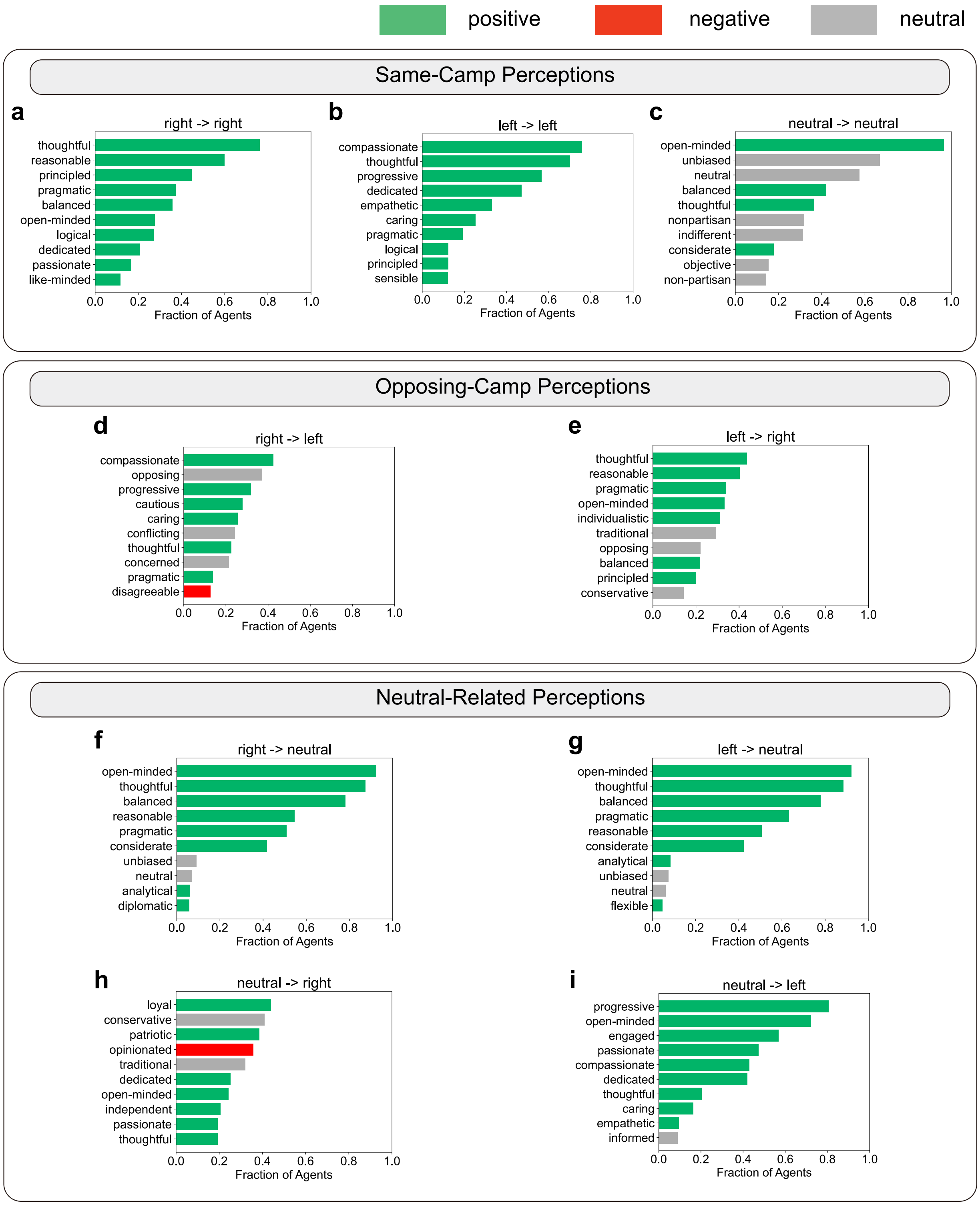

Figure S41. **The 10 most frequently mentioned adjectives in the discussion of gun control.**

Table S3. **Psychological process emerges at the opinion update stage.**

Topic	Opinion Update Direction	Top 3 Psychological Processes and Their Descriptions
**Partisan Alignment**	More Polarized	• Confirmation Bias (97%): Agents selectively focus on information that aligns with their pre-existing beliefs, reinforcing and polarizing their opinions.<br>• Motivated Reasoning (57%): Agents interpret new information in a way that supports their desired conclusions, driven by ideological goals, emotional commitments, or a desire to uphold their political identity.<br>• Value Alignment (26%): Agents update their opinions based on perceived alignment between new information and their core personal values.
	Unchanged	• Confirmation Bias (71%): Agents selectively focus on information that aligns with their pre-existing beliefs and values, reinforcing their current opinions while ignoring contradictory evidence.<br>• Belief Perseverance (44%): Agents maintain their original opinion despite exposure to new information due to strong prior convictions and deeply ingrained beliefs.<br>• Motivated Reasoning (41%): Agents selectively process information to maintain consistency with their pre-existing beliefs, values, or ideological commitments.
	Less Polarized	• Motivated Reasoning (44%): Agents selectively process information to align with their pre-existing beliefs and values, reinforcing their political identity.<br>• Confirmation Bias (29%): Agents selectively focus on information that aligns with their pre-existing beliefs, reinforcing their stance while disregarding opposing views.<br>• Value Alignment (28%): Agents update their opinions based on perceived alignment between new information and their core values or political party principles.
**Abortion Ban**	More Polarized	• Moral Conviction (84%): Opinion updates are driven by strong moral or ethical beliefs, leading to more polarized and resistant stances.<br>• Confirmation Bias (69%): Agents selectively focus on information that aligns with their pre-existing beliefs about abortion, reinforcing its polarized opinion.<br>• Motivated Reasoning (35%): Agents selectively process information to align with their pre-existing beliefs and values, reinforcing polarized opinions.
	Unchanged	• Moral Conviction (70%): Agents' opinions are strongly tied to deeply held moral or ethical principles, making them resistant to change based on new information.<br>• Confirmation Bias (65%): Agents selectively focus on information that aligns with their pre-existing beliefs about reproductive rights, autonomy, or ethical concerns.<br>• Belief Perseverance (60%): Agents maintain their original opinions despite new information due to strong prior beliefs.

Topic	Opinion Update Direction	Top 3 Psychological Processes and Their Descriptions
	Less Polarized	• Cognitive Dissonance Reduction (81%): Agents adjust their opinion to reduce discomfort from conflicting beliefs about women's rights and ethical concerns.<br>• Value-Based Reasoning (32%): Agents update their opinions by weighing and prioritizing core values such as autonomy, reproductive rights, and ethical concerns.<br>• Motivated Reasoning (22%): Agents adjust their opinions based on pre-existing values and motivations, seeking moderation or ethical consistency.
**Gun Control**	More Polarized	• Motivated Reasoning (91%): Agents selectively process information to align with pre-existing beliefs or values, reinforcing a polarized stance that prioritizes safety or individual rights.<br>• Confirmation Bias (90%): Agents selectively focus on information that aligns with their pre-existing beliefs about gun control, reinforcing their polarized stance.<br>• Moral Conviction (16%): Agents' opinions are driven by strong moral values like public safety and preventing harm.
	Unchanged	• Confirmation Bias (90%): Agents selectively focus on information that aligns with their pre-existing beliefs about gun control.<br>• Motivated Reasoning (79%): Agents selectively process new information to align with their pre-existing values and goals.<br>• Belief Perseverance (66%): Agents maintain their original opinions despite exposure to new information.
	Less Polarized	• Cognitive Dissonance Reduction (50%): Agents adjust their opinions to reduce discomfort from conflicting beliefs about individual rights and public safety.<br>• Motivated Reasoning (47%): Agents adjust their opinion based on desired outcomes (e.g., public safety, reducing gun violence).<br>• Social Influence (27%): Agents adjust their opinions toward moderation in response to perceived societal norms.

10 Supplementary Note 9: Experiments on LLMs with Varying Temperatures

To explore whether the temperature setting — which controls the diversity of LLM-generated outputs — affects the emergence of polarization, we conduct additional simulations with lower (0.5) and higher (1.5) temperature values. Figure S42 shows agents' collective opinion dynamics while varying temperatures in their underlying LLMs. We observe that regardless of whether the temperature is lower (Figure S42a) or higher (Figure S42c), these agents spontaneously develop collective opinions through social interaction, leading to the emergence of polarization. Meanwhile, at the network level, temperature changes do not alter the homophily observed in naturalistic LLM social interactions. This indicates that temperature, despite being a key parameter for LLMs, has a limited effect on whether polarization emerges among a collective of interacting agents.

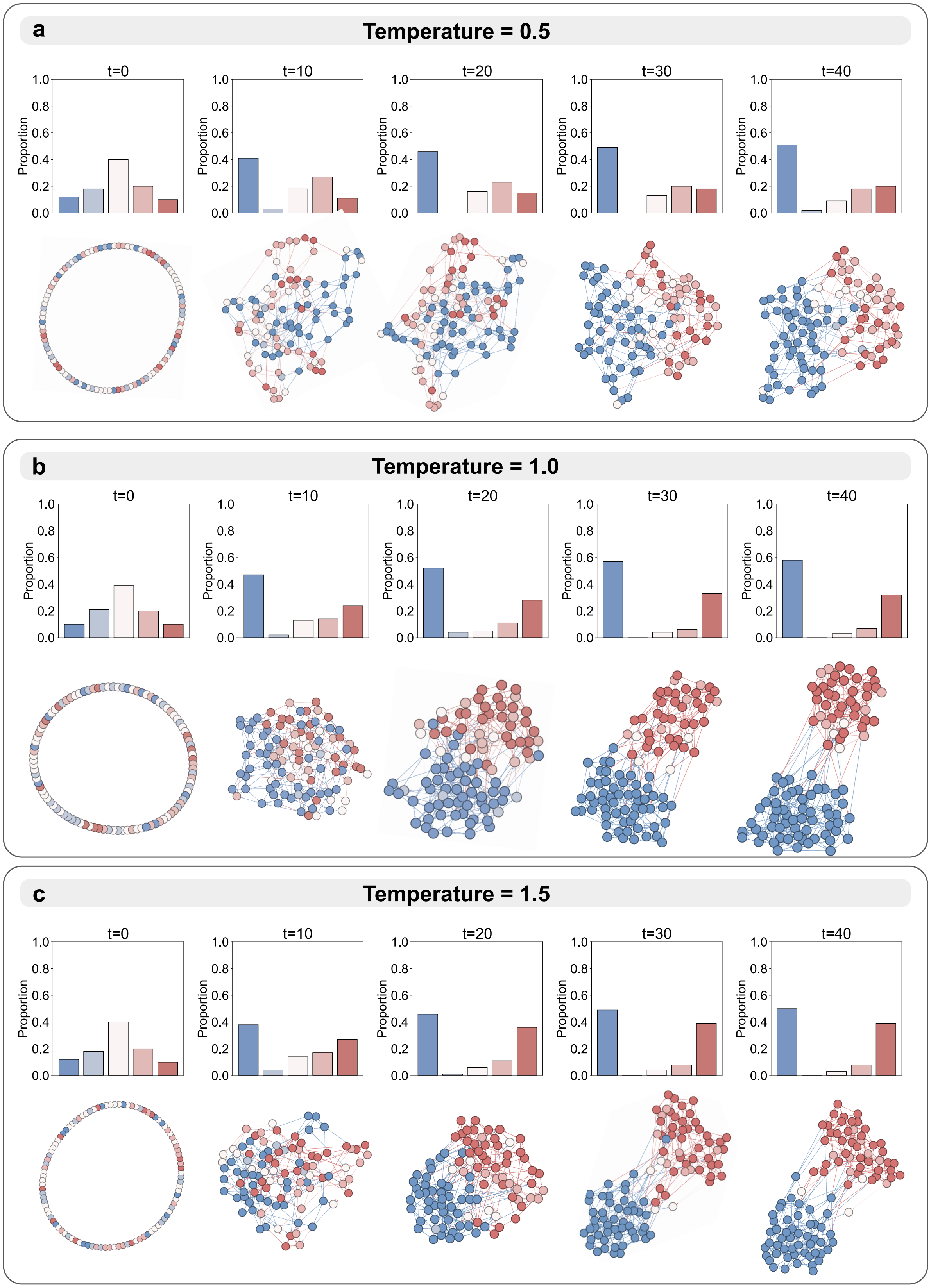

Figure S42. Evolution of LLM agents' collective opinions and network structures, where the underlying temperature is **a,** 0.5, **b,** 1.0, and **c,** 1.5.

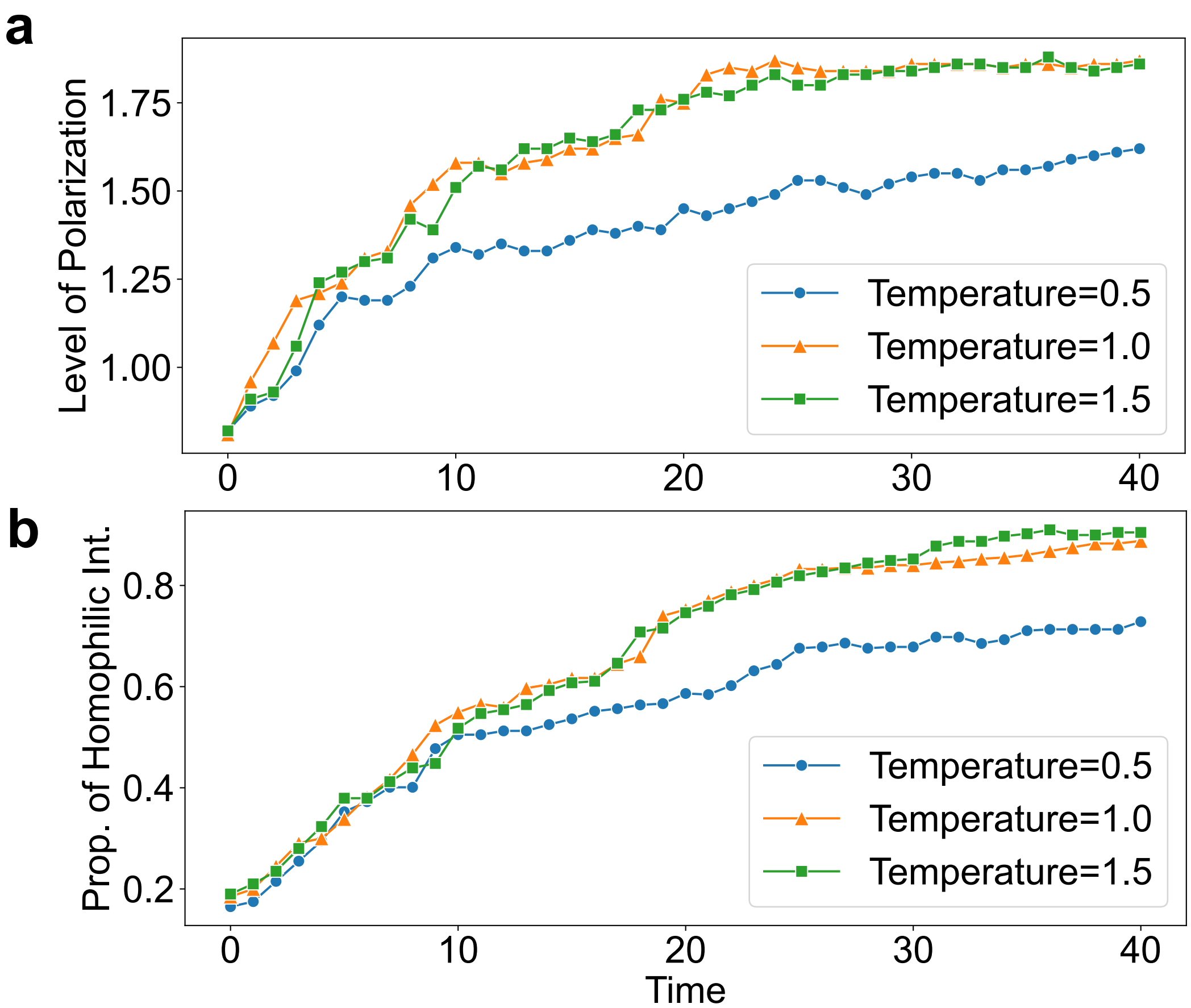

Figure S43. **Changes in a, the level of polarization, and b, the proportion of homophilic interactions over time.**

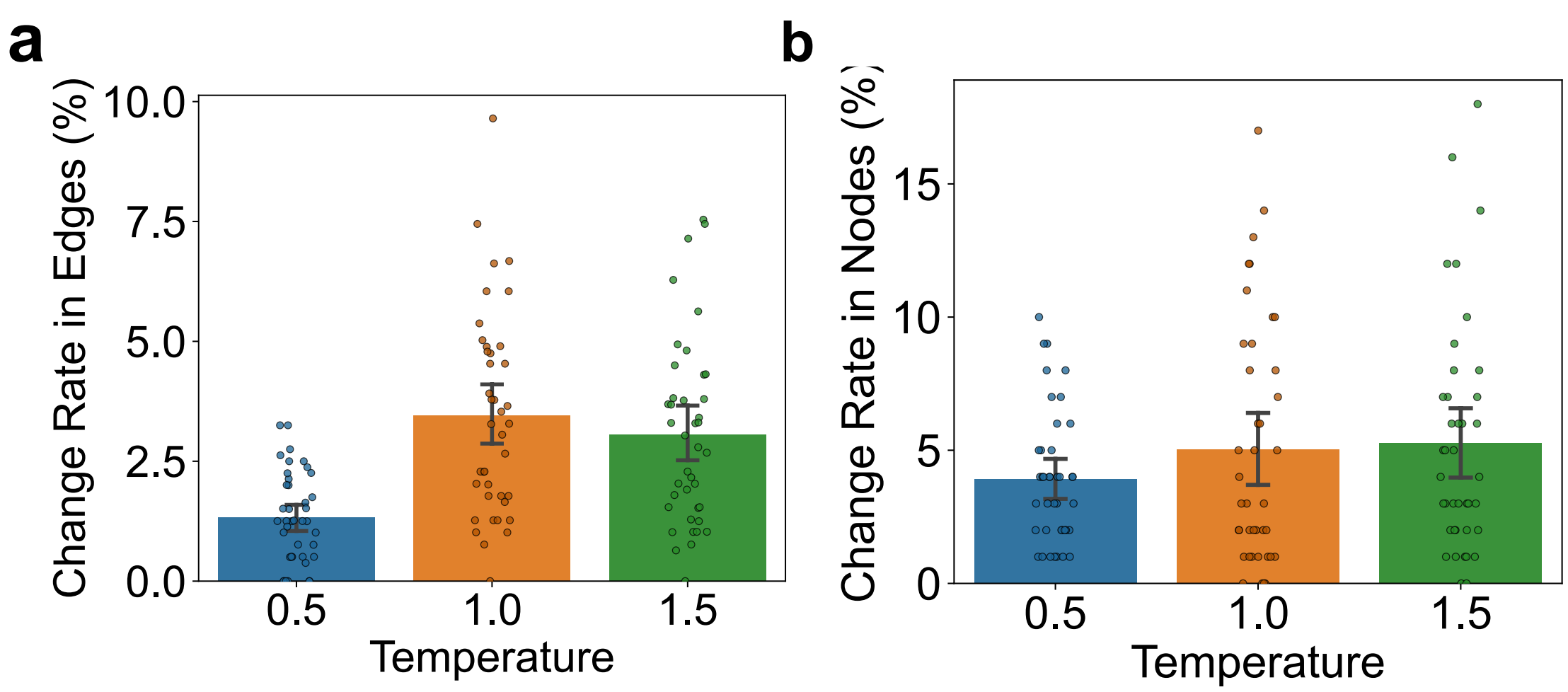

Figure S44. **Effects of temperature on the evolution a, of edges (i.e., social relationships between LLM agents), and b, nodal states (i.e., agents' opinions) in the social network.** Here, bars represent mean values, and error bars represent the corresponding 95% CIs. Each bar includes 40 samples, with each sample corresponding to the change from one timestep to the previous timestep.

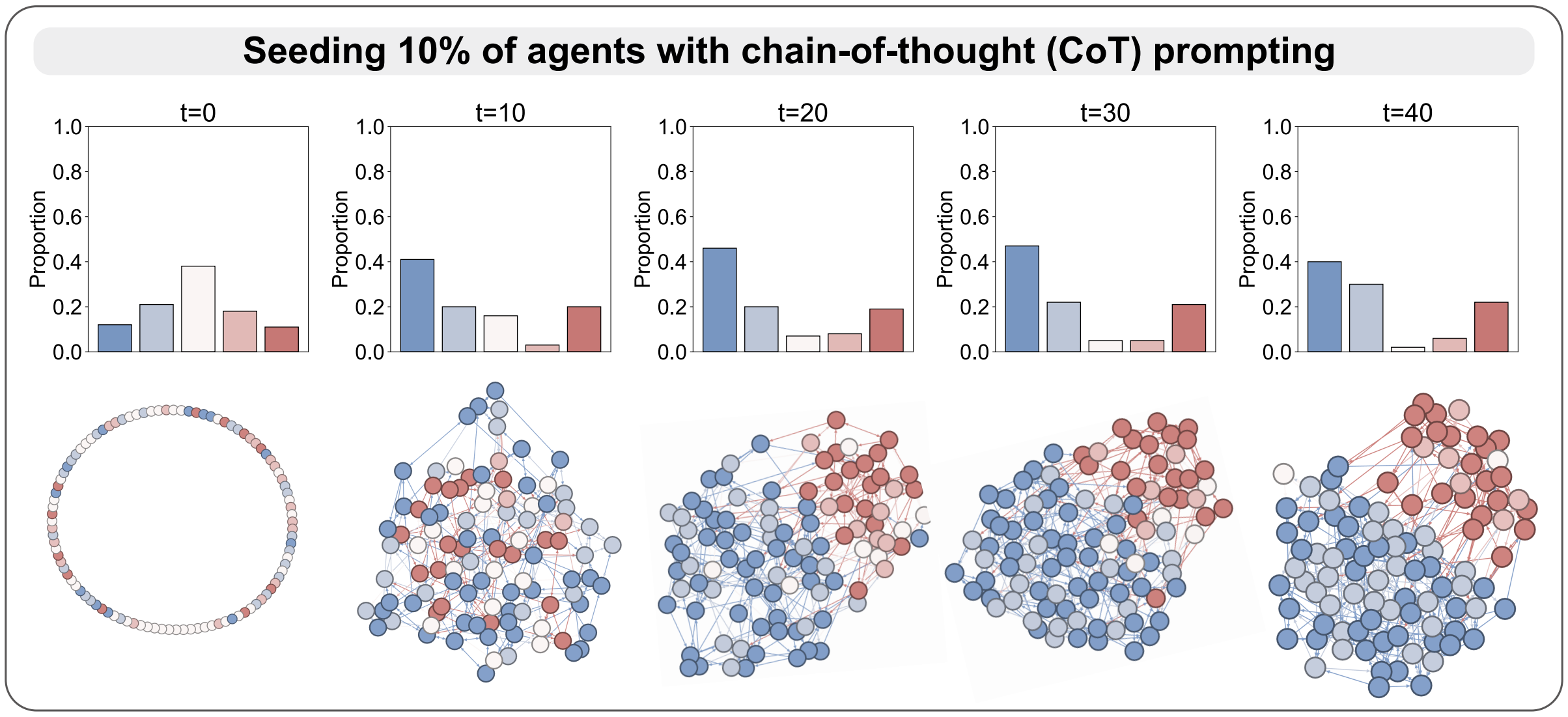

Figure S45. Evolution of LLM agents' collective opinions and network structures, where we seed 10% of agents with chain-of-thought (CoT) prompting. All agents are driven by GPT-3.5 and they discuss the political issue of abortion bans.

Furthermore, to precisely evaluate temperature effects, we measure both the level of polarization and proportion of homophilic interactions. As presented in Figure S43, our results show that while a higher temperature does not have a noticeable impact, a lower temperature markedly decreases the level of polarization in LLM agents' opinions and reduces the tendency of agents with similar opinions to cluster. This observation raises an interesting question: how does the diversity of LLM outputs, parameterized by temperature, affect the social behaviours of these agents, and in turn shape the level of polarization?

To answer this question, we further compute the change rate of edges, reflecting how frequently agents switch their communication partners, as well as the change rate of nodal states, indicating how often agents revise their opinions, across different temperature settings. Figure S44 illustrates the average values of both change rates over the entire simulation period. Interestingly, we observe that when the temperature is lowered to 0.5, agents exhibit a significantly lower propensity to switch communication partners, instead showing a strong preference for maintaining existing relationships (one-way ANOVA, F(2,117)= 16.90, p « .001; two-sided Student's t-test, temperature 0.5 vs. 1.5, t=-5.79, p«.001). Notably, agents driven by LLMs with a temperature of 0.5 adjust only 1.32% of their edges on average per timestep, which is less than half the rate observed at temperatures of 1.0 or 1.5. Their preference for maintaining existing relationships keeps them within their initial random networks, rather than selectively approaching more homophilic peers (Figure S44b), thereby contributing to the observed reduction in polarization level. Meanwhile, a lower temperature also moderately suppresses agents' tendency to adopt new opinions, although this effect is not statistically significant (Figure S44b). This tendency, combined with their reluctance to change communication partners, further contributes to the reduction in observed polarization.

11 Supplementary Note 10: Experiments on LLMs with Enhanced Prompting

We first explore whether seeding a minority of agents with a markedly different reasoning strategy can temper or amplify polarization. Specifically, we equip 10% of all agents with chain-of-thought (CoT) prompting, enabling them to think step by step as they establish social relationships, communicate, and form opinions on political issues. These CoT agents are seeded at the beginning of the simulation and randomly distributed across the network. The proportion of CoT agents across different opinions aligns with the overall initialization distribution.

Comparing the evolution of the original system with that of the system seeded with 10% CoT agents (Figures S45 and S46), we observe a substantial shift: the proportion of agents holding moderate or neutral opinions increases from 8% to 38% (Figure S45), and the overall polarization level drops by 15% (Figure S46a). Meanwhile, the network-level division between agents with opposing opinions is largely alleviated, with final modularity scores declining from 0.43 to 0.25 (Figure S46b; a modularity score above 0.3 typically marks clear community structures). Overall, these observations suggest that even a small minority of agents equipped with a markedly different reasoning strategy and seeded into the system can effectively temper polarization in the synthetic testbed. These results illustrate how the proposed synthetic LLM-agent testbed can be used to generate and examine hypotheses about how heterogeneity in agent-level reasoning configurations may shape collective opinion dynamics.

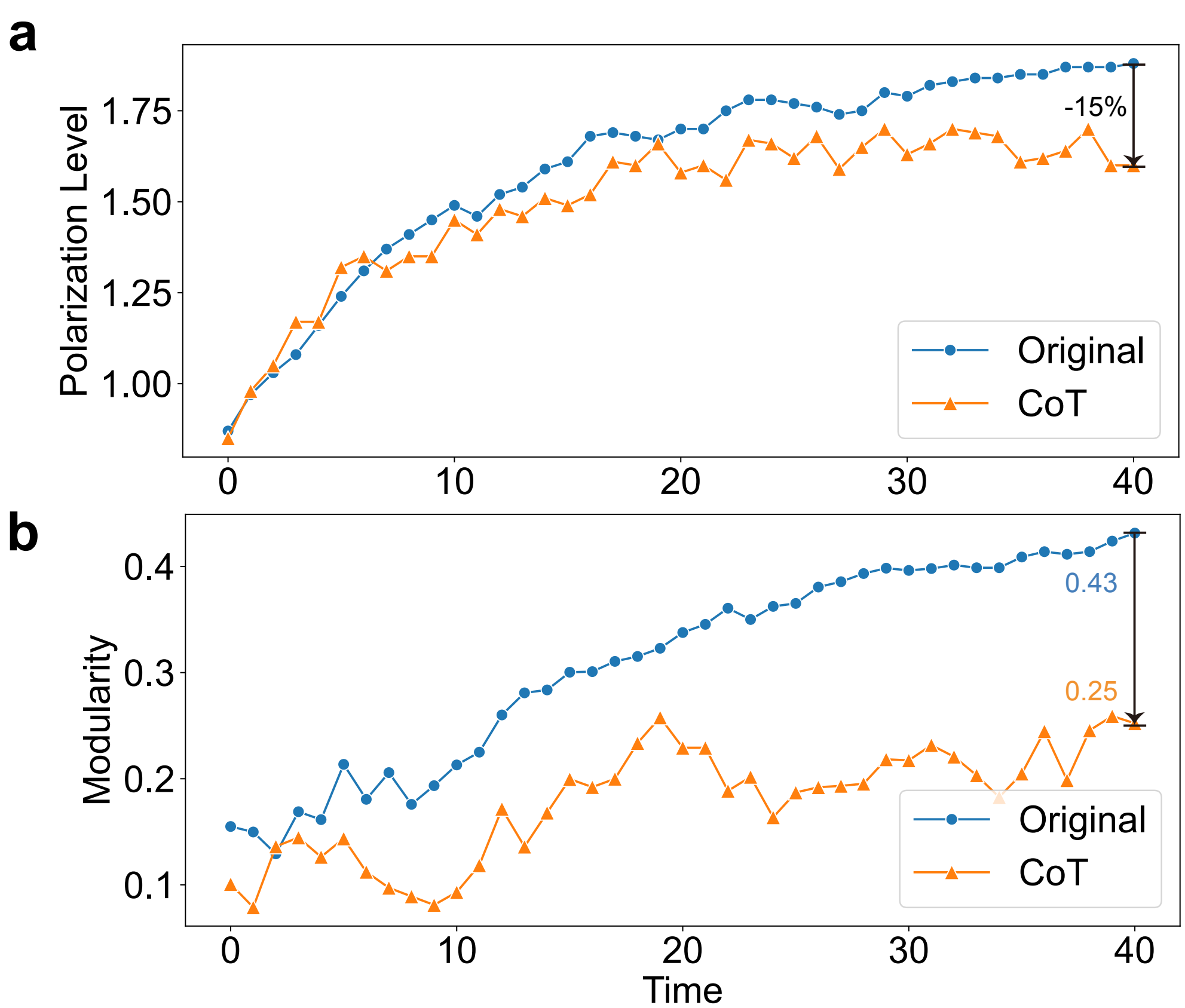

Figure S46. Comparison between the original system and a system where 10% of agents are seeded with CoT prompting, in terms of **a,** polarization level, and **b,** network modularity.

Furthermore, we investigate whether the proposed system can be extended, through enhanced prompting, to instantiate agents with subgroup-inspired profiles that reflect heterogeneous behavioural tendencies observed in human populations.. For example, we have conducted a simple case shown in Figure S47, where we use LLMs to generate two representative profiles of human subgroups in the U.S. and incorporate these profiles into the process of forming political opinions. Results show that LLM agents can simulate the two subgroups, capturing their distinctive behavioural patterns. Overall, this extended experiment illustrates that the proposed system remains flexible for modeling diverse human subgroups and their behavioural differences. We note that, in the main experiments of the paper, we intentionally do not insert additional subgroup-specific information into the agents' prompts in order to preserve the spontaneous nature of their behaviour.

12 Supplementary Note 11: Experiments on the Effect of Increasing Severing Costs

We examine the sensitivity of our results to the cost of severing social ties. To explore whether even minimal friction can slow the formation of ideological silos, we introduce a simple mechanism in which agents' decisions to sever existing social ties are blocked with a fixed probability (e.g., 10%), simulating minimal social friction. Figures S48 and S49 show the results under this minimal friction condition, where the cost of severing ties is increased by only 10%. Notably, while the minimal friction leads to marginal mitigation in polarization levels (Figure S49a), its effect on suppressing the formation of ideological silos is substantial, as consistently reflected across four different network-level metrics (Figures S49b–e). This experiment suggests that even minimal social friction can play a nontrivial role in slowing the formation of ideological silos and fostering communication between opposing camps.

13 Supplementary Note 12: Results of Simulation under Different Initial Conditions

To assess the robustness and generalizability of the proposed system, we investigate how different initial conditions affect the emergent collective behaviours of LLM agents. Specifically, we focus on two key aspects of initialization: the initial distribution of agents' opinions and the initial structure of the social network.

We first modify the initial opinion distribution by transitioning from a near-Gaussian distribution of $[0.1, 0.2, 0.4, 0.2, 0.1]$ to a highly centralized distribution of $[0, 0.1, 0.8, 0.1, 0]$. In this setting, a system that initially lacks polarized agents should be less likely to become polarized. Figure S50 shows the evolution of collective opinions in the system. We observe that the system, though taking a longer time to evolve, eventually converges to a polarized opinion distribution. Along with the emergence of opinion polarization, the social network gradually splits into two communities with opposing opinions. To sum up, this experiment demonstrates that even when the synthetic system is initialized in a setting unlikely to produce polarization, long-term free-form social interactions among LLM agents still lead to opinion polarization.

We also conduct experiments to explore whether the initial network affects the emergent collective behaviours of LLM agents. Specifically, we initialize LLM agents' social network with an Erdős–Rényi model, a Barabási–Albert model, and Watts–Strogatz models with rewiring probabilities of 0.001 and 0.05. In the BA model setup, a subset of agents naturally become highly connected nodes, resembling opinion leaders who exert disproportionate influence on others. Figures S51–S54 show how these agents self-organize their social networks and collective opinions under different initial conditions. All networks are visualized using the ForceAtlas2 algorithm with identical parameters in Gephi, ensuring direct visual comparability. We find that despite substantial differences in initial network structure, the final outcomes are similar. In all these cases, agents spontaneously organize into two well-defined communities: one that predominantly supports left-leaning opinions and the other right-leaning ones. Moreover, the final opinion distributions converge into a stable polarized pattern, demonstrating that the emergence of polarization and homophilic clustering is not driven by initial configurations, equitable or inequitable. Rather, these patterns are inherently driven by agents' autonomous social interactions. Overall, these results demonstrate the robustness and generalizability of our findings across diverse initial network structures, underscoring that LLM agents, through autonomous interactions, consistently self-organize into polarized communities in ways not determined by initial conditions.

14 Supplementary Note 13: Results of Simulation on Different Issues

To test the system's generalizability across different types of issues, we conduct two additional experiments beyond the political issues previously studied. The first relates to immigration restrictions, a socially relevant and critical issue. The second is the flat Earth theory, a non-political, fact-based issue with clear scientific consensus. These additions help demonstrate the generalizability of the proposed system and its corresponding results across both high-stakes political topics and more neutral or non-controversial domains in the synthetic testbed.

In particular, we illustrate the opinion dynamics when LLM agents discuss immigration restrictions in Figure S55. We observe that these agents spontaneously develop their collective opinions into a polarized pattern. This pattern emerges consistently across issues that have been shown to trigger polarization observed in human society. This consistency suggests that the proposed system can serve as a synthetic LLM-agent testbed for exploring opinion-dynamics hypotheses across different issue domains.

Furthermore, we wanted to understand how the proposed system would function on non-political, non-controversial issues. To this end, we test the system on a scientific issue – the flat Earth theory – where the topic is grounded in well-established scientific facts, rather than in polarized or contested opinions. As shown in Figure S56, we find that, unlike in political discussions, no polarization phenomenon emerges. Instead, agents rapidly reach a consensus within ten timesteps. These findings demonstrate that our system is not inherently predisposed to generate polarization. Rather, polarization emerges from free-form social interactions among LLM agents when dealing with inherently divisive topics, while it remains stable and convergent in neutral or fact-based domains.

15 Supplementary Note 14: Supplementary Notes on Statistical Testing and Robustness Checks

We assessed the assumptions of each reported frequentist test. For two-sample comparisons, normality was assessed using Shapiro–Wilk tests and equality of variances using Levene's tests; Student's t-tests were used when the equal-variance assumption was not violated, whereas Welch's t-tests were used otherwise. For tests involving large samples or sample proportions, the normal approximation of the relevant sampling distributions was justified by the central limit theorem. For proportion z-tests, formal normality and equal-variance tests were not applicable because the outcomes were binary proportions rather than continuous variables. For one-sample t-tests, equal-variance assumptions were not applicable. Where appropriate, we additionally conducted non-parametric robustness checks using Mann–Whitney U tests or Wilcoxon signed-rank tests.

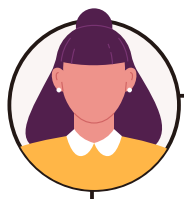

Profile: “A 29-year-old mixed White and Hispanic woman living in Brooklyn, New York, with a master’s degree in public policy. She works for a nonprofit organization, cares about social justice, climate change, and gender equality, and believes in strengthening community support systems. She mainly follows NPR and The New York Times and is active on Twitter and Instagram.”

Opinion on Partisan Alignment: I strongly support the Democratic Party.
“Most of my values—like advancing social justice, addressing climate change, protecting reproductive rights, and strengthening social safety nets—align with Democratic policies. While the party isn’t perfect and still has a lot of work to do, it’s the one actively pushing for more inclusive and equitable policies that reflect the kind of society I want to live in. I also believe in protecting democracy and voting rights, which I see as being under threat from the Republican side right now.”

Opinion on Abortion Ban: I strongly oppose abortion bans.
“I believe reproductive rights are fundamental to gender equality and personal freedom—no one should be forced to carry a pregnancy against their will. Abortion bans disproportionately harm low-income women and marginalized communities who already face barriers to healthcare, and they strip people of the right to make deeply personal decisions about their own bodies and lives.”

Opinion on Gun Control: I strongly support stricter gun control.
“The level of gun violence in this country is unacceptable, and common-sense measures like universal background checks, closing loopholes, and restricting access to high-capacity weapons can save lives without infringing on responsible ownership. Communities, especially children, deserve to feel safe in schools, neighborhoods, and public spaces, and stronger gun laws are a necessary step toward that safety.”

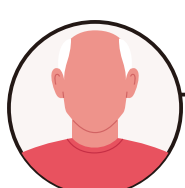

Profile: “A 66-year-old White man from rural Texas, a retired truck driver with a high school education. He values traditional family norms, personal responsibility, gun rights, low taxes, and stricter immigration policies, strongly supporting the Republican Party. He primarily consumes Fox News and Newsmax and engages on Facebook and in local church communities.”

Opinion on Partisan Alignment: I strongly support the Republican Party.
“They stand for the values I was raised on—faith, family, hard work, and personal freedom. I believe they do a better job protecting our rights, like the Second Amendment, keeping taxes low so folks can keep more of what they earn, and securing our borders to keep our communities safe.”

Opinion on Abortion Ban: I strongly support abortion bans.
“I believe life begins at conception, and every unborn child deserves a chance to live—that’s a value rooted in both my faith and my sense of personal responsibility. To me, protecting the most vulnerable is a moral duty, and I think communities and churches should step up to support mothers and families instead of turning to abortion as an answer.”

Opinion on Gun Control: I strongly support weaker gun control.
“The right to bear arms is a fundamental freedom that protects us from both criminals and government overreach. Out here in rural Texas, responsible gun ownership is a way of life, and I believe law-abiding citizens shouldn’t be punished or restricted because of the actions of a few bad actors.”

Figure S47. Two representative profiles of human subgroups in the U.S. and their different behaviours when discussing political issues. Credit: Profile icons designed by Magnific.

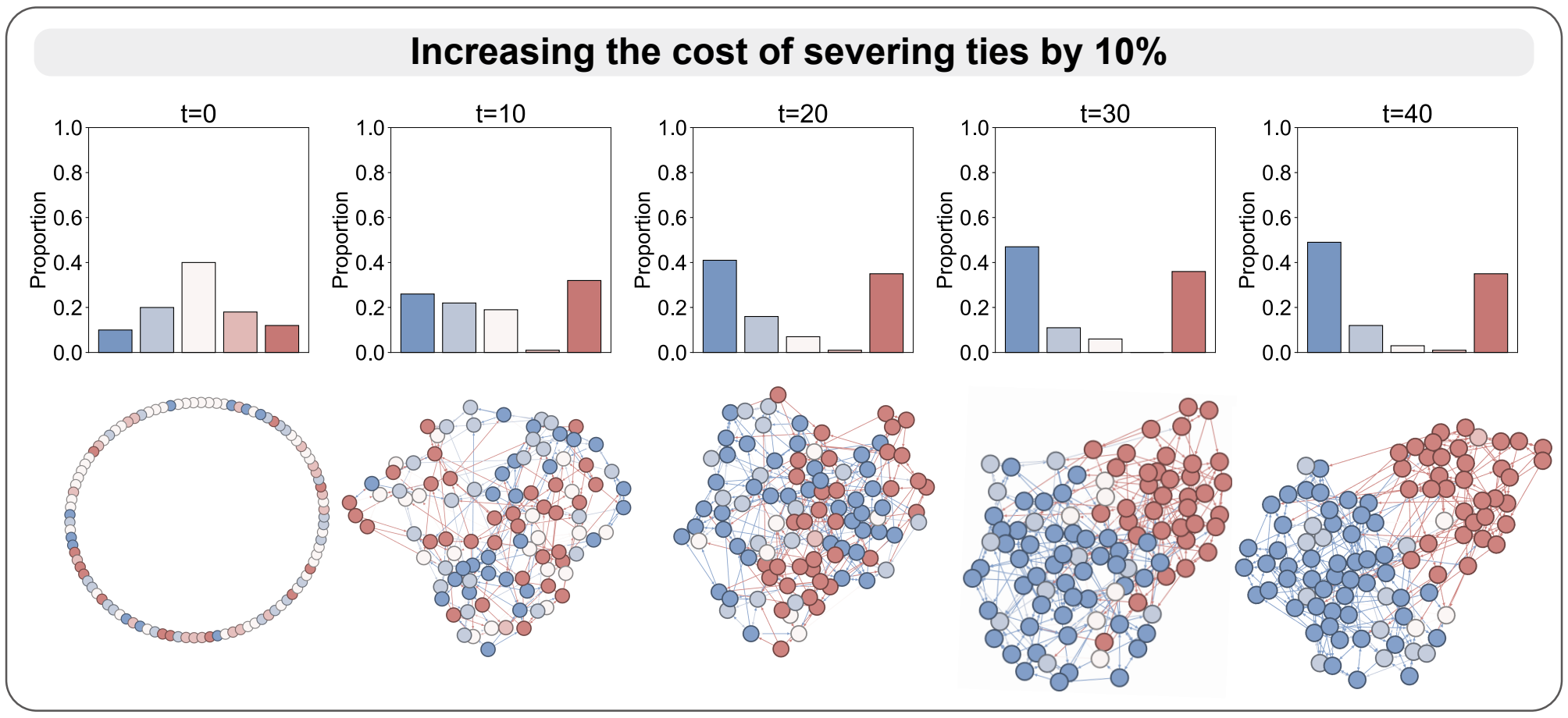

Figure S48. Evolution of LLM agents' collective opinions and network structures, where we increase the cost of severing ties by 10%. All agents are driven by GPT-3.5 and they discuss the political issue of abortion bans.

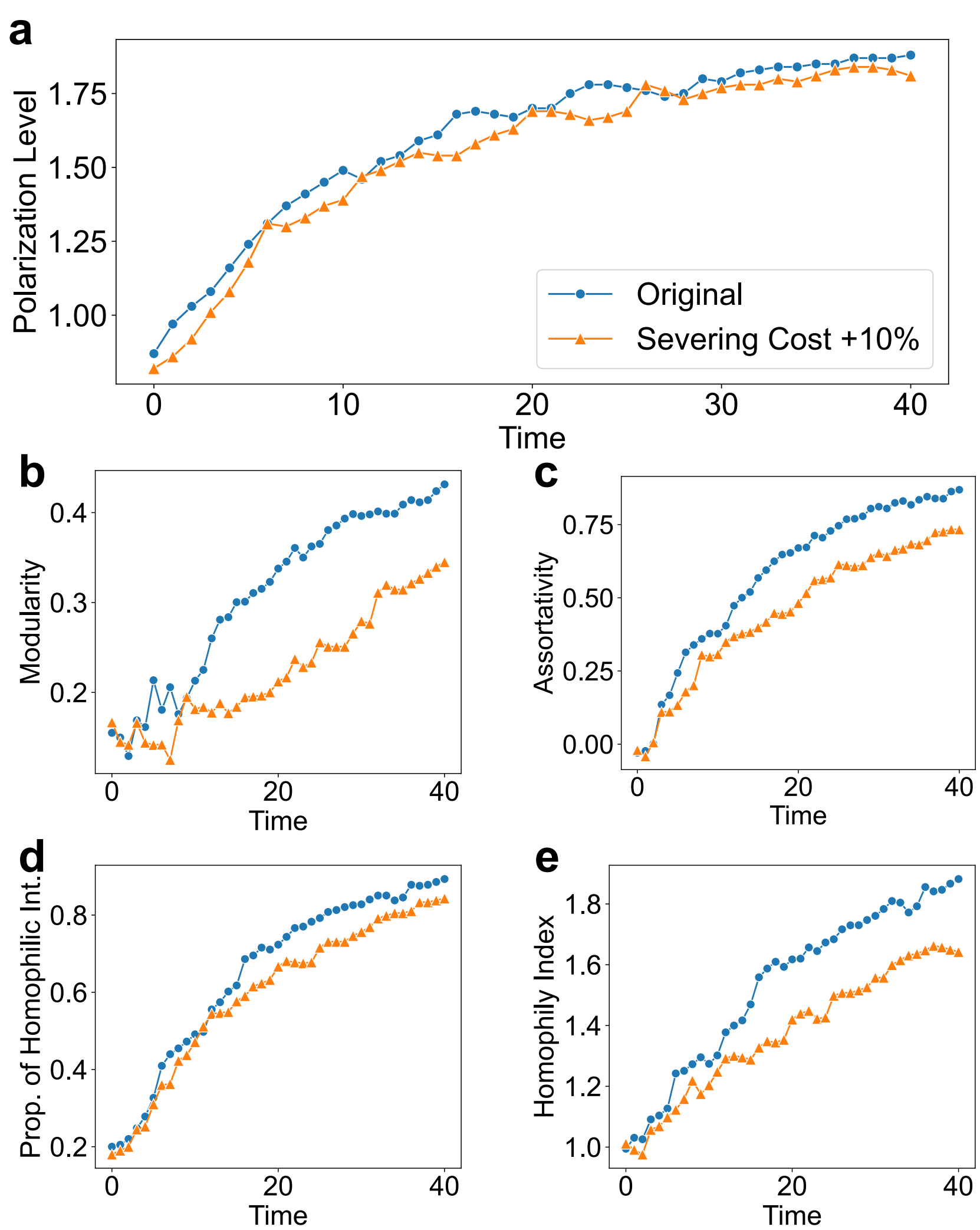

Figure S49. Comparison between the original system and a system where the cost of severing ties is increased by 10%, in terms of **a,** polarization level, **b,** network modularity, **c,** assortativity, **d,** proportion of homophilic interactions, and **e,** homophily index.

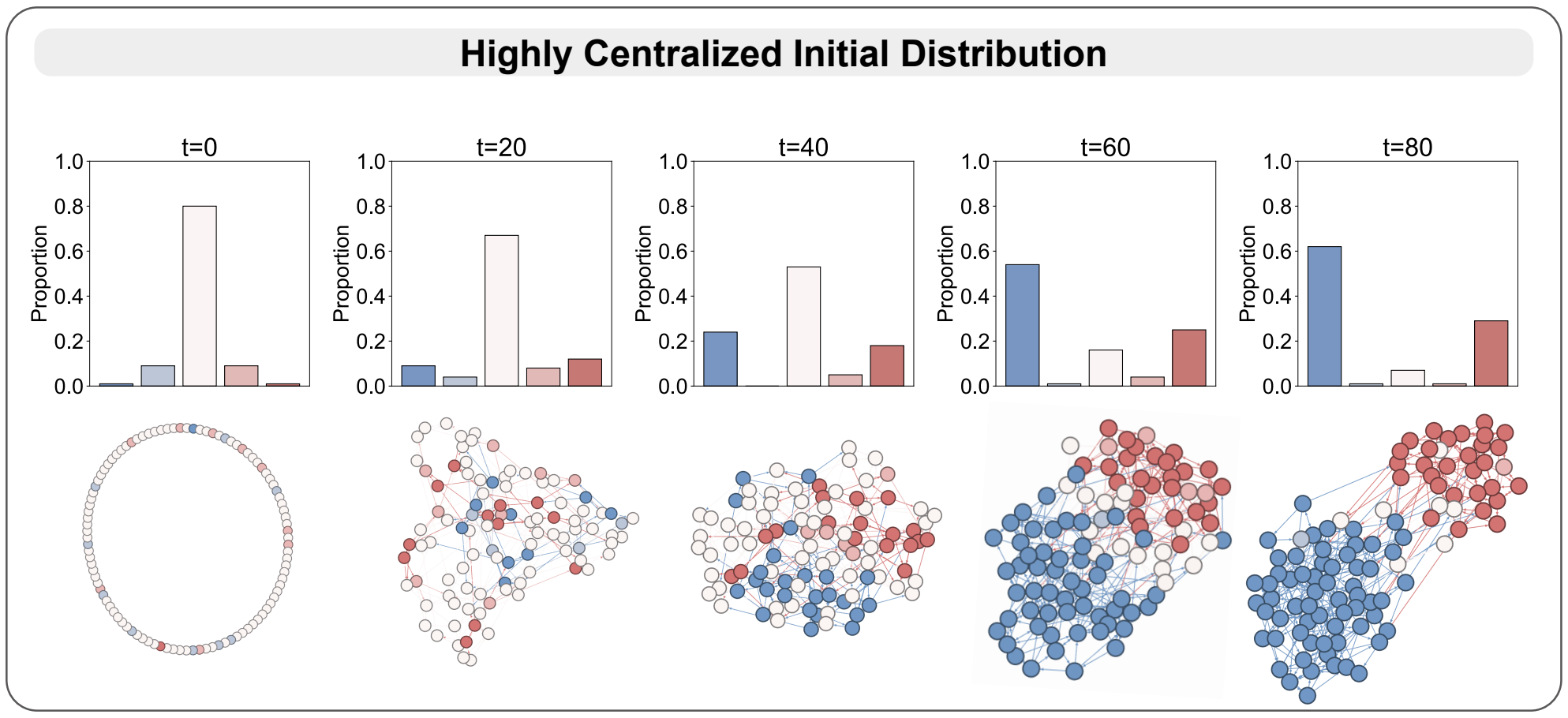

Figure S50. **Evolution of LLM agents' collective opinions and network structure**, where the network is initialized using a highly centralized distribution, and agents discuss the political issue of abortion ban.

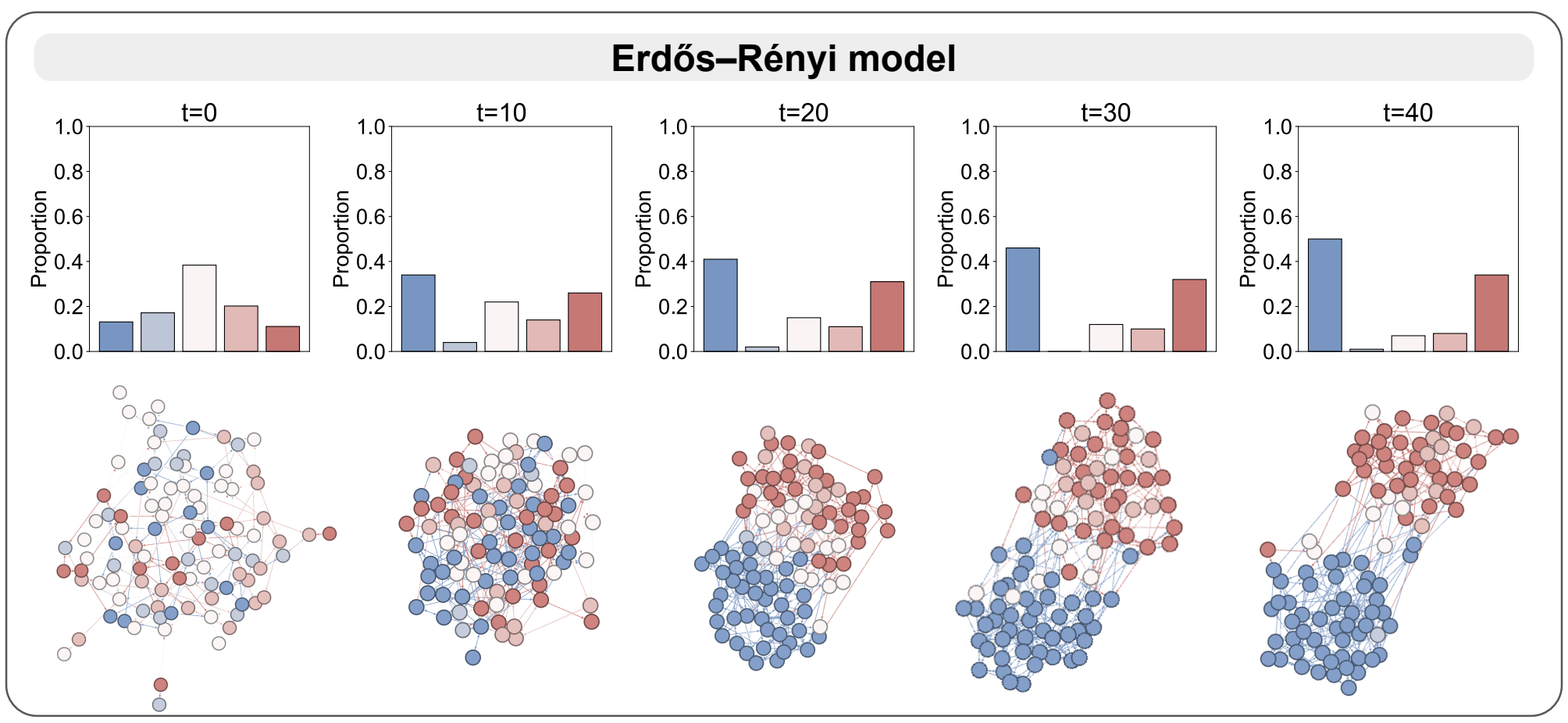

Figure S51. **Evolution of LLM agents' collective opinions and network structures**, where the network is initialized using an Erdős–Rényi model with an average degree of 4, and agents discuss the political issue of abortion ban.

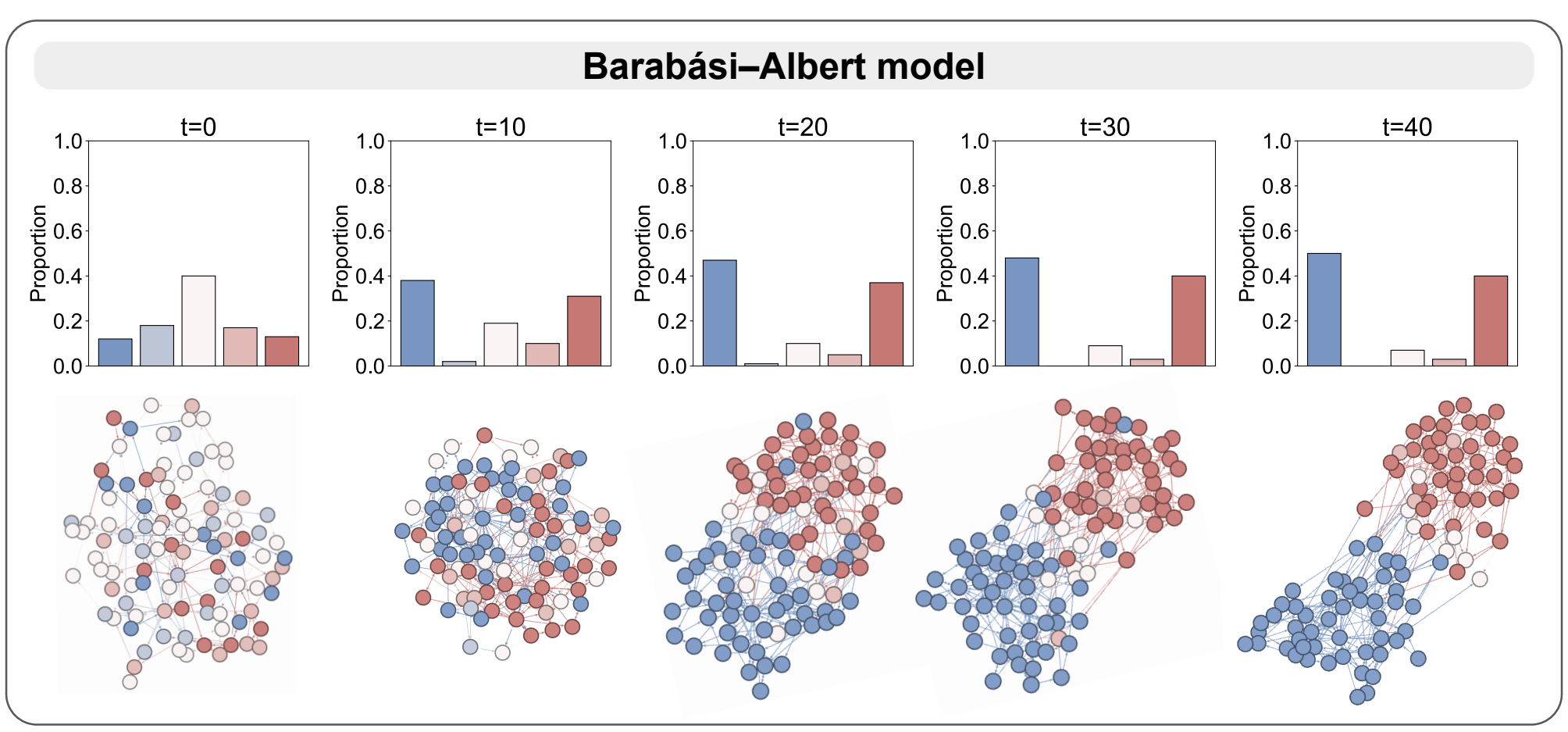

Figure S52. **Evolution of LLM agents' collective opinions and network structures**, where the network is initialized using a Barabási–Albert model with an average degree of 4, and agents discuss the political issue of abortion ban.

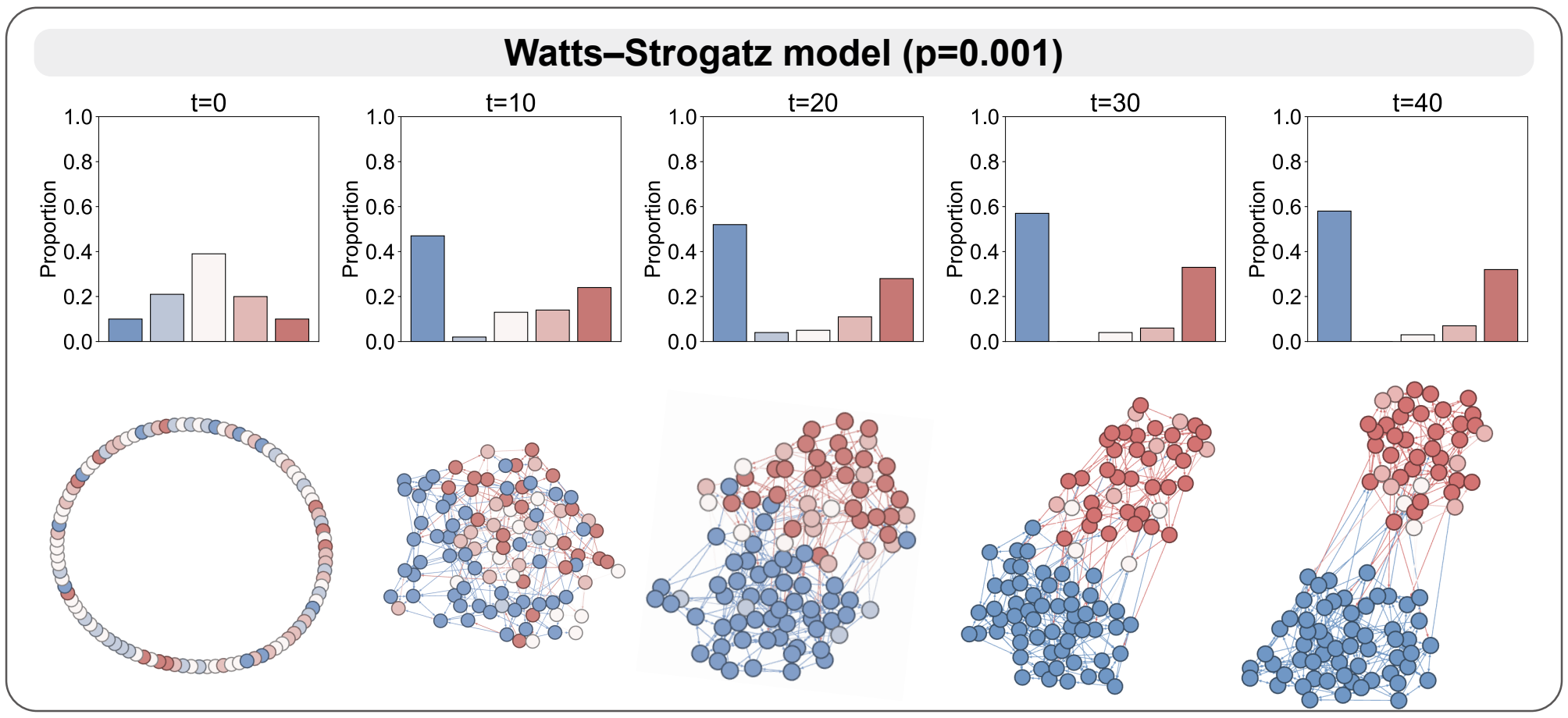

Figure S53. Evolution of LLM agents' collective opinions and network structures, where the network is initialized using a Watts–Strogatz model with the rewiring probability of 0.001, and agents discuss the political issue of abortion ban.

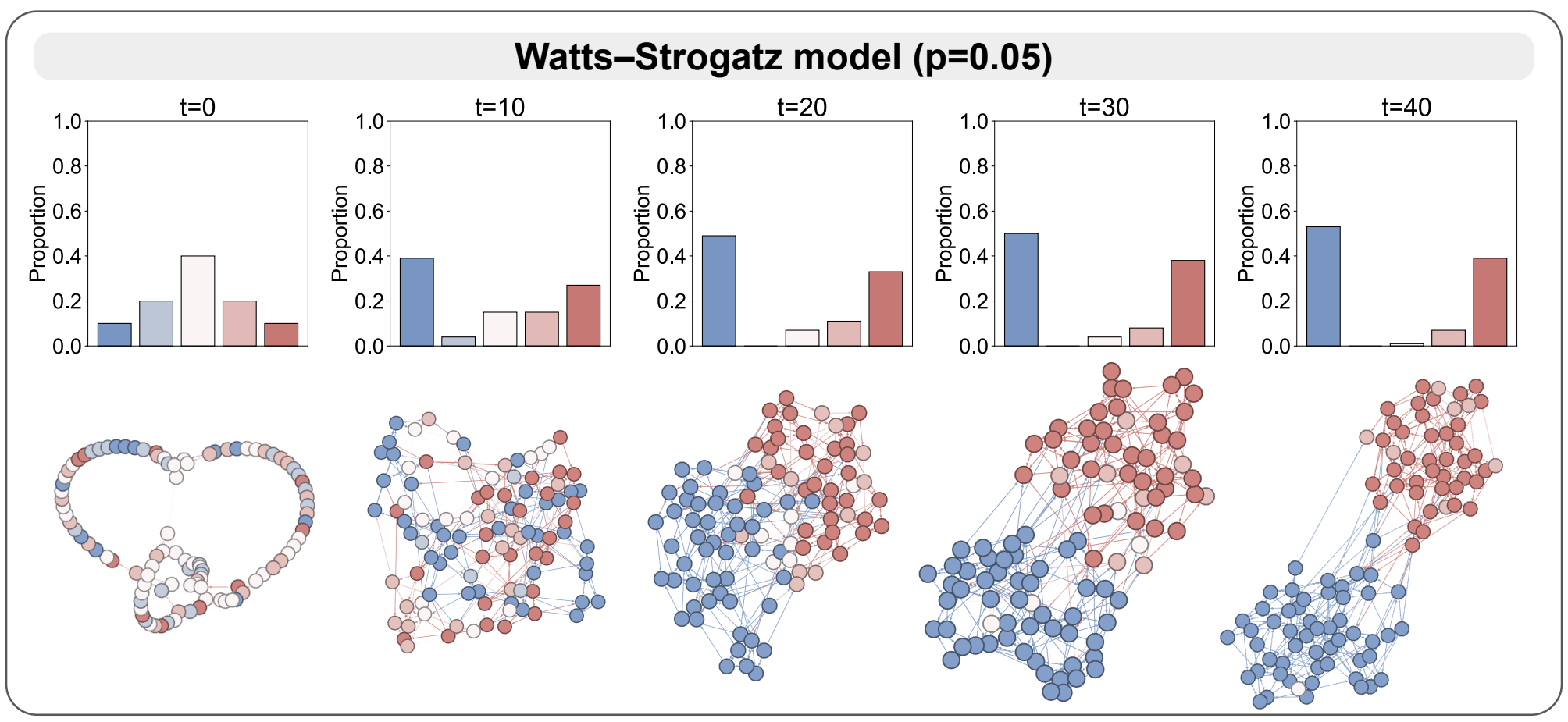

Figure S54. Evolution of LLM agents' collective opinions and network structures, where the network is initialized using a Watts–Strogatz model with the rewiring probability of 0.05, and agents discuss the political issue of abortion ban.

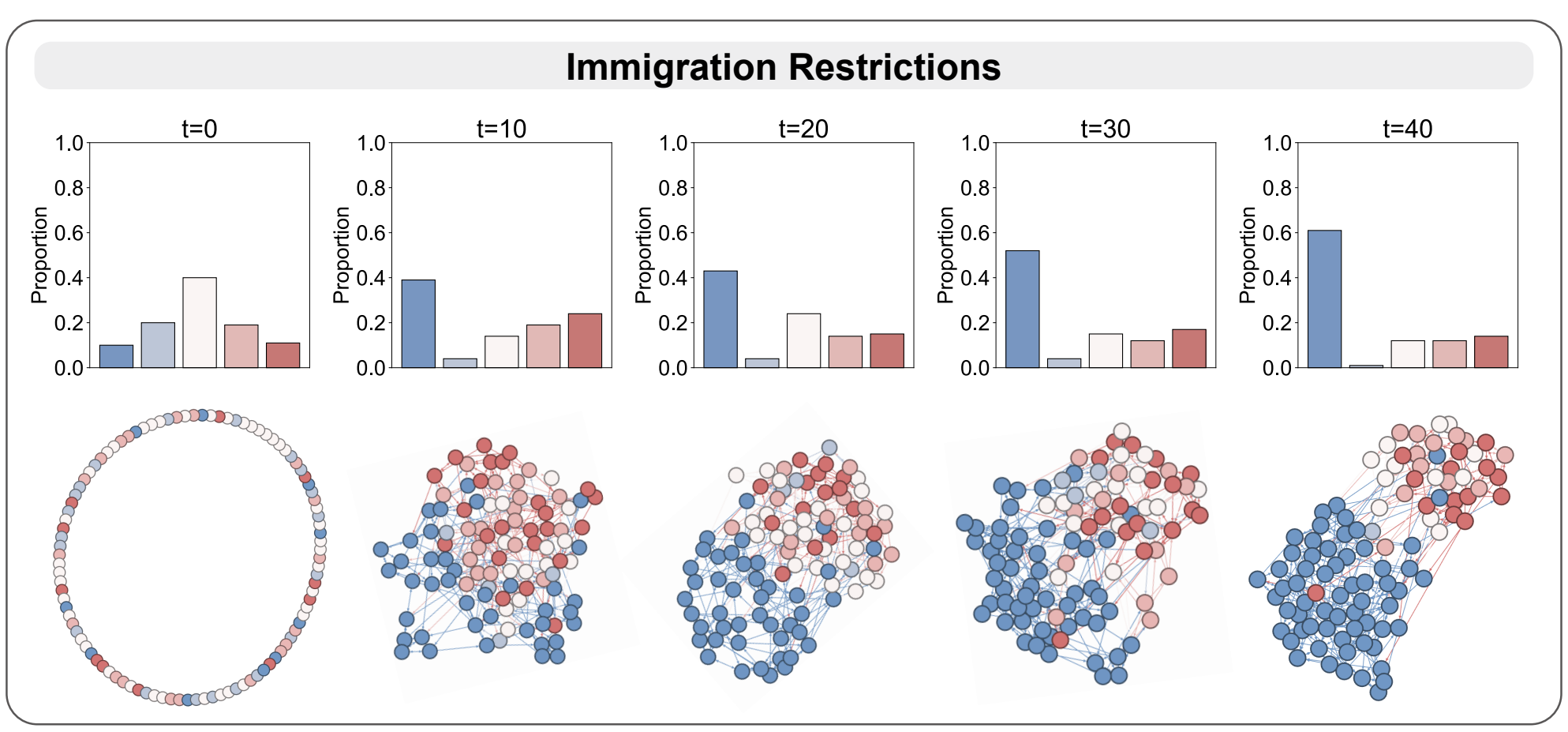

Figure S55. Evolution of LLM agents' collective opinions and network structures in the discussion of immigration.

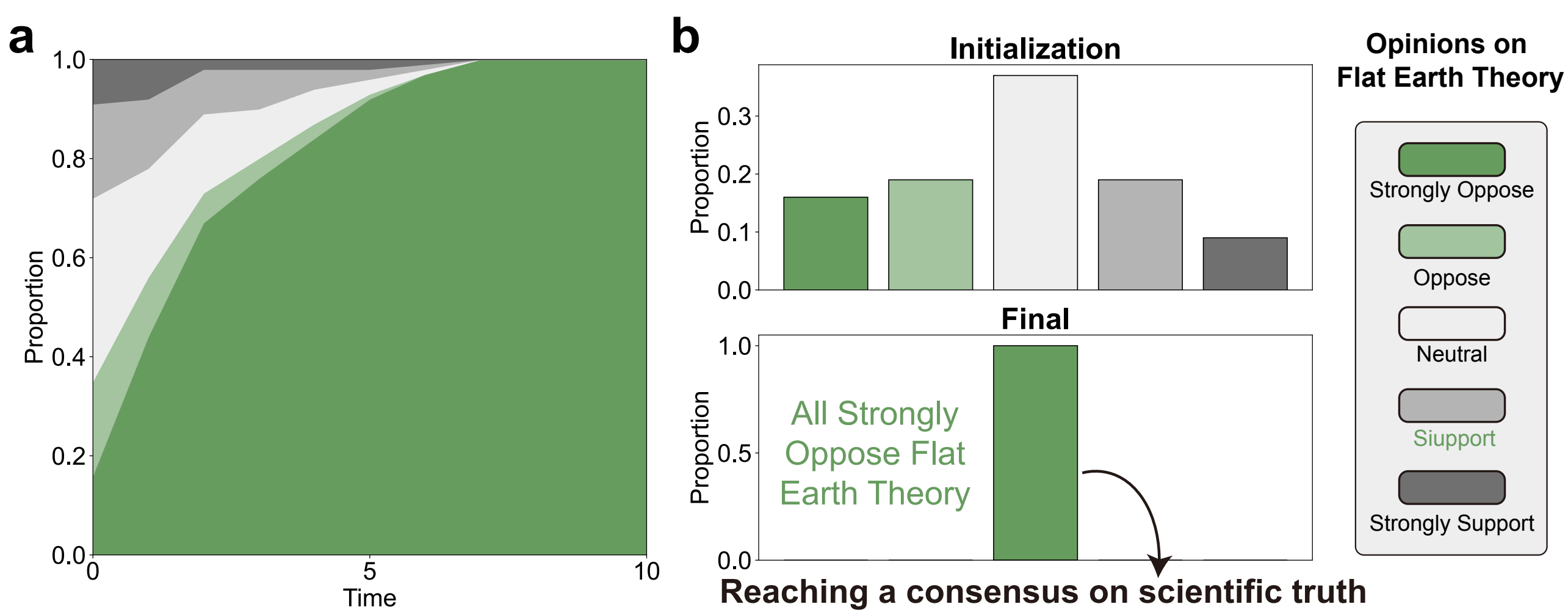

Figure S56. Opinion dynamics when LLM agents discuss about scientific truth. After 10 timesteps, all agents are persuaded to strongly oppose the flat Earth theory, reaching consensus.

References

[1] Lada A Adamic and Natalie Glance. "The political blogosphere and the 2004 US election: divided they blog". In: *Proceedings of the 3rd international workshop on Link discovery*. 2005, pp. 36–43.

[2] Douglas J Ahler and Gaurav Sood. "The parties in our heads: Misperceptions about party composition and their consequences". In: *The Journal of Politics* 80.3 (2018), pp. 964–981.

[3] American National Election Studies. *ANES 2020 Time Series Study Full Release [dataset and documentation]*. https://www.electionstudies.org. July 19, 2021 version. 2021.

[4] Christopher A. Bail, Lisa P. Argyle, Taylor W. Brown, John P. Bumpus, Haohan Chen, M. B. Fallin Hunzaker, Jaemin Lee, Marcus Mann, Friedolin Merhout, and Alexander Volfovsky. "Exposure to opposing views on social media can increase political polarization". In: *Proceedings of the National Academy of Sciences* 115.37 (2018), pp. 9216–9221.

[5] Eytan Bakshy, Solomon Messing, and Lada A Adamic. "Exposure to ideologically diverse news and opinion on Facebook". In: *Science* 348.6239 (2015), pp. 1130–1132.

[6] Albert-László Barabási and Réka Albert. "Emergence of scaling in random networks". In: *science* 286.5439 (1999), pp. 509–512.

[7] Fabian Baumann, Philipp Lorenz-Spreen, Igor M Sokolov, and Michele Starnini. "Emergence of polarized ideological opinions in multidimensional topic spaces". In: *Physical Review X* 11.1 (2021), p. 011012.

[8] Serina Chang, Alicja Chaszczewicz, Emma Wang, Maya Josifovska, Emma Pierson, and Jure Leskovec. "LLMs generate structurally realistic social networks but overestimate political homophily". In: *arXiv preprint arXiv:2408.16629* (2024).

[9] Matteo Cinelli, Gianmarco De Francisci Morales, Alessandro Galeazzi, Walter Quattrociocchi, and Michele Starnini. "The echo chamber effect on social media". In: *Proceedings of the National Academy of Sciences* 118.9 (2021), e2023301118.

[10] Alexander Coppock. *Persuasion in parallel: How information changes minds about politics*. University of Chicago Press, 2023.

[11] James N Druckman, Jordan Fein, and Thomas J Leeper. "A source of bias in public opinion stability". In: *American Political Science Review* 106.2 (2012), pp. 430–454.

[12] James N Druckman, Erik Peterson, and Rune Slothuus. "How elite partisan polarization affects public opinion formation". In: *American political science review* 107.1 (2013), pp. 57–79.

[13] Max Falkenberg, Alessandro Galeazzi, Maddalena Torricelli, Niccolò Di Marco, Francesca Larosa, Madalina Sas, Amin Mekacher, Warren Pearce, Fabiana Zollo, Walter Quattrociocchi, et al. "Growing polarization around climate change on social media". In: *Nature Climate Change* (2022), pp. 1–8.

[14] Shangbin Feng, Chan Young Park, Yuhan Liu, and Yulia Tsvetkov. "From Pretraining Data to Language Models to Downstream Tasks: Tracking the Trails of Political Biases Leading to Unfair NLP Models". In: *Proceedings of the 61st Annual Meeting of the Association for Computational Linguistics (Volume 1: Long Papers)*. 2023, pp. 11737–11762.

[15] Andreas Flache, Michael Mäs, Thomas Feliciani, Edmund Chattoe-Brown, Guillaume Deffuant, Sylvie Huet, and Jan Lorenz. "Models of social influence: Towards the next frontiers". In: *Jasss-The journal of artificial societies and social simulation* 20.4 (2017), p. 2.

[16] James Flamino, Alessandro Galeazzi, Stuart Feldman, Michael W Macy, Brendan Cross, Zhenkun Zhou, Matteo Serafino, Alexandre Bovet, Hernán A Makse, and Boleslaw K Szymanski. "Political polarization of news media and influencers on Twitter in the 2016 and 2020 US presidential elections". In: *Nature Human Behaviour* (2023), pp. 1–13.

[17] Santo Fortunato and Marc Barthelemy. "Resolution limit in community detection". In: *Proceedings of the national academy of sciences* 104.1 (2007), pp. 36–41.

[18] Eric Groenendyk and Yanna Krupnikov. "What motivates reasoning? A theory of goal-dependent political evaluation". In: *American Journal of Political Science* 65.1 (2021), pp. 180–196.

[19] Andrew Guess, Brendan Nyhan, Benjamin Lyons, and Jason Reifler. "Avoiding the echo chamber about echo chambers". In: *Knight Foundation* 2.1 (2018), pp. 1–25.

[20] Jochen Hartmann, Jasper Schwenzow, and Maximilian Witte. "The political ideology of conversational AI: Converging evidence on ChatGPT's pro-environmental, left-libertarian orientation". In: *arXiv preprint arXiv:2301.01768* (2023).

[21] John T Jost, Delia S Baldassarri, and James N Druckman. "Cognitive–motivational mechanisms of political polarization in social-communicative contexts". In: *Nature Reviews Psychology* 1.10 (2022), pp. 560–576.

[22] Woosub Lee, Seong-Gyu Yang, and Beom Jun Kim. “The effect of media on opinion formation”. In: *Physica A: Statistical Mechanics and its Applications* 595 (2022), p. 127075.

[23] Jeffrey Lees and Mina Cikara. “Inaccurate group meta-perceptions drive negative out-group attributions in competitive contexts”. In: *Nature human behaviour* 4.3 (2020), pp. 279–286.

[24] Jiazhen Liu, Shengda Huang, Nathaniel M Aden, Neil F Johnson, and Chaoming Song. “Emergence of polarization in coevolving networks”. In: *Physical Review Letters* 130.3 (2023), p. 037401.

[25] Ruibo Liu, Chenyan Jia, Jason Wei, Guangxuan Xu, and Soroush Vosoughi. “Quantifying and alleviating political bias in language models”. In: *Artificial Intelligence* 304 (2022), p. 103654.

[26] Charles G Lord, Lee Ross, and Mark R Lepper. “Biased assimilation and attitude polarization: The effects of prior theories on subsequently considered evidence.” In: *Journal of personality and social psychology* 37.11 (1979), p. 2098.

[27] Philipp Lorenz-Spreen, Lisa Oswald, Stephan Lewandowsky, and Ralph Hertwig. “A systematic review of worldwide causal and correlational evidence on digital media and democracy”. In: *Nature human behaviour* 7.1 (2023), pp. 74–101.

[28] Michael Mäs and Andreas Flache. “Differentiation without distancing. Explaining bi-polarization of opinions without negative influence”. In: *PloS one* 8.11 (2013), e74516.

[29] Fabio Motoki, Valdemar Pinho Neto, and Victor Rodrigues. “More human than human: Measuring ChatGPT political bias”. In: *Public Choice* 198.1 (2024), pp. 3–23.

[30] Mark EJ Newman. “Mixing patterns in networks”. In: *Physical review E* 67.2 (2003), p. 026126.

[31] Raymond S Nickerson. “Confirmation bias: A ubiquitous phenomenon in many guises”. In: *Review of general psychology* 2.2 (1998), pp. 175–220.

[32] Brendan Nyhan, Jaime Settle, Emily Thorson, Magdalena Wojcieszak, Pablo Barberá, Annie Y Chen, Hunt Allcott, Taylor Brown, Adriana Crespo-Tenorio, Drew Dimmery, et al. “Like-minded sources on Facebook are prevalent but not polarizing”. In: *Nature* (2023), pp. 1–8.

[33] Jon C Rogowski and Joseph L Sutherland. “How ideology fuels affective polarization”. In: *Political behavior* 38 (2016), pp. 485–508.

[34] Jérôme Rutinowski, Sven Franke, Jan Endendyk, Ina Dormuth, Moritz Roidl, and Markus Pauly. “The Self-Perception and Political Biases of ChatGPT”. In: *Human Behavior and Emerging Technologies* 2024.1 (2024), p. 7115633.

[35] Mohammad Reza Sanatkar. “The dynamics of polarized beliefs in networks governed by viral diffusion and media influence”. In: *Social Network Analysis and Mining* 10.1 (2020), p. 17.

[36] Fernando P Santos, Yphtach Lelkes, and Simon A Levin. “Link recommendation algorithms and dynamics of polarization in online social networks”. In: *Proceedings of the National Academy of Sciences* 118.50 (2021), e2102141118.

[37] Shibani Santurkar, Esin Durmus, Faisal Ladhak, Cinoo Lee, Percy Liang, and Tatsunori Hashimoto. “Whose opinions do language models reflect?” In: *International Conference on Machine Learning*. PMLR. 2023, pp. 29971–30004.

[38] Michael C Schwalbe, Geoffrey L Cohen, and Lee D Ross. “The objectivity illusion and voter polarization in the 2016 presidential election”. In: *Proceedings of the National Academy of Sciences* 117.35 (2020), pp. 21218–21229.

[39] Steven H Strogatz. “Exploring complex networks”. In: *nature* 410.6825 (2001), pp. 268–276.

[40] Natalie Jomini Stroud. “Polarization and partisan selective exposure”. In: *Journal of communication* 60.3 (2010), pp. 556–576.

[41] Cass R Sunstein. *# Republic: Divided democracy in the age of social media*. Princeton University Press, 2018.

[42] Isaac Waller and Ashton Anderson. “Quantifying social organization and political polarization in online platforms”. In: *Nature* 600.7888 (2021), pp. 264–268.

[43] Duncan J Watts and Steven H Strogatz. “Collective dynamics of ‘small-world’networks”. In: *nature* 393.6684 (1998), pp. 440–442.
