# Supplementary material for: Emergence of polarization in networks of large language model agents
Source: Nat Commun. 2026 Oct 8;17:11463. doi: 10.1038/s41467-026-78228-y (PMC13740318; doi:10.1038/s41467-026-78228-y)
Supplement: Supplementary file 2 [file 41467_2026_78228_MOESM2_ESM.pdf]

nature portfolio

Corresponding author(s): Fernando P. Santos, Yong Li, and James A. Evans

Last updated by author(s): Jul 12, 2026

Reporting Summary

Nature Portfolio wishes to improve the reproducibility of the work that we publish. This form provides structure for consistency and transparency in reporting. For further information on Nature Portfolio policies, see our Editorial Policies and the Editorial Policy Checklist.

Statistics

For all statistical analyses, confirm that the following items are present in the figure legend, table legend, main text, or Methods section.

n/a	Confirmed	
☐	☒	The exact sample size (*n*) for each experimental group/condition, given as a discrete number and unit of measurement
☒	☐	A statement on whether measurements were taken from distinct samples or whether the same sample was measured repeatedly
☐	☒	The statistical test(s) used AND whether they are one- or two-sided<br>*Only common tests should be described solely by name; describe more complex techniques in the Methods section.*
☐	☒	A description of all covariates tested
☒	☐	A description of any assumptions or corrections, such as tests of normality and adjustment for multiple comparisons
☐	☒	A full description of the statistical parameters including central tendency (e.g. means) or other basic estimates (e.g. regression coefficient) AND variation (e.g. standard deviation) or associated estimates of uncertainty (e.g. confidence intervals)
☐	☒	For null hypothesis testing, the test statistic (e.g. *F*, *t*, *r*) with confidence intervals, effect sizes, degrees of freedom and *P* value noted<br>*Give P values as exact values whenever suitable.*
☒	☐	For Bayesian analysis, information on the choice of priors and Markov chain Monte Carlo settings
☒	☐	For hierarchical and complex designs, identification of the appropriate level for tests and full reporting of outcomes
☒	☐	Estimates of effect sizes (e.g. Cohen's *d*, Pearson's *r*), indicating how they were calculated

Our web collection on statistics for biologists contains articles on many of the points above.

Software and code

Policy information about availability of computer code

Data collection

Custom python codes are used to implement the networked system of large language model agents.
We use ChatGPT (GPT-3.5) through the public OpenAI API to run all the experiments in the Main Text. We also perform experiments using other LLMs, including GPT-4o, ChatGLM, and Llama-3.
Code is available at: https://github.com/tsinghua-fib-lab/LLM_for_Polarization.

Codes are written in Python using the following packages:
python == 3.8.11
numpy == 1.23.5
scipy == 1.10.1
scikit-learn == 1.2.2
matplotlib == 3.7.1
seaborn == 0.12.2
jupyter notebook == 6.4.8
openai==0.28.0

Data analysis

Analyses are performed in Python. Code is available at: https://github.com/tsinghua-fib-lab/LLM_for_Polarization.

Codes are written in Python using the following packages:
python == 3.8.11
numpy == 1.23.5
scipy == 1.10.1

```
scikit-learn ==  1.2.2
matplotlib == 3.7.1
seaborn == 0.12.2
jupyter notebook == 6.4.8
openai==0.28.0
```

For manuscripts utilizing custom algorithms or software that are central to the research but not yet described in published literature, software must be made available to editors and reviewers. We strongly encourage code deposition in a community repository (e.g. GitHub). See the Nature Portfolio guidelines for submitting code & software for further information.

Data

Policy information about availability of data

All manuscripts must include a data availability statement. This statement should provide the following information, where applicable:

- Accession codes, unique identifiers, or web links for publicly available datasets
- A description of any restrictions on data availability
- For clinical datasets or third party data, please ensure that the statement adheres to our policy

We have provided a full data availability statement in the manuscript. The data used in this research is available at https://github.com/tsinghua-fib-lab/LLM_for_Polarization.

Research involving human participants, their data, or biological material

Policy information about studies with human participants or human data. See also policy information about sex, gender (identity/presentation), and sexual orientation and race, ethnicity and racism.

Reporting on sex and gender	We did not have human research participants.
Reporting on race, ethnicity, or other socially relevant groupings	We did not have human research participants.
Population characteristics	We did not have human research participants.
Recruitment	We did not have human research participants.
Ethics oversight	We did not have human research participants.

Note that full information on the approval of the study protocol must also be provided in the manuscript.

Field-specific reporting

Please select the one below that is the best fit for your research. If you are not sure, read the appropriate sections before making your selection.

☐ Life sciences ☒ Behavioural & social sciences ☐ Ecological, evolutionary & environmental sciences

For a reference copy of the document with all sections, see nature.com/documents/nr-reporting-summary-flat.pdf

Behavioural & social sciences study design

All studies must disclose on these points even when the disclosure is negative.

Study description	This paper simulates thousands of large language model agents in networked social systems to explore their opinion dynamics and collective behaviours.
Research sample	Large language models: GPT-3.5 (and other GPT-4o, ChatGLM, and Llama-3 models reported in Supplementary Information)
Sampling strategy	We do not apply any sampling strategy on the large language model agents.
Data collection	We simulate thousands of large language model agents in networked social systems and collect their opinions and interactions.
Timing	Data was collected between October 2023 and August 2025.
Data exclusions	No data were excluded from the analyses.
Non-participation	Our research did not include any direct human participants.
Randomization	Our research is based on the simulation of large language model agents. Thus, randomization is not involved.

Reporting for specific materials, systems and methods

We require information from authors about some types of materials, experimental systems and methods used in many studies. Here, indicate whether each material, system or method listed is relevant to your study. If you are not sure if a list item applies to your research, read the appropriate section before selecting a response.

Materials & experimental systems

n/a	Involved in the study
☒	☐ Antibodies
☒	☐ Eukaryotic cell lines
☒	☐ Palaeontology and archaeology
☒	☐ Animals and other organisms
☒	☐ Clinical data
☒	☐ Dual use research of concern
☒	☐ Plants

Methods

n/a	Involved in the study
☒	☐ ChIP-seq
☒	☐ Flow cytometry
☒	☐ MRI-based neuroimaging

Plants

Seed stocks	*Report on the source of all seed stocks or other plant material used. If applicable, state the seed stock centre and catalogue number. If plant specimens were collected from the field, describe the collection location, date and sampling procedures.*
Novel plant genotypes	*Describe the methods by which all novel plant genotypes were produced. This includes those generated by transgenic approaches, gene editing, chemical/radiation-based mutagenesis and hybridization. For transgenic lines, describe the transformation method, the number of independent lines analyzed and the generation upon which experiments were performed. For gene-edited lines, describe the editor used, the endogenous sequence targeted for editing, the targeting guide RNA sequence (if applicable) and how the editor was applied.*
Authentication	*Describe any authentication procedures for each seed stock used or novel genotype generated. Describe any experiments used to assess the effect of a mutation and, where applicable, how potential secondary effects (e.g. second site T-DNA insertions, mosiacism, off-target gene editing) were examined.*
