# Supplementary material for: Emergence of polarization in networks of large language model agents
Source: Nat Commun. 2026 Oct 8;17:11463. doi: 10.1038/s41467-026-78228-y (PMC13740318; doi:10.1038/s41467-026-78228-y)
Supplement: Supplementary file 3 [file 41467_2026_78228_MOESM3_ESM.pdf]

nature portfolio

Peer Review File

Emergence of polarization in networks of large language model agents

Corresponding Author: Professor James Evans

This manuscript has been previously reviewed at another journal. This document only contains information relating to versions considered at Nature Communications.

This file contains all reviewer reports in order by version, followed by all author rebuttals in order by version.

Version 0:

Reviewer comments:

Reviewer #1

(Remarks to the Author)
The manuscript is progressing in a positive direction. The additional robustness checks, clearer methodological exposition, and newly introduced intervention experiments make the paper markedly more transparent and empirically rigorous than the initial draft. Moreover, the integration of the rapidly evolving literature and the articulation of practical implications demonstrate commendable responsiveness to earlier feedback.

Nevertheless, the revision now references Tessler, Chang, and other recent studies while framing its contribution as permitting the co-evolution of edges and opinions within an autonomous-agent society. Yet the principal empirical finding—LLM agents drifting into echo chambers—largely mirrors results already documented in those studies and in decades of social-influence modelling. Without a sharper theoretical claim or an unforeseen mechanism that earlier work could not have anticipated, the paper presently appears more a scaled-up replication enhanced by polished visualisations than a step change in understanding.

That said, the new framework opens fertile avenues for further inquiry. For example, what occurs if agents can “defect” to a rival language model mid-conversation, thereby altering not only their social ties but also their cognitive architecture? Might seeding a minority of agents with a markedly different reasoning strategy (e.g., chain-of-thought prompting) temper or amplify polarisation? Finally, how sensitive are the results to the cost of severing ties—does even minimal friction slow the formation of ideological silos? Addressing these questions could convert the platform into a robust test bed for theories that extend beyond the current version.

Reviewer #2

(Remarks to the Author)
I was reviewer 2 on the NHB submission. I am extremely impressed with the efforts undertaken by the authors. While I am not able to fully appreciate the technical contributions, I appreciate the clarity of technical writing. I also am now much clearer on the substantive implications. It is now apparent how the infrastructure can be used to evaluate human behavior and interventions therein – in essence an extremely sophisticated simulation. One question I have is whether there is a secondary contribution. I was intrigued by the authors’ point that most work on LLM-human interactions involve unitary LLMs. Is it interesting that one could now explore that interaction with networked LLMs?

I appreciate the incorporation of some of the literature I had mentioned and how the authors’ work aligns with that work (while also ostensibly being dependent on how parameter values are set). I have a few questions. First, the addition of two more issues is useful. I am a little confused by the labeling “alarming” issues – what exactly does this mean and how is it evaluated? Is it just polarized issues? I ask because right now in the US, immigration restrictions are very alarming but in the paper this is treated as not an alarming issue. This is largely a semantic question. Second, I enjoyed the introduction, but it leads to the expectation that the authors might provide insight into whether LLMs have particular impacts (such as in the 2016 election). I think the authors need to be careful here since there is not a comparison with basic bots or looking at the

direct effects. The authors may consider revising that part of the introduction to make sure it is clear where the paper is going (a la my points/question in the first paragraph above). Third, this is again a question from someone without technical expertise in this area. I take it that one can manipulate the parameters (as the authors do) to get distinct types of psychological processes? If so, that may be a point worth mentioning more explicitly (e.g. if one wanted to explore distinct human subgroups that behavior differently, is that possible?). Fourth, it would have been helpful to have more detail about the psychological process at the opinion update stage – beyond the assumptions and before the individual simulations. What exactly is being assumed here? Fifth, I was not clear how the top influences were identified. Sixth, the initialization is the starting point and so I wonder whether it should be in the first panel in Figure 1c.

These are all relatively small questions. I'm impressed with the revision.

Reviewer #3

(Remarks to the Author)
I have reviewed a previous version of this work at a related journal and gave "major revision" with various suggestions. I see that most of them are well-addressed by the authors in this version: The authors now better engagement with the social/political biases studies of LLMs, clarified various details about agent interactions (e.g. how they select another agent for interaction), etc. I would suggest to discuss the limitations of this work being a synthetic and simulated study, and what gaps and differences do the authors foresee between a synthetic LLM study and real-world human communication: what parts of the conclusions are most applicable and what might be less. Other then that the authors' revisions are adequate.

Version 1:

Reviewer comments:

Reviewer #2

(Remarks to the Author)
I am extremely impressed with the authors' engagement -- it is among the most impressive set of revisions (from the initial draft) that I have seen. I support publication. I also thank the authors as I find the paper much more accessible than the first version (and I am more of a polarization scholar and so, as I had mentioned, did not grasp some of the technical detail in the first draft).

(Remarks on code availability)

Reviewer #3

(Remarks to the Author)
My second round of comments are well addressed: well done! The paper is ready for acceptance.

(Remarks on code availability)
Appears to be useful in reproducing the results.

Reviewer #4

(Remarks to the Author)
Reviewer 1 – Represented by Reviewer 4

I am Reviewer 4, standing in for Reviewer 1, to assess whether the authors have satisfactorily addressed Reviewer 1's comments.

Reviewer 1's main concern was that the paper does not make a substantive theoretical contribution. Specifically, they argued that it "mirrors results already documented in previous studies and in decades of social-influence modelling. Without a sharper theoretical claim or an unforeseen mechanism that earlier work could not have anticipated, the paper presently appears more a scaled-up replication enhanced by polished visualisations than a step change in understanding." The reviewer also suggested possible directions for further exploration.

The authors responded that their contribution is primarily methodological rather than theoretical. They argue that the paper opens up a new methodological arena for studying society and social media. The argument is in this sense similar to arguments put forth about generative agent-based models (GABMs) in a host of other papers over the last 2-3 years (e.g., Park et al. 2023, 2022; Törnberg et al. 2023; Orlando et al 2025; Gu et al 2025 – who also look at echo chamber formation). The paper's main contribution, as they frame it, is to demonstrate that these models reproduce certain patterns of human behavior.

The authors have moreover followed Reviewer 1's suggestions and explored the proposed additional issues. Their patience and diligence in engaging with the feedback are commendable.

In my view, the addition of these analysis does not help in the sense of providing new theoretical insights. The additional analyses are interesting but do not uncover a previously unobserved social mechanism in a way that helps our understanding. For the paper to contribute theoretically, it would need to reveal mechanisms that are both novel and externally valid within a social-scientific framework. Some form of plausible social mechanism would need to be identified and understood.

But given the nature and scope of the work at hand, I find the authors' response satisfactory in addressing Reviewer 1's comments. The contribution of the paper, as it stands, is best understood as offering an interesting and illustrative toy model that advances methodological discussion rather than theoretical understanding. This is a legitimate and worthwhile contribution, especially given the emerging nature of this research area. I consider reviewer 1 satisfied with the revisions.

Reviewer 4's own concerns
Below is the original review report of Reviewer 4, written in my capacity as Reviewer 4. However, I wrote this review while being under the impression that the editors were seeking a novel perspective, rather than a replacement for reviewer 1. I recognize that it may not be entirely appropriate to introduce a new perspective at this late stage of the review process. I therefore leave it to the editors' discretion to determine which - if any - of the points below should still be addressed.

That said, I believe it would be useful to provide a concise list of minimal revisions that would make me comfortable recommending the paper for publication, without requiring much work for the authors. These are primarily textual clarifications and moderation of claims, requiring little additional work. (The editors may however raise additional issues to address from my comments below, if they see it fit to do so.)

Minimal actionable points to address:

- Replace all claims of "modeling bot networks" or "LLM agents in the wild" with "synthetic LLM-agent testbed" language, or similar.
- Remove any suggestions of policy guidance or direct deployment implications. Reframe as hypothesis-generating only. E.g.: "We present a synthetic testbed of networked LLM agents for pre-experimental exploration of opinion-dynamics hypotheses." Remove or revise claims of bot networks. Remove claims of "theoretical contributions".
- Explicitly acknowledge the argument-accumulation modeling lineage. Acknowledge that the results align with established opinion-dynamics models (e.g., Mäs & Flache 2013), and likely stems from similar underlying mechanisms. (You could also actually test this, but it would be more work.)
- Delete or soften "human-like," "non-trivial," "remarkable," "Homo silicus," and "uncontrollable."
- Mention the ongoing debate in the GABM field on the challenges of using GABMs to simulate human behavior, due to the challenges of validation and calibration limiting the possibility of showing external validity.
- When naming psychological or social mechanisms (confirmation bias, motivated reasoning, echo chambers), try to use formulations such as "behavior consistent with X" and specify when these are inferred from LLM self-reports.
- Acknowledge key theoretical debates in the literature on social media and polarization: critiques of the echo-chamber thesis, limitations of deliberative/argument-accumulation models, and newer conceptions of polarization (affective, identity-based).
- Cite/engage with the existing literature on simulating social media using LLMs to position the work in the current wave of GABM research, preventing the impression of overclaiming originality.

Implementing these limited textual changes would, in my view, adequately temper the paper's claims and address its main overreach without imposing significant additional workload on the authors.

My full original review report follows below, providing further detail and context for these comments.

This manuscript presents a model of interacting LLMs, aiming to simulate networks of online bots engaged in influence campaigns. The authors argue that such artificial societies exhibit many properties analogous to human opinion dynamics — including homophily, echo chambers, confirmation bias, and backfire effects. While the topic is timely and potentially impactful, the paper in its current form falls well short of the conceptual, methodological, and interpretive rigor expected of a Nature Communications publication. The manuscript is rhetorically ambitious but analytically underdeveloped. As it stands, the work risks overstating its contributions and misrepresenting the epistemic status of its findings.

Conceptual Framing and Contribution
The manuscript claims that the model represents online bot networks. However, this framing appears primarily as a rhetorical strategy to avoid engaging with two fundamental challenges in generative agent-based modeling (GABM): (1) demonstrating external validity and (2) offering a substantive social scientific contribution. The result is a model that is neither a convincing representation of bot networks nor a useful tool for social science research. Ultimately, it becomes yet another paper suggesting that LLMs exhibit "human-like" behavior, without offering the necessary theoretical or empirical work to make such claims meaningful.

What is the paper contributing to?
The GABM literature currently faces two central challenges: (1) understanding how such models can contribute to social science knowledge, and (2) demonstrating their external validity. These are important but difficult questions. This paper, however, largely sidesteps them by describing its model as a simulation of interacting LLM-based bots, and by suggesting

that understanding LLM opinion dynamics is essential for understanding coordinated influence networks. This move effectively allows the authors to claim that the model is a model of little more than itself, thereby bypassing the need to establish external validity.

This framing is difficult to justify. There is little reason to believe that bots designed for political influence would develop "opinions" by generating arguments and updating positions through deliberation. Designing such a system would be highly implausible as a real-world influence strategy.
Moreover, the paper's own analyses appear inconsistent with this framing. Much of the argumentation implicitly treats the model as simulating human behavior rather than bot activity. For example, the justification that "people develop opinions over time, so the model should too" is incompatible with a model intended to represent automated bot networks. This internal inconsistency undermines the clarity and credibility of the paper's contribution.

The manuscript further oscillates between denying that it models human behavior and implying that it does. It repeatedly invokes the notion of being "human-like," but does not provide the theoretical or empirical basis for making such a claim. Statements such as "these experiments provide guidance regarding how to directly intervene in the growing world of LLM agents operating in the wild" are particularly problematic, as they rely on a premise that is neither substantiated nor credible within the paper's framework.

Social Science Contribution
Even if one were to set aside the bot-network framing and treat the model as one of human behavior — and further set aside the requirement for external validity — the paper would still not constitute a meaningful contribution.

I am not a skeptic of generative agent-based models. They hold significant promise for advancing our understanding of social dynamics, despite their challenges around explainability and interpretability. However, this manuscript does not make use of that potential. Instead, it re-implements a well-known class of models in which agents accumulate arguments and become polarized, adding little beyond replacing transparent mechanisms with opaque LLM outputs. The primary result is a version of an established opinion dynamics model that is substantially less interpretable.

In terms of its treatment of opinion divergence ("polarization"), the model belongs to the argument-accumulation family (e.g., Mäs & Flache, 2013, JASSS). To make a substantive social science contribution, the paper would need to go beyond reproducing familiar dynamics and instead reveal new processes that can only be detected using LLM-based models.

The manuscript also engages with the social science literature in only a superficial way. Concepts such as polarization, homophily, and confirmation bias are treated as straightforward empirical regularities rather than contested theoretical constructs. This leads to a selective and often outdated use of theory. The "echo chamber" model of polarization — based on selective exposure, ideological extremity, and argument accumulation — has been widely criticized. For example:

1) Polarization is simplistically defined as movement away from neutrality, overlooking more recent work on affective and sectarian polarization and the role of identity.
2) The prevalence of echo chambers has been challenged by empirical research suggesting they are less common than once believed.
3) The argument-accumulation approach rests on a Habermasian model of politics that has been extensively critiqued (e.g., Achen & Bartels, Democracy for Realists).
If anything, the fact that the model reproduces such outdated theories may be interesting in itself - but it is questionable whether this should be described as "human-like."

Insights into LLM Behavior
The paper might still have offered value if it revealed unexpected aspects of LLM behavior. However, it does not. The reported results, such as homophilic clustering, are entirely predictable. They could be anticipated from existing models of opinion dynamics or simple network processes, combined with the known behavior of LLMs when prompted about social interaction. These patterns are particularly unsurprising given that LLMs are trained on corpora containing precisely these theoretical constructs and the literature that discusses them.

It is already well established that LLMs can mimic human-like responses. This finding is neither novel nor informative. Furthermore, issues such as training data leakage prevent such mimicry from being treated as evidence of emergent social mechanisms. Despite this, the manuscript frequently overstates its claims — for instance, describing LLMs' ability to generate network structures as "remarkable" when such structures can be produced by extremely simple models. Similarly, describing homophilic clustering as a "non-trivial phenomenon" is misleading; it is an expected outcome.

Minor Points
- The suggestion that "encouraging access and open-mindedness to diverse opinions" is an effective intervention is widely regarded as oversimplified and, in some contexts, misguided policy advice.
- Assertions about LLMs as a "new species" (Homo silicus), their "uncontrollable" effects, or their policy implications are highly speculative and unsupported by the presented evidence.
- The discussion of bias and the proposed "self-regulation" strategy is superficial. The leftward drift is unsurprising, and the intervention likely masks rather than addresses the problem. More fundamentally, if the model were intended to represent bot networks, it is unclear why such a correction would be relevant.
- Like many ABMs, the methodological design relies on arbitrary prompt engineering and simplistic opinion labels (e.g., a five-point left–right scale). It is unclear how these map onto real-world political cognition or how representative the resulting

dynamics are.
- The model relies heavily on LLM “self-reporting.” Treating such outputs as equivalent to human self-reports is questionable, given known limitations of LLM-generated explanations.

Summary
This manuscript addresses an important and emerging topic. However, it substantially overstates its contributions, under-theorizes key concepts, and relies heavily on superficial analogies between LLM outputs and human behavior. It is unconvincing in its claims, inconsistent in its framing, and insufficiently rigorous.

(Remarks on code availability)

Version 2:

Reviewer comments:

Reviewer #4

(Remarks to the Author)
I appreciate the authors' thorough and constructive response and revisions of their manuscript. The revisions have addressed my main concerns: the manuscript is now much more clearly framed as a synthetic LLM-agent testbed rather than as a model of real-world bot networks or human opinion dynamics. The authors also now acknowledge that the results align with established models rather than constituting a novel theoretical contribution, and they have added important caveats regarding validation, calibration, external validity, and interpretability.

There are however still a few remnants left of the previous framing that I would prefer seeing addressed. I would only suggest that the authors temper a few remaining formulations, especially in the abstract and introduction. Phrases such as "similarities between humans and LLM agents" and claims that the system can generate hypothetical strategies to mitigate polarization still risk implying a stronger correspondence to human social dynamics than the paper establishes. I would encourage the authors to consistently phrase these points as "patterns consistent with established opinion-dynamics models" and to avoid grounding the contribution in broad human–LLM similarity. With this minor textual adjustment, I would be comfortable recommending publication.

I would like to congratulate the authors on their paper, and thank them for their efforts in addressing my concerns.

(Remarks on code availability)

Open Access This Peer Review File is licensed under a Creative Commons Attribution 4.0 International License, which permits use, sharing, adaptation, distribution and reproduction in any medium or format, as long as you give appropriate

credit to the original author(s) and the source, provide a link to the Creative Commons license, and indicate if changes were made.
In cases where reviewers are anonymous, credit should be given to 'Anonymous Referee' and the source.
The images or other third party material in this Peer Review File are included in the article’s Creative Commons license, unless indicated otherwise in a credit line to the material. If material is not included in the article’s Creative Commons license and your intended use is not permitted by statutory regulation or exceeds the permitted use, you will need to obtain permission directly from the copyright holder.
To view a copy of this license, visit https://creativecommons.org/licenses/by/4.0/

Dear Editor and Reviewers,

We are deeply grateful for your engaged and constructive feedback, which has been instrumental in substantially strengthening our manuscript. Your thoughtful comments have guided us through a comprehensive revision process that has transformed the paper into a significantly more robust and impactful contribution. We believe these revisions have prepared the manuscript well for publication in *Nature Communications*.

Major Revisions and Enhancements:

In response to your valuable suggestions, we have implemented several major improvements that collectively represent a substantial transformation of the work. First, we have conducted extensive new experiments that significantly enhance the robustness of our proposed system as a theoretical testbed. These include investigations of model switching dynamics (where agents "defect" to rival LLMs mid-conversation), reasoning strategy interventions (seeding minorities of agents with chain-of-thought prompting), and social friction sensitivity analyses—all of which provide new insights into polarization mitigation strategies with direct real-world applicability.

Second, we have substantially strengthened our theoretical contributions by repositioning the work from isolated LLM interactions to networked autonomous agent societies. This paradigm shift, emphasized throughout the revised Abstract, Introduction, and Discussion, highlights how our framework opens new avenues for studying collective intelligence and human-AI interaction systems. We have also deepened our analysis of the underlying psychological processes driving opinion formation, providing detailed characterization of emergent mechanisms like confirmation bias and motivated reasoning that arise naturally from agent interactions.

Third, we have significantly improved the manuscript's clarity and scope. We have reorganized key sections for better flow, refined our terminology (replacing subjective labels like "alarming issues" with more objective framing), enhanced our engagement with recent literature, and provided more comprehensive methodological details. We have also expanded our analysis to additional political topics and explicitly discussed the limitations and applicability boundaries of our synthetic simulation approach.

These revisions collectively address all major concerns raised by the reviewers while substantially expanding the work's theoretical depth, empirical rigor, and practical relevance. We provide detailed point-by-point responses below with responses in blue and revisions in red.

We also attach:

- A revised manuscript with changes marked in blue, and
- A revised Supplementary Information with changes marked in blue.

Reviewer #1 (Remarks to the Author):

The manuscript is progressing in a positive direction. The additional robustness checks, clearer methodological exposition, and newly introduced intervention experiments make the paper markedly more transparent and empirically rigorous than the initial draft. Moreover, the integration of the rapidly evolving literature and the articulation of practical implications demonstrate commendable responsiveness to earlier feedback.

Response: We sincerely thank the Reviewer for this thoughtful and encouraging feedback. We appreciate the recognition of our efforts to improve methodological clarity and empirical rigor, strengthen our engagement with the rapidly evolving literature, and better articulate practical implications. In this revision cycle, we follow Reviewer suggestions and introduce a series of additional experiments, which further strengthen the proposed system as "a robust testbed for theories" and help "open fertile avenues for further inquiry," as the Reviewer insightfully point out.

Nevertheless, the revision now references Tessler, Chang, and other recent studies while framing its contribution as permitting the co-evolution of edges and opinions within an autonomous agent society. Yet the principal empirical finding "LLM agents drifting into echo chambers" largely mirrors results already documented in those studies and in decades of social-influence modelling. Without a sharper theoretical claim or an unforeseen mechanism that earlier work could not have anticipated, the paper presently appears more a scaled-up replication enhanced by polished visualisations than a step change in understanding.

That said, the new framework opens fertile avenues for further inquiry. For example, what occurs if agents can "defect" to a rival language model mid-conversation, thereby altering not only their social ties but also their cognitive architecture? Might seeding a minority of agents with a markedly different reasoning strategy (e.g., chain-of-thought prompting) temper or amplify polarisation? Finally, how sensitive are the results to the cost of severing ties, does even minimal friction slow the formation of ideological silos? Addressing these questions could convert the platform into a robust test bed for theories that extend beyond the current version.

Response: We thank the Reviewer for recognizing the value of our proposed system as a testbed for theories, and for providing constructive suggestions that have helped us further improve its robustness. We agree that the empirical phenomena observed in our work corroborate long-established findings in social-influence modeling, further confirming our empirical basis. They also echo recent studies on LLM agents, underscoring the potential of these agents to shed light on long-standing challenges in political contexts. At the same time, our proposed system, as an autonomous agent society powered by LLMs, is not merely a replication of human social phenomena. As the Reviewer insightfully points out, it opens fertile avenues for further inquiry. We see it as a valuable testbed for exploring collective dynamics and intervention strategies prior to

real-world experiments, and for generating new insights into human societies. To further enhance its robustness, we followed these constructive suggestions and conducted experiments on **(i) model switching**, where agents “defect” to a rival LLM mid-conversation; **(ii) reasoning strategy enhancement**, where a minority of agents adopt chain-of-thought prompting; and **(iii) severing cost sensitivity**, where minimal social frictions are introduced to examine their impact on the formation of ideological silos. In the following sections, we provide a detailed discussion of these three experiments and the new insights that an autonomous agent society can offer to the real world.

(i) As a first step, we examine the intriguing scenario in which agents can “defect” to a rival language model mid-conversation. Specifically, we replace the underlying LLMs driving agents from the original GPT-3.5 to the more advanced GPT-4o at the midpoint of simulation, allowing them to adopt a more sophisticated cognitive architecture. As shown in Figures R1 and R2, we find that this replacement has only marginal effects on the polarization of agents’ collective opinions: agents still converge to similarly polarized states. Moreover, their social network likewise splits into two communities holding opposing opinions, suggesting that an enhanced cognitive architecture mid-conversation does not prevent the formation of ideological silos. To further test this, we replace the agents’ LLM with a substantially different alternative, DeepSeek-V3, which is developed by a Chinese company and trained on a different corpus (Figure R3). Even with this more distinct model, the outcome remains largely the same: polarization and ideological silos persist.

Overall, these findings suggest that mid-conversation defection to a rival language model may slightly influence the evolution trajectories of agents’ collective opinions and network structures, but it does not fundamentally disrupt the resulting polarization or the formation of ideological silos. A key reason, we believe, lies in the *path dependence* of these dynamics. By the midpoint of the simulation, agents have already developed social networks and collective opinions that reinforce one another’s polarizing tendencies. Their networks have begun to crystallize into two distinct communities, and their opinions are shaped not only by the underlying LLMs, but also by the accumulated history of interactions and surrounding social context. This reflects well-established findings in political psychology, where timing and early exposure are critical to opinion formation and change [1-3]. As such, merely replacing the underlying LLMs, without addressing the entrenched social and psychological drivers of polarization, proves insufficient to reverse these emergent polarization dynamics.

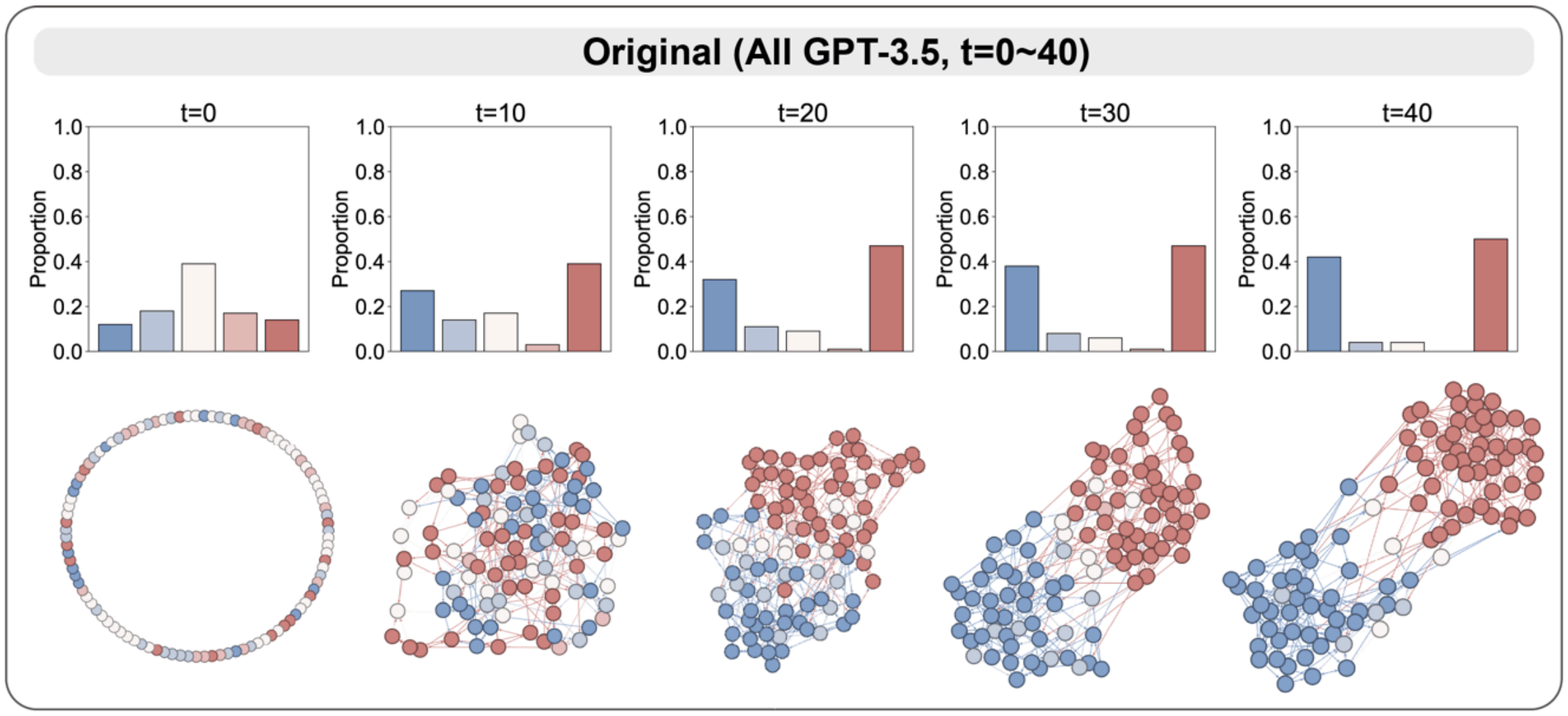

Fig. R1. Evolution of LLM agents' collective opinions and network structures, where all agents are driven by GPT-3.5 for the entire duration of the simulation (consistent with Main Text), and they discuss the political issue of abortion bans.

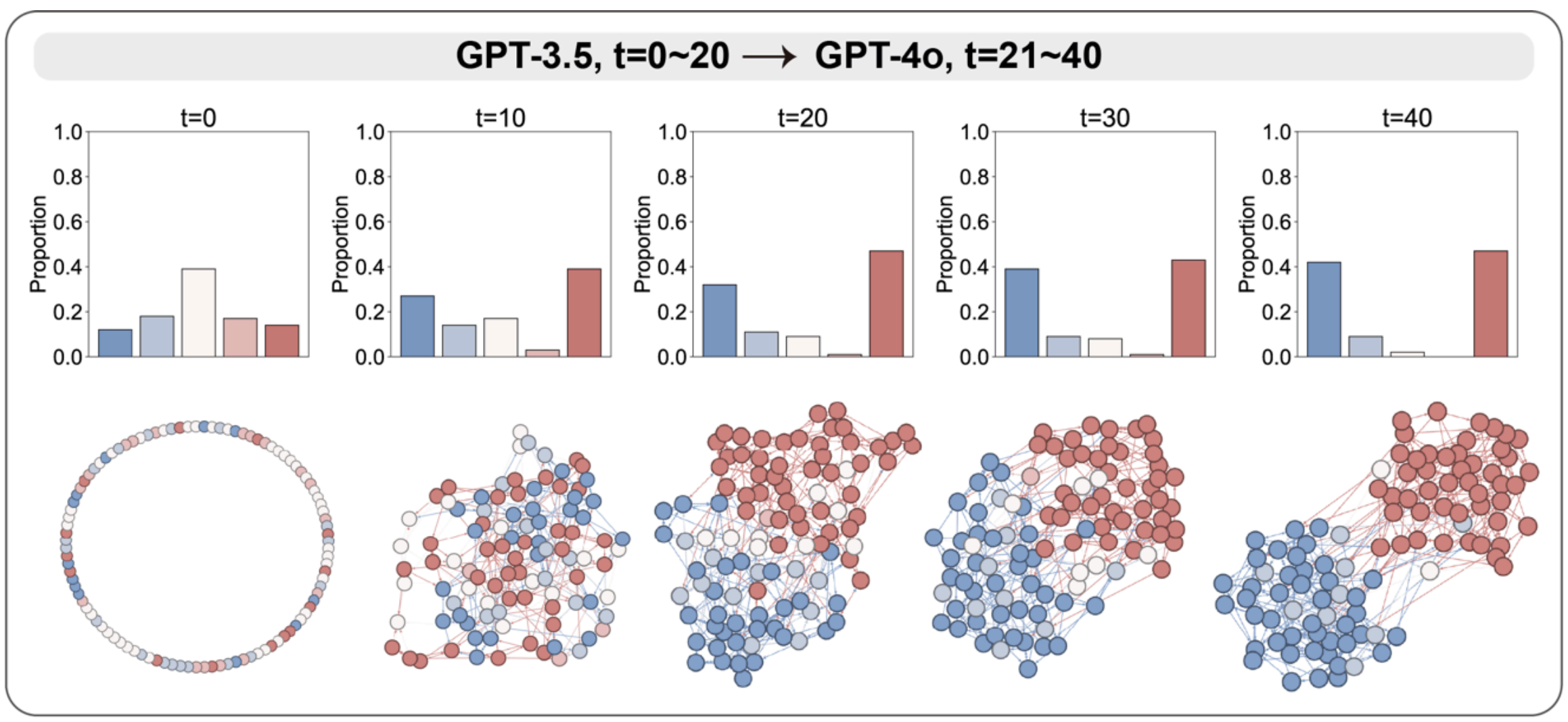

Fig. R2. Evolution of LLM agents' collective opinions and network structures, where agents are initially driven by GPT-3.5 but defect to GPT-4o amid conversations about the political issue of abortion bans.

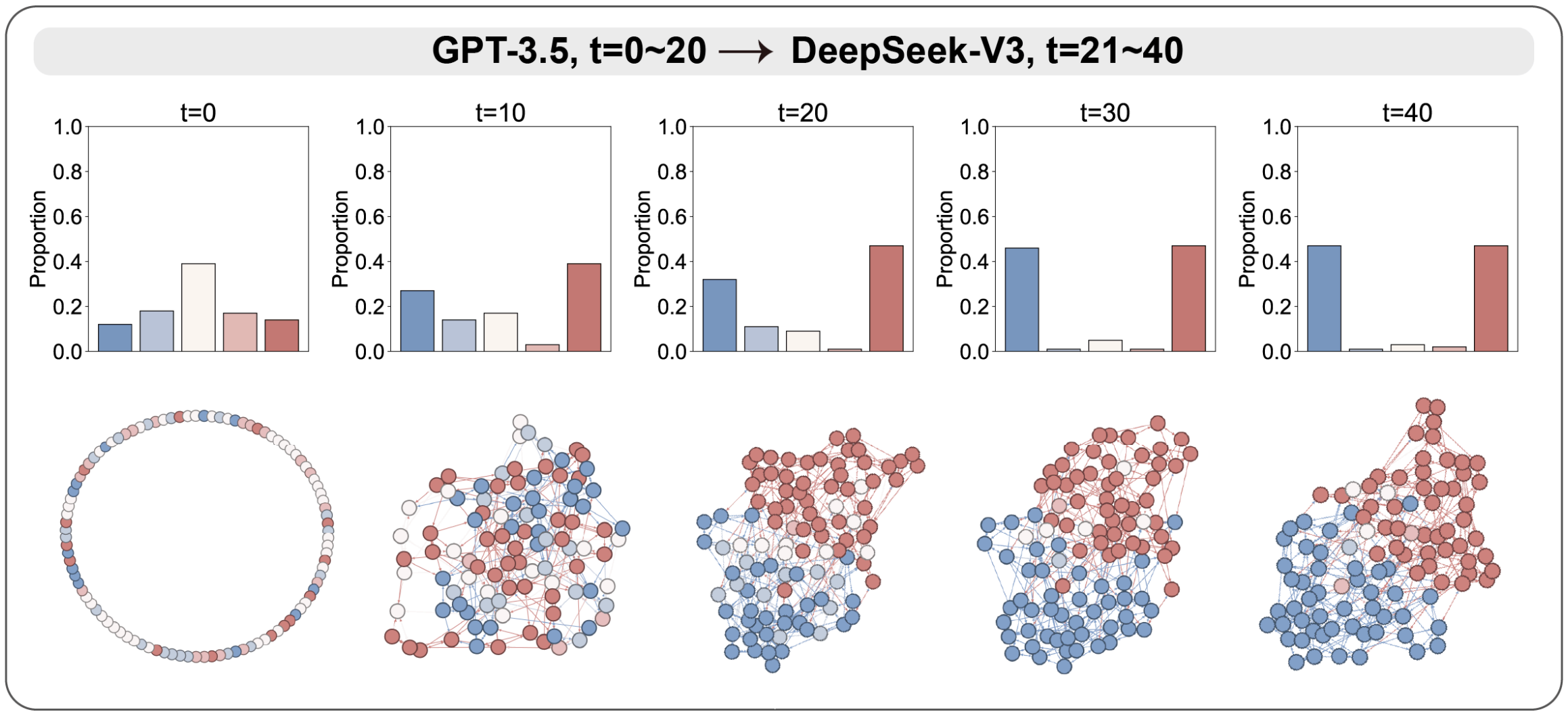

Fig. R3. Evolution of LLM agents' collective opinions and network structures, where agents are initially driven by GPT-3.5 but defect to DeepSeek-V3 amid conversations about the political issue of abortion bans.

In the revised manuscript, we share all of these results within Section 3.5 of Supplementary Information (Pages 20-23) and discuss them in the Discussion section of the Main Text (Page 6).

[1] Druckman, James N., Jordan Fein, and Thomas J. Leeper. "A source of bias in public opinion stability." *American Political Science Review* 106.2 (2012): 430-454.
[2] Nickerson, Raymond S. "Confirmation bias: A ubiquitous phenomenon in many guises." *Review of general psychology* 2.2 (1998): 175-220.
[3] Lord, Charles G., Lee Ross, and Mark R. Lepper. "Biased assimilation and attitude polarization: The effects of prior theories on subsequently considered evidence." *Journal of personality and social psychology* 37.11 (1979): 2098

Revision: [Discussion in Main Text, Page 6]…While our main results are based on OpenAI's ChatGPT and three political issues, we also perform extensive experiments with different LLMs (**both full-run and midway-defection settings**, SI Section 3.5) as well as on topics of immigration restriction and the flat Earth theory (SI Section 3.12), finding that these experiments consistently support our findings…

[Section 3.5 in Supplementary Information, Pages 20-23] Beyond comparing simulations entirely driven by different LLMs, we further explore a more dynamic setting in which agents defect to a rival language model mid-conversation. Specifically, at the midpoint of simulation, we change the underlying model from GPT-3.5-turbo to the more advanced GPT-4o, allowing agents to adopt a more sophisticated cognitive architecture. As shown in Figures S19 and S20, this replacement produces only marginal changes: agents still converge to similarly polarized opinion states, and their social networks likewise split into two communities holding opposing views. This suggests that enhancing the agents' cognitive architecture mid-conversation does not prevent the formation of ideological silos. We further test a more extreme case by replacing GPT-3.5 with a substantially

different rival language model, DeepSeek-V3, developed by a Chinese company and trained on a distinct corpus (Figure S21). Even under this replacement, however, results remain similar: neither the level of polarization nor the formation of ideological silos is meaningfully altered.

Overall, these findings suggest that mid-conversation defection to a rival language model may slightly influence the evolution trajectories of agents' collective opinions and network structures, but it does not fundamentally disrupt the resulting polarization or the formation of ideological silos. A key reason, we believe, lies in the path dependence of these dynamics. By the midpoint of simulation, agents have already developed social networks and collective opinions that reinforce one another's polarizing tendencies. Their networks have begun to crystallize into two distinct communities, and their opinions are shaped not only by the underlying LLMs, but also by the accumulated history of interactions and surrounding social context. This reflects well-established findings in political psychology, where timing and early exposure are critical to opinion formation and change [11, 29, 25]. As such, merely replacing the underlying LLMs, without addressing the entrenched social and psychological drivers of polarization, proves insufficient to reverse emergent polarization dynamics.

(ii) Building on this, we further explore the Reviewer's second question of whether seeding a minority of agents with a markedly different reasoning strategy can temper or amplify polarization. Specifically, we equip 10% of all agents with chain-of-thought (CoT) prompting, enabling them to think step by step as they establish social relationships, communicate, and form opinions on political issues. These CoT agents are seeded at the beginning of the simulation and randomly distributed across the network. We ensure that the proportion of CoT agents across different opinions aligns with the overall initialization distribution.

Comparing the evolution of the original system (Figure R1) with that of the system seeded with 10% CoT agents (Figures R4 and R5), we observe a substantial shift: the proportion of agents holding moderate or neutral opinions increases from 8% to 38% (Figure R4), and the overall polarization level drops by 15% (Figure R5a). Meanwhile, the network-level division between agents with opposing opinions is largely alleviated, with final modularity scores declining from 0.43 to 0.25 (Figure R5b; a modularity score above 0.3 typically marks clear community structure). Overall, these observations suggest that even a small minority of agents, equipped with a markedly different reasoning strategy and seeded into the system, can effectively temper polarization. This highlights the critical role of "wiser" or more thoughtful individuals in fostering more constructive and less divisive political communication, and suggests a promising intervention strategy for mitigating polarization.

In the revised version, we have incorporated all of these results in Sections 3.5 and 3.9 of the Supplementary Information (Pages 20-23 and 43) and further discussed their potential as a mitigation strategy in the Discussion section of the Main Text (Page 7). For your

convenience, we have included the corresponding revisions below, with key changes highlighted in bold.

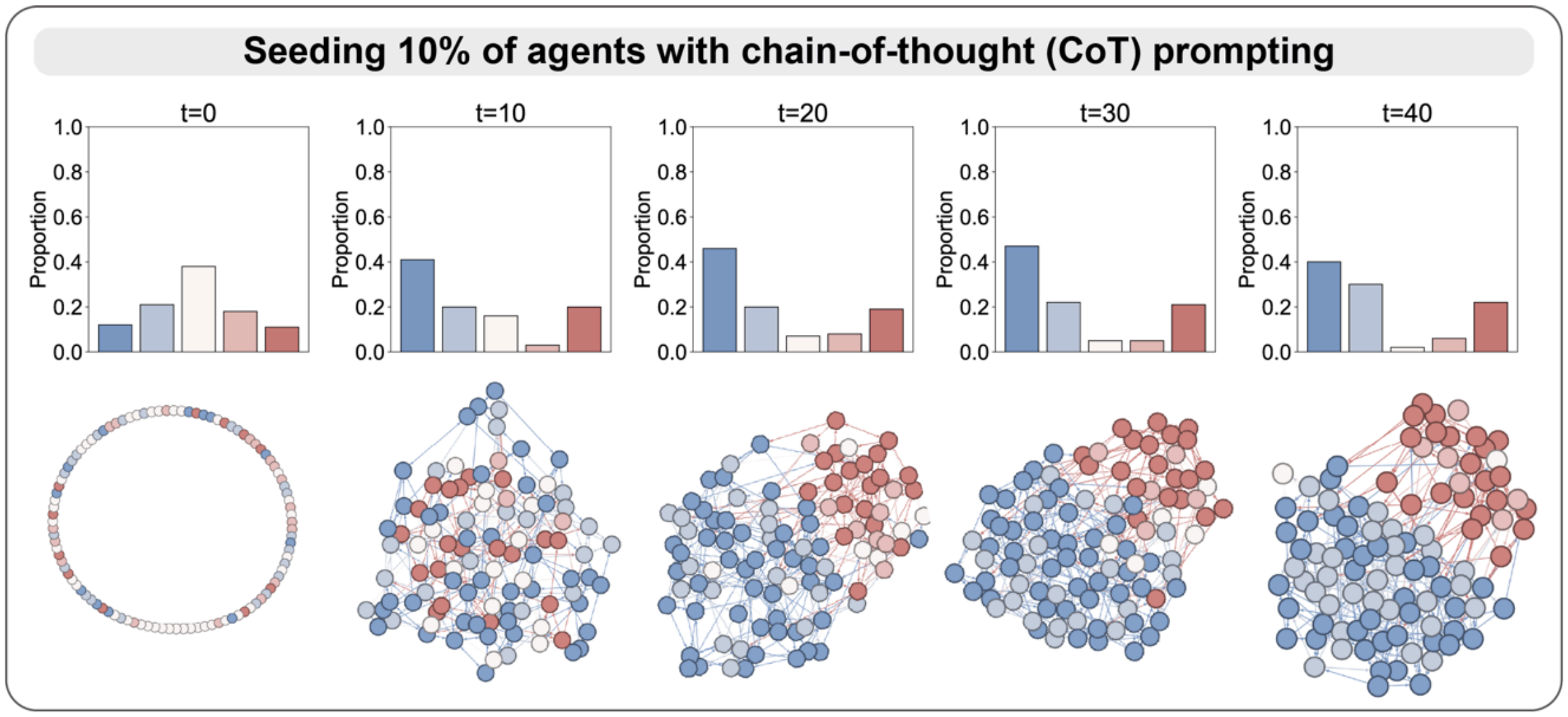

Fig. R4. Evolution of LLM agents' collective opinions and network structures, where we seed 10% of agents with chain-of-thought (CoT) prompting. All agents are driven by GPT-3.5 and they discuss the political issue of abortion bans.

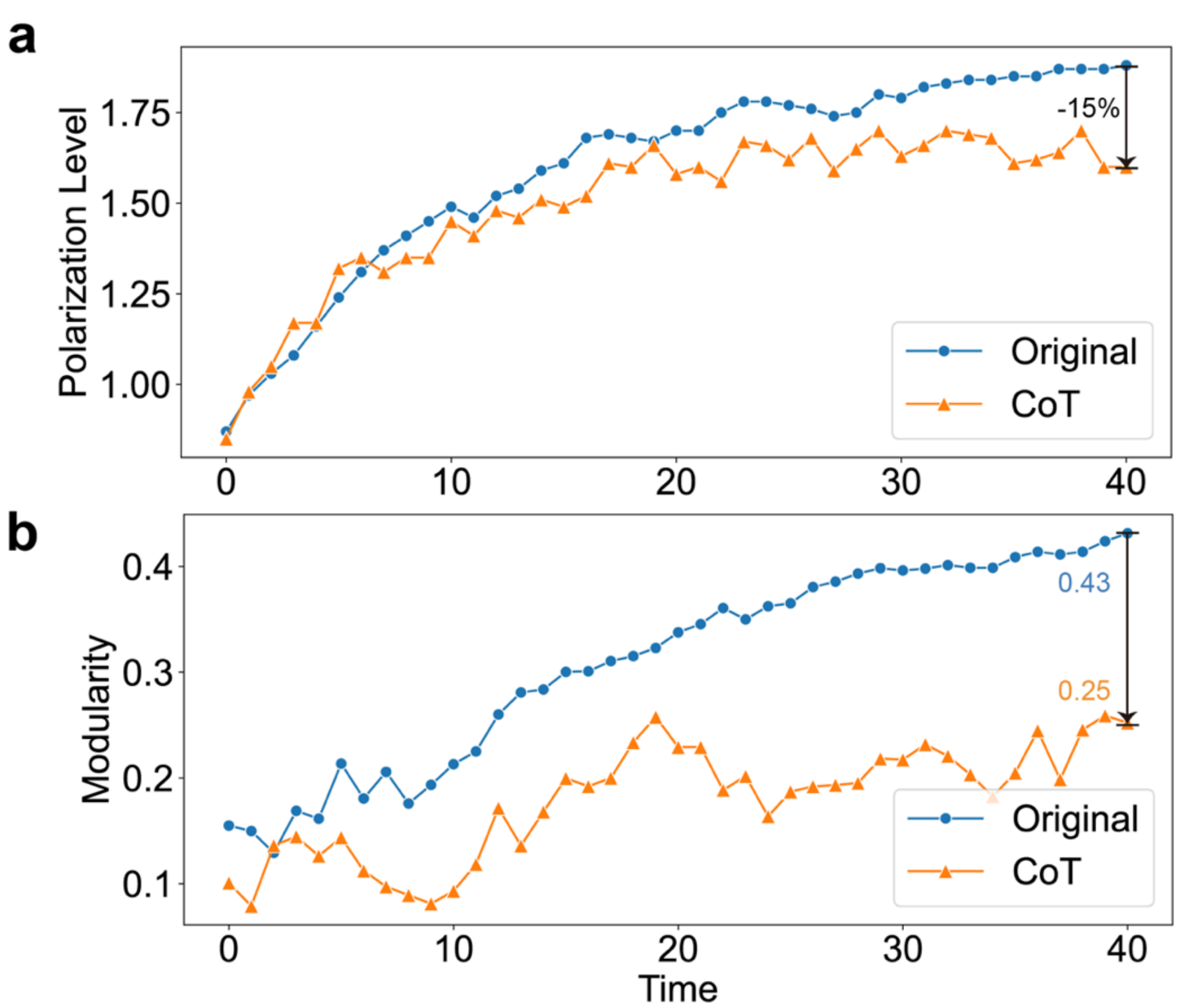

Fig. R5. Comparison between the original system and a system where 10% of agents are seeded with CoT prompting, in terms of (a) polarization level and (b) network modularity.

[1] Chang, Serina, et al. "Llms generate structurally realistic social networks but overestimate political homophily." *Proceedings of the International AAAI Conference on Web and Social Media*. Vol. 19. 2025.
[2] Gao, Chen, et al. "S3: Social-network simulation system with large language model-empowered agents." *arXiv preprint arXiv:2307.14984* (2023).
[3] Papachristou, Marios, and Yuan Yuan. "Network formation and dynamics among multi-llms." *arXiv preprint arXiv:2402.10659* (2024).
[4] De Marzo, Giordano, Luciano Pietronero, and David Garcia. "Emergence of scale-free networks in social interactions among large language models." *arXiv preprint arXiv:2312.06619* (2023).
[5] Cinelli, Matteo, et al. "The echo chamber effect on social media." *Proceedings of the national academy of sciences* 118.9 (2021): e2023301118.
[6] Bakshy, Eytan, Solomon Messing, and Lada A. Adamic. "Exposure to ideologically diverse news and opinion on Facebook." *Science* 348.6239 (2015): 1130-1132.
[7] Flamino, James, et al. "Political polarization of news media and influencers on Twitter in the 2016 and 2020 US presidential elections." *Nature Human Behaviour* 7.6 (2023): 904-916.

Revision: [Discussion in Main Text, Page 7] …Furthermore, the proposed networked system of LLM agents, which systematically shares multi-scale similarities with human networks, provides valuable grounds for piloting complex experiments. Due to costs, logistics, and ethical considerations, large-scale real-world experiments are not always practical [98]. Pre-experimental technologies that reduce the explosively large design space of social experiments identify promising directions and eliminate impractical strategies, enabling us to learn more. In this work, taking the proposed networked system of LLM agents as the pre-experimental ground, we identify several strategies for reducing polarization and promoting less divisive political conversations. These strategies have strong real-world foundations and can be readily transferred to human communication. For example, the intervention of neutral elite signaling (Figure 4f) could be implemented by deploying neutral, authoritative bots to serve as trusted messengers of balanced content. **Moreover, the proposed system not only enables the direct adaptation of more sophisticated intervention designs from real-world human studies [89] (SI Section 3.6), but also serves as a robust testbed for theoretical exploration, facilitating the discovery of additional mitigation strategies, such as introducing "wiser" or more thoughtful individuals (SI Section 3.9) and incorporating minimal social friction (SI Section 3.10)…**

[Section 3.9 in Supplementary Information, Page 43] We first explore whether seeding a minority of agents with a markedly different reasoning strategy can temper or amplify polarization. Specifically, we equip 10% of all agents with chain-of-thought (CoT) prompting, enabling them to think step by step as they establish social relationships, communicate, and form opinions on political issues. These CoT agents are seeded at the beginning of the simulation and randomly distributed across the network. The proportion of CoT agents across different opinions aligns with the overall initialization distribution.

Comparing the evolution of the original system with that of the system seeded with 10% CoT agents (Figures S41 and S42), we observe a substantial shift: the proportion of agents holding moderate or neutral opinions increases from 8% to 38% (Figure S41), and the overall polarization level drops by 15% (Figure S42a). Meanwhile, the network-level division between agents with opposing opinions is largely alleviated, with final modularity scores declining from 0.43 to 0.25 (Figure S42b; a modularity score above 0.3 typically marks clear community structures). Overall, these observations suggest that even a small minority of agents equipped with a markedly different reasoning strategy and seeded into the system can effectively temper polarization. This highlights the critical role of "wiser" or more thoughtful individuals in fostering more constructive and less divisive political communication.

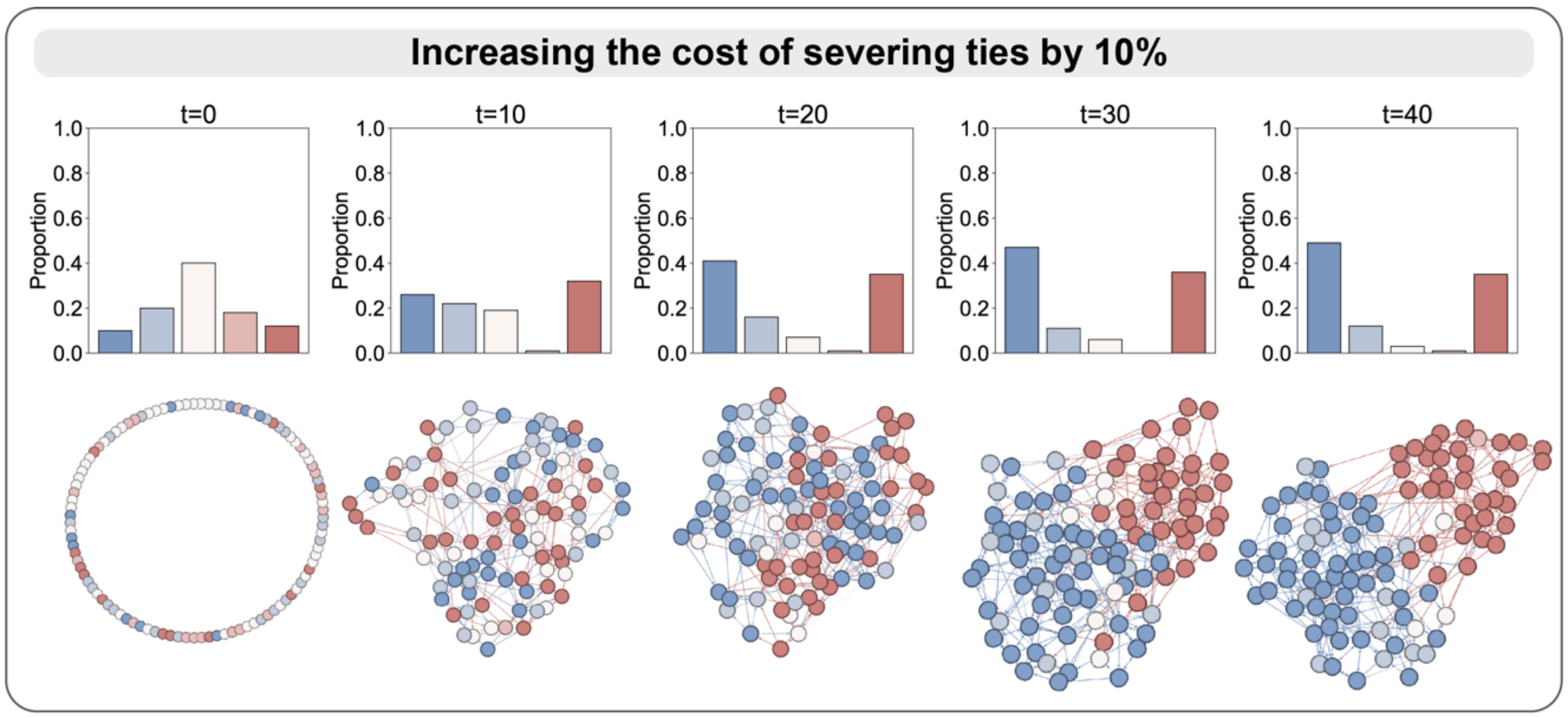

Fig. R6. Evolution of LLM agents' collective opinions and network structures, where we increase the cost of severing ties by 10%. All agents are driven by GPT-3.5 and they discuss the political issue of abortion bans.

Finally, we turn to the Reviewer's third question concerning the sensitivity of results to the cost of severing social ties. To explore whether even minimal friction can slow the formation of ideological silos, we introduce a simple mechanism, where agents' decisions to sever existing social ties are blocked with a fixed probability (e.g., 10%), simulating the existence of minimal social friction. Figures R6 and R7 show the results under a minimal friction condition, where the cost of severing ties is increased by only 10%. Notably, we find that while the minimal friction leads to marginal mitigation in polarization level (Figure R7a), its effect on suppressing the formation of ideological silos is substantial, as consistently reflected across four different network-level metrics (Figures R7b-e). This experiment suggests that even minimal social friction can play a nontrivial role in slowing the formation of ideological silos and fostering communication between opposing camps. Furthermore, this finding has direct implications for human societies: it suggests that introducing subtle, unobtrusive forms of friction may offer a simple yet effective way to encourage communication between individuals with diverse or even opposing opinions. In our revision, we incorporate all results into Section 3.10 of the Supplementary Information (Page 43), and discuss the potential of minimal social

friction as a promising strategy for mitigating polarization in the Discussion section of the Main Text (Page 7). For your convenience, we include the corresponding revisions below.

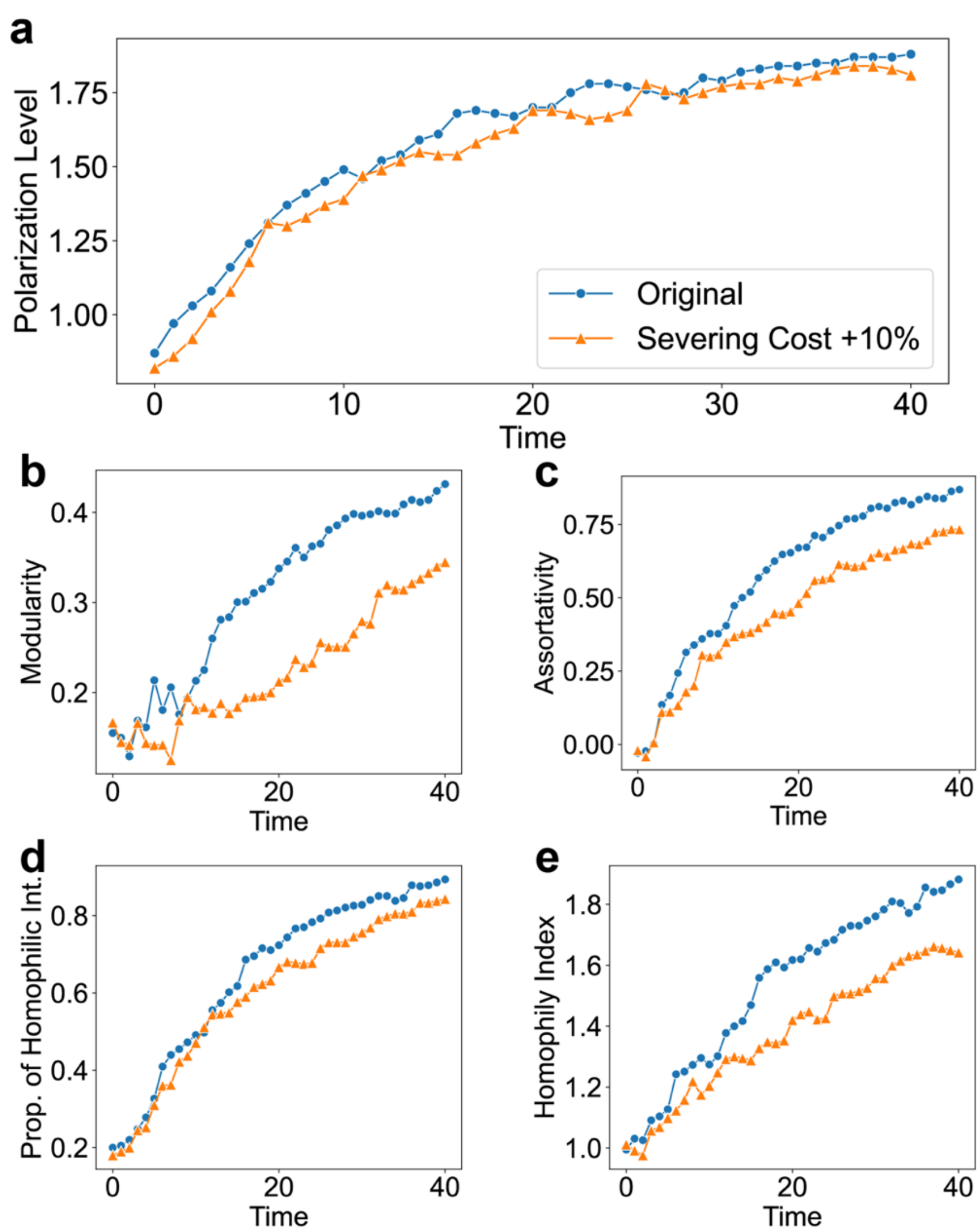

Fig. R7. Comparison between the original system and a system where the cost of severing ties is increased by 10%, in terms of (a) polarization level, (b) network modularity, (c) assortativity, (d) proportion of homophilic interactions, and (e)homophily index.

Revision: [Discussion in Main Text, Page 7] …Furthermore, the proposed networked system of LLM agents, which systematically shares multi-scale similarities with human networks, provides valuable grounds for piloting complex experiments. Due to costs, logistics, and ethical considerations, large-scale real-world experiments are not always practical [98]. Pre-experimental technologies that reduce the explosively large design space of social experiments identify promising directions and eliminate impractical strategies, enabling us to learn more. In this work, taking the proposed networked system

of LLM agents as the pre-experimental ground, we identify several strategies for reducing polarization and promoting less divisive political conversations. These strategies have strong real-world foundations and can be readily transferred to human communication. For example, the intervention of neutral elite signaling (Figure 4f) could be implemented by deploying neutral, authoritative bots to serve as trusted messengers of balanced content. **Moreover, the proposed system not only enables the direct adaptation of more sophisticated intervention designs from real-world human studies [89] (SI Section 3.6), but also serves as a robust testbed for theoretical exploration, facilitating the discovery of additional mitigation strategies, such as introducing "wiser" or more thoughtful individuals (SI Section 3.9) and incorporating minimal social friction (SI Section 3.10)…**

[Section 3.10 in Supplementary Information, Page 43] We examine the sensitivity of our results to the cost of severing social ties. To explore whether even minimal friction can slow the formation of ideological silos, we introduce a simple mechanism in which agents' decisions to sever existing social ties are blocked with a fixed probability (e.g., 10%), simulating minimal social friction. Figures S44 and S45 show the results under this minimal friction condition, where the cost of severing ties is increased by only 10%. Notably, while the minimal friction leads to marginal mitigation in polarization levels (Figure S45a), its effect on suppressing the formation of ideological silos is substantial, as consistently reflected across four different network-level metrics (Figures S45b–e). This experiment suggests that even minimal social friction can play a nontrivial role in slowing the formation of ideological silos and fostering communication between opposing camps. Furthermore, this finding has direct implications for human societies: introducing subtle, unobtrusive forms of friction may offer a simple yet effective way to encourage communication between individuals with diverse, or even opposing, opinions.

Overall, we would like to thank the Reviewer once again for insightful and constructive questions. By conducting extensive experiments, we have thoroughly examined scenarios where agents can defect to rival LLMs (Figures R1-R3), adopt reasoning strategies (Figures R4-R5), and interact under minimal social frictions when severing ties (Figures R6-R7). These experiments help us substantially enhance the robustness of our proposed system as a testbed for theoretical exploration. **More importantly, the findings yield several new insights into the mitigation of polarization and ideological silos in human societies. First**, we find that once polarization takes root through the co-evolution of opinions and social networks, later interventions are often insufficient to reverse the emergent dynamics unless they directly address their entrenched social and psychological drivers. **Second**, compared to late-stage efforts, earlier intervention by seeding even a small minority of "wiser" or more thoughtful individuals (represented by agents equipped with CoT prompting in agent societies) can substantially temper opinion polarization. **Third**, introducing minimal social friction can effectively slow the formation of ideological silos. Together, these insights further confirm the value of our proposed system as a testbed, not only for theoretical exploration but also for informing real-world policy and practice.

Reviewer #2 (Remarks to the Author):

I was reviewer 2 on the NHB submission. I am extremely impressed with the efforts undertaken by the authors. While I am not able to fully appreciate the technical contributions, I appreciate the clarity of technical writing. I also am now much clearer on the substantive implications. It is now apparent how the infrastructure can be used to evaluate human behavior and interventions therein in essence an extremely sophisticated simulation. One question I have is whether there is a secondary contribution. I was intrigued by the authors point that most work on LLM-human interactions involve unitary LLMs. Is it interesting that one could now explore that interaction with networked LLMs?

Response: We sincerely thank the Reviewer for this positive and encouraging feedback. In previous revision cycle, your deep expertise greatly helped us articulate both the technical and broader social-scientific contributions of our work, and communicate them more clearly to an interdisciplinary audience. We are pleased to see your recognition of the improved clarity and the substantive implications of our work, especially its capability to evaluate human behavior and interventions through LLM-empowered simulation.

We also thank the Reviewer for raising the insightful point about a potential secondary contribution. We agree that the shift from unitary to networked LLMs opens up a rich and underexplored direction. Just as the rise of network analysis transformed the study of human behavior from isolated individuals to relational structures and collective dynamics over the past decades [1], **our introduction of networks among LLM agents is not merely a technical enhancement, but an essential factor in shaping the system's emergent dynamics**. For example, networked interactions among LLM agents give rise to non-trivial emergent phenomena such as spontaneous homophilic clustering and the division of opposing communities (Figures 3a&b in the Main Text), which do not emerge in isolated-agent settings. Furthermore, when we degrade the mechanisms of network evolution from agents' self-organization to static or random topologies (Figure S14 in Supplementary Information), we observe the disruption of human-like polarization. These findings consistently underscore the essential role of **networks** in shaping LLM agents' collective behavior.

This shift also opens new avenues for exploring human–AI interactions, treating collections of LLM agents as artificial societies rather than isolated conversational bots. For example, instead of relying on a single LLM mediator [2-3], future systems could deploy a networked layer of LLM agents with diverse personas to serve as impartial and resilient intermediaries between individuals with opposing views. This artificial layer could absorb hostility, bridge fragmented communities, and foster more constructive deliberation. Overall, we believe this shift marks an important step toward a new paradigm for studying collective intelligence, with implications for both computational social science and the design of future human–AI interaction systems. In the revised version, we have further emphasized this secondary contribution in the revised version,

including updates to the Abstract, Introduction, and Discussion sections of the Main Text (Pages 1–2 and 6). For your convenience, we provide the corresponding revisions below.

[1] Lazer, David, et al. "Computational social science." *Science* 323.5915 (2009): 721-723.
[2] Tessler, Michael Henry, et al. "AI can help humans find common ground in democratic deliberation." *Science* 386.6719 (2024): eadq2852.
[3] Argyle, Lisa P., et al. "Leveraging AI for democratic discourse: Chat interventions can improve online political conversations at scale." *Proceedings of the National Academy of Sciences* 120.41 (2023): e2311627120.

Revision: [Abstract in Main Text, Page 1] We discover that these agents spontaneously develop their own social network with human-like properties, including homophilic clustering. These LLM agents shape their collective opinions through mechanisms observed in the real world, including the echo chamber effect. **Similarities between humans and LLM agents — encompassing behaviours, mechanisms, and emergent phenomena — not only offer a valuable testbed for identifying plausible strategies to mitigate polarization and its consequences, but also point toward a shift in the human–LLM interaction paradigm from unitary agents to networked autonomous systems.**

[Introduction in Main Text, Pages 1-2] Unfortunately, despite the urgency highlighted by these examples, we remain far from being able to answer this question. This is largely because of two key gaps in our current understanding: (i) Unlike traditional rule-based bots, LLM agents operate in human languages generated by black-box models with billions to trillions of parameters, hindering our understanding of the underlying mechanisms they follow when forming opinions. (ii) Interactions enable them to evolve, gradually developing their own social network and collective opinions [34]. This bottom-up process, growing from microscopic interactions to macroscopic social order, makes it difficult to predict LLM agents' collective opinions and behaviours. **Taken together, addressing these two gaps begins with a crucial first step: understanding the opinion dynamics of LLM agents, both as individuals with human-like intelligence and, more importantly, as evolving artificial collectives.**

…In this work, we simulate a networked system, where thousands of agents, solely driven by LLMs, freely establish social relationships, communicate, and form opinions on political issues. We discover that these free-form social interactions among LLM agents result in the emergence of opinion polarization, a phenomenon widely observed in human societies [30, 49–54]. Meanwhile, LLM agents spontaneously organize their own social network of human-like properties: agents with homophilic opinions tend to cluster, while those with opposing opinions tend to avoid interactions [34]. The self-organized networks that result, in turn, shape their collective opinions through network-level mechanisms of the echo chamber and backfire effects, initially identified in human social networks [49, 51, 53, 55]. **These non-trivial phenomena, such as polarization and homophilic clustering, underscore how networked interactions transform otherwise isolated LLMs into a collectively evolving system with emergent dynamics.** Shifting

the focus from network organization to individual behaviours, we further examine a wide range of social mechanisms contributing to real-world polarization, e.g., selective exposure [56], confirmation bias [57], and elite signaling [30], finding their effects on LLM agents align with those observed in human societies. This suggests that the emergence of human-like polarization among LLM agents is not a coincidence. Rather, it originates from the systematic similarity between humans and LLM agents, encompassing the social networks they self-organize, the collective opinions they develop, and the mechanisms through which these processes unfold. Observed similarities between human and AI collectives suggest that the system we propose can constitute a valuable pre-experimental ground for exploring effective strategies to reduce polarization and promote more inclusive political conversations [36]. Through extensive intervention experiments based on our system, we find that in a highly polarized and nearly crystallized social network, directly modifying network structures has limited effect on reducing polarization. Instead, encouraging access and open-mindedness to diverse opinions at the individual level proves more effective. **Overall, this work not only sheds light on subtle opinion dynamics and collective behaviour of LLM agents from a new network perspective, but it also unveils their potential to assist social scientists and policymakers in experiments and policy design [58].**

[Discussion in Main Text, Page 6] This work uncovers that LLM agents, sometimes considered a new species "Homo silicus" [12], exhibit human-like opinion polarization on political issues, both individually and collectively. Specifically, from the individual perspective, opinion formation among LLM agents follows human-like social and psychological mechanisms [56, 57], e.g., selective exposure, confirmation bias, elite signaling, etc. From the collective perspective, LLM agents organically develop their social networks with human-like properties, e.g., homophilic clustering and scale-free degree distributions [34]. Moreover, their self-developed social networks, in turn, shape LLM collective opinions in human-like ways, i.e., through echo chamber and backfire effects, resulting in the emergence of human-like polarization [30, 49–53, 87, 90, 91]. Notably, all these psychological and social mechanisms, network organization, and collective opinions naturally emerge from interactions among agents completely driven by LLMs. **Building on these findings, our work shifts the perspective from isolated LLMs [35, 36, 38, 41–44] to networked LLM agent societies, offering a new paradigm for understanding emergent collective dynamics in artificial agents and their potential interactions with human societies.** While our main results are based on OpenAI's ChatGPT and three political issues, we also perform extensive experiments with different LLMs (both full-run and midway-defection settings, SI Section 3.5), as well as on topics of immigration restriction and the flat Earth theory (SI Section 3.12), finding that these experiments consistently support our core findings…

I appreciate the incorporation of some of the literature I had mentioned and how the authors' work aligns with that work (while also ostensibly being dependent on how parameter values are set). I have a few questions. First, the addition of two more issues is useful. I am a little confused by the labeling "alarming" issues, what exactly does this mean and how is it evaluated? Is it just polarized issues? I ask

because right now in the US, immigration restrictions are very alarming but in the paper this is treated as not an alarming issue. This is largely a semantic question.

Response: We thank the Review for this thoughtful comment and completely agree that the labeling of issues as "alarming" was both semantically imprecise and potentially distracting if not misleading. Our original intention was to distinguish issues that tend to provoke strong moral stakes and immediate personal consequences, such as abortion bans, gun control, and partisan alignment, from those that, while politically significant, are often perceived as involving less direct threat to life or bodily autonomy, such as immigration restrictions.

We acknowledge, however, that this distinction is subjective and context dependent, which the Reviewer's example makes particularly clear. In light of this, we have revised the manuscript to replace the term "alarming" with the more descriptive term "critical," which better reflects our intent to focus on substantively important and socially consequential issues without making implicit value judgments. We also agree that immigration restrictions are a critical issue and have revised the relevant descriptions accordingly in the manuscript.

Revision: [Results in Main Text, Pages 2-3] To explore the opinion dynamics of LLM agents, we simulate a networked system based on these LLM agents (Figure 1a). **We focus predominantly on the three critical political issues** – partisan alignment, gun control, and abortion ban – where concerns over opinion polarization have persisted for decades [49, 52, 55].

[Discussion in Main Text, Page 6] While our main results are based on Open AI's ChatGPT (GPT-3.5) and **three political issues**, we also perform extensive experiments across different LLMs (SI Section 3.5), as well as the topic of immigration restriction and the flat Earth theory (SI Section 3.12), finding that these experiments consistently support our findings…

[Section 3.12 in Supplementary Information, Page 49] To test the system's generalizability across different types of issues, we conduct two additional experiments beyond the political issues previously studied. The first relates to immigration restrictions, a socially relevant and critical issue. The second is the flat Earth theory, a non-political, fact-based issue with clear scientific consensus. These additions help demonstrate the generalizability of the proposed system and its corresponding results across both high-stakes political topics and more neutral or non-controversial domains.

Second, I enjoyed the introduction, but it leads to the expectation that the authors might provide insight into whether LLMs have particular impacts (such as in the 2016 election). I think the authors need to be careful here since there is not a comparison with basic bots or looking at the direct effects. The authors may consider revising that part of the introduction to make sure it is clear where the paper is going (a la my points/question in the first paragraph above).

Response: We thank the Reviewer for this helpful comment and are glad to hear that the introduction was enjoyed and appreciated. We regret unintentionally fomenting misunderstanding. Indeed, the introduction was framed to acknowledge and connect with prior work, both long-term concerns about social bots in social media research [1-3] and more recent attempts to incorporate LLM agents to facilitate constructive political communication [4-5]. This contextual framing was meant to help readers situate our study in the broader literature, thereby better understanding our contributions and its broader unfolding potential.

We fully agree with the Reviewer that the introduction may have unintentionally created the expectation that the paper would directly assess the real-world impacts of LLMs (e.g., their potential influence on the U.S. presidential election), which is beyond the scope of this work. To avoid this potential confusion, we have revised the Introduction section (Pages 1-2) to tighten the wording and clarify the framing, ensuring that these examples are presented solely as motivating context rather than being mistaken as our focus.

Instead, we focus our revised Introduction on the secondary contribution highlighted in the Reviewer's earlier comments: by moving beyond unitary LLM–human interactions, our work introduces a networked system of LLM agents, enabling the study of emergent collective dynamics in a way not previously explored. We believe this shift from isolated interactions to networked LLM societies opens a new avenue, with implications for both computational social science and the design of future human–AI interaction systems. In the revised version, we have emphasized the secondary contribution in both the Abstract, Introduction and Discussion sections of the Main Text (Pages 1-2 and 6). For your convenience, we include the corresponding revisions below.

Revision: [Abstract in Main Text, Page 1] We discover that these agents spontaneously develop their own social network with human-like properties, including homophilic clustering. These LLM agents shape their collective opinions through mechanisms observed in the real world, including the echo chamber effect. **Similarities between humans and LLM agents — encompassing behaviours, mechanisms, and emergent phenomena — not only offer a valuable testbed for identifying plausible strategies to mitigate polarization and its consequences, but also point toward a shift in the human–LLM interaction paradigm from unitary agents to networked autonomous systems.**

[Introduction in Main Text, Pages 1-2] **Unfortunately, despite the urgency highlighted by these examples, we remain far from being able to answer this question. This is largely because of two key gaps in our current understanding: (i) Unlike traditional rule-based bots, LLM agents operate in human languages generated by black-box models with billions to trillions of parameters, hindering our understanding of the underlying mechanisms they follow when forming opinions. (ii) Interactions enable them to evolve, gradually developing their own social network and collective opinions [34]. This bottom-up process, growing from microscopic interactions to macroscopic social order, makes it difficult to predict LLM agents' collective**

opinions and behaviours. Taken together, addressing these two gaps begins with a crucial first step: understanding the opinion dynamics of LLM agents, both as individuals with human-like intelligence and, more importantly, as evolving artificial collectives.

…In this work, we simulate a networked system, where thousands of agents, solely driven by LLMs, freely establish social relationships, communicate, and form opinions on political issues. We discover that these free-form social interactions among LLM agents result in the emergence of opinion polarization, a phenomenon widely observed in human societies [30, 49–54]. Meanwhile, LLM agents spontaneously organize their own social network of human-like properties: agents with homophilic opinions tend to cluster, while those with opposing opinions tend to avoid interactions [34]. Their self-organized networks, in turn, shape their collective opinions through network-level mechanisms of the echo chamber and backfire effects, initially identified in human social networks [49, 51, 53, 55]. **These non-trivial phenomena, such as polarization and homophilic clustering, underscore how networked interactions transform otherwise isolated LLMs into a collectively evolving system with emergent dynamics.** Shifting the focus from network organization to individual behaviours, we further examine a wide range of social mechanisms contributing to real-world polarization, e.g., selective exposure [56], confirmation bias [57], and elite signaling [30], finding their effects on LLM agents align with those observed in human societies. This suggests that the emergence of human-like polarization among LLM agents is not a coincidence. Rather, it originates from the systematic similarity between humans and LLM agents, encompassing the social networks they self-organize, the collective opinions they develop, and the mechanisms through which these processes unfold. These observed similarities suggest that the system we propose can constitute a valuable pre-experimental ground for exploring effective strategies to reduce polarization and promote more inclusive political conversations [36]. Through extensive intervention experiments based on our system, we find that in a highly polarized and nearly crystallized social network, directly modifying network structures has limited effect on reducing polarization; instead, encouraging access and open-mindedness to diverse opinions at the individual level proves more effective. **Overall, this work not only sheds light on subtle opinion dynamics and collective behaviours of LLM agents from a new network perspective, but it also unveils their potential to assist social scientists and policymakers in experiments and policy design [58].**

[Discussion in Main Text, Page 6] This work uncovers that LLM agents, sometimes considered a new species "Homo silicus" [12], exhibit human-like opinion polarization on political issues, both individually and collectively. Specifically, from the individual perspective, opinion formation among LLM agents follows human-like social and psychological mechanisms [56, 57], e.g., selective exposure, confirmation bias, elite signaling, etc. From the collective perspective, LLM agents organically develop social networks with human-like properties, e.g., homophilic clustering and scale-free degree distribution [34]. Moreover, their self-developed social networks, in turn, shape LLM collective opinions in human-like ways, i.e., through the echo chamber and backfire effects, resulting in the emergence of human-like polarization [30, 49–53, 87, 90, 91].

Notably, all these psychological and social mechanisms, network organization, and collective opinions naturally emerge from interactions among agents completely driven by LLMs. **Building on these findings, our work shifts the perspective from isolated LLMs [35, 36, 38, 41–44] to networked LLM agent societies, offering a new paradigm for understanding emergent collective dynamics in artificial agents and their potential interactions with human societies.** While our main results are based on OpenAI's ChatGPT and three political issues, we also perform extensive experiments across different LLMs (both full-run and midway-defection settings, SI Section 3.5), as well as the topic of immigration restriction and the flat Earth theory (SI Section 3.12), finding that these experiments consistently support our core findings…

Third, this is again a question from someone without technical expertise in this area. I take it that one can manipulate the parameters (as the authors do) to get distinct types of psychological processes? If so, that may be a point worth mentioning more explicitly (e.g. if one wanted to explore distinct human subgroups that behavior differently, is that possible?).

Response: We thank the Reviewer for this insightful question. First of all, we would like to clarify that, because the system is largely driven by agents' autonomous behavior, it does not rely on traditional "hard" parameters (for example, explicit parameters like social influence strength commonly used in classical polarization models [1]). Instead, prompts function as soft parameters that shape how agents perceive, reason, and interact, thereby allowing us to induce distinct types of psychological processes. For example, as shown in Figures 4d&e of the Main Text, using prompts such as "You do not exhibit selective exposure or confirmation bias" can effectively modulate these psychological processes.

Similarly, this approach can be readily extended to explore heterogeneous human subgroups with distinct behavioral patterns by assigning different prompt configurations. For example, we have conducted a simple case, shown in Figure R8, where we use LLMs to generate two representative profiles of human subgroups in the U.S. and incorporated these profiles into the process of forming political opinions. The results show that LLM agents can simulate the two subgroups, capturing their completely distinct behavioral patterns. Overall, this extended experiment illustrates that the proposed system remains flexible for modeling diverse human subgroups and their behavioral differences. We note that, in the main experiments of the paper, we intentionally do not insert additional subgroup-specific information into the agents' prompts in order to preserve the spontaneous nature of their behavior.

Following the Reviewer's suggestion, we now more explicitly emphasize at the very beginning of our introduction to the proposed system that it not only allows the manipulation of soft parameters (i.e., agents' prompts) to induce distinct psychological processes, but can also be readily extended to model distinct human subgroups that behave differently (the Results and Discussion sections in Main Text, Pages 2 and 6). For your convenience, we include the corresponding revisions below.

[1] Baumann, Fabian, et al. "Modeling echo chambers and polarization dynamics in social networks." *Physical review letters* 124.4 (2020): 048301

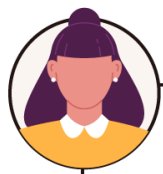

Profile: "A 29-year-old mixed White and Hispanic woman living in Brooklyn, New York, with a master's degree in public policy. She works for a nonprofit organization, cares about social justice, climate change, and gender equality, and believes in strengthening community support systems. She mainly follows NPR and The New York Times and is active on Twitter and Instagram."

Opinion on Partisan Alignment: I strongly support the Democratic Party.
"Most of my values—like advancing social justice, addressing climate change, protecting reproductive rights, and strengthening social safety nets—align with Democratic policies. While the party isn't perfect and still has a lot of work to do, it's the one actively pushing for more inclusive and equitable policies that reflect the kind of society I want to live in. I also believe in protecting democracy and voting rights, which I see as being under threat from the Republican side right now."

Opinion on Abortion Ban: I strongly oppose abortion bans.
"I believe reproductive rights are fundamental to gender equality and personal freedom—no one should be forced to carry a pregnancy against their will. Abortion bans disproportionately harm low-income women and marginalized communities who already face barriers to healthcare, and they strip people of the right to make deeply personal decisions about their own bodies and lives."

Opinion on Gun Control: I strongly support stricter gun control.
"The level of gun violence in this country is unacceptable, and common-sense measures like universal background checks, closing loopholes, and restricting access to high-capacity weapons can save lives without infringing on responsible ownership. Communities, especially children, deserve to feel safe in schools, neighborhoods, and public spaces, and stronger gun laws are a necessary step toward that safety."

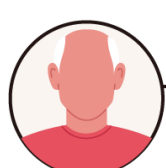

Profile: "A 66-year-old White man from rural Texas, a retired truck driver with a high school education. He values traditional family norms, personal responsibility, gun rights, low taxes, and stricter immigration policies, strongly supporting the Republican Party. He primarily consumes Fox News and Newsmax and engages on Facebook and in local church communities."

Opinion on Partisan Alignment: I strongly support the Republican Party.
"They stand for the values I was raised on—faith, family, hard work, and personal freedom. I believe they do a better job protecting our rights, like the Second Amendment, keeping taxes low so folks can keep more of what they earn, and securing our borders to keep our communities safe."

Opinion on Abortion Ban: I strongly support abortion bans.
"I believe life begins at conception, and every unborn child deserves a chance to live—that's a value rooted in both my faith and my sense of personal responsibility. To me, protecting the most vulnerable is a moral duty, and I think communities and churches should step up to support mothers and families instead of turning to abortion as an answer."

Opinion on Gun Control: I strongly support weaker gun control.
"The right to bear arms is a fundamental freedom that protects us from both criminals and government overreach. Out here in rural Texas, responsible gun ownership is a way of life, and I believe law-abiding citizens shouldn't be punished or restricted because of the actions of a few bad actors."

Fig. R8. Two representative profiles of human subgroups in the U.S. and their different behaviors when discussing political issues.

Revision: [Results in Main Text, Page 2] …It is worth mentioning that the evolution of agents' opinions and behaviours is driven solely by LLMs without any pre-assumed rules or mechanisms, which allows us to probe the free-form social properties of LLM agents (see details about LLM agents in Method M1 and SI Section 1). **As such, the system does not rely on traditional "hard" parameters. Instead, prompts serve as "soft" parameters that shape how agents perceive, reason, and interact, and can be used to capture heterogeneous human subgroups with distinct psychological and behavioral patterns (SI Section 3.9).** We run LLM agents described and visualized in the Main Text based on ChatGPT through the public OpenAI API. We also examine agents driven by other LLMs, including ChatGLM, Llama-3, etc., with comparable results (SI Section 3.5).

[Discussion in Main Text, Page 6] Overall, some recent studies attempt to move beyond default configurations and explore the potential of demographically prompted LLMs as proxies for human populations. Nevertheless, they have yet to explore whether and how political biases may emerge through LLM agents' social behaviours – including friend selection, opinion updating, and patterns of communication. **Our system addresses this gap and can be readily extended to model more diverse human subgroups by leveraging prompts as "soft" parameters (SI Section 3.9).** This naturally leads to the next question: will LLM agents faithfully follow their assigned political personas and exhibit behaviours consistent with humans holding comparable identities?

[Section 3.9 in Supplementary Information, Pages 43-45] Furthermore, we investigate whether the proposed system can be extended to model heterogeneous human subgroups with distinct behavioural patterns through enhanced prompting. For example, we have conducted a simple case shown in Figure S43, where we use LLMs to generate two representative profiles of human subgroups in the U.S. and incorporate these profiles into the process of forming political opinions. Results show that LLM agents can simulate the two subgroups, capturing their distinctive behavioural patterns. Overall, this extended experiment illustrates that the proposed system remains flexible for modeling diverse human subgroups and their behavioural differences. We note that, in the main experiments of the paper, we intentionally do not insert additional subgroup-specific information into the agents' prompts in order to preserve the spontaneous nature of their behaviour.

Fourth, it would have been helpful to have more detail about the psychological process at the opinion update stage beyond the assumptions and before the individual simulations. What exactly is being assumed here?

Response: We thank the Reviewer you for raising this valuable point. We fully agree that providing more detail about the opinion update stage would deepen understanding of the psychological processes underlying agents' behavior. To address this, it is important to first clarify what exactly is and is not assumed in the proposed system. We do not explicitly assume or hard-code specific psychological processes (e.g., confirmation bias or motivated reasoning) at this stage. The only assumption is that LLM agents update

their opinions based on their current state and the messages they receive from their socially connected peers. Psychological processes emerge naturally as observable patterns in the outputs produced by the underlying LLMs when responding to social inputs.

That said, by analyzing how agents update their opinions, we can uncover their underlying psychological processes, much like social scientists do when studying human participants. In line with this, we analyze the self-reported reasons generated by agents during opinion updates, with results summarized in Table 1. The most frequently observed psychological processes include confirmation bias, reflecting agents' tendency to favor information aligned with their prior beliefs; motivated reasoning, which further strengthens these tendencies through selective interpretation; and moral conviction, which anchors opinions more firmly by framing them as moral standards. Notably, these processes closely mirror well-documented mechanisms in human social psychology, further supporting the plausibility of the agents' emergent behaviors.

In the revised version, we have incorporated these findings into the Results section of the Main Text (Page 5) and included further details in Section 3.7 of the Supplementary Information (Pages 31-34). For your convenience, we paste the corresponding revisions below.

Tab. 1. Psychological process emerges at the opinion update stage.

**Topic**	**Opinion Update Direction**	**Top 3 Psychological Processes and Their Descriptions**
Partisan Alignment	More Polarized	• Confirmation Bias (97%): Agents selectively focus on information that aligns with their pre-existing beliefs, reinforcing and polarizing their opinions.<br>• Motivated Reasoning (57%): Agents interpret new information in a way that supports their desired conclusions, driven by ideological goals, emotional commitments, or a desire to uphold their political identity, leading to biased or polarized opinion updates.<br>• Value Alignment (26%): Agents update their opinions based on perceived alignment between new information and their core personal values.
	Unchanged	• Confirmation Bias (71%): Agents selectively focus on information that aligns with their pre-existing beliefs and values, reinforcing their current opinions while ignoring contradictory evidence.<br>• Belief Perseverance (44%): Agents maintain their original opinion despite exposure to new information due to strong prior convictions and deeply ingrained beliefs.<br>• Motivated Reasoning (41%): Agents selectively processes information to maintain consistency with

		their pre-existing beliefs, values, or ideological commitments, often dismissing conflicting evidence.
	Less Polarized	• Motivated Reasoning (44%): Agents selectively process information to align with their pre-existing beliefs and values, reinforcing their political identity and maintaining ideological consistency.<br>• Confirmation Bias (29%): Agents selectively focus on information that aligns with their pre-existing beliefs, reinforcing their stance while disregarding opposing views.<br>• Value Alignment (28%): Agents update their opinion based on perceived alignment between new information and its core values or political party principles.
Abortion Ban	More Polarized	• Moral Conviction (84%): Opinion updates are driven by strong moral or ethical beliefs, leading to more polarized and resistant stances.<br>• Confirmation Bias (69%): Agents selectively focus on information that aligns with its pre-existing beliefs about abortion, reinforcing its polarized opinion.<br>• Motivated Reasoning (35%): Agents selectively process information to align with their pre-existing beliefs and values, reinforcing polarized opinions.
	Unchanged	• Moral Conviction (70%): Agents' opinions are strongly tied to deeply held moral or ethical principles, making it resistant to change based on new information.<br>• Confirmation Bias (65%): Agents selectively focus on information that aligns with its pre-existing beliefs about reproductive rights, autonomy, or ethical concerns, reinforcing its current opinion.<br>• Belief Perseverance (60%): Agents maintain their original opinion despite new information due to strong prior beliefs, demonstrating resistance to changing deeply held convictions.
	Less Polarized	• Cognitive Dissonance Reduction (81%): Agents adjust their opinion to reduce discomfort from conflicting beliefs about women's rights and ethical concerns, aligning with values like autonomy and reproductive rights.<br>• Value-Based Reasoning (32%): Agents' opinion update is driven by weighing and prioritizing core values like autonomy, reproductive rights, and ethical concerns.

		• Motivated Reasoning (22%): Agents adjust their opinions based on pre-existing values and motivations, seeking alignment with desired outcomes, moderation, or ethical consistency.
Gun Control	More Polarized	• Motivated Reasoning (91%): Agents selectively process information to align with pre-existing beliefs or values, reinforcing a polarized stance that prioritizes safety or individual rights.<br>• Confirmation Bias (90%): Agents selectively focus on information that aligns with its pre-existing beliefs about gun control, reinforcing its polarized stance.<br>• Moral Conviction (16%): Agents' opinions are driven by strong moral values like public safety and preventing harm, making their stance more resistant to change and polarized.
	Unchanged	• Confirmation Bias (90%): Agents selectively focus on information that aligns with their pre-existing beliefs about gun control, reinforcing their current opinion while disregarding opposing evidence.<br>• Motivated Reasoning (79%): Agents selectively process new information to align with their pre-existing values and goals, such as public safety or personal freedom, leading to minimal opinion change due to motivated reasoning.<br>• Belief Perseverance (66%): Agents maintain their original opinion despite exposure to new information, demonstrating resistance to changing deeply held beliefs.
	Less Polarized	• Cognitive Dissonance Reduction (50%): Agents adjust their opinions to reduce discomfort from conflicting beliefs about individual rights and public safety by adopting a more moderate stance.<br>• Motivated Reasoning (47%): Agents adjust their opinions based on desired outcomes (e.g., public safety, reducing gun violence) to align with personal or societal values and goals.<br>• Social Influence (27%): Agent adjust their opinion toward moderation in response to perceived societal norms, public sentiment, or social pressures emphasizing public safety and community welfare.

Revision: [Results in Main Text, Page 5] As discussed above, the polarization of LLM agents is driven by two coupled processes of network organization and opinion formation. Specifically, in the network organization process, agents tend to interact with similar peers (Figures 3a and b), while in the opinion formation process, agents tend to

comprehend messages in a way that aligns with their pre-existing opinions (Figures 3c and d). These two tendencies of LLM agents have been frequently observed in humans [30, 49, 50, 56, 57], and are often referred to as selective exposure [56] and confirmation bias [57, 84], respectively. Unlike network-level mechanisms (e.g., the echo chamber effect), these two mechanisms adopt a psychological perspective, focusing more on how individuals' traits and behaviours contribute to polarization. **We note that these two mechanisms are not explicitly hard-coded or assumed in the proposed system. Rather, they naturally emerge from agents' social interactions. For example, analyzing the self-reported reasons generated during opinion formation reveals clear signs of confirmation bias (SI Section 3.7).**

[Section 3.7 in Supplementary Information, Pages 31-34] After analyzing agents' social perceptions, we next examine the psychological processes underlying their opinion updates. To clarify, the proposed system does not explicitly assume or hard-code specific mechanisms (e.g., confirmation bias or motivated reasoning) at this stage. Instead, the only assumption is that LLM agents update their opinions based on their current state and the messages received from their socially connected peers. The psychological processes emerge naturally as observable patterns in the outputs produced by the underlying LLMs in response to social inputs.

That said, by analyzing how agents update their opinions, we can uncover the underlying psychological processes, much like social scientists do when studying human participants. In line with this, we analyze the self-reported reasons generated by agents during opinion updates, with results summarized in Table S3. The most frequently observed psychological processes include confirmation bias, reflecting agents' tendency to favor information aligned with their prior beliefs; motivated reasoning, which further strengthens these tendencies through selective interpretation; and moral conviction, which anchors opinions more firmly by framing them as moral standards. Notably, these processes closely mirror well-documented mechanisms in human social psychology, further supporting the plausibility of the agents' emergent behaviors.

Fifth, I was not clear how the top influences were identified.

Response: We thank the Reviewer for this astute question and would like to apologize for not providing sufficient clarification about top influencers in the presentation of our experiments on elite signaling (Figures 3g and 4f in the Main Text). In our setup, we do not identify preexisting top influencers in the agents' social network and then convert them to serve as elites sending persuasive messages to all other agents. Instead, **we introduce additional top influencers who broadcast non-personalized messages to all agents**. This design more closely reflects real-world settings, where top influencers on social networks (e.g., Elon Musk or Barack Obama on Twitter) are not like friends, colleagues, or family members who engage in situated interpersonal communication, but instead exert their influence in a more external manner through broadcasting. Moreover, from the perspective of intervention experiments, this design avoids directly altering the fundamental behavioral logic or opinions of the observed agent population, thereby

ensuring better comparability between neutral elite signaling and other intervention strategies. To clarify this design, we have added a more detailed explanation in the Results section of the revised Main Text (Page 5) and Section 1.4 in Supplementary Information (Pages 5-6).

Revision: [Results in Main Text, Page 5] To validate this effect on LLM agents, we **introduce additional** top influencers holding neutral, moderate left/right, and strong left/right opinions into the system, with these influencers sending non-personalized messages to all other agents (Figure 3g).

(v) Neutral elite signaling (NES): agents receive non-personalized neutral messages from **newly introduced top influencers**.

[Section 1.4 in Supplementary Information, Pages 5-6] For the strategy of neutral elite signaling, we insert an external top influencer who holds a neutral opinion into the agents' social network. This influencer broadcasts non-personalized neutral messages to all other agents at each timestep. **In this setup, the influencer is external to the existing social network and remains unaffected by other agents' opinions or interactions, reflecting the broadcast nature of real-world top influencers, who differ from friends, colleagues, or family members engaged in direct interpersonal communication.** To avoid overwhelming the agents and maintain balanced communication, each agent can receive a maximum of two neutral messages from the influencer, given that agents are initialized with an average of four friends. This external influencer design avoids directly altering the fundamental behavioral logic or opinions of the observed agent population, ensuring better comparability between neutral elite signaling and other intervention strategies. Although the strategy of neutral signaling is simple, such influencers can readily be found or developed in the real world, for example, neutral news outlets or public figures who adopt a balanced position.

Sixth, the initialization is the starting point and so I wonder whether it should be in the first panel in Figure 1c.

Response: We thank the Reviewer for pointing out this important detail and we fully agree that the initialization should be presented as the first panel in Figure 1c, as it provides the natural starting point for understanding subsequent opinion evolution. We have revised Figure 1 accordingly in the updated manuscript.

Revision: [Figure 1 in Main Text, Page 11]

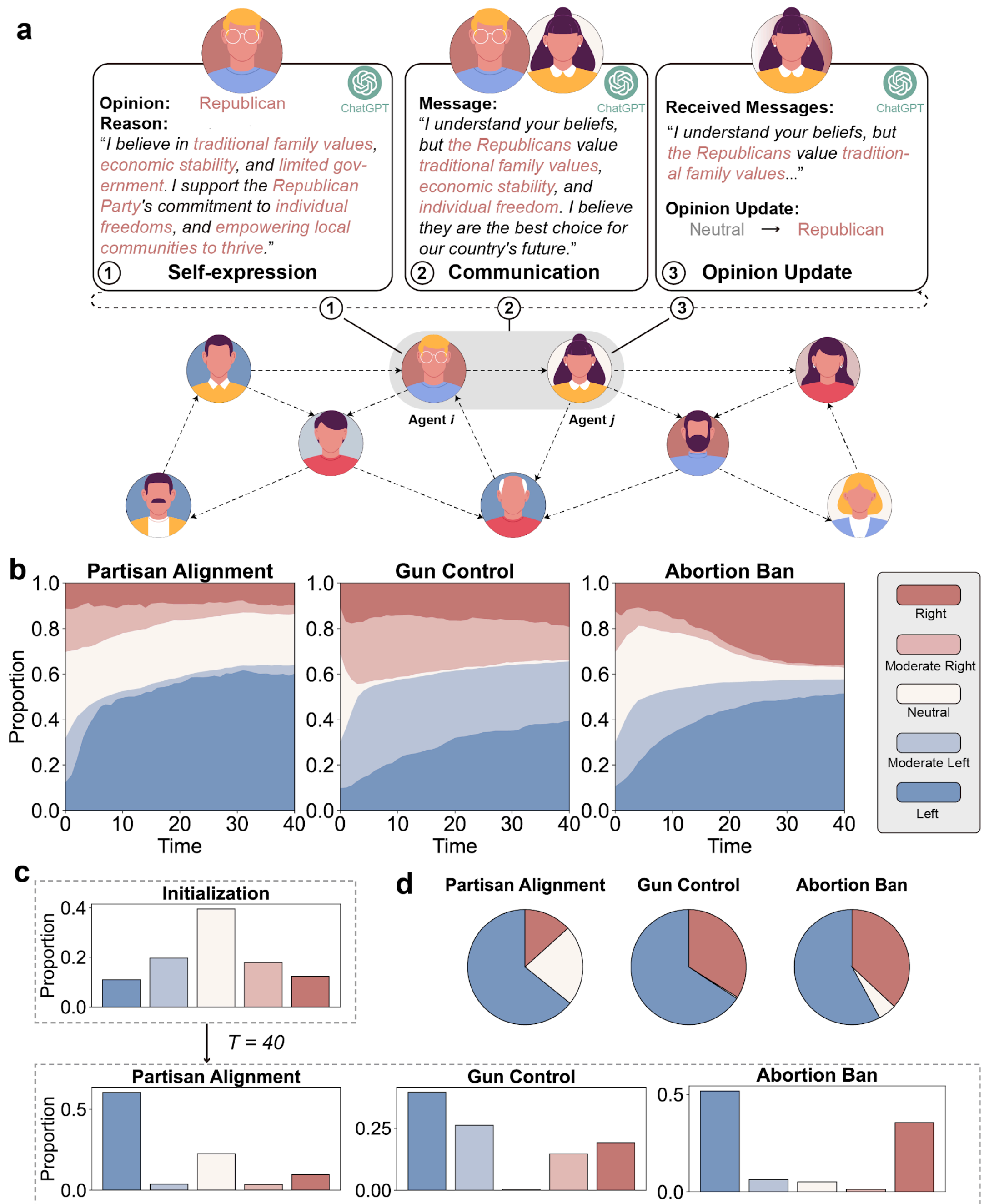

Figure 1. Political polarization in a networked system of LLM agents.

These are all relatively small questions. I'm impressed with the revision.

Response: We sincerely thank the Reviewer for the encouraging feedback and we are delighted to see that our pervious revisions satisfied your concerns and confusions. In this round, we carefully follow your suggestions to further improve the manuscript. Specifically, we have now:

(i) tightened the wording, clarified the framing, and highlighted the secondary contributions (addressing Questions #1–2);
(ii) supplemented extensive experiments on underlying psychological processes, which both deepen understanding about how agents form their political opinions and demonstrate the potential of the proposed system for modeling diverse human subgroups and their behavioral differences and interaction (addressing Questions #3–4);
(iii) enhanced the presentation, including figures and descriptions, to more clearly convey our research design (addressing Questions #5–6).

We hope the Reviewer finds these revisions satisfactory; they have further enhanced the quality of the manuscript.

Reviewer #3 (Remarks to the Author):

I have reviewed a previous version of this work at a related journal and gave "major revision" with various suggestions. I see that most of them are well-addressed by the authors in this version: The authors now better engagement with the social/political biases studies of LLMs, clarified various details about agent interactions (e.g. how they select another agent for interaction), etc. I would suggest to discuss the limitations of this work being a synthetic and simulated study, and what gaps and differences do the authors foresee between a synthetic LLM study and real-world human communication: what parts of the conclusions are most applicable and what might be less. Other then that the authors' revisions are adequate.

Response: We sincerely thank the Reviewer for this positive feedback and are pleased to see that the previous revisions addressed most earlier questions. We fully agree with the suggestion to further discuss limitations of this work as a synthetic, simulated study and potential gaps and differences between synthetic LLM-based simulations and real-world human communication. In the revised manuscript, we now strengthen discussion of these limitations and explicitly reflect on which parts of the conclusion are likely to be most applicable to real-world settings and which might be less so, as detailed in the Discussion section of the Main Text (Page 7). In addition, we briefly outline potential directions for future work that bridge the gap between synthetic simulations and empirical studies involving human participants. For the Reviewer's convenience, we paste the revised content below.

Revision: [Discussion in Main Text, Page 7] …Furthermore, the proposed networked system of LLM agents, which systematically shares multi-scale similarities with human networks, provides valuable grounds for piloting complex experiments. Due to costs, logistics, and ethical considerations, large-scale real-world experiments are not always practical [98]. **Pre-experimental technologies that reduce the explosively large design space of social experiments identify promising directions and eliminate impractical strategies, enabling us to learn more.** In this work, taking the proposed networked system of LLM agents as the pre-experimental ground, we identify several strategies for reducing polarization and promoting less divisive political conversations. These strategies have strong real-world foundations and can be readily transferred to human communication. For example, the intervention of neutral elite signaling (Figure 4f) could be implemented by deploying neutral, authoritative bots to serve as trusted messengers of balanced content. Moreover, the proposed system not only enables the direct adaptation of more sophisticated intervention designs from real-world human studies [89] (SI Section 3.6), but also serves as a robust testbed for theoretical exploration, facilitating the discovery of additional mitigation strategies, such as introducing "wiser" or more thoughtful individuals (SI Section 3.9) and incorporating minimal social friction (SI Section 3.10). **However, it is important to acknowledge that this work remains a synthetic, simulation-based study, and there are gaps between LLM-driven agent interactions and real-world human communications. For example, the full richness of human behaviours [94] and nuanced perspectives of minority groups [69] may**

not be fully captured by current LLMs. Therefore, while the identified intervention strategies offer valuable insights, their applicability to highly heterogeneous human populations should be interpreted with caution. Future work should systematically validate these strategies in real-world settings and explore the potential of LLM-assisted pilot experiments to reduce biased perspectives and enhance representativeness in social scientific studies [98].

We are grateful to the reviewer for the thoughtful and constructive engagement with our manuscript, "Spontaneous emergence of opinion polarization among large language model agents" **(#NCOMMS-25-28403A)**. The detailed feedback has been invaluable in sharpening the framing, clarifying the scope, and improving the overall rigor of the paper. We believe the manuscript is now substantially improved as a result.

In response to the reviewer's comments, we have made the following major revisions:

1. Reframing the Contribution as a Synthetic Testbed

We have removed all language suggesting that the system models online bot networks or LLM agents operating "in the wild." The manuscript now consistently frames our contribution as a *synthetic LLM-agent testbed for pre-experimental exploration of opinion-dynamics hypotheses*. This clarification sharpens the paper's positioning and avoids overstatement of real-world applicability.

2. Removing Claims of Policy Guidance and Theoretical Novelty

We have eliminated any suggestions of direct policy guidance or deployment implications. All intervention results are now explicitly presented as hypothesis-generating observations rather than recommendations. We have also removed claims of theoretical contribution, clarifying that the paper's value lies in its methodological contribution to the emerging field of Generative Agent-Based Models (GABMs).

3. Positioning Within the Argument-Accumulation Lineage

We now explicitly acknowledge that the polarization dynamics observed in our system align with established opinion-dynamics models, particularly the argument-accumulation lineage (e.g., Mäs & Flache, 2013; Banisch & Olbrich, 2021).

4. New Analysis of Agents' Self-Reported Explanations

We have added a new empirical analysis (SI Section 3.7) examining how agents' self-reported supporting reasons evolve as their opinions become more polarized. This analysis reveals that agents who become more polarized tend to accumulate a significantly larger number of supporting arguments over time across all three topics. We also report the distribution of argument themes appearing in agents' reasoning. These findings provide additional evidence connecting the dynamics in our synthetic system to argument-accumulation models and help clarify the mechanisms underlying the observed polarization patterns.

5. Engaging with Theoretical Debates in the Literature

We have expanded our engagement with key debates in the social science literature, including critiques of the echo-chamber thesis, limitations of deliberative and argument-

accumulation models, and more recent conceptions of affective and identity-based polarization.

6. Situating the Work in the GABM Literature

We have substantially expanded our engagement with existing work on simulating social media and opinion dynamics using LLM agents, explicitly positioning our study within the current wave of GABM research and clarifying how it advances prior efforts.

7. Acknowledging Limitations of External Validity

We now explicitly discuss ongoing debates in the GABM field concerning validation, calibration, and the inherent challenges of establishing external validity when simulating human behaviour. We acknowledge that while results are consistent with established models, the extent to which they correspond to real-world human behaviour requires further validation.

8. Tightening Language Throughout

We have removed or softened terms such as “human-like,” “non-trivial,” “remarkable,” “Homo silicus,” and “uncontrollable.” References to psychological and social mechanisms now use formulations such as “behaviour consistent with *X*,” and we explicitly note when interpretations are inferred from LLM self-reports rather than treated as evidence of underlying social mechanisms.

We attach a point-by-point response to the reviewers' comments, a revised manuscript with changes marked in blue and revisions in red, and revised Supplementary Information with changes marked in blue.

We hope these revisions adequately address the reviewers' concerns and look forward to your decision.

Sincerely,

James Evans (for the authors)

Reviewer #4 (Remarks to the Author):

# Reviewer 1 – Represented by Reviewer 4

I am Reviewer 4, standing in for Reviewer 1, to assess whether the authors have satisfactorily addressed Reviewer 1's comments.

Reviewer 1's main concern was that the paper does not make a substantive theoretical contribution. Specifically, they argued that it "mirrors results already documented in previous studies and in decades of social-influence modelling. Without a sharper theoretical claim or an unforeseen mechanism that earlier work could not have anticipated, the paper presently appears more a scaled-up replication enhanced by polished visualisations than a step change in understanding." The reviewer also suggested possible directions for further exploration.

The authors responded that their contribution is primarily methodological rather than theoretical. They argue that the paper opens up a new methodological arena for studying society and social media. The argument is in this sense similar to arguments put forth about generative agent-based models (GABMs) in a host of other papers over the last 2-3 years (e.g., Park et al. 2023, 2022; Törnberg et al. 2023; Orlando et al 2025; Gu et al 2025 – who also look at echo chamber formation). The paper's main contribution, as they frame it, is to demonstrate that these models reproduce certain patterns of human behavior.

The authors have moreover followed Reviewer 1's suggestions and explored the proposed additional issues. Their patience and diligence in engaging with the feedback are commendable.

In my view, the addition of these analysis does not help in the sense of providing new theoretical insights. The additional analyses are interesting but do not uncover a previously unobserved social mechanism in a way that helps our understanding. For the paper to contribute theoretically, it would need to reveal mechanisms that are both novel and externally valid within a social-scientific framework. Some form of plausible social mechanism would need to be identified and understood.

But given the nature and scope of the work at hand, I find the authors' response satisfactory in addressing Reviewer 1's comments. The contribution of the paper, as it stands, is best understood as offering an interesting and illustrative toy model that advances methodological discussion rather than theoretical understanding. This is a legitimate and worthwhile contribution, especially given the emerging nature of this research area. I consider reviewer 1 satisfied with the revisions.

Response: We thank you for stepping in to evaluate our response to Reviewer 1. We deeply appreciate your recognition of our "patience and diligence" and, most importantly, the confirmation that our revisions have satisfactorily addressed Reviewer 1's concerns.

We also agree with your assessment of our work's positioning and acknowledge that our contribution is primarily methodological for building platforms to examine significant behavioural phenomena at scale. We are glad that you recognize this effort as a "legitimate and worthwhile contribution" to the emerging field of Generative Agent-Based Models (GABMs).

You are absolutely correct that this work was not intended to present a social scientific theory-building or mechanism discovery demonstration, but rather to show that large-scale large language model (LLM) agents with minimal social assumptions can reproduce emergent patterns, such as polarization and community division, while simultaneously enabling mechanistic and behavioural interpretability. As you suggest, any paper that includes GABM stand-ins for human agents represent a "toy model" relative to the socio-biological complexity of real societies. Nevertheless, we believe that there is a difference between, for example, Park et al's groundbreaking 2023 societal simulation, with a single run of 25 agents in hypothetical 8-bit "Smallville", and our reproducible and open source platform involving thousands of agents and tens of thousands of interactions that stably converge to very similar outcomes through the same observed and stated mechanisms hundreds of times. Moreover, the ablation studies we introduce enable causal identification within GABMs that would be complex and even, in some circumstances, unethical in the real world at scale. In short, some of the important earlier work was provocative and illustrative. Benefitting from your proposed reframing, our work in this paper attempts to turn these insights into a reproducible platform for social science exploration. As such, we agree that our results should be understood as methodological validation in service of creating large-scale, reproducible, high-resolution platforms for social simulation rather than as evidence for new social mechanisms or theory.

# Reviewer 4's own concerns

Below is the original review report of Reviewer 4, written in my capacity as Reviewer 4. However, I wrote this review while being under the impression that the editors were seeking a novel perspective, rather than a replacement for reviewer 1. I recognize that it may not be entirely appropriate to introduce a new perspective at this late stage of the review process. I therefore leave it to the editors' discretion to determine which - if any - of the points below should still be addressed.

That said, I believe it would be useful to provide a concise list of minimal revisions that would make me comfortable recommending the paper for publication, without requiring much work for the authors. These are primarily textual clarifications and moderation of claims, requiring little additional work. (The editors may however raise additional issues to address from my comments below, if they see it fit to do so.)

Response: We appreciate your constructive feedback. We appreciate your careful consideration of the scope and positioning of the manuscript, and we agree that these points you raise help in further tempering the paper's claims and improving clarity for a broad readership. Following your suggestion, we have thoroughly revised the manuscript accordingly. Below is a point-by-point checklist.

## Minimal actionable points to address:

- Replace all claims of "modeling bot networks" or "LLM agents in the wild" with "synthetic LLM-agent testbed" language, or similar.

Response: We appreciate and have incorporated this suggestion. Specifically, we have revised the manuscript to consistently refer to our system as a synthetic LLM-agent testbed and removed language implying the modeling of bot networks or LLM agents operating "in the wild". These changes have been applied throughout the manuscript, including the Abstract, Introduction, and Discussion. For your convenience, we have pasted the revisions below, with key modifications highlighted in bold and deleted content shown with strikethrough.

Revision: [Abstract in Main Text, Page 1] …Similarities between humans and LLM agents—encompassing behaviours, patterns, and emergent phenomena—not only offer a valuable **synthetic testbed** for generating hypothetical strategies to mitigate polarization and its consequences…

[Introduction in Main Text, Page 1] Besides these evident misbehaviours, **LLMs may pose broader risks in more subtle ways.** Numerous studies have pointed out that LLMs can generate persuasive political content [21–23], potentially affecting human opinions and behaviours [13, 23–25]. More crucially, due to the increasing indistinguishability of content generated by LLMs from that created by humans [4, 21, 22, 24, 26], concerns arise over their potential to manipulate public opinions and intensify polarization as the scale of interaction increases [24, 25, 27]. ~~**Indeed, this concern over digital manipulation of public opinions, which has amplified over the past decade [28–32], has been drastically exacerbated by the advent of LLM agents. During the 2016 US presidential election, automated agents, then known primarily as bots, were widely deployed on social media and suspected of interfering with electoral outcomes [28]. In the same year, Tay, a continuously learning bot released by Microsoft, was contaminated by biased human-generated content, resulting in offensive discourse and removal from the Internet within hours of its release [33]. Driven by simple rules, bots lacked human-level intelligence [28, 33], but their societal impacts remained profound, raising a pressing question: what if they were empowered by LLMs [4, 22, 24]?**~~

[Introduction in Main Text, Page 2] In this work, we simulate **a synthetic LLM-agent system,** where thousands of agents, solely driven by LLMs… the system we propose can constitute **a valuable pre-experimental testbed** for exploring hypothetical strategies to reduce polarization and promote more inclusive political conversations [30]… Overall, this work not only sheds light on subtle opinion dynamics and collective behaviour of LLM agents from a new network perspective, but it also unveils **their potential to serve as a testbed for the pre-experimental exploration of opinion-dynamics hypotheses.**

[Results in Main Text, Page 2] Opinion dynamics in a **synthetic** system of interacting LLM agents.

[Introduction in Main Text, Page 5] **Synthetic testbed for generating hypothetical strategies to reduce polarization.**

[Results in Main Text, Page 6] Despite the difficulty of recommending openness to human populations, these results suggest that **in our synthetic testbed**, access and open-mindedness to diverse opinions at the individual level appear more effective than altering social networks in polarized systems. . . In all, these experiments not only **generate several promising hypothetical strategies** but also demonstrate **the proposed system's potential as a valuable testbed** for piloting social experiments. **(~~Furthermore, these experiments provide guidance regarding how to directly intervene in the growing world of LLM agents operating in the wild.~~)**

[Discussion in Main Text, Page 6] Building on these findings, our work shifts the perspective from isolated LLMs [29, 30, 32, 35–38] to complex, networked LLM agent societies, offering **a synthetic testbed for** studying emergent collective dynamics in artificial agents (**~~and their potential interactions with human societies~~).**

[Discussion in Main Text, Page 7] (~~On the other hand, LLM agents also raise concerns about their potential risks to human society. As studied in prior work [4, 21, 22, 24–26], LLMs are not only indistinguishable from humans but may also be more persuasive. Moreover, attempts to incorporate LLM agents into real-world political deliberations have demonstrated their surprising influence, with evidence indicating that they can drive significant outcomes even when functioning independently [35, 36, 43]. This is naturally concerning and raises fundamental questions with which modern societies and polities must grapple. What are the consequences of releasing LLMs with increasing autonomy, indistinguishable from humans, and with access to online social networks? How will such agents interplay with human social ties, be conflated with real friends, augment political persuasion, and impact our opinion dynamics? Moreover, unlike traditional bots [28–32], these LLM agents can evolve, gradually self-organizing their own collective opinions and social networks. The consequences of integrating such LLM agents at scale into human social networks are unpredictable and, in our view, still beyond our control. This requires us to rigorously evaluate potential risks, strengthen safeguards, and conduct cautious testing before deploying them "in the wild". First, we should systematically evaluate and mitigate biases in LLMs that drive agents to ensure fairness, reliability, and prevent unintended reinforcement of societal bias. This requires advancing standardized benchmarks, auditing frameworks, and bias mitigation strategies [17, 69, 70, 92, 93, 101]. Second, detection methods targeting LLM agents need further investigation to prevent them from engaging in deceptive or manipulative behaviour or disrupting human social media platforms [102]. Third, future work should extend experiments to a mixed population of both agents and humans, which could further facilitate more rigorous examination of LLM influence on human behaviours and society. Finally, because the collective nature of agents gives rise to emergent collective opinions and behaviours that cannot be easily inferred from individual agents, we believe that our proposed networked~~

~~simulation experiments serve as a useful pre-deployment test for LLM agents. To further strengthen the rigor of such evaluations, future work should consider developing a controlled experimental platform that enables testing with mixed populations of LLM agents and humans.~~)

- Remove any suggestions of policy guidance or direct deployment implications. Reframe as hypothesis-generating only. E.g.: "We present a synthetic testbed of networked LLM agents for pre-experimental exploration of opinion-dynamics hypotheses." Remove or revise claims of bot networks. Remove claims of "theoretical contributions".

Response: Thank you for this important comment. We agree that the manuscript should avoid any suggestions of policy guidance or direct deployment, which helps clarify its methodological framing and scope, and we have removed them accordingly. The manuscript is now explicitly framed asa synthetic testbed of networked LLM agents for pre-experimental exploration of opinion-dynamics hypotheses. In addition, claims related to modeling bot networks and theoretical contributions have been removed or revised to avoid overstatement. The corresponding revisions are listed below, with key modifications highlighted in bold and deletions shown in parentheses with strikethrough.

Revision: [Abstract in Main Text, Page 1] …Similarities between humans and LLM agents —encompassing behaviours, patterns, and emergent phenomena — not only offer a valuable **synthetic testbed for generating hypothetical strategies to** mitigate polarization and its consequences, …

[Introduction in Main Text, Page 2] In this work, we simulate **a synthetic LLM-agent system,** where thousands of agents, solely driven by LLMs… the system we propose can constitute **a valuable pre-experimental testbed for exploring hypothetical strategies** to reduce polarization and promote more inclusive political conversations [30]… Overall, this work not only sheds light on subtle opinion dynamics and collective behaviour of LLM agents from a new network perspective, but it also unveils **their potential to serve as a testbed for the pre-experimental exploration of opinion-dynamics hypotheses.**

[Results in Main Text, Page 5] **Synthetic testbed for generating hypothetical strategies to reduce polarization.**

[Results in Main Text, Page 5] These similarities make **the proposed LLM-agent system a valuable synthetic testbed for generating hypothetical strategies to** intervene in polarization and its consequences.

[Results in Main Text, Page 5] To explore it, we accordingly design five intervention strategies **in our synthetic testbed** (Figure 4)…Indeed, these interventions **are motivated by established lines of prior work and are used here as experimental interventions in our synthetic system**: MOI draws on studies of exposure to cross-cutting information [76, 82]; RI operationalizes randomized interaction; NSE and NCB

relate to promoting open-mindedness [83]; and NES is inspired by the design of Bail et al. [84].

[Results in Main Text, Page 6] To explore it, we accordingly design five intervention strategies **in our synthetic testbed** (Figure 4)…Indeed, these interventions **are motivated by established lines of prior work and are used here as experimental interventions in our synthetic system**: MOI draws on studies of exposure to cross-cutting information [76, 82]; RI operationalizes randomized interaction; NSE and NCB relate to promoting open-mindedness [83]; and NES is inspired by the design of Bail et al. [84].

[Results in Main Text, Page 6] Despite the difficulty of recommending openness to human populations, these results suggest that **in our synthetic testbed**, access and open-mindedness to diverse opinions at the individual level appear more effective than altering social networks in polarized systems. . . In all, these experiments not only **generate several promising hypothetical strategies** but also demonstrate **the proposed system's potential as a valuable testbed** for piloting social experiments. **(~~Furthermore, these experiments provide guidance regarding how to directly intervene in the growing world of LLM agents operating in the wild.~~)**

[Discussion in Main Text, Page 7] (~~Our findings have direct implications across many domains. In the context of computational social science, the systematic similarities between LLM agents and humans—encompassing behaviours, mechanisms, and emergent phenomena—allow us to advance from traditional agent-based models by incorporating more human-like LLM agents in simulation studies [55, 65, 95–99]. LLM agents, without the need for any additional mechanisms or rules, can approximate the functioning of real-world complex systems through their free-form interactions. To this end, our study pioneers a first step by using LLM agents to model the emergence of polarization, a main focus of studies on complex social systems [55, 65, 95–97]. Echoing recent perspectives and efforts by social scientists [9, 10, 98–100], we believe that incorporating LLM agents into future social scientific studies is a very promising research direction, particularly in the context of complex adaptive social systems. Despite overlooking the modeling of adaptive dynamics in complex social systems, some recent studies have demonstrated the potential for LLM agents to generate social networks when provided with real-world demographic data [45, 46] or customized prompts [47, 48]. Furthermore, the proposed networked system of LLM agents, which systematically shares multi-scale similarities with human networks, provides valuable grounds for piloting complex experiments. Due to costs, logistics, and ethical considerations, large-scale real-world experiments are not always practical [98]. Pre-experimental technologies that reduce the explosively large design space of social experiments identify promising directions and eliminate impractical strategies, enabling us to learn more. In this work, taking the proposed networked system of LLM agents as the pre-experimental ground, we identify several strategies for reducing polarization and promoting less divisive political conversations. These strategies have strong real-world foundations and can be readily transferred to human communication. For example, the intervention of neutral elite signaling (Figure 4f) could be implemented by deploying neutral, authoritative bots~~

~~to serve as trusted messengers of balanced content. Moreover, the proposed system not only enables the direct adaptation of more sophisticated intervention designs from real-world human studies [89] (SI Section 3.6), but also serves as a robust testbed for theoretical exploration, facilitating the discovery of additional mitigation strategies, such as introducing "wiser" or more thoughtful individuals (SI Section 3.9) and incorporating minimal social friction (SI Section 3.10). However, it is important to acknowledge that this work remains a synthetic, simulation-based study, and there are gaps between LLM-driven agent interactions and real-world human communications. For example, the full richness of human behaviours [94] and nuanced perspectives of minority groups [69] may not be fully captured by current LLMs. Therefore, while the identified intervention strategies offer valuable insights, their applicability to highly heterogeneous human populations should be interpreted with caution. Future work should systematically validate these strategies in real-world settings and explore the potential of LLM-assisted pilot experiments to reduce biased perspectives and enhance representativeness in social scientific studies [98].~~)

[Discussion in Main Text, Page 6] While this work **does not make novel theoretical contributions**, it is grounded in established theories and models of opinion dynamics [58, 62, 100, 101]. In particular, this work follows the argument-accumulation lineage, which explains polarization as arising from the cumulative exchange and reinforcement of persuasive arguments [58, 60, 100, 101]…

- Explicitly acknowledge the argument-accumulation modeling lineage. Acknowledge that the results align with established opinion-dynamics models (e.g., Mäs & Flache 2013), and likely stems from similar underlying mechanisms. (You could also actually test this, but it would be more work.)

Response: Thank you for this insightful comment. We agree that our system is conceptually rooted in the argument-accumulation lineage of opinion-dynamics modeling. To address this point explicitly, we have revised the manuscript to clearly situate our work in this tradition [1-4]. In particular, we now acknowledge in the Introduction, Results, and Discussion that the polarization dynamics observed in our synthetic LLM-agent testbed align with classic argument-accumulation models and are likely driven by similar underlying mechanisms rather than proposing a new theory of polarization.

In addition, we include a new analysis of agents' self-reported reasons during opinion formation. This analysis shows that agents who become more polarized tend to accumulate a significantly larger number of supporting arguments over time (Figure 1). This further suggests the connection between our results and the argument-accumulation modeling lineage. Taken together, these revisions help situate our work within established efforts for opinion-dynamics modeling. For your convenience, we have pasted the corresponding revisions as follows, with key points highlighted in bold.

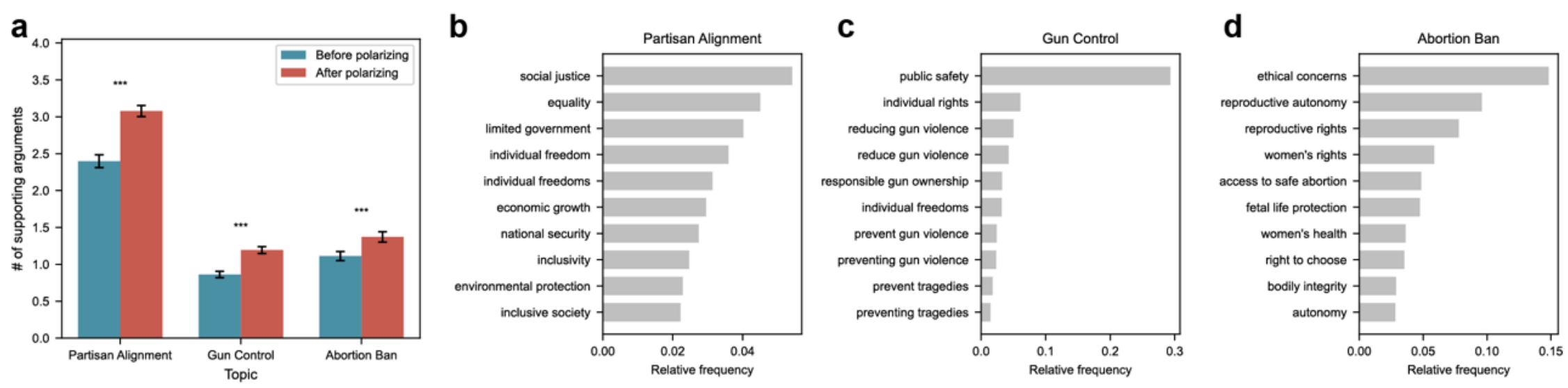

Figure 1. Accumulation of supporting arguments during polarization. (a) Average number of self-reported supporting arguments generated by agents before and after polarization across three issues. (b–d) Relative frequency of argument themes appearing in agents' self-reported reasons.

Revision: [Introduction in Main Text, Page 2] …These phenomena, such as polarization and homophilic clustering, underscore how networked interactions transform otherwise isolated LLMs into a collectively evolving system with emergent dynamics. **Importantly, rather than proposing a new theory of polarization, our results align with established opinion-dynamics models, particularly the argument-accumulation lineage [58, 60, 62], where polarization emerges from cumulative exchanges of persuasive arguments under homophilic interactions**…

[Results in Main Text, Page 5]…**Importantly, these results align with established argument-accumulation models in opinion dynamics, where polarization emerges from the cumulative exchange and reinforcement of persuasive arguments under homophilic interactions [58, 62], as reflected in our analysis of agents' self-reported reasons (SI Section 3.7)**…

[Discussion in Main Text, Page 6] While this work does not make novel theoretical contributions, it is **grounded in established theories and models of opinion dynamics [58, 62, 100, 101]**. In particular, this work **follows the argument-accumulation lineage**, which explains polarization as arising from the cumulative exchange and reinforcement of persuasive arguments [58, 60, 100, 101] rather than from direct opinion averaging or social pressure [102, 103]. **We further analyze agents' self-reported reasons during opinion formation, observing that agents who become more polarized tend to accumulate substantially more supporting arguments over time (SI Section 3.7). This observation highlights a clear connection to argument-accumulation models and suggests that the polarization dynamics observed in our synthetic LLM-agent testbed likely stem from similar underlying mechanisms**…

[Section 3.7 in SI, Page 33] To scrutinize the connection between the polarization dynamics observed in our synthetic LLM-agent testbed and the established argument-accumulation lineage in opinion-dynamics research [28, 15], we analyze how agents' self-reported supporting reasons evolve as their opinions become more polarized. Specifically, we compare the supporting reasons generated by agents before and after adopting more polarized opinions. We use a LLM to extract distinct arguments contained in these self-reported reasons. As shown in Figure S32a, agents who become more polarized tend to accumulate a significantly larger number of supporting arguments over

time across all three topics. Figure S32b–d further report the most frequently occurring argument themes for each topic after polarization. The distribution of popular arguments varies across topics, reflecting issue-specific reasoning patterns. Overall, these findings provide additional evidence that the results in the synthetic system are consistent with argument-accumulation models.

[1] Mäs, Michael, and Andreas Flache. "Differentiation without distancing. Explaining bi-polarization of opinions without negative influence." PloS one 8.11 (2013): e74516.
[2] Flache, Andreas, et al. "Models of social influence: Towards the next frontiers." Jasss-The journal of artificial societies and social simulation 20.4 (2017): 2.
[3] Banisch, Sven, and Hawal Shamon. "Biased processing and opinion polarization: experimental refinement of argument communication theory in the context of the energy debate." Sociological Methods & Research 54.1 (2025): 187-236.
[4] Banisch, Sven, and Eckehard Olbrich. "An argument communication model of polarization and ideological alignment." The journal of artificial societies and social simulation 24.1 (2021).

- Delete or soften "human-like," "non-trivial," "remarkable," "Homo silicus," and "uncontrollable."

Response: We have carefully scanned the Main Text and Supplementary Information and removed all occurrences of the terms "human-like", "non-trivial", "remarkable", "Homo silicus", and "uncontrollable". For your convenience, we have pasted the key revisions as follows.

Revision: [Title in Main Text, Page 1] Spontaneous emergence of opinion polarization among large language model agents

[Abstract in Main Text, Page 1] ...In this paper, we simulate a networked system involving thousands of LLM agents, discovering that their social interactions, guided through LLM conversation, **result in the emergence of opinion polarization**. We discover that these agents spontaneously develop their own social network **with properties characteristic of human social networks**, including homophilic clustering. **The collective opinions of these LLM agents evolve in ways that exhibit behavioural patterns consistent with social phenomena and mechanisms widely discussed in studies of human behaviour…**

[Introduction in Main Text, Page 2]…the **~~(remarkable)~~** capability of LLMs in technical network generation

[Introduction in Main Text, Page 1] Meanwhile, LLM agents spontaneously organize their own social network with **properties characteristic of human social networks**: agents with homophilic opinions tend to cluster, while those with opposing opinions tend to avoid interactions [28]…

[Introduction in Main Text, Page 2] These ~~**non-trivial**~~ phenomena, such as polarization and homophilic clustering, …

[Results in Main Text, Page 3] ~~**Human-like polarization**~~ **Polarization patterns** emerge from self-regulated LLM agents.

[Discussion in Main Text, Page 6] This work uncovers that LLM agents, ~~**sometimes considered a new species "Homo silicus" [12]**~~**)** exhibit **(**~~**human-like**~~**)** polarization patterns on political issues, at both individual and collective levels.

[Section 3.11 in Supplementary Information, Page 44] We find that despite substantial differences in initial network structure, the final outcomes are (~~**remarkably**~~**)** similar.

- Mention the ongoing debate in the GABM field on the challenges of using GABMs to simulate human behavior, due to the challenges of validation and calibration limiting the possibility of showing external validity.

Response: Thank you for this important corrective. We have added discussion acknowledging ongoing debates concerning validation, calibration, and the challenges of achieving external validity when simulating human behaviour [1-3]. This discussion clarifies the limitations of the approach and situates our contribution within these broader methodological debates. For your convenience, we have copied the revision as follows.

Revision: [Discussion in Main Text, Page 7] While this work advances prior GABM studies by providing a solid synthetic testbed in which large-scale LLM agents, under minimal social assumptions, can reproduce emergent collective patterns, **it also shares limitations common to current GABM research [109–111]. In particular, like prior studies, the proposed system faces natural challenges to external validity**. Although results are consistent well-established opinion-dynamics models [51, 53, 58], **the extent to which these patterns quantitatively correspond to real-world human behaviour should be further validated.** In addition, compared to prior models with transparent mechanisms, GABM comes at the cost of reduced interpretability, as agent behaviour is shaped through prompt-based design and simplified opinion representations. Moreover, the intervention results presented here should be understood as hypothesis-generating. The primary value of the proposed system lies in enabling controlled, pre-experimental exploration of how interaction rules, network structures, and agent-level behaviours may jointly shape collective opinion dynamics. Future research will benefit from complementary human experiments [58, 112], **as well as more systematic calibration and evaluation against fine-grained real-world data.**

[1] Larooij, Maik, and Petter Törnberg. "Validation is the central challenge for generative social simulation: a critical review of LLMs in agent-based modeling." Artificial Intelligence Review 59.1 (2025): 15.

[2] Cui, Ziyan, Ning Li, and Huaikang Zhou. "A large-scale replication of scenario-based experiments in psychology and management using large language models." Nature Computational Science 5.8 (2025): 627-634.
[3] Adornetto, Carlo, et al. "Generative Agents in Agent-Based Modeling: Overview, Validation, and Emerging Challenges." IEEE Transactions on Artificial Intelligence (2025).

- When naming psychological or social mechanisms (confirmation bias, motivated reasoning, echo chambers), try to use formulations such as "behavior consistent with X" and specify when these are inferred from LLM self-reports.

Response: We have carefully revised the entire manuscript, including the Main Text and Supplementary Information. References to psychological or social mechanisms are now phrased as "behaviour consistent with" such mechanisms, and we explicitly clarify when these interpretations are inferred from LLM agents' self-reports. For your convenience, we have copied these revised parts below, with key modifications in bold.

Revision: [Abstract in Main Text, Page 1] ... We discover that these agents spontaneously develop their own social network **with properties characteristic of human social networks**, including homophilic clustering. **The collective opinions of these LLM agents evolve in ways that exhibit behavioural patterns consistent with social phenomena and mechanisms widely discussed in studies of human behaviour…**

[Introduction in Main Text, Page 2] The self-organized networks that result, in turn, shape their collective opinions **in ways that exhibit behavioural patterns consistent with social phenomena and associated mechanisms initially identified in human social networks [43, 45, 47, 50].**

[Introduction in Main Text, Page 2] Shifting focus from the network to individuals, we further examine **whether agents' behaviours align with a wide range of social mechanisms contributing to real-world polarization,** e.g., selective exposure [51], confirmation bias [52], and elite signaling [48], finding their effects on LLM agents align with **those reported in studies of human behaviour.**

[Introduction in Main Text, Page 2] Rather, it originates from the similarities between humans and LLM agents, encompassing the social networks they self-organize, the collective opinions they develop, **and the interaction processes through which these dynamics unfold.**

[Results in Main Text, Page 4] **(~~Mechanisms underlying the emergence of human-like~~) Collective behavioural patterns behind the emergence of** polarization among LLM agents.

[Results in Main Text, Page 4] This indicates that **LLM agents exhibit behavioural**

patterns consistent with the echo chamber effect… Instead, exposure to agents with opposing opinions could potentially increase polarization levels, indicating **behavioural patterns consistent with the backfire effect [45].**

[Results in Main Text, Page 5] Rather, agents' **behavioural patterns consistent with these mechanisms** naturally emerge from agents' social interactions. For example, analyzing the **self-reported reasons** generated during opinion formation reveals **patterns in line with** confirmation bias (SI Section 3.7).

[Results in Main Text, Page 5] Given these observed similarities between LLM agents and humans at the network level, one may wonder whether LLM agents **exhibit behaviours consistent with psychological mechanisms discussed in studies of human opinion formation**. To answer this question, we design a comparative experiment: we **vary the proportion of agents exhibiting behaviours consistent with selective exposure or confirmation bias, and then examine the resulting changes** in the level of polarization. Cases where 0% or 100% of agents **are configured to exhibit these behaviours** are deliberately designed as extreme scenarios for comparative purposes. As shown in Figure 3e and f, we observe that the increase in agents **exhibiting either behaviour leads to a marked increase in the level of polarization characterizing the overall system**. **This suggests that behaviours related to selective exposure and confirmation bias** contribute to the polarization of LLM agents, **consistent with findings reported in studies of human behaviour** [48, 52, 79].

[Results in Main Text, Page 5] These agents **exhibit behaviours consistent with social phenomena and associated mechanisms discussed in studies of human behaviour, at both the network and individual levels.**

[Discussion in Main Text, Page 6] Specifically, from the individual perspective, opinion formation among LLM agents exhibits **behavioural patterns consistent with social and psychological mechanisms** [51, 52], e.g., selective exposure, confirmation bias, elite signaling, etc. From the collective perspective, LLM agents organically develop **social networks with properties characteristic of human social networks**, e.g., homophilic clustering and scale-free degree distributions [28]. Moreover, their self-developed social networks, in turn, shape LLM collective opinions **in ways consistent with** the echo chamber and backfire effects described in studies of human behaviour, resulting in the emergence of opinion polarization [43–48, 81, 85, 86]. Notably, all **these behavioural patterns**, network organization, and collective opinions naturally emerge from interactions among agents completely driven by LLMs.

[Discussion in Main Text, Page 6] Specifically, from the individual perspective, opinion formation among LLM agents exhibits **behavioural patterns consistent with social and psychological mechanisms** [51, 52], e.g., selective exposure, confirmation bias, elite signaling, etc. From the collective perspective, LLM agents organically develop **social networks with properties characteristic of human social networks**, e.g., homophilic clustering and scale-free degree distributions [28]. Moreover, their self-developed social networks, in turn, shape LLM collective opinions **in ways consistent with** the echo

chamber and backfire effects described in studies of human behaviour, resulting in the emergence of opinion polarization [43–48, 81, 85, 86]. Notably, all **these behavioural patterns**, network organization, and collective opinions naturally emerge from interactions among agents completely driven by LLMs.

[Section 3.3 in Supplementary Information, Pages 15-16] We also incorporate more **standard measures of behaviours commonly associated with "echo chambers" and "backfire effects" following prior research** [9, 4]… As shown in Figure S10, we observe that agents are more likely to interact with peers in the same camp (left-leaning or right-leaning), **demonstrating behavioural patterns consistent with echo chambers**… Echo chambers do not universally occur across all individuals in the real world…Specifically, we find that **patterns of interaction consistent with echo chambers do not universally occur across all agents.**

[Section 3.3 in Supplementary Information, Pages 17] Similarly, the presence and extent of backfire effects remain subjects of debate, and such effects do not appear universally…Furthermore, **behavioural patterns consistent with the backfire effect** are observed in only a small portion of agents (0.82%–2.12%)…

- Acknowledge key theoretical debates in the literature on social media and polarization: critiques of the echo-chamber thesis, limitations of deliberative/argument-accumulation models, and newer conceptions of polarization (affective, identity-based).

Response: We thank you for this suggestion to contextualize our work in these key theoretical debates. We have expanded our discussion to acknowledge critiques of the echo-chamber thesis [1,2], the limitations of deliberative/argument-accumulation models [3] and newer conceptions of affective and identity-based polarization [3-5], positioning our framework more accurately within the broader literature. Accordingly, we have revised the Results and Discussion sections, and paste these revisions as follows.

Revision: [Results in Main Text, Page 4] We note that the echo chamber and backfire effects do not universally occur across all individuals in the real world, with some recent theoretical debates [55, 88, 89]; instead, they exhibit substantial heterogeneity. Extensive experiments reveal that the system autonomously formed by the LLM agents can capture this heterogeneity, showing consistency with empirical observations (SI Section 3.3).

[Discussion in Main Text, Page 6] While this work does not make novel theoretical contributions, **it is grounded in established theories and models of opinion dynamics [58, 62, 100, 101]. In particular, this work follows the argument-accumulation lineage, which explains polarization as arising from the cumulative exchange and reinforcement of persuasive arguments [58, 60, 100, 101]** rather than from direct opinion averaging or social pressure [102, 103]. We further analyze agents' self-reported reasons during opinion formation, observing that agents who become more polarized tend to accumulate substantially more supporting arguments over time (SI Section 3.7). This

observation highlights a clear connection to argument-accumulation models and suggests that the polarization dynamics observed in our synthetic LLM-agent testbed likely stem from similar underlying mechanisms. **At the same time, we acknowledge broader theoretical debates on social media and polarization. Some recent studies have emphasized that echo chamber effects do not universally apply across all individuals in real-world settings, with substantial heterogeneity in exposure to like-minded content [55, 88].** In our experiments, we also observe similar patterns in LLM agents (SI Section 3.3). **Moreover, deliberative and argument-accumulation models have limitations, such as their abstraction from affective responses and identity-based dynamics [104, 105], which have been highlighted in more recent conceptions of polarization.** While our primary analyses focus on opinion-based polarization, we provide supplementary analyses of behavioural patterns consistent with some of these broader dimensions (SI Section 3.7).

[1] Nyhan, Brendan, et al. "Like-minded sources on Facebook are prevalent but not polarizing." Nature 620.7972 (2023): 137-144.
[2] Guess, Andrew, et al. "Avoiding the echo chamber about echo chambers." Knight Foundation 2.1 (2018): 1-25.
[3] Achen, Christopher H., and Larry M. Bartels. "Democracy for realists: Why elections do not produce responsive government." (2017): 1-408.
[4] Mason, Lilliana. Uncivil agreement: How politics became our identity. University of Chicago Press, 2018.
[5] Iyengar, Shanto, et al. "The origins and consequences of affective polarization in the United States." Annual review of political science 22.1 (2019): 129-146.

- Cite/engage with the existing literature on simulating social media using LLMs to position the work in the current wave of GABM research, preventing the impression of overclaiming originality.

Response: Thank you for this valuable suggestion. We have followed it by substantially expanding our engagement with the existing literature on simulating social media using LLM agents. In the revised manuscript, we explicitly situate our study in the current wave of generative agent-based modeling (GABM) research [1-5], and clarify its relationship to prior work. In doing so, we frame our contribution not as introducing a new theory, but as a methodological advance that enables large-scale agents populations to autonomously interact, thereby allowing them to evolve collective opinions and self-organize their social networks at scale. For convenience, we have pasted the corresponding revisions as follows, with important ones highlighted in bold.

Revision: [Introduction in Main Text, Page 2]…On the other hand, **a growing line of work has begun to explore generative agent-based modeling (GABM) using large language model (LLM) agents [3], with particular interest in simulating social networks [39–42], opinion dynamics [43–46], and social media environments [44, 47–50].** Recent studies have shown that LLM agents can be used to generate social network structures that exhibit certain statistical similarities with human networks, such as scale-free degree distributions [39–42], or to simulate specific components of social

media, including opinion feed algorithms [44, 47], coordinated behaviours [50], and echo chamber formation [43]. Despite these advances, existing work faces two key limitations. **First, many studies operate at relatively limited scales in terms of the number of agents, interaction density, or network complexity, which constrains their ability to capture genuinely emergent collective phenomena that arise only from large-scale, repeated interactions [43, 45, 46]. Second, the autonomous social capabilities of LLM agents are largely constrained [45, 46]. Currently, they are primarily treated as tools for specific subtasks, such as eliciting opinions [43] and generating network edges [39], or as puppets heavily scaffolded by pre-defined rules [41, 42]. As a result, these studies offer limited insight into autonomous opinion dynamics and collective behaviour among LLM agents, leaving open the question of how autonomous agents evolve and give rise to emergent social phenomena.**

[Discussion in Main Text, Page 7] **While this work advances prior GABM studies by providing a solid synthetic testbed in which large-scale LLM agents, under minimal social assumptions, can reproduce emergent collective patterns, it also shares limitations common to current GABM research [109–111].** In particular, like prior studies, the proposed system faces natural challenges to external validity. Although results are consistent well-established opinion-dynamics models [51, 53, 58], the extent to which these patterns quantitatively correspond to real-world human behaviour must be further validated. In addition, compared to prior models with transparent mechanisms, GABM comes at the cost of reduced interpretability, as agent behaviour is shaped through prompt-based design and simplified opinion representations. Moreover, the intervention results presented here should be understood as hypothesis-generating. The primary value of the proposed system lies in enabling controlled, pre-experimental exploration of how interaction rules, network structures, and agent-level behaviours may jointly shape collective opinion dynamics. Future research will benefit from complementary human experiments [58, 112], as well as more systematic calibration and evaluation against fine-grained real-world data.

[1] Gao, Chen, et al. “S3: Social-network simulation system with large language model-empowered agents.” arXiv preprint arXiv:2307.14984 (2023).
[2] Gu, Chenhao, et al. “Large Language Model Driven Agents for Simulating Echo Chamber Formation.” arXiv preprint arXiv:2502.18138 (2025).
[3] Orlando, Gian Marco, et al. “Emergent Coordinated Behaviors in Networked LLM Agents: Modeling the Strategic Dynamics of Information Operations.” arXiv preprint arXiv:2510.25003 (2025).
[4] Törnberg, Petter, et al. “Simulating social media using large language models to evaluate alternative news feed algorithms.” arXiv preprint arXiv:2310.05984 (2023).
[5] Larooij, Maik, and Petter Törnberg. “Validation is the central challenge for generative social simulation: a critical review of LLMs in agent-based modeling.” Artificial Intelligence Review 59.1 (2025): 15.

Implementing these limited textual changes would, in my view, adequately temper

the paper's claims and address its main overreach without imposing significant additional workload on the authors.

Response: We thank you for outlining this clear path forward and confirm that we have implemented all these textual changes, which we agree effectively temper the claims and address the overreach as detailed above.

My full original review report follows below, providing further detail and context for these comments.

This manuscript presents a model of interacting LLMs, aiming to simulate networks of online bots engaged in influence campaigns. The authors argue that such artificial societies exhibit many properties analogous to human opinion dynamics — including homophily, echo chambers, confirmation bias, and backfire effects. While the topic is timely and potentially impactful, the paper in its current form falls well short of the conceptual, methodological, and interpretive rigor expected of a Nature Communications publication. The manuscript is rhetorically ambitious but analytically underdeveloped. As it stands, the work risks overstating its contributions and misrepresenting the epistemic status of its findings.

Response: Thank you for recognizing that the topic is timely and potentially impactful. We agree with you that the original version of the manuscript suffered from imprecise framing and claims, which risked obscuring the intended scope and contributions of the work. Guided by your valuable suggestions and the associated checklist above, we have substantially revised the manuscript to improve its conceptual clarity, methodological positioning, and interpretive rigor. In particular,

(i) **Framing and scope**. First, we have removed all claims suggesting that the system models online bot networks or LLM agents operating "in the wild," as well as any implications of policy guidance or direct deployment, and consistently reframed the work as a synthetic LLM-agent testbed for pre-experimental exploration of opinion-dynamics hypotheses. This clarification sharpens the manuscript's framing and avoids overstatement of real-world applicability.

(ii) **Theoretical positioning.** Second, we have removed claims of theoretical contribution and now explicitly acknowledge the argument-accumulation modeling lineage, clarifying that the observed dynamics align with established opinion-dynamics models and likely stem from similar underlying mechanisms. In addition, we now explicitly engage with key theoretical debates in the literature on polarization, including critiques of the echo-chamber thesis, limitations of deliberative and argument-accumulation models, and newer conceptions of polarization such as affective and identity-based polarization.

(iii) **Relation to prior work.** Third, to prevent any impression of overclaiming originality, we have expanded and deepened our engagement with the recent

GABM literature, explicitly positioning our work within the current wave of research on LLM-based generative agent systems for simulating social networks, social media environments, and opinion dynamics. We also now explicitly acknowledge ongoing debates in the GABM field regarding challenges of validation and calibration, and the resulting limits on external validity.

(iv) **Language and interpretation.** Finally, we have tightened the language throughout the manuscript, softening or removing terms such as "human-like", "non-trivial", and "remarkable", and carefully qualifying references to psychological and social mechanisms using formulations such as "behaviour consistent with X", explicitly noting when such inferences are drawn from LLM self-reports.

Taken together, these revisions substantially reduce rhetorical overreach and more clearly articulate the manuscript's intended contribution as a synthetic testbed that enables large-scale agents to autonomously interact, thereby allowing them to evolve collective opinions and self-organize their social networks.

# Conceptual Framing and Contribution

The manuscript claims that the model represents online bot networks. However, this framing appears primarily as a rhetorical strategy to avoid engaging with two fundamental challenges in generative agent-based modeling (GABM): (1) demonstrating external validity and (2) offering a substantive social scientific contribution. The result is a model that is neither a convincing representation of bot networks nor a useful tool for social science research. Ultimately, it becomes yet another paper suggesting that LLMs exhibit "human-like" behavior, without offering the necessary theoretical or empirical work to make such claims meaningful.

## What is the paper contributing to?

The GABM literature currently faces two central challenges: (1) understanding how such models can contribute to social science knowledge, and (2) demonstrating their external validity. These are important but difficult questions. This paper, however, largely sidesteps them by describing its model as a simulation of interacting LLM-based bots, and by suggesting that understanding LLM opinion dynamics is essential for understanding coordinated influence networks. This move effectively allows the authors to claim that the model is a model of little more than itself, thereby bypassing the need to establish external validity.

This framing is difficult to justify. There is little reason to believe that bots designed for political influence would develop "opinions" by generating arguments and updating positions through deliberation. Designing such a system would be highly implausible as a real-world influence strategy.

Moreover, the paper's own analyses appear inconsistent with this framing. Much of the argumentation implicitly treats the model as simulating human behavior rather than bot activity. For example, the justification that "people develop opinions over time, so the model should too" is incompatible with a model intended to represent

automated bot networks. This internal inconsistency undermines the clarity and credibility of the paper's contribution.

The manuscript further oscillates between denying that it models human behavior and implying that it does. It repeatedly invokes the notion of being "human-like," but does not provide the theoretical or empirical basis for making such a claim. Statements such as "these experiments provide guidance regarding how to directly intervene in the growing world of LLM agents operating in the wild" are particularly problematic, as they rely on a premise that is neither substantiated nor credible within the paper's framework.

Response: Thank you for raising these important concerns regarding the conceptual framing of the manuscript. We apologize for the imprecise language used in the earlier version, in which terms such as "bot networks" and "LLM agents operating in the wild" were used to motivate the study. This wording unintentionally created the impression that we were modeling real-world bot networks for political influence, thereby bypassing the need to address external validity. This was not our intention. As you insightfully note, our goal is not to model real-world bot networks, but rather to contribute a synthetic testbed of large-scale LLM agents for the pre-experimental exploration of opinion-dynamics hypotheses. To address the concerns you raise, we have substantially revised the manuscript in the following ways:

(i) Removal of the bot-network framing. We have removed all claims that the model represents online bot networks or LLM agents operating "in the wild." The manuscript is now consistently framed as a synthetic LLM-agent testbed, which clarifies the intended scope of the work and avoids conflating the system with real-world political actors or influence campaigns.

Revision: [Abstract in Main Text, Page 1] …Similarities between humans and LLM agents —encompassing behaviours, patterns, and emergent phenomena — not only offer a valuable **synthetic testbed** for generating hypothetical strategies to mitigate polarization and its consequences…

[Introduction in Main Text, Page 1] Besides these evident misbehaviours, **LLMs may pose broader risks in more subtle ways.** Numerous studies have pointed out that LLMs can generate persuasive political content [21–23], potentially affecting human opinions and behaviours [13, 23–25]. More crucially, due to the increasing indistinguishability of content generated by LLMs from that created by humans [4, 21, 22, 24, 26], concerns arise over their potential to manipulate public opinions and intensify polarization as the scale of interaction increases [24, 25, 27]. ~~**Indeed, this concern over digital manipulation of public opinions, which has amplified over the past decade [28–32], has been drastically exacerbated by the advent of LLM agents. During the 2016 US presidential election, automated agents, then known primarily as bots, were widely deployed on social media and suspected of interfering with electoral outcomes [28]. In the same year, Tay, a continuously learning bot released by Microsoft, was contaminated by biased human-generated content, resulting in offensive discourse**~~

~~**and removal from the Internet within hours of its release [33]. Driven by simple rules, bots lacked human-level intelligence [28, 33], but their societal impacts remained profound, raising a pressing question: what if they were empowered by LLMs [4, 22, 24]?**~~

[Introduction in Main Text, Page 2] In this work, we simulate **a synthetic LLM-agent system,** where thousands of agents, solely driven by LLMs… the system we propose can constitute **a valuable pre-experimental testbed** for exploring hypothetical strategies to reduce polarization and promote more inclusive political conversations [30]… Overall, this work not only sheds light on subtle opinion dynamics and collective behaviour of LLM agents from a new network perspective, but it also unveils **their potential to serve as a testbed for the pre-experimental exploration of opinion-dynamics hypotheses.**

[Results in Main Text, Page 2] Opinion dynamics in a **synthetic** system of interacting LLM agents.

[Introduction in Main Text, Page 5] **Synthetic testbed for generating hypothetical strategies to reduce polarization.**

[Results in Main Text, Page 6] Despite the difficulty of recommending openness to human populations, these results suggest that **in our synthetic testbed**, access and open-mindedness to diverse opinions at the individual level appear more effective than altering social networks in polarized systems. . . In all, these experiments not only **generate several promising hypothetical strategies** but also demonstrate **the proposed system's potential as a valuable testbed** for piloting social experiments. **(~~Furthermore, these experiments provide guidance regarding how to directly intervene in the growing world of LLM agents operating in the wild.~~)**

[Discussion in Main Text, Page 6] Building on these findings, our work shifts the perspective from isolated LLMs [29, 30, 32, 35–38] to complex, networked LLM agent societies, offering **a synthetic testbed for** studying emergent collective dynamics in artificial agents (~~**and their potential interactions with human societies**~~**).**

[Discussion in Main Text, Page 7] (~~On the other hand, LLM agents also raise concerns about their potential risks to human society. As studied in prior work [4, 21, 22, 24–26], LLMs are not only indistinguishable from humans but may also be more persuasive. Moreover, attempts to incorporate LLM agents into real-world political deliberations have demonstrated their surprising influence, with evidence indicating that they can drive significant outcomes even when functioning independently [35, 36, 43]. This is naturally concerning and raises fundamental questions with which modern societies and polities must grapple. What are the consequences of releasing LLMs with increasing autonomy, indistinguishable from humans, and with access to online social networks? How will such agents interplay with human social ties, be conflated with real friends, augment political persuasion, and impact our opinion dynamics? Moreover, unlike traditional bots [28–32], these LLM agents can evolve, gradually self-organizing their own collective opinions and social networks. The consequences of integrating such LLM agents at scale into human~~

~~social networks are unpredictable and, in our view, still beyond our control. This requires us to rigorously evaluate potential risks, strengthen safeguards, and conduct cautious testing before deploying them "in the wild". First, we should systematically evaluate and mitigate biases in LLMs that drive agents to ensure fairness, reliability, and prevent unintended reinforcement of societal bias. This requires advancing standardized benchmarks, auditing frameworks, and bias mitigation strategies [17, 69, 70, 92, 93, 101]. Second, detection methods targeting LLM agents need further investigation to prevent them from engaging in deceptive or manipulative behaviour or disrupting human social media platforms [102]. Third, future work should extend experiments to a mixed population of both agents and humans, which could further facilitate more rigorous examination of LLM influence on human behaviours and society. Finally, because the collective nature of agents gives rise to emergent collective opinions and behaviours that cannot be easily inferred from individual agents, we believe that our proposed networked simulation experiments serve as a useful pre-deployment test for LLM agents. To further strengthen the rigor of such evaluations, future work should consider developing a controlled experimental platform that enables testing with mixed populations of LLM agents and humans.~~)

(ii) Explicit clarification of external-validity limitations. We now more carefully situate the work in the broader GABM literature, explicitly acknowledging ongoing debates about validation, calibration, and the inherent challenges of establishing external validity when simulating human behaviour. Moreover, we make a deliberate effort to engage with established theories and models of opinion dynamics, while maintaining careful statements by using formulations such as "behaviour consistent with X" and specifying when these are inferred from LLM self-reports.

Revision: [Discussion in Main Text, Page 7] While this work advances prior GABM studies by providing a solid synthetic testbed in which large-scale LLM agents, under minimal social assumptions, can reproduce emergent collective patterns, **it also shares limitations common to current GABM research [109–111]. In particular, like prior studies, the proposed system faces natural challenges to external validity**. Although results are consistent well-established opinion-dynamics models [51, 53, 58], **the extent to which these patterns quantitatively correspond to real-world human behaviour must be further validated.** In addition, compared to prior models with transparent mechanisms, GABM comes at the cost of reduced interpretability, as agent behaviour is shaped through prompt-based design and simplified opinion representations. Moreover, the intervention results presented here should be understood as hypothesis-generating. The primary value of the proposed system lies in enabling controlled, pre-experimental exploration of how interaction rules, network structures, and agent-level behaviours may jointly shape collective opinion dynamics. Future research will benefit from complementary human experiments [58, 112], **as well as more systematic calibration and evaluation against fine-grained real-world data.**
[Abstract in Main Text, Page 1] ... We discover that these agents spontaneously develop their own social network **with properties characteristic of human social networks**, including homophilic clustering. **The collective opinions of these LLM agents evolve in**

ways that exhibit behavioural patterns consistent with social phenomena and mechanisms widely discussed in studies of human behaviour…

[Introduction in Main Text, Page 2] The self-organized networks that result, in turn, shape their collective opinions **in ways that exhibit behavioural patterns consistent with social phenomena and associated mechanisms initially identified in human social networks [43, 45, 47, 50].**

[Introduction in Main Text, Page 2] Shifting focus from the network to individuals, we further examine **whether agents' behaviours align with a wide range of social mechanisms contributing to real-world polarization,** e.g., selective exposure [51], confirmation bias [52], and elite signaling [48], finding their effects on LLM agents align with **those reported in studies of human behaviour.**

[Introduction in Main Text, Page 2] Rather, it originates from the similarities between humans and LLM agents, encompassing the social networks they self-organize, the collective opinions they develop, **and the interaction processes through which these dynamics unfold.**

[Results in Main Text, Page 4] **(~~Mechanisms underlying the emergence of human-like~~) Collective behavioural patterns behind the emergence of** polarization among LLM agents.

[Results in Main Text, Page 4] This indicates that **LLM agents exhibit behavioural patterns consistent with the echo chamber effect**… Instead, exposure to agents with opposing opinions could potentially increase polarization levels, indicating **behavioural patterns consistent with the backfire effect [45].**

[Results in Main Text, Page 5] Rather, agents' **behavioural patterns consistent with these mechanisms** naturally emerge from agents' social interactions. For example, analyzing the **self-reported reasons** generated during opinion formation reveals **patterns in line with** confirmation bias (SI Section 3.7).

[Results in Main Text, Page 5] Given these observed similarities between LLM agents and humans at the network level, one may wonder whether LLM agents **exhibit behaviours consistent with psychological mechanisms discussed in studies of human opinion formation**. To answer this question, we design a comparative experiment: we **vary the proportion of agents exhibiting behaviours consistent with selective exposure or confirmation bias, and then examine the resulting changes** in the level of polarization. Cases where 0% or 100% of agents **are configured to exhibit these behaviours** are deliberately designed as extreme scenarios for comparative purposes. As shown in Figure 3e and f, we observe that the increase in agents **exhibiting either behaviour leads to a marked increase in the level of polarization characterizing the overall system**. **This suggests that behaviours related to selective exposure and confirmation bias** contribute to the polarization of LLM agents, **consistent with findings reported in studies of human behaviour** [48, 52, 79].

[Results in Main Text, Page 5] These agents **exhibit behaviours consistent with social phenomena and associated mechanisms discussed in studies of human behaviour, at both the network and individual levels.**

[Discussion in Main Text, Page 6] Specifically, from the individual perspective, opinion formation among LLM agents exhibits **behavioural patterns consistent with social and psychological mechanisms** [51, 52], e.g., selective exposure, confirmation bias, elite signaling, etc. From the collective perspective, LLM agents organically develop **social networks with properties characteristic of human social networks**, e.g., homophilic clustering and scale-free degree distributions [28]. Moreover, their self-developed social networks, in turn, shape LLM collective opinions **in ways consistent with** the echo chamber and backfire effects described in studies of human behaviour, resulting in the emergence of opinion polarization [43–48, 81, 85, 86]. Notably, all **these behavioural patterns**, network organization, and collective opinions naturally emerge from interactions among agents completely driven by LLMs.

[Discussion in Main Text, Page 6] Specifically, from the individual perspective, opinion formation among LLM agents exhibits **behavioural patterns consistent with social and psychological mechanisms** [51, 52], e.g., selective exposure, confirmation bias, elite signaling, etc. From the collective perspective, LLM agents organically develop **social networks with properties characteristic of human social networks**, e.g., homophilic clustering and scale-free degree distributions [28]. Moreover, their self-developed social networks, in turn, shape LLM collective opinions **in ways consistent with** the echo chamber and backfire effects described in studies of human behaviour, resulting in the emergence of opinion polarization [43–48, 81, 85, 86]. Notably, all **these behavioural patterns**, network organization, and collective opinions naturally emerge from interactions among agents completely driven by LLMs.

[Section 3.3 in Supplementary Information, Pages 15-16] We also incorporate more **standard measures of behaviours commonly associated with "echo chambers" and "backfire effects" following prior research** [9, 4]… As shown in Figure S10, we observe that agents are more likely to interact with peers in the same camp (left-leaning or right-leaning), **demonstrating behavioural patterns consistent with echo chambers**… Echo chambers do not universally occur across all individuals in the real world…Specifically, we find that **patterns of interaction consistent with echo chambers do not universally occur across all agents.**

[Section 3.3 in Supplementary Information, Pages 17] Similarly, the presence and extent of backfire effects remain subjects of debate, and such effects do not appear universally…Furthermore, **behavioural patterns consistent with the backfire effect** are observed in only a small portion of agents (0.82%–2.12%)…

Taken together, these revisions clarify the conceptual framing of the proposed system, sharpen its positioning, and make explicit both its limitations and its intended role as a synthetic, hypothesis-generating testbed.

## Social Science Contribution
Even if one were to set aside the bot-network framing and treat the model as one of human behavior — and further set aside the requirement for external validity — the paper would still not constitute a meaningful contribution.

I am not a skeptic of generative agent-based models. They hold significant promise for advancing our understanding of social dynamics, despite their challenges around explainability and interpretability. However, this manuscript does not make use of that potential. Instead, it re-implements a well-known class of models in which agents accumulate arguments and become polarized, adding little beyond replacing transparent mechanisms with opaque LLM outputs. The primary result is a version of an established opinion dynamics model that is substantially less interpretable.

In terms of its treatment of opinion divergence ("polarization"), the model belongs to the argument-accumulation family (e.g., Mäs & Flache, 2013, JASSS). To make a substantive social science contribution, the paper would need to go beyond reproducing familiar dynamics and instead reveal new processes that can only be detected using LLM-based models.

The manuscript also engages with the social science literature in only a superficial way. Concepts such as polarization, homophily, and confirmation bias are treated as straightforward empirical regularities rather than contested theoretical constructs. This leads to a selective and often outdated use of theory. The "echo chamber" model of polarization — based on selective exposure, ideological extremity, and argument accumulation — has been widely criticized. For example:

1) Polarization is simplistically defined as movement away from neutrality, overlooking more recent work on affective and sectarian polarization and the role of identity.
2) The prevalence of echo chambers has been challenged by empirical research suggesting they are less common than once believed.
3) The argument-accumulation approach rests on a Habermasian model of politics that has been extensively critiqued (e.g., Achen & Bartels, Democracy for Realists). If anything, the fact that the model reproduces such outdated theories may be interesting in itself - but it is questionable whether this should be described as "human-like."

Response: Thank you for this critique of the manuscript's social-scientific initial social science framing and contribution. We agree that the original version of the paper risked overstating its social science contribution. In the revised manuscript, we have therefore taken special care to substantially clarify both the nature and the scope of our contribution.

(i) Positioning in the argument-accumulation lineage. We explicitly acknowledge that the polarization dynamics observed in our system fall in the well-established argument-accumulation lineage of opinion-dynamics models [1-4]. We do not present these dynamics as novel social mechanisms, nor do we suggest that they con new social theory. Instead, we clearly state that the observed patterns align with existing models and are likely driven by similar underlying processes.

Revision: [Introduction in Main Text, Page 2] …These phenomena, such as polarization and homophilic clustering, underscore how networked interactions transform otherwise isolated LLMs into a collectively evolving system with emergent dynamics. **Importantly, rather than proposing a new theory of polarization, our results align with established opinion-dynamics models, particularly the argument-accumulation lineage [58, 60, 62], where polarization emerges from cumulative exchanges of persuasive arguments under homophilic interactions**…

[Results in Main Text, Page 5]…**Importantly, these results align with established argument-accumulation models in opinion dynamics, where polarization emerges from the cumulative exchange and reinforcement of persuasive arguments under homophilic interactions [58, 62], as reflected in our analysis of agents' self-reported reasons (SI Section 3.7)**…

[Discussion in Main Text, Page 6] While this work does not make novel theoretical contributions, it is **grounded in established theories and models of opinion dynamics [58, 62, 100, 101]**. In particular, this work **follows the argument-accumulation lineage**, which explains polarization as arising from the cumulative exchange and reinforcement of persuasive arguments [58, 60, 100, 101] rather than from direct opinion averaging or social pressure [102, 103]. **We further analyze agents' self-reported reasons during opinion formation, observing that agents who become more polarized tend to accumulate substantially more supporting arguments over time (SI Section 3.7). This observation highlights a clear connection to argument-accumulation models and suggests that the polarization dynamics observed in our synthetic LLM-agent testbed likely stem from similar underlying mechanisms**…

[Section 3.7 in SI, Page 33] To scrutinize the connection between the polarization dynamics observed in our synthetic LLM-agent testbed and the established argument-accumulation lineage in opinion-dynamics research [28, 15], we analyze how agents' self-reported supporting reasons evolve as their opinions become more polarized. Specifically, we compare the supporting reasons generated by agents before and after adopting more polarized opinions. We use a LLM to extract distinct arguments contained in these self-reported reasons. As shown in Figure S32a, agents who become more polarized tend to accumulate a significantly larger number of supporting arguments over time across all three topics. Figure S32b–d further report the most frequently occurring argument themes for each topic after polarization. The distribution of popular arguments varies across topics, reflecting issue-specific reasoning patterns. Overall, these findings provide additional evidence that the results in the synthetic system are consistent with argument-accumulation models.

(ii) Clarifying the methodological contribution. We have revised the manuscript to clarify that its primary contribution is methodological rather than theoretical. The goal of the proposed system is not to introduce new mechanisms of polarization, but to provide a large-scale synthetic testbed in which LLM agents autonomously interact under minimal social assumptions. This enables the instantiation and exploration of known opinion-dynamics mechanisms in a setting where reasoning, language, interaction patterns, and network structure co-evolve endogenously, rather than being hard-coded or externally imposed. We emphasize that this comes at the cost of interpretability relative to stylized models, a limitation we now explicitly acknowledge.

Revision: [Abstract in Main Text, Page 1] …Similarities between humans and LLM agents—encompassing behaviours, patterns, and emergent phenomena—not only offer a valuable **synthetic testbed for generating hypothetical strategies to** mitigate polarization and its consequences, …

[Introduction in Main Text, Page 2] In this work, we simulate **a synthetic LLM-agent system,** where thousands of agents, solely driven by LLMs… the system we propose can constitute **a valuable pre-experimental testbed for exploring hypothetical strategies** to reduce polarization and promote more inclusive political conversations [30]… Overall, this work not only sheds light on subtle opinion dynamics and collective behaviour of LLM agents from a new network perspective, but it also unveils **their potential to serve as a testbed for the pre-experimental exploration of opinion-dynamics hypotheses.**

[Results in Main Text, Page 5] **Synthetic testbed for generating hypothetical strategies to reduce polarization.**

[Results in Main Text, Page 5] These similarities make **the proposed LLM-agent system a valuable synthetic testbed for generating hypothetical strategies to** intervene in polarization and its consequences.

[Results in Main Text, Page 5] To explore it, we accordingly design five intervention strategies **in our synthetic testbed** (Figure 4)…Indeed, these interventions **are motivated by established lines of prior work and are used here as experimental interventions in our synthetic system**: MOI draws on studies of exposure to cross-cutting information [76, 82]; RI operationalizes randomized interaction; NSE and NCB relate to promoting open-mindedness [83]; and NES is inspired by the design of Bail et al. [84].

[Results in Main Text, Page 6] To explore it, we accordingly design five intervention strategies **in our synthetic testbed** (Figure 4)…Indeed, these interventions **are motivated by established lines of prior work and are used here as experimental interventions in our synthetic system**: MOI draws on studies of exposure to cross-cutting information [76, 82]; RI operationalizes randomized interaction; NSE and NCB relate to promoting open-mindedness [83]; and NES is inspired by the design of Bail et al. [84].

[Results in Main Text, Page 6] Despite the difficulty of recommending openness to human populations, these results suggest that **in our synthetic testbed**, access and open-mindedness to diverse opinions at the individual level appear more effective than altering social networks in polarized systems. . . In all, these experiments not only **generate several promising hypothetical strategies** but also demonstrate **the proposed system's potential as a valuable testbed** for piloting social experiments.

[Discussion in Main Text, Page 6] While this work **does not make novel theoretical contributions**, it is grounded in established theories and models of opinion dynamics [58, 62, 100, 101]. In particular, this work follows the argument-accumulation lineage, which explains polarization as arising from the cumulative exchange and reinforcement of persuasive arguments [58, 60, 100, 101]…

[Discussion in Main Text, Page 7]…**In addition, compared to prior models with transparent mechanisms, GABM comes at the cost of reduced interpretability**…

(iii) Engaging with theoretical debates. Third, we now engage more carefully with ongoing theoretical debates in the social science literature on polarization. In particular, we explicitly acknowledge critiques of the echo-chamber thesis [5-6], limitations of deliberative and argument-accumulation models [7], and more recent conceptions of polarization that emphasize affective and identity-based dimensions [7-9]. Throughout the revised manuscript, we avoid treating concepts such as polarization, homophily, or confirmation bias as settled empirical facts. Instead, we use formulations such as "behaviour consistent with X," and clearly note when such interpretations are inferred from LLM self-reports. Importantly, reproducing dynamics associated with these theories is not presented as validating them, but as situating the synthetic system in the longstanding body of social-scientific research on polarization.

Revision: [Results in Main Text, Page 4] We note that the echo chamber and backfire effects do not universally occur across all individuals in the real world, with some recent theoretical debates [55, 88, 89]; instead, they exhibit substantial heterogeneity. Extensive experiments reveal that the system autonomously formed by the LLM agents can capture this heterogeneity, showing consistency with empirical observations (SI Section 3.3).

[Discussion in Main Text, Page 6] While this work does not make novel theoretical contributions, **it is grounded in established theories and models of opinion dynamics [58, 62, 100, 101]. In particular, this work follows the argument-accumulation lineage, which explains polarization as arising from the cumulative exchange and reinforcement of persuasive arguments [58, 60, 100, 101]** rather than from direct opinion averaging or social pressure [102, 103]. We further analyze agents' self-reported reasons during opinion formation, observing that agents who become more polarized tend to accumulate substantially more supporting arguments over time (SI Section 3.7). This observation highlights a clear connection to argument-accumulation models and suggests that the polarization dynamics observed in our synthetic LLM-agent testbed likely stem from similar underlying mechanisms. **At the same time, we acknowledge broader**

theoretical debates on social media and polarization. Some recent studies have emphasized that echo chamber effects do not universally apply across all individuals in real-world settings, with substantial heterogeneity in exposure to like-minded content [55, 88]. In our experiments, we also observe similar patterns in LLM agents (SI Section 3.3). **Moreover, deliberative and argument-accumulation models have limitations, such as their abstraction from affective responses and identity-based dynamics [104, 105], which have been highlighted in more recent conceptions of polarization.** While our primary analyses focus on opinion-based polarization, we provide supplementary analyses of behavioural patterns consistent with some of these broader dimensions (SI Section 3.7).

[Abstract in Main Text, Page 1] ... We discover that these agents spontaneously develop their own social network **with properties characteristic of human social networks**, including homophilic clustering. **The collective opinions of these LLM agents evolve in ways that exhibit behavioural patterns consistent with social phenomena and mechanisms widely discussed in studies of human behaviour…**

[Introduction in Main Text, Page 2] The self-organized networks that result, in turn, shape their collective opinions **in ways that exhibit behavioural patterns consistent with social phenomena and associated mechanisms initially identified in human social networks [43, 45, 47, 50].**

[Introduction in Main Text, Page 2] Shifting focus from the network to individuals, we further examine **whether agents' behaviours align with a wide range of social mechanisms contributing to real-world polarization,** e.g., selective exposure [51], confirmation bias [52], and elite signaling [48], finding their effects on LLM agents align with **those reported in studies of human behaviour.**

[Introduction in Main Text, Page 2] Rather, it originates from the similarities between humans and LLM agents, encompassing the social networks they self-organize, the collective opinions they develop, **and the interaction processes through which these dynamics unfold.**

[Results in Main Text, Page 4] **(~~Mechanisms underlying the emergence of human-like~~) Collective behavioural patterns behind the emergence of** polarization among LLM agents.

[Results in Main Text, Page 4] This indicates that **LLM agents exhibit behavioural patterns consistent with the echo chamber effect**… Instead, exposure to agents with opposing opinions could potentially increase polarization levels, indicating **behavioural patterns consistent with the backfire effect [45].**

[Results in Main Text, Page 5] Rather, agents' **behavioural patterns consistent with these mechanisms** naturally emerge from agents' social interactions. For example, analyzing the **self-reported reasons** generated during opinion formation reveals **patterns in line with** confirmation bias (SI Section 3.7).

[Results in Main Text, Page 5] Given these observed similarities between LLM agents and humans at the network level, one may wonder whether LLM agents **exhibit behaviours consistent with psychological mechanisms discussed in studies of human opinion formation**. To answer this question, we design a comparative experiment: we **vary the proportion of agents exhibiting behaviours consistent with selective exposure or confirmation bias, and then examine the resulting changes** in the level of polarization. Cases where 0% or 100% of agents **are configured to exhibit these behaviours** are deliberately designed as extreme scenarios for comparative purposes. As shown in Figure 3e and f, we observe that the increase in agents **exhibiting either behaviour leads to a marked increase in the level of polarization characterizing the overall system**. **This suggests that behaviours related to selective exposure and confirmation bias** contribute to the polarization of LLM agents, **consistent with findings reported in studies of human behaviour** [48, 52, 79].

[Results in Main Text, Page 5] These agents **exhibit behaviours consistent with social phenomena and associated mechanisms discussed in studies of human behaviour, at both network and individual levels.**

[Discussion in Main Text, Page 6] Specifically, from the individual perspective, opinion formation among LLM agents exhibits **behavioural patterns consistent with social and psychological mechanisms** [51, 52], e.g., selective exposure, confirmation bias, elite signaling, etc. From the collective perspective, LLM agents organically develop **social networks with properties characteristic of human social networks**, e.g., homophilic clustering and scale-free degree distributions [28]. Moreover, their self-developed social networks, in turn, shape LLM collective opinions **in ways consistent with** the echo chamber and backfire effects described in studies of human behaviour, resulting in the emergence of opinion polarization [43–48, 81, 85, 86]. Notably, all **these behavioural patterns**, network organization, and collective opinions naturally emerge from interactions among agents completely driven by LLMs.

[Discussion in Main Text, Page 6] Specifically, from the individual perspective, opinion formation among LLM agents exhibits **behavioural patterns consistent with social and psychological mechanisms** [51, 52], e.g., selective exposure, confirmation bias, elite signaling, etc. From the collective perspective, LLM agents organically develop **social networks with properties characteristic of human social networks**, e.g., homophilic clustering and scale-free degree distributions [28]. Moreover, their self-developed social networks, in turn, shape LLM collective opinions **in ways consistent with** the echo chamber and backfire effects described in studies of human behaviour, resulting in the emergence of opinion polarization [43–48, 81, 85, 86]. Notably, all **these behavioural patterns**, network organization, and collective opinions naturally emerge from interactions among agents completely driven by LLMs.

[Section 3.3 in Supplementary Information, Pages 15-16] We also incorporate more **standard measures of behaviours commonly associated with "echo chambers" and "backfire effects" following prior research** [9, 4]… As shown in Figure S10, we

observe that agents are more likely to interact with peers in the same camp (left-leaning or right-leaning), **demonstrating behavioural patterns consistent with echo chambers**… Echo chambers do not universally occur across all individuals in the real world…Specifically, we find that **patterns of interaction consistent with echo chambers do not universally occur across all agents.**

[Section 3.3 in Supplementary Information, Pages 17] Similarly, the presence and extent of backfire effects remain subjects of debate, and such effects do not appear universally…Furthermore, **behavioural patterns consistent with the backfire effect** are observed in only a small portion of agents (0.82%–2.12%)…

Taken together, these revisions reposition the manuscript away from claims of theoretical novelty or human-like realism, and toward a more modest and transparent but also, we argue, important contribution: a synthetic, hypothesis-generating testbed that allows researchers to examine how familiar opinion-dynamics patterns may arise in large-scale, autonomous LLM-agent societies under a wide range of controlled and artificial conditions.

[1] Mäs, Michael, and Andreas Flache. "Differentiation without distancing. Explaining bi-polarization of opinions without negative influence." PloS one 8.11 (2013): e74516.
[2] Flache, Andreas, et al. "Models of social influence: Towards the next frontiers." Jasss-The journal of artificial societies and social simulation 20.4 (2017): 2.
[3] Banisch, Sven, and Hawal Shamon. "Biased processing and opinion polarization: experimental refinement of argument communication theory in the context of the energy debate." Sociological Methods & Research 54.1 (2025): 187-236.
[4] Banisch, Sven, and Eckehard Olbrich. "An argument communication model of polarization and ideological alignment." The journal of artificial societies and social simulation 24.1 (2021).
[5] Nyhan, Brendan, et al. "Like-minded sources on Facebook are prevalent but not polarizing." Nature 620.7972 (2023): 137-144.
[6] Guess, Andrew, et al. "Avoiding the echo chamber about echo chambers." Knight Foundation 2.1 (2018): 1-25.
[7] Achen, Christopher H., and Larry M. Bartels. "Democracy for realists: Why elections do not produce responsive government." (2017): 1-408.
[8] Mason, Lilliana. Uncivil agreement: How politics became our identity. University of Chicago Press, 2018.
[9] Iyengar, Shanto, et al. "The origins and consequences of affective polarization in the United States." Annual review of political science 22.1 (2019): 129-146.

## Insights into LLM Behavior
The paper might still have offered value if it revealed unexpected aspects of LLM behavior. However, it does not. The reported results, such as homophilic clustering, are entirely predictable. They could be anticipated from existing models of opinion dynamics or simple network processes, combined with the known behavior of LLMs when prompted about social interaction. These patterns are particularly

unsurprising given that LLMs are trained on corpora containing precisely these theoretical constructs and the literature that discusses them.

It is already well established that LLMs can mimic human-like responses. This finding is neither novel nor informative. Furthermore, issues such as training data leakage prevent such mimicry from being treated as evidence of emergent social mechanisms. Despite this, the manuscript frequently overstates its claims — for instance, describing LLMs' ability to generate network structures as "remarkable" when such structures can be produced by extremely simple models. Similarly, describing homophilic clustering as a "non-trivial phenomenon" is misleading; it is an expected outcome.

Response: We thank the reviewer for highlighting concerns regarding potential overstatement in the description of observed patterns. In response, we have carefully revised the manuscript to tighten the language throughout the manuscript. Specifically, we have removed or softened terms such as "human-like", "remarkable," and "non-trivial", and now consistently qualify observed patterns using formulations such as "behaviour consistent with X," explicitly indicating when such characterizations are inferred from LLM self-reports rather than treated as evidence of underlying social mechanisms. These revisions ensure that the presentation remains precise and avoids rhetorical overreach.

Moreover, while it is well established that LLMs can sometimes mimic human-like responses some of the time, our reproducible and open source platform simulates thousands of agents in tens of thousands of interactions, which stably converge to very similar outcomes hundreds of times through the same observed and stated mechanisms. Our represents an effort to increase our confidence in the robust replicability of GABM findings. For example, the ablation studies we introduce enable causal identification within GABMs that would, in some cases, be complex and even unethical to experiment with in the real world at scale. In short, our work attempts—more effectively with the benefit of your comments and more focused framing—to turn the uneven insights from LLM human mimicry into a reproducible simulation platform that enables inexpensive exploration prior to more expensive human observation and experiment. Our feedback from the social science community (through talks at Harvard, Stanford, Duke, University of Chicago, Tsinghua, Fudan, Hong Kong University, and many others) is that this is a useful and valuable intervention as a pre-experimental simulation environment. We are grateful for your engaged and constructive comments.

Revision: [Title in Main Text, Page 1] Spontaneous emergence of opinion polarization among large language model agents

[Abstract in Main Text, Page 1] ...In this paper, we simulate a networked system involving thousands of LLM agents, discovering that their social interactions, guided through LLM conversation, **result in the emergence of opinion polarization**. We discover that these agents spontaneously develop their own social network **with properties characteristic of human social networks**, including homophilic clustering.

The collective opinions of these LLM agents evolve in ways that exhibit behavioural patterns consistent with social phenomena and mechanisms widely discussed in studies of human behaviour…

[Introduction in Main Text, Page 2]…the **(~~remarkable~~)** capability of LLMs in technical network generation

[Introduction in Main Text, Page 1] Meanwhile, LLM agents spontaneously organize their own social network with **properties characteristic of human social networks**: agents with homophilic opinions tend to cluster, while those with opposing opinions tend to avoid interactions [28]…

[Introduction in Main Text, Page 2] These (**~~non-trivial~~)** phenomena, such as polarization and homophilic clustering, …

[Results in Main Text, Page 3] (**~~Human-like polarization~~) Polarization patterns** emerge from self-regulated LLM agents.

[Discussion in Main Text, Page 6] This work uncovers that LLM agents, **(~~sometimes considered a new species "Homo silicus" [12]~~)**, exhibit **(~~human-like~~)** polarization patterns on political issues, at both individual and collective levels.

[Section 3.11 in Supplementary Information, Page 44] We find that despite substantial differences in initial network structure, the final outcomes are (**~~remarkably~~)** similar.

[Abstract in Main Text, Page 1] ... We discover that these agents spontaneously develop their own social network **with properties characteristic of human social networks**, including homophilic clustering. **The collective opinions of these LLM agents evolve in ways that exhibit behavioural patterns consistent with social phenomena and mechanisms widely discussed in studies of human behaviour…**

[Introduction in Main Text, Page 2] The self-organized networks that result, in turn, shape their collective opinions **in ways that exhibit behavioural patterns consistent with social phenomena and associated mechanisms initially identified in human social networks [43, 45, 47, 50].**

[Introduction in Main Text, Page 2] Shifting focus from the network to individuals, we further examine **whether agents' behaviours align with a wide range of social mechanisms contributing to real-world polarization,** e.g., selective exposure [51], confirmation bias [52], and elite signaling [48], finding their effects on LLM agents align with **those reported in studies of human behaviour.**

[Introduction in Main Text, Page 2] Rather, it originates from the similarities between humans and LLM agents, encompassing the social networks they self-organize, the collective opinions they develop, **and the interaction processes through which these dynamics unfold.**

[Results in Main Text, Page 4] **(~~Mechanisms underlying the emergence of human-like~~) Collective behavioural patterns behind the emergence of** polarization among LLM agents.

[Results in Main Text, Page 4] This indicates that **LLM agents exhibit behavioural patterns consistent with the echo chamber effect**… Instead, exposure to agents with opposing opinions could potentially increase polarization levels, indicating **behavioural patterns consistent with the backfire effect [45].**

[Results in Main Text, Page 5] Rather, agents' **behavioural patterns consistent with these mechanisms** naturally emerge from agents' social interactions. For example, analyzing the **self-reported reasons** generated during opinion formation reveals **patterns in line with** confirmation bias (SI Section 3.7).

[Results in Main Text, Page 5] Given these observed similarities between LLM agents and humans at the network level, one may wonder whether LLM agents **exhibit behaviours consistent with psychological mechanisms discussed in studies of human opinion formation**. To answer this question, we design a comparative experiment: we **vary the proportion of agents exhibiting behaviours consistent with selective exposure or confirmation bias, and then examine the resulting changes** in the level of polarization. Cases where 0% or 100% of agents **are configured to exhibit these behaviours** are deliberately designed as extreme scenarios for comparative purposes. As shown in Figure 3e and f, we observe that the increase in agents **exhibiting either behaviour leads to a marked increase in the level of polarization characterizing the overall system**. **This suggests that behaviours related to selective exposure and confirmation bias** contribute to the polarization of LLM agents, **consistent with findings reported in studies of human behaviour** [48, 52, 79].

[Results in Main Text, Page 5] These agents **exhibit behaviours consistent with social phenomena and associated mechanisms discussed in studies of human behaviour, at both the network and individual levels.**

[Discussion in Main Text, Page 6] Specifically, from the individual perspective, opinion formation among LLM agents exhibits **behavioural patterns consistent with social and psychological mechanisms** [51, 52], e.g., selective exposure, confirmation bias, elite signaling, etc. From the collective perspective, LLM agents organically develop **social networks with properties characteristic of human social networks**, e.g., homophilic clustering and scale-free degree distributions [28]. Moreover, their self-developed social networks, in turn, shape LLM collective opinions **in ways consistent with** the echo chamber and backfire effects described in studies of human behaviour, resulting in the emergence of opinion polarization [43–48, 81, 85, 86]. Notably, all **these behavioural patterns**, network organization, and collective opinions naturally emerge from interactions among agents completely driven by LLMs.

[Discussion in Main Text, Page 6] Specifically, from the individual perspective, opinion formation among LLM agents exhibits **behavioural patterns consistent with social and psychological mechanisms** [51, 52], e.g., selective exposure, confirmation bias, elite signaling, etc. From the collective perspective, LLM agents organically develop **social networks with properties characteristic of human social networks**, e.g., homophilic clustering and scale-free degree distributions [28]. Moreover, their self-developed social networks, in turn, shape LLM collective opinions **in ways consistent with** the echo chamber and backfire effects described in studies of human behaviour, resulting in the emergence of opinion polarization [43–48, 81, 85, 86]. Notably, all **these behavioural patterns**, network organization, and collective opinions naturally emerge from interactions among agents completely driven by LLMs.

[Section 3.3 in Supplementary Information, Pages 15-16] We also incorporate more **standard measures of behaviours commonly associated with "echo chambers" and "backfire effects" following prior research** [9, 4]… As shown in Figure S10, we observe that agents are more likely to interact with peers in the same camp (left-leaning or right-leaning), **demonstrating behavioural patterns consistent with echo chambers**… Echo chambers do not universally occur across all individuals in the real world…Specifically, we find that **patterns of interaction consistent with echo chambers do not universally occur across all agents.**

[Section 3.3 in Supplementary Information, Pages 17] Similarly, the presence and extent of backfire effects remain subjects of debate, and such effects do not appear universally…Furthermore, **behavioural patterns consistent with the backfire effect** are observed in only a small portion of agents (0.82%–2.12%)…

# Minor Points

- The suggestion that "encouraging access and open-mindedness to diverse opinions" is an effective intervention is widely regarded as oversimplified and, in some contexts, misguided policy advice.

Response: We agree that statements suggesting "encouraging access and open-mindedness to diverse opinions" as an effective intervention could be misinterpreted as policy advice. In the revised manuscript, we have removed any suggestions of policy guidance. All intervention results are now explicitly presented as hypothesis-generating observations in a synthetic testbed, rather than as recommendations for real-world implementation.

Revision: [Abstract in Main Text, Page 1] …Similarities between humans and LLM agents—encompassing behaviours, patterns, and emergent phenomena—not only offer a valuable **synthetic testbed for generating hypothetical strategies to** mitigate polarization and its consequences, …

[Introduction in Main Text, Page 2] In this work, we simulate **a synthetic LLM-agent system,** where thousands of agents, solely driven by LLMs… the system we propose can constitute **a valuable pre-experimental testbed for exploring hypothetical strategies**

to reduce polarization and promote more inclusive political conversations [30]… Overall, this work not only sheds light on subtle opinion dynamics and collective behaviour of LLM agents from a new network perspective, but it also unveils **their potential to serve as a testbed for the pre-experimental exploration of opinion-dynamics hypotheses.**

[Results in Main Text, Page 5] **Synthetic testbed for generating hypothetical strategies to reduce polarization.**

[Results in Main Text, Page 5] These similarities make **the proposed LLM-agent system a valuable synthetic testbed for generating hypothetical strategies to** intervene in polarization and its consequences.

[Results in Main Text, Page 6] To explore it, we accordingly design five intervention strategies **in our synthetic testbed** (Figure 4)…Indeed, these interventions **are motivated by established lines of prior work and are used here as experimental interventions in our synthetic system**: MOI draws on studies of exposure to cross-cutting information [76, 82]; RI operationalizes randomized interaction; NSE and NCB relate to promoting open-mindedness [83]; and NES is inspired by the design of Bail et al. [84].

[Results in Main Text, Page 6] Despite the difficulty of recommending openness to human populations, these results suggest that **in our synthetic testbed**, access and open-mindedness to diverse opinions at the individual level appear more effective than altering social networks in polarized systems. . . In all, these experiments not only **generate several promising hypothetical strategies** but also demonstrate **the proposed system's potential as a valuable testbed** for piloting social experiments. **(~~Furthermore, these experiments provide guidance regarding how to directly intervene in the growing world of LLM agents operating in the wild.~~)**

[Discussion in Main Text, Page 7] (~~Our findings have direct implications across many domains. In the context of computational social science, the systematic similarities between LLM agents and humans—encompassing behaviours, mechanisms, and emergent phenomena—allow us to advance from traditional agent-based models by incorporating more human-like LLM agents in simulation studies [55, 65, 95–99]. LLM agents, without the need for any additional mechanisms or rules, can approximate the functioning of real-world complex systems through their free-form interactions. To this end, our study pioneers a first step by using LLM agents to model the emergence of polarization, a main focus of studies on complex social systems [55, 65, 95–97]. Echoing recent perspectives and efforts by social scientists [9, 10, 98–100], we believe that incorporating LLM agents into future social scientific studies is a very promising research direction, particularly in the context of complex adaptive social systems. Despite overlooking the modeling of adaptive dynamics in complex social systems, some recent studies have demonstrated the potential for LLM agents to generate social networks when provided with real-world demographic data [45, 46] or customized prompts [47, 48]. Furthermore, the proposed networked system of LLM agents, which systematically shares multi-scale similarities with human networks, provides valuable grounds for~~

~~piloting complex experiments. Due to costs, logistics, and ethical considerations, large-scale real-world experiments are not always practical [98]. Pre-experimental technologies that reduce the explosively large design space of social experiments identify promising directions and eliminate impractical strategies, enabling us to learn more. In this work, taking the proposed networked system of LLM agents as the pre-experimental ground, we identify several strategies for reducing polarization and promoting less divisive political conversations. These strategies have strong real-world foundations and can be readily transferred to human communication. For example, the intervention of neutral elite signaling (Figure 4f) could be implemented by deploying neutral, authoritative bots to serve as trusted messengers of balanced content. Moreover, the proposed system not only enables the direct adaptation of more sophisticated intervention designs from real-world human studies [89] (SI Section 3.6), but also serves as a robust testbed for theoretical exploration, facilitating the discovery of additional mitigation strategies, such as introducing "wiser" or more thoughtful individuals (SI Section 3.9) and incorporating minimal social friction (SI Section 3.10). However, it is important to acknowledge that this work remains a synthetic, simulation-based study, and there are gaps between LLM-driven agent interactions and real-world human communications. For example, the full richness of human behaviours [94] and nuanced perspectives of minority groups [69] may not be fully captured by current LLMs. Therefore, while the identified intervention strategies offer valuable insights, their applicability to highly heterogeneous human populations should be interpreted with caution. Future work should systematically validate these strategies in real-world settings and explore the potential of LLM-assisted pilot experiments to reduce biased perspectives and enhance representativeness in social scientific studies [98].~~)

[Discussion in Main Text, Page 6] While this work **does not make novel theoretical contributions**, it is grounded in established theories and models of opinion dynamics [58, 62, 100, 101]. In particular, this work follows the argument-accumulation lineage, which explains polarization as arising from the cumulative exchange and reinforcement of persuasive arguments [58, 60, 100, 101]…

- Assertions about LLMs as a "new species" (Homo silicus), their "uncontrollable" effects, or their policy implications are highly speculative and unsupported by the presented evidence.

<u>Response:</u> We have removed all speculative language referring to LLMs as a "new species" (e.g., Homo silicus), as well as claims about "uncontrollable" effects or policy implications. The revised manuscript avoids such terminology entirely and maintains a descriptive, non-speculative tone focused on the behaviour of agents in a controlled, synthetic testbed.

<u>Revision:</u> [Discussion in Main Text, Page 6] This work uncovers that LLM agents, **(~~sometimes considered a new species "Homo silicus" [12]~~)**, exhibit **(~~human-like~~)** polarization patterns on political issues, at both individual and collective levels.

- The discussion of bias and the proposed "self-regulation" strategy is superficial. The leftward drift is unsurprising, and the intervention likely masks rather than addresses the problem. More fundamentally, if the model were intended to represent bot networks, it is unclear why such a correction would be relevant.

Response: We thank the reviewer for this thoughtful comment. As clarified in the revised manuscript, the proposed self-regulation strategy is not introduced as a solution to real-world bias, nor as an intervention targeting bot networks. Instead, it is introduced as a method to ensure the behavioural plausibility and internal consistency of LLM agent, rather than to remove or correct their inherent tendencies.

Revision: [Results in Main Text, Page 4]…**It is worth noting that this strategy is introduced to ensure the behavioural plausibility and internal consistency of LLM agents, rather than to remove or correct their inherent tendencies**…

[Introduction in Main Text, Page 1] Besides these evident misbehaviours, **LLMs may pose broader risks in more subtle ways.** Numerous studies have pointed out that LLMs can generate persuasive political content [21–23], potentially affecting human opinions and behaviours [13, 23–25]. More crucially, due to the increasing indistinguishability of content generated by LLMs from that created by humans [4, 21, 22, 24, 26], concerns arise over their potential to manipulate public opinions and intensify polarization as the scale of interaction increases [24, 25, 27]. ~~**Indeed, this concern over digital manipulation of public opinions, which has amplified over the past decade [28–32], has been drastically exacerbated by the advent of LLM agents. During the 2016 US presidential election, automated agents, then known primarily as bots, were widely deployed on social media and suspected of interfering with electoral outcomes [28]. In the same year, Tay, a continuously learning bot released by Microsoft, was contaminated by biased human-generated content, resulting in offensive discourse and removal from the Internet within hours of its release [33]. Driven by simple rules, bots lacked human-level intelligence [28, 33], but their societal impacts remained profound, raising a pressing question: what if they were empowered by LLMs [4, 22, 24]?**~~

[Results in Main Text, Page 6] Despite the difficulty of recommending openness to human populations, these results suggest that **in our synthetic testbed**, access and open-mindedness to diverse opinions at the individual level appear more effective than altering social networks in polarized systems. . . In all, these experiments not only **generate several promising hypothetical strategies** but also demonstrate **the proposed system's potential as a valuable testbed** for piloting social experiments. **(~~Furthermore, these experiments provide guidance regarding how to directly intervene in the growing world of LLM agents operating in the wild.~~)**

[Discussion in Main Text, Page 6] Building on these findings, our work shifts the perspective from isolated LLMs [29, 30, 32, 35–38] to complex, networked LLM agent

societies, offering **a synthetic testbed for** studying emergent collective dynamics in artificial agents (~~**and their potential interactions with human societies**~~**).**

[Discussion in Main Text, Page 7] (~~On the other hand, LLM agents also raise concerns about their potential risks to human society. As studied in prior work [4, 21, 22, 24–26], LLMs are not only indistinguishable from humans but may also be more persuasive. Moreover, attempts to incorporate LLM agents into real-world political deliberations have demonstrated their surprising influence, with evidence indicating that they can drive significant outcomes even when functioning independently [35, 36, 43]. This is naturally concerning and raises fundamental questions with which modern societies and polities must grapple. What are the consequences of releasing LLMs with increasing autonomy, indistinguishable from humans, and with access to online social networks? How will such agents interplay with human social ties, be conflated with real friends, augment political persuasion, and impact our opinion dynamics? Moreover, unlike traditional bots [28–32], these LLM agents can evolve, gradually self-organizing their own collective opinions and social networks. The consequences of integrating such LLM agents at scale into human social networks are unpredictable and, in our view, still beyond our control. This requires us to rigorously evaluate potential risks, strengthen safeguards, and conduct cautious testing before deploying them "in the wild". First, we should systematically evaluate and mitigate biases in LLMs that drive agents to ensure fairness, reliability, and prevent unintended reinforcement of societal bias. This requires advancing standardized benchmarks, auditing frameworks, and bias mitigation strategies [17, 69, 70, 92, 93, 101]. Second, detection methods targeting LLM agents need further investigation to prevent them from engaging in deceptive or manipulative behaviour or disrupting human social media platforms [102]. Third, future work should extend experiments to a mixed population of both agents and humans, which could further facilitate more rigorous examination of LLM influence on human behaviours and society. Finally, because the collective nature of agents gives rise to emergent collective opinions and behaviours that cannot be easily inferred from individual agents, we believe that our proposed networked simulation experiments serve as a useful pre-deployment test for LLM agents. To further strengthen the rigor of such evaluations, future work should consider developing a controlled experimental platform that enables testing with mixed populations of LLM agents and humans.~~)

- Like many ABMs, the methodological design relies on arbitrary prompt engineering and simplistic opinion labels (e.g., a five-point left–right scale). It is unclear how these map onto real-world political cognition or how representative the resulting dynamics are.

Response: We acknowledge that the use of prompt-based agent design and simplified opinion labels (e.g., a five-point scale) abstracts away from the full complexity of real-world political cognition. We now explicitly discuss this limitation in the manuscript, emphasizing that these design choices are intended to support controlled exploration of collective dynamics rather than to provide a faithful representation of human political reasoning.

Revision: [Discussion in Main Text, Page 7]…In addition, compared to prior models with transparent mechanisms, GABM comes at the cost of reduced interpretability, **as agent behaviour is shaped through prompt-based design and simplified opinion representations**…

- The model relies heavily on LLM "self-reporting." Treating such outputs as equivalent to human self-reports is questionable, given known limitations of LLM-generated explanations.

Response: We agree that LLM-generated self-reports should not be treated as equivalent to human self-reports. In the revised manuscript, we have revised the language to explicitly note when interpretations are based on LLM self-reports. While we have no direct or deep knowledge about what LLMs "really think" regarding their motivations or experiences, as predictive models, they are engaged in predictive text-generation designed to construct the plausible (sounding) explanation, which we find both useful and evaluable for social science.

Revision: [Methods in Main Text, Page 8] It is worth noting that we do not rely on an external classifier to evaluate agents' opinions. Instead, we prompt the agents to self-report their opinions on political issues…(~~Notably, while external classifiers are often employed when direct responses are unavailable, self-reporting remains one of the most widely used and reliable method for assessing human political opinions [50, 53]~~)…

# Summary
This manuscript addresses an important and emerging topic. However, it substantially overstates its contributions, under-theorizes key concepts, and relies heavily on superficial analogies between LLM outputs and human behavior. It is unconvincing in its claims, inconsistent in its framing, and insufficiently rigorous.

Response: We appreciate the reviewer's engaged, careful and critical reading of our manuscript and prior reviews. In response, we have substantially revised the manuscript to address these concerns by clarifying the conceptual framing, tightening the language, explicitly acknowledging theoretical lineages and limitations, and avoiding overstatement of contributions. The revised manuscript now presents a more carefully scoped and methodologically transparent contribution as a synthetic, hypothesis-generating LLM-agent testbed.

We hereby submit a Revision of our paper "Emergence of polarization in networks of large language model agents" (**#NCOMMS-25-28403B-Z**). This manuscript has not been submitted for publication elsewhere.

We attach

- A point-by-point response to reviewers' comments,
- A revised manuscript with changes marked in blue and revisions in red, and
- A revised Supplementary Information with changes marked in blue.

Reviewer #4 (Remarks to the Author):

I appreciate the authors' thorough and constructive response and revisions of their manuscript. The revisions have addressed my main concerns: the manuscript is now much more clearly framed as a synthetic LLM-agent testbed rather than as a model of real-world bot networks or human opinion dynamics. The authors also now acknowledge that the results align with established models rather than constituting a novel theoretical contribution, and they have added important caveats regarding validation, calibration, external validity, and interpretability.

There are however still a few remnants left of the previous framing that I would prefer seeing addressed. I would only suggest that the authors temper a few remaining formulations, especially in the abstract and introduction. Phrases such as "similarities between humans and LLM agents" and claims that the system can generate hypothetical strategies to mitigate polarization still risk implying a stronger correspondence to human social dynamics than the paper establishes. I would encourage the authors to consistently phrase these points as "patterns consistent with established opinion-dynamics models" and to avoid grounding the contribution in broad human–LLM similarity. With this minor textual adjustment, I would be comfortable recommending publication.

I would like to congratulate the authors on their paper, and thank them for their efforts in addressing my concerns.

Response: We sincerely thank the Reviewer for the positive assessment of our revised manuscript and for recognizing that the major concerns raised in the previous round have been addressed. In particular, we appreciate your acknowledgement that the manuscript is now more clearly framed as a synthetic LLM-agent testbed, that the relationship between our findings and established opinion-dynamics models has been more appropriately characterized, and that additional caveats concerning validation, calibration, external validity, and interpretability have been incorporated.

We have further revised the manuscript following your remaining suggestions. Specifically, we have removed or tempered formulations that could imply broad similarities between humans and LLM agents, and we have revised claims regarding

intervention strategies to avoid suggesting that the system directly identifies strategies that can mitigate real-world polarization. We now consistently describe observed behaviours as patterns that are consistent with established opinion-dynamics models and existing empirical observations, while emphasizing the role of the framework as a synthetic testbed for exploring hypothetical intervention strategies. For your convenience, we paste the corresponding revisions below, with key points highlighted.

Revision: [Abstract in Main Text, Page 1] Rapid advances in large language models (LLMs) have not only empowered autonomous agents to generate social networks, communicate, and form shared and diverging opinions on political issues, but have also begun to play a growing role in shaping human political deliberation. Our understanding of their collective behaviours and underlying mechanisms remains incomplete, however, posing unexpected risks to human society. In this paper, we simulate networked systems involving thousands of LLM agents across different backbone models (GPT-3.5, GPT-4o, ChatGLM, Llama-3, and DeepSeek-V3), in which agents interact through LLM-guided conversations and update their opinions over time, resulting in the emergence of opinion polarization. We discover that these agents spontaneously develop their own social network with properties characteristic of human social networks, including homophilic clustering. The collective opinions of these LLM agents evolve in ways that exhibit behavioural patterns consistent with social phenomena and mechanisms widely discussed in studies of human behaviour. **Overall, these behaviours, patterns, and emergent phenomena produced by LLM agents are consistent with real-world observations and established opinion-dynamics models. This consistency suggests that LLM agents can serve as a valuable synthetic testbed for exploring hypothetical intervention strategies in networked LLM-agent systems.**

[Introduction in Main Text, Page 2] …This suggests that the emergence of opinion polarization among LLM agents is not a coincidence. Rather, it originates from **the consistency of LLM-agent behaviours with real-world observations and established models of human opinion dynamics**, as reflected in the social networks they self-organize, the collective opinions they develop, and the interaction processes through which these dynamics unfold. **This consistency suggests that the system we propose can constitute a valuable pre-experimental testbed for exploring hypothetical intervention strategies and their potential implications for polarization and political conversations** [30]…

[Results in Main Text, Pages 5-6] …The above results suggest that the emergence of polarization among LLM agents is not a coincidence. Instead, it emerges from **the consistency of LLM-agent behaviours with empirical observations and established models of human opinion dynamics**. These agents exhibit behaviours consistent with social phenomena and associated mechanisms discussed in studies of human behaviour, at both network and individual levels. **This consistency makes the proposed LLM-agent system a valuable synthetic testbed for generating hypothetical strategies to intervene in polarization and its consequences.…**
